# Alkynyl Thioethers in Gold‐Catalyzed Annulations To Form Oxazoles

**DOI:** 10.1002/anie.201706850

**Published:** 2017-09-19

**Authors:** Raju Jannapu Reddy, Matthew P. Ball‐Jones, Paul W. Davies

**Affiliations:** ^1^ School of Chemistry University of Birmingham Birmingham UK

**Keywords:** cycloaddition, gold, heterocycles, regioselectivity, sulfur

## Abstract

Non‐oxidative, regioselective, and convergent access to densely functionalized oxazoles is realized in a functional‐group tolerant manner using alkynyl thioethers. Sulfur‐terminated alkynes provide access to reactivity previously requiring strong donor‐substituted alkynes such as ynamides. Sulfur does not act in an analogous donor fashion in this gold‐catalyzed reaction, thus leading to complementary regioselective outcomes and addressing the limitations of using ynamides.

Compared to other heteroatom‐substituted alkynes, alkynyl thioethers are remarkably little explored in intermolecular late‐transition‐metal catalysis, despite being readily accessed and robust.^1^, ^2^ Ynamides, in contrast, are privileged substrates: in π‐acid catalysis their donor nature aids metal–alkyne coordination and affords highly polarized electrophiles, thus providing the high chemo‐ and regioselectivity required for the discovery of efficient intermolecular reactions (Scheme 1 a).^3^, ^4^ As the resulting inclusion of a donor‐nitrogen atom limits the utility of the products, retaining the reactivity profile of these transformations whilst accessing more flexible and readily elaborated substitution patterns would be desirable. The value of sulfur‐substituted compounds^5^ coupled with progress in C−C and C–heteroatom bond formation from C−S bonds,^6^ renders alkynyl thioethers appealing alternatives to ynamides. Indeed the ketenethionium pathway (Scheme 1 a) from alkynyl thioethers has recently been invoked in proton‐catalyzed reactions with nitriles2g,2h and gold‐catalyzed reactions with sulfides.2i

**Scheme 1:**
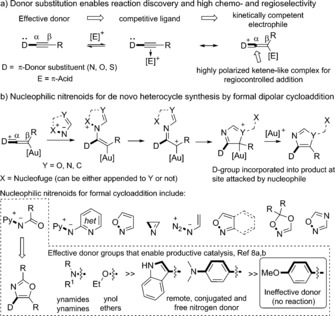
Donor substituent dictated reactivity and regioselectivity in π‐acid catalysis, and its application in enabling new cycloaddition reactions.

Ynamides enabled the discovery of formal [3+2] dipolar cycloadditions with nucleophilic nitrenoids,^7^ thus allowing intermolecular access to α‐imino gold carbene‐type reactivity for heterocycle synthesis (Scheme 1 b).^8^, ^9^ Such reactions, which do not depend on ynamides, are scarce.8b,8h A strong donor alkyne substituent proved critical in the formation of oxazoles using *N*‐acyl pyridinium *N*‐aminides, as electron‐rich alkynes such as anisole derivatives did not react (Scheme 1 b, inset).8a,8b Oxazoles are valuable synthetic intermediates^10^, ^11^ and structural components in bioactive natural products,^12^ agrochemicals,^13^ ligands,^14^ and functional materials.^15^ Despite recent advances, a single modular and convergent route to trisubstituted oxazoles, which provides the structural and functional‐group diversity needed across the 2‐, 4‐, and 5‐positions, remains unrealized.^16^

Following our interest in the use of gold catalysis with sulfides^17^ we report here on the reactivity of alkynyl thioethers with nucleophilic nitrenoids to prepare oxazoles. Importantly, the regioselectivity is not consistent with a controlling ketenethionium species. The sulfur group plays an alternative role in enabling reactivity, thus proving complementary to donor‐enabled approaches.

The reaction of the alkynyl thioether **1 a** and aminide **2 a** (Table 1) showed that conversion into the oxazole **3** was possible at 125 °C in 1,2‐dichlorobenzene (1,2‐DCB; see the Supporting Information for a survey of reaction conditions and pyridine‐modified aminides). No reaction was seen without catalyst, with dichloro(pyridine‐2‐carboxylato)gold being superior to other metal salts, including cationic gold and [Ir(cod)Cl]_2_.^1^ 5‐Methylthio‐oxazole (**3 aa**) was favored over 4‐methylthio‐oxazole (**3 aa′**) in all cases,^18^ thus contradicting the predicted outcome if sulfur were acting as a π‐donor substituent.

Effective reaction was seen with alkyl and aryl substitution at sulfur (Table 1, entries 1–5). Smaller *S* substituents gave improved conversion and higher regioselectivity. Conjugating the alkyne with a strongly electron‐withdrawing group shut down the reaction while an electron‐donating substituent saw smooth reactions and excellent regioselectivities across the *S*‐alkyl and *S*‐aryl series (entries 6–10). The sulfur substituent is critical, and the internal alkynes **4 a**/**b** did not react (entries 11 and 12).

**Table 1:** Scope of the reaction with respect to the alkynyl thioethers.^[a]^ 
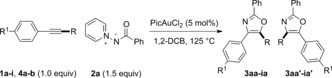

Entry	**1**	R	R^1^	*t* [h]	**3**	Yield [%] (**3**/**3′**)
1	**1 a**	SMe	H	24	**3 aa**	72 (8.4:1)
2	**1 b**	SEt	H	24	**3 ba**	70 (6.5:1)
3	**1 c**	S*i*Pr	H	24	**3 ca**	65 (4.5:1)
4	**1 d**	SPh	H	24	**3 da**	61 (4.8:1)
5	**1 e**	SBn	H	24	**3 ea**	51 (6.3:1)
6^[b]^	**1 f**	SMe	CO_2_Et	48	**3 fa**	‐
7	**1 g**	SEt	OMe	36	**3 ga**	64 (>20:1)
8^[b]^	**1 g**	SEt	OMe	24	**3 ga**	78 (>20:1)
9	**1 h**	SPh	OMe	24	**3 ha**	67 (15:1)
10	**1 i**	SMe	OMe	24	**3 ia**	73 (>20:1)
11	**4 a**	Ph	OMe	48	–	–
12	**4 b**	Me	OMe	48	–	–

[a] Reactions performed using alkynyl thioether (0.2 mmol) and PicAuCl_2_ (5 mol %), unless otherwise stated. Yields of the isolated regioisomers with the ratio determined by ^1^H NMR analysis. [b] PicAuCl_2_ (10 mol %).

Site‐specific nickel‐catalyzed cross‐coupling with MeMgBr saw conversion of the thio‐oxazoles **3**/**3′** into the known and separable methyl oxazoles **5 a**/**5 a′** or **5 b**, thus confirming preferential formation of 5‐thio‐oxazoles in the annulation (Scheme 2).^19^, ^20^ X‐ray diffraction subsequently confirmed the structures of **3 aa** and **3 ga** (see the Supporting Information).^21^ These first nickel‐mediated Kumada‐type couplings with 5‐thioether oxazoles^22^ demonstrate the value of the thioether handle, in this case providing substitution patterns which are not directly accessible from the annulation (see **4 b** in Table 1).

**Scheme 2:**
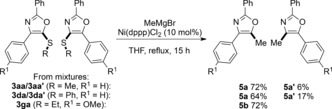
Nickel‐catalyzed Kumada cross‐coupling of thioether substituted oxazoles. dppp=1,3‐bis(diphenylphosphino)propane, THF=tetrahydrofuran.

The reactivity of alkynyl thioethers was evaluated across functionalized *N*‐acyl aminides (**2**, Scheme 3; accessible from carboxylic acids or esters in one step^23^). Broad functional‐group and structural tolerance was seen, with incorporation of electron‐rich and electron‐poor (hetero)aromatics, alkyl chains, acetals, aryl halides, Lewis bases, carbamates, aromatic and aliphatic amines, aromatic or enolisable carboxylic esters, and even a benzylic tertiary alcohol (0.2 to 3.0 mmol scale). Motifs found in bioactive compounds and natural products, such as peptidic oxazoles^24^ derived from aminides **2 h**–**k** and (3‐indolyl)oxazoles^12^ derived from the alkynyl thioether **1 n**, are readily prepared. The alkynyl thioether **1 j** gave 5‐thioethers **3 ja**/**c** as the major isomers, thus providing 4‐alkyl oxazoles. Sterically‐congested bi(hetero)aryl linkages may also be formed as single regioisomers (**3 lc**).^21^

**Scheme 3:**
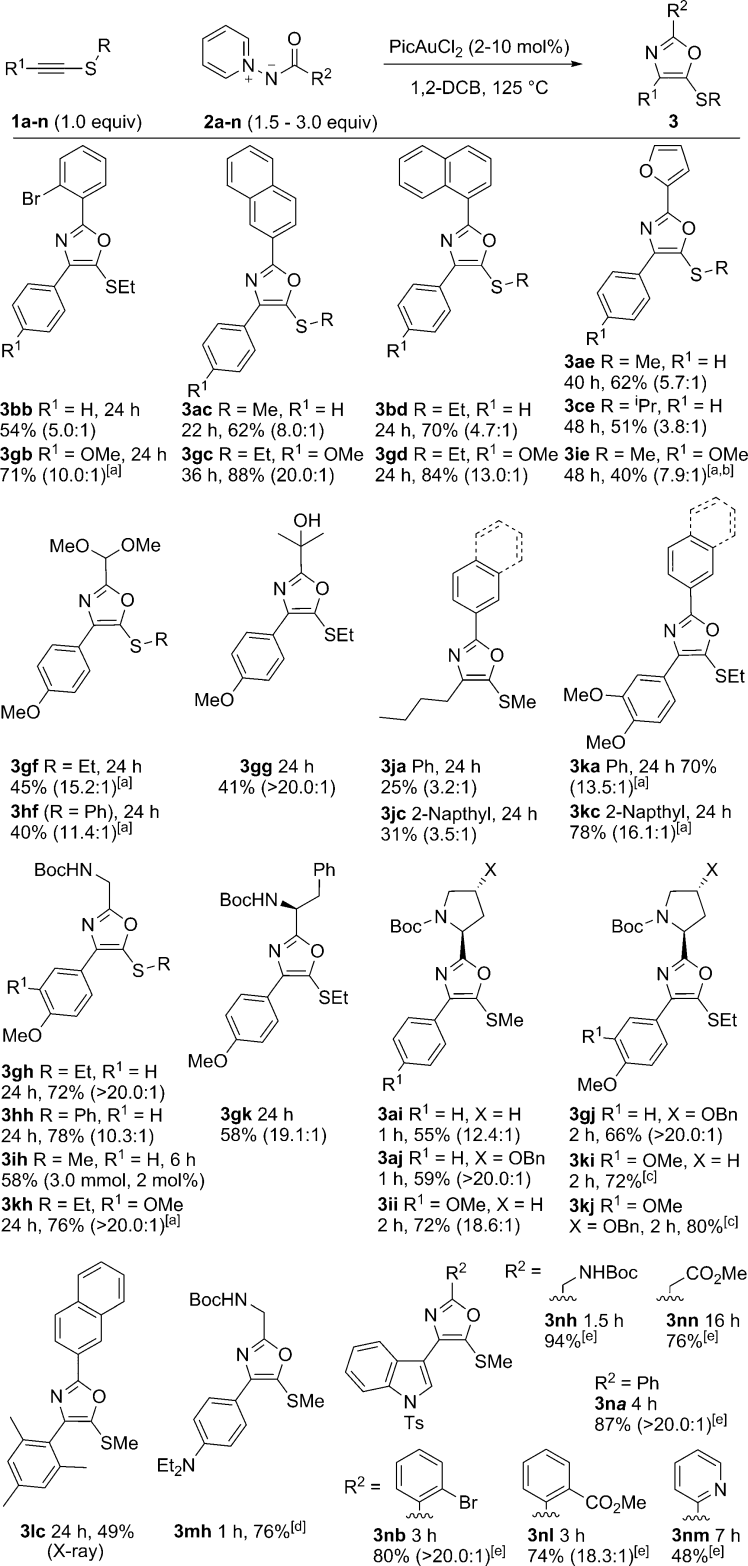
Intermolecular formal [3+2] dipolar cycloaddition of alkynyl thioethers with *N*‐acyl pyridinium *N*‐aminides. PicAuCl_2_ (5 mol %) unless otherwise mentioned. Shown are the yields of the isolated regioisomers with the ratio determined by ^1^H NMR analysis. [a] PicAuCl_2_ (10 mol %). [b] 2.0 equiv. of **2**. [c] Reaction carried out on 0.4 mmol scale. [d] 0.5 mmol scale. [e] 3.0 equiv. of **2**. Boc=*tert*‐butoxycarbonyl, Ts=4‐toluenesulfonyl.

The favored addition of the nitrenoid β to the sulfur atom (an inversion of regioselectivity compared to ynamides) is rationalized by a stabilizing Au−S interaction in the development of vinyl gold carbenoid **D^2^**, an interaction which is carried through into the to aurated heterocycle **E^2^** (Scheme 4). Three‐membered metal–S dative interactions are known,2c,2d though stabilizing hyperconjugative σ_C‐Au_ to σ*_C‐S_ interactions (**D^2^** inset) could also be invoked.^25^ Sulfur–gold coordination (**B**) may aid formation of a π‐activated complex in the presence of other effective ligands to the metal. Ground‐state perturbation of the alkyne–gold complex with slippage of gold toward sulfur (extreme form **C^2^**) is reinforced by more‐electron‐donating groups at R^1^. The aminide nitrogen atom reconfigures as the nucleofuge is extruded with cyclization, thus requiring the acyl group to move up toward the aurated carbon atom. The lower regioselectivities seen with larger acyl (**3 gc** vs. **3 gd**, Scheme 3) or sulfur substituents are consistent with the conformations imposed in **D^2^**. To maintain the S–Au interactions the sulfur substituent is positioned toward the approaching aminide, thus causing repulsive interactions.^26^

**Scheme 4:**
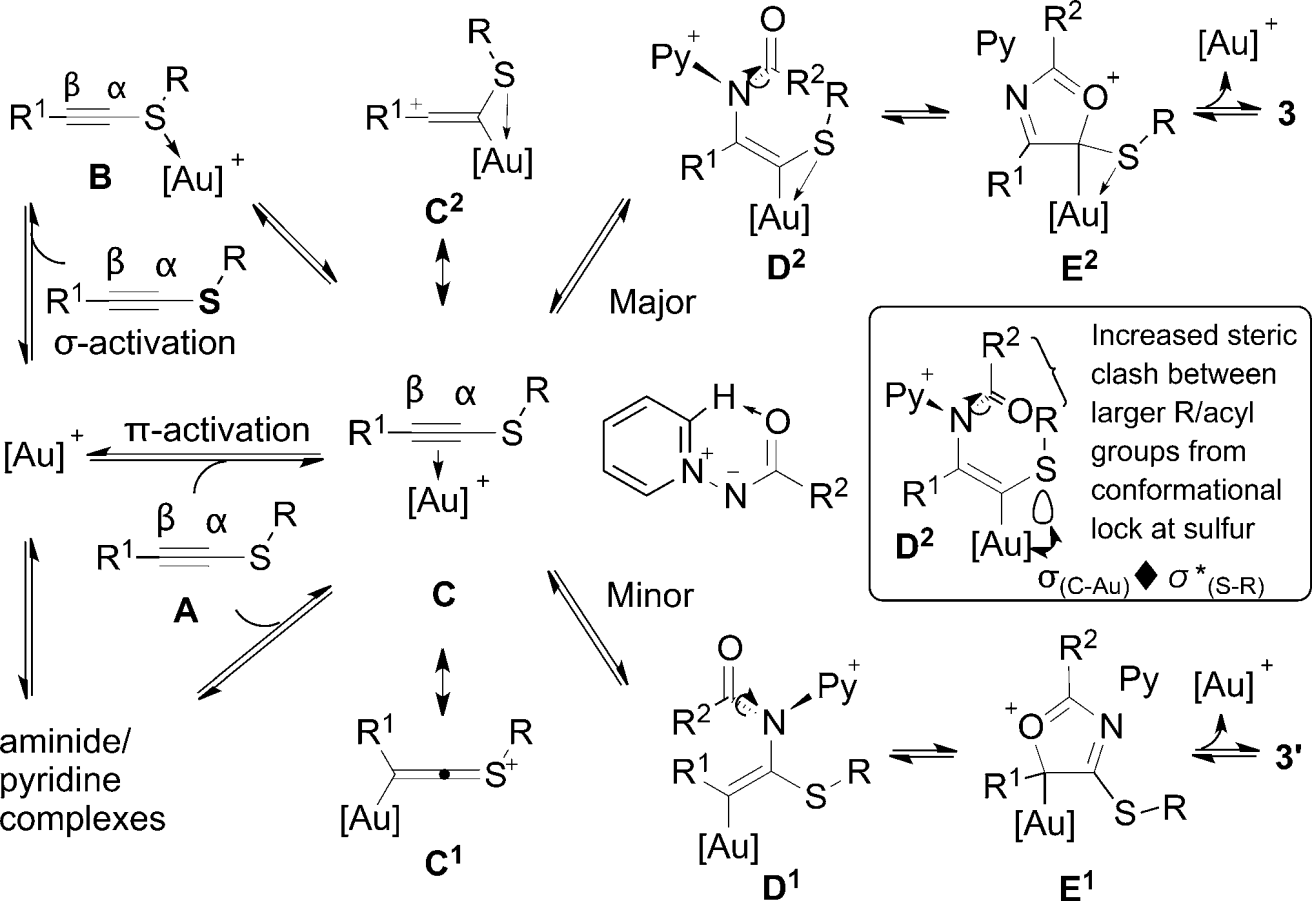
Proposed mechanistic rationale for the observed regioselectivity.

To rule out a controlling ketenethionium pathway in the gold‐catalyzed transformation, we attempted to access such an intermediate using Brønsted acid catalysis.2g,2h No reaction was seen between **1 a** and **2 a** in the presence of Tf_2_NH. Using the dioxazole **7** 8i,8m in place of **2 a** led to the formation of **3 aa′** and no trace of **3 aa** (Scheme 5). In the presence of a cationic Au^I^ catalyst **3 aa** was formed as the major isomer, thus ruling out the nitrenoid's role in switching regioselectivity. These preliminary results show the potential of alkynyl thioethers in regiodivergent heterocycle synthesis by selective application of gold or protic catalysis with nucleophilic nitrenoids.

**Scheme 5:**
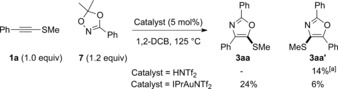
Regiodivergent synthesis of thio‐oxazoles using either gold or protic catalysis. [a] Yield of the material isolated after column chromatography, with further **3 aa′** contaminated with dioxalane **7**. Tf=trifluoromethanesulfonyl.

In summary, broad functional‐group and structural tolerance allows convergent and regioselective access into densely substituted oxazoles in the first example of gold‐catalyzed group‐transfer reactions onto alkynyl thioethers. Such alkynes are complementary to strong π‐donor‐substituted alkynes, and the sulfur is required for reactivity but gives inverted regioselectivity relative to the heteroatom, thus indicating that (metal)ketenethionium‐directed pathways invoked in other annulation processes do not apply here. Limitations from forming a donor‐atom‐substituted product are addressed by this approach, as demonstrated by the Kumada coupling with 5‐thioether‐oxazoles.

## Conflict of interest

The authors declare no conflict of interest.

## Supporting information

As a service to our authors and readers, this journal provides supporting information supplied by the authors. Such materials are peer reviewed and may be re‐organized for online delivery, but are not copy‐edited or typeset. Technical support issues arising from supporting information (other than missing files) should be addressed to the authors.

Supplementary

## References

[1] Review: V. J. Gray , J. D. Wilden , Org. Biomol. Chem. 2016, 14, 9695–9711. 27714241 10.1039/c6ob01776b

[2a] N. Riddell , W. Tam , J. Org. Chem. 2006, 71, 1934–1937; 16496978 10.1021/jo052295a

[2b] K.-H. Huang , M. Isobe , Eur. J. Org. Chem. 2014, 4733–4740;

[2c] S. Ding , G. Jia , J. Sun , Angew. Chem. Int. Ed. 2014, 53, 1877–1880; 10.1002/anie.201309855 24474668

[2d] Q. Luo , G. Jia , J. Sun , Z. Lin , J. Org. Chem. 2014, 79, 11970–11980; 25222638 10.1021/jo5018348

[2e] S. Ding , L.-J. Song , Y. Wang , X. Zhang , L. W. Chung , Y.-D. Wu , J. Sun , Angew. Chem. Int. Ed. 2015, 54, 5632–5635; 10.1002/anie.201500372 25784284

[2f] F. Nahra , S. R. Patrick , D. Bello , M. Brill , A. Obled , D. B. Cordes , A. M. Z. Slawin , D. O'Hagan , S. P. Nolan , ChemCatChem 2015, 7, 240–244; 26236406 10.1002/cctc.201402891 PMC4515107

[2g] L.-G. Xie , S. Shaaban , X. Chen , N. Maulide , Angew. Chem. Int. Ed. 2016, 55, 12864–12867; 10.1002/anie.201606604 27623988

[2h] L.-G. Xie , S. Niyomchon , A. J. Mota , L. Gonzalez , N. Maulide , Nat. Commun. 2016, 7, 10914; 26975182 10.1038/ncomms10914 PMC4796318

[2i] X. Shi , X. Ye , J. Wang , S. Ding , S. Hosseyni , L. Wojtas , N. Akhmedov , Chem. Eur. J. 2017, 23, 10506–10510. 28614600 10.1002/chem.201702710 PMC6139294

[3a] G. Evano , A. Coste , K. Jouvin , Angew. Chem. Int. Ed. 2010, 49, 2840–2859; 10.1002/anie.200905817 20354981

[3b] K. A. DeKorver , H. Li , A. G. Lohse , R. Hayashi , Z. Lu , Y. Zhang , R. P. Hsung , Chem. Rev. 2010, 110, 5064–5106; 20429503 10.1021/cr100003s PMC2927719

[3c] F. Pan , C. Shu , L. W. Ye , Org. Biomol. Chem. 2016, 14, 9456–9465. 27714280 10.1039/c6ob01774f

[4] π-Acid catalysis review: A. Fürstner , P. W. Davies , Angew. Chem. Int. Ed. 2007, 46, 3410–3449; 10.1002/anie.200604335 17427893

[5] B. R. Smith , C. M. Eastman , J. T. Njardarson , J. Med. Chem. 2014, 57, 9764–9773. 25255063 10.1021/jm501105n

[6a] L. Wang , W. He , Z. Yu , Chem. Soc. Rev. 2013, 42, 599–621; 23079733 10.1039/c2cs35323g

[6b] F. Pan , Z.-J. Shi , ACS Catal. 2014, 4, 280–288;

[6c] A. P. Pulis , D. J. Procter , Angew. Chem. Int. Ed. 2016, 55, 9842–9860; 10.1002/anie.201601540 27409984

[7] Review: P. W. Davies , M. Garzon , Asian J. Org. Chem. 2015, 4, 694–708.

[8] [3+2] modes:

[8a] P. W. Davies , A. Cremonesi , L. Dumitrescu , Angew. Chem. Int. Ed. 2011, 50, 8931–8935; 10.1002/anie.201103563 21793141

[8b] E. Chatzopoulou , P. W. Davies , Chem. Commun. 2013, 49, 8617–8619; 10.1039/c3cc45410j 23958931

[8c] M. Garzón , P. W. Davies , Org. Lett. 2014, 16, 4850–4853; 25203969 10.1021/ol502346d

[8d] A.-H. Zhou , Q. He , C. Shu , Y.-F. Yu , S. Liu , T. Zhao , W. Zhang , X. Lu , L.-W. Ye , Chem. Sci. 2015, 6, 1265–1271; 10.1039/c4sc02596b PMC5811108 29560212

[8e] L. Zhu , Y. Yu , Z. Mao , X. Huang , Org. Lett. 2015, 17, 30–33; 25514612 10.1021/ol503172h

[8f] Y. Wu , L. Zhu , Y. Yu , X. Luo , X. Huang , J. Org. Chem. 2015, 80, 11407–11416; 26503292 10.1021/acs.joc.5b02057

[8g] S. K. Pawar , R. L. Sahani , R.-S. Liu , Chem. Eur. J. 2015, 21, 10843–10850; 26094616 10.1002/chem.201500694

[8h] H. Jin , L. Huang , J. Xie , M. Rudolph , F. Rominger , A. S. K. Hashmi , Angew. Chem. Int. Ed. 2016, 55, 794–797; 10.1002/anie.201508309 26610069

[8i] M. Chen , N. Sun , H. Chen , Y. Liu , Chem. Commun. 2016, 52, 6324–6327; 10.1039/c6cc02776h 27086554

[8j] Y. Yu , G. Chen , L. Zhu , Y. Liao , Y. Wu , X. Huang , J. Org. Chem. 2016, 81, 8142–8154; 27569125 10.1021/acs.joc.6b01948

[8k] Z. Zeng , H. Jin , J. Xie , B. Tian , M. Rudolph , F. Rominger , A. S. K. Hashmi , Org. Lett. 2017, 19, 1020–1023; 28218539 10.1021/acs.orglett.7b00001

[8l] Y. Zhao , Y. Hu , X. Li , B. Wan , Org. Biomol. Chem. 2017, 15, 3413–3417; 28383597 10.1039/c7ob00701a

[8m] Y. Zhao , Y. Hu , C. Wang , X. Li , B. Wan , J. Org. Chem. 2017, 82, 3935–3942. 28276692 10.1021/acs.joc.7b00076

[9] Related reactivity for other cycloadditions:

[9a] C. Shu , Y.-H. Wang , C.-H. Shen , P.-P. Ruan , X. Lu , L.-W. Ye , Org. Lett. 2016, 18, 3254–3257; 27331406 10.1021/acs.orglett.6b01503

[9b] J. González , J. Santamaría , A. L. Suárez-Sobrino , A. Ballesteros , Adv. Synth. Catal. 2016, 358, 1398–1403;

[9c] M. S. H. Jin , M. S. B. Tian , M. S. X. Song , J. Xie , M. Rudolph , F. Rominger , A. S. K. Hashmi , Angew. Chem. Int. Ed. 2016, 55, 12688–12692; 10.1002/anie.201606043 27629266

[9d] W.-B. Shen , X.-Y. Xiao , Q. Sun , B. Zhou , X.-Q. Zhu , J.-Z. Yan , X. Lu , L.-W. Ye , Angew. Chem. Int. Ed. 2017, 56, 605–609; 10.1002/anie.201610042 27936302

[9e] R. L. Sahani , R.-S. Liu , Angew. Chem. Int. Ed. 2017, 56, 1026–1030; 10.1002/anie.201610665 27981725

[10a] V. S. C. Yeh , Tetrahedron 2004, 60, 11995–12042;

[10b] X. Zhang , X. Sun , H. Fan , L. Chang , P. Li , H. Zhang , W. Rao , RSC Adv. 2016, 6, 56319–56322.

[11] The Chemistry of Heterocyclic Compounds, Oxazoles: Synthesis Reactions and Spectroscopy, Parts A & B (Ed.: D. C. Palmer), Wiley-Interscience, Hoboken, NJ, 2004.

[12a] P. Wipf , S. Venkatraman , J. Org. Chem. 1996, 61, 6517–6522; 11667514 10.1021/jo960891m

[12b] J. R. Davies , P. D. Kane , C. J. Moody , A. M. Z. Slawin , J. Org. Chem. 2005, 70, 5840–5851; 16018676 10.1021/jo050303h

[12c] Z. Jin , Nat. Prod. Rep. 2013, 30, 869–915; 23644572 10.1039/c3np70006b

[12d] N. David , R. Pasceri , R. R. A. Kitson , A. Pradal , C. J. Moody , Chem. Eur. J. 2016, 22, 10867–10876. 27346186 10.1002/chem.201601605

[13a] Q. Zhao , S. Liu , Y. Li , Q. Wang , J. Agric. Food Chem. 2009, 57, 2849–2855; 19271709 10.1021/jf803632t

[13b] F. Grundmann , V. Dill , A. Dowling , A. Thanwisai , E. Bode , N. Chantratita , R. Ffrench-Constant , H. B. Bode , Beilstein J. Org. Chem. 2012, 8, 749–752. 23015823 10.3762/bjoc.8.85 PMC3388863

[14] J. Mazuela , P. Tolstoy , O. Pamies , P. G. Andersson , M. Dieguez , Org. Biomol. Chem. 2011, 9, 941–946. 21152643 10.1039/c0ob00656d

[15] V. T. T. Huong , T. B. Tai , M. T. Nguyen , J. Phys. Chem. A 2014, 118, 3335–3343. 24720499 10.1021/jp500899k

[16] Review:

[16a] S. Bresciani , N. C. O. Tomkinson , Heterocycles 2014, 89, 2479–2543; For recent examples, see:

[16b] T.-T. Zeng , J. Xuan , W. Ding , K. Wang , L.-Q. Lu , W.-J. Xiao , Org. Lett. 2015, 17, 4070–4073; 26250789 10.1021/acs.orglett.5b01994

[16c] P. Querard , S. A. Girard , N. Uhlig , C.-J. Li , Chem. Sci. 2015, 6, 7332–7335. 10.1039/c5sc02933c PMC5950834 29861964

[17] M. Dos Santos , P. W. Davies , Chem. Commun. 2014, 50, 6001–6004 and references therein. 10.1039/c4cc01059k 24763977

[18] 4-Methylthiooxazole synthesis: A. Herrera , R. Martínez-Alvarez , P. Ramiro , D. Molero , J. Almy , J. Org. Chem. 2006, 71, 3026–3032. 16599597 10.1021/jo052619v

[19] G. Zhu , W. Kong , H. Feng , Z. Qian , J. Org. Chem. 2014, 79, 1786–1795. 24494629 10.1021/jo4028402

[20] For structural comparisons see Ref. [16b]; and for **5 a**, see:

[20a] Y.-m. Pan , F.-j. Zheng , H. - x. Lin, Z.-p. Zhan, J. Org. Chem. **2009**, 74, 3148–3151; for **5 a′**, see:10.1021/jo8027533 19354325

[20b] M. Keni , J. J. Tepe , J. Org. Chem. 2005, 70, 4211–4213. 15876123 10.1021/jo0501590

[21] CCDC 1537012–1537014 contain the supplementary crystallographic data for compounds **3 aa**, **3 ga**, and **3 lc** These data can be obtained free of charge from The Cambridge Crystallographic Data Centre.

[22] Oxazole thioethers in cross-couplings, see:

[22a] L. N. Pridgen , Synthesis 1984, 1047–1048;

[22b] K. Lee , C. M. Counceller , J. P. Stambuli , Org. Lett. 2009, 11, 1457–1459. 19239243 10.1021/ol900260g

[23] A. D. Gillie , R. J. Reddy , P. W. Davies , Adv. Synth. Catal. 2016, 358, 226–239.

[24] Boc removal for **3 ki** and **3 kj** (trifluoroacetic acid/CH_2_Cl_2_, RT, 2 h) affords elaborated pyrrolidin-2-yl oxazoles in 82 and 73 % yield, respectively.

[25] B. R. Beno , K.-S. Yeung , M. D. Bartberger , L. D. Pennington , N. A. Meanwell , J. Med. Chem. 2015, 58, 4383–4438. 25734370 10.1021/jm501853m

[26] An analogous argument applies for an inner-sphere *syn* addition of gold and aminide across the alkyne as the stabilizing S–Au interactions would position the S substituent toward the alkyne C substituent.

