# Supplementary material for: Alkynyl Thioethers in Gold‐Catalyzed Annulations To Form Oxazoles
Source: Angew Chem Int Ed Engl. 2017 Sep 19;56(43):13310–3. doi: 10.1002/anie.201706850 (PMC5656920; doi:10.1002/anie.201706850)

Angewandte Chemie
Eine Zeitschrift der Gesellschaft Deutscher Chemiker

Supporting Information

Alkynyl Thioethers in Gold-Catalyzed Annulations to form Oxazoles

*Raju Jannapu Reddy, Matthew P. Ball-Jones, and Paul W. Davies**

anie_201706850_sm_miscellaneous_information.pdf

Author Contributions

R.J. Conceptualization: Supporting; Formal analysis: Equal; Funding acquisition: Supporting; Investigation: Lead; Methodology: Lead; Visualization: Equal; Writing – original draft: Supporting; Writing – review & editing: Supporting
M.B. Formal analysis: Equal; Investigation: Supporting; Methodology: Supporting; Validation: Lead; Visualization: Supporting; Writing – review & editing: Supporting
P.D. Conceptualization: Lead; Formal analysis: Equal; Funding acquisition: Lead; Investigation: Supporting; Methodology: Equal; Project administration: Lead; Resources: Lead; Supervision: Lead; Validation: Supporting; Visualization: Equal; Writing – original draft: Lead; Writing – review & editing: Lead.

Contents

General Experimental 2
Experimental Procedures and Spectroscopic Data 3
Synthesis of Starting Materials 3
Synthesis of Alkynyl Thioethers 3
Synthesis of Internal Alkynes 6
Synthesis of Pyridinium-*N*-Aminides 7
Survey of Reaction Conditions 10
Synthesis of 5-Thio-oxazoles 10
Reactions of Cycloaddition Adducts 23
Stability Studies of Chiral Pyrrolidine Derived Aminides 25
Single Crystal X-Ray Data Analysis 25
References 27
^{1}H NMR and ^{13}C NMR Spectra of New Compounds 28

General Experimental

All reagents were purchased from Sigma-Aldrich, Fluka, Fisher, Acros, Alfa Aesar or VWR and used without further purification unless otherwise stated. Dichloro(2-pyridincarboxylato)gold(III) (Au-I) was purchased from Sigma-Aldrich. All catalysis reactions and other reactions using dried or anhydrous solvents were carried out under inert atmosphere in glassware dried using a heat gun at high vacuum. Masses of reactants, catalysts and products were measured using an AND GH-252 balance with a standard deviation of 0.03 mg and ±0.10 mg linearity. Solvents were degassed and dried using a Pure Solv-MD solvent purification system and transferred under inert atmosphere unless otherwise stated. 1,4-Dioxane and 1,2-dichlorobenzene were dried over activated 4 Å molecular sieves. For reactions above room temperature pre-heated paraffin oil baths or Asynt Dry Syn heating blocks on stirrer hotplates were employed and the temperature was controlled using an external probe. The following cooling baths were used: 0 °C (ice/water) and -78 °C (dry ice/acetone). Reactions were monitored by thin layer chromatography (TLC) using Merck silica gel 60 F254 (aluminium support) TLC plates. Visualisation was achieved by a combination of ultraviolet light (254 nm) and potassium permanganate/Δ solution. Flash column chromatography was performed using Merck Geduran Si 60 (40-63 µm) silica gel as the stationary phase.

Melting points were measured in open capillaries using Stuart Scientific melting point apparatus and are uncorrected. Specific rotation is given as follows: (in deg $dm^{-1}cm^{3}g^{-1}$), ***c*** (in g/100 mL) solvent used. Infra-red spectra were recorded neat on a Perkin-Elmer Spectrum 100 FTIR spectrometer. Wavelengths of selected absorbencies are reported (ν_{max}) in cm^{-1}. ^{1}H NMR and ^{13}C NMR experiments were recorded in commercial, TMS free, deuterated solvents using Bruker AV300 (^{1}H = 300 MHz, ^{13}C = 75 MHz), AVIII400 (^{1}H = 400 MHz, ^{13}C = 101 MHz) or AVIII300 (^{1}H = 300 MHz, ^{13}C = 75 MHz), with the spectrometers at 300 K. ^{13}C NMR spectra were recorded using the UDEFT, PENDANT or JMOD pulse sequences from the Bruker standard pulse program library. Chemical shifts (*δ*) are given in ppm relative to TMS calibrated by reference to the residual solvent[1] and coupling constants (*J*) are quoted in Hz to one decimal place. 2D ^{13}C NMR HSQC and HMBC spectra were recorded using the Bruker standard pulse program library. Spectral data for ^{1}H NMR spectroscopy is reported as follows: Chemical shift (multiplicity, coupling constant, number of protons); and for ^{13}C NMR spectroscopy: Chemical shift. The following abbreviations were used for multiplicity in ^{1}H NMR: s (singlet), d (doublet), t (triplet), q (quadruplet), dd (doublet of doublets), td (triplet of doublets), quin (quintuplet), br (broad), m (multiplet), app. (apparent). All NMR spectra are processed using MestReNova version 10.0. Mass spectra were obtained using Waters GCT Premier (EI), Waters LCT (ES) or Waters Synapt (ES) spectrometers. High resolution spectra used a lock-mass to adjust the calibrated mass scale.

Experimental Procedures and Spectroscopic Data

Synthesis of Starting Materials

Synthesis of Alkynyl Thioethers

The following alkynyl thioethers were synthesised using literature methods to give spectroscopic data matching that reported: *Methyl(2-phenylethynyl)sulfide*[2] (**1a**, 80%), *ethyl(2-phenylethynyl)sulfide*[2] (**1b**, 78%), *isopropyl(2-phenylethynyl)sulfide*[2] (**1c**, 30%), *phenyl(phenylethynyl)sulfide*[3] (**1d**, 75%), *benzyl(2-phenylethynyl)sulfide*[2] (**1e**, 45%), *hexa-1-ynyl (methyl)sulfide*[2] (**1j**, 65%). Other alkynyl thioethers were prepared as described below.

Synthesis of precursors

Terminal alkynes (**TA1-5**) were synthesised using literature methods to give spectroscopic data matching that reported: *Methyl 4-ethynylbenzoate*[4] (**TA1** 91% over 2 steps) *1-ethynyl-4-methoxybenzene*[5] (**TA2** 57% over 2 steps); *4-ethynyl-1,2-dimethoxybenzene*[6] (**TA3** 87% over 2 steps); *2-ethynyl-1,3,5-trimethylbenzene*[7] (**TA4** 53% over 2 steps); *3-ethynyl-1-tosyl-1H-indole*[8] (**TA5** 71% from indole (4 steps)).

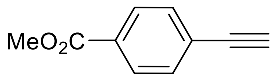

TA1

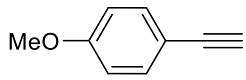

TA2

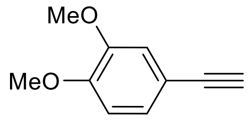

TA3

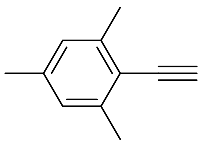

TA4

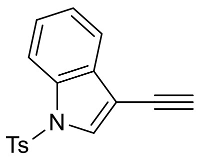

TA5

4-(2,2-Dibromovinyl)-N,N-diethylaniline[9] (56%) was synthesised using a literature method to give spectroscopic data matching that reported.[9]

The following benzenethiosulfonates (Scheme S1) were synthesised using literature methods to give spectroscopic data matching that reported: *S-Methyl benzenethiosulfonate*[10] (92%); *S-ethyl benzenethiosulfonate*[4] (88%); *S-methyl 4-methylbenzenesulfonothioate*[11] (84%); *S-phenyl benzenethiosulfonate*[12] (82%).

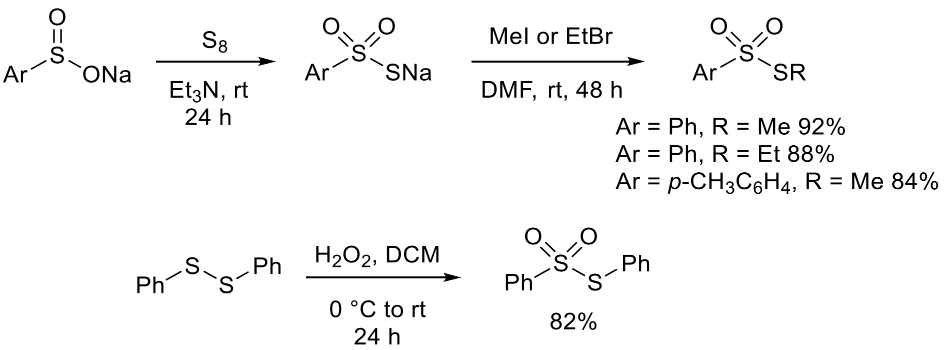

Scheme S1 Synthesis of benzenethiosulfonates

General Procedure (Scheme 2) for the preparation of alkynyl thioethers (GP1):

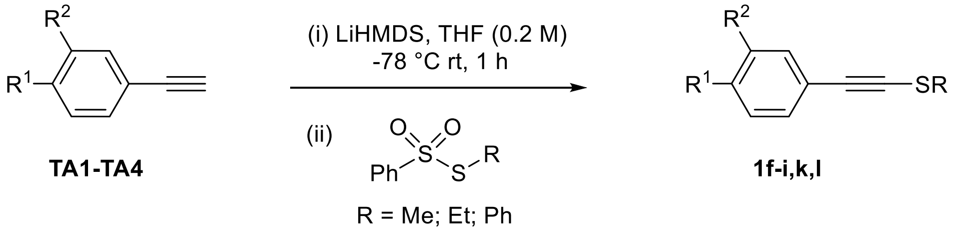

LiHMDS (1 M in THF, 1.1 equiv.) or BuLi (2.5 M in hexane, 1.1. equiv.) was slowly added to a stirred solution of alkyne (1 equiv.) in dry THF (0.2 M) over 10 min at -78 °C. After 15 min, the lithium acetaminide was treated with the corresponding benzene thiosulfonate ($PhSO_2SEt$, $PhSO_2SMe$ or $PhSO_2SPh$, 1.1 equiv.) in dry THF (1.1 M) which was added drop-wise over 15 min and the solution was then stirred for a further 15 min at -78 °C. The mixture was allowed to warm to room temperature and stirred for 1 h. The reaction mixture was quenched with satd. $NH_4Cl_{(aq)}$ and then extracted with diethyl ether (2 × 40 mL). The combined organic layers were dried over Na_2SO_4, filtered and concentrated under reduced pressure to give a residue which was purified by silica gel flash column chromatography to afford the desired alkynyl thioethers.

Methyl 4-[(methylthio)ethynyl]benzoate (1f):

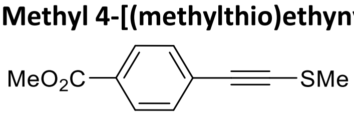

Following **GP1**, using methyl 4-ethynylbenzoate **TA1** (320 mg, 2.0 mmol) in dry THF (10 mL), LiHMDS (2.2 mL, 1 M in THF, 2.2 mmol) and then *S*-methyl benzenethiosulfonate (414 mg, 2.2 mmol) in dry THF (2 mL). Purification by flash column chromatography (3% then 5% EtOAc in hexane) afforded the alkynyl thioether **1f** as a pale-yellow liquid (288 mg, 70%); mp. 86-88 °C; IR (neat): *v* = 2993, 2947, 2158, 1706, 1600, 1597, 1435, 1274, 1252, 1175, 1113, 1014, 961, 852, 767, 697 cm^{-1}; ^1H NMR (300 MHz, $CDCl_3$): δ = 7.95 (d, *J* = 8.5 Hz, 2H), 7.43 (d, *J* = 8.5 Hz, 2H), 3.90 (s, 3H), 2.50 (s, 3H); ^{13}C NMR (101 MHz, $CDCl_3$): δ = 166.7, 131.0 (2C), 129.6 (2C), 129.1, 128.2, 91.6, 85.2, 52.3, 19.5; HRMS (ES): *m/z* calculated for $C_{11}H_{10}O_2S$: 206.0402, found 206.0399 $[M]^+$.

Ethyl(4-methoxyphenyl)ethynylsulfide (1g):

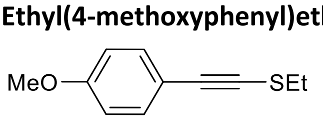

Following **GP1**, using 1-ethynyl-4-methoxybenzene **TA2** (330 mg, 2.5 mmol) in dry THF (12.5 mL), LiHMDS (2.8 mL, 1 M in THF, 2.8 mmol) and then *S*-ethyl benzenethiosulfonate (555 mg, 2.75 mmol) in dry THF (3.0 mL). Purification by flash column chromatography (5% EtOAc in hexane) afforded the alkynyl thioether **1g** as a pale-yellow liquid (428 mg, 89%); IR (neat): *v* = 2963, 2927, 2164, 1604, 1505, 1440, 1288, 1247, 1171, 1031, 831, 778 cm^{-1}; ^1H NMR (300 MHz, $CDCl_3$): δ = 7.38 (d, *J* = 8.9 Hz, 2H), 6.83 (d, *J* = 8.9 Hz, 2H), 3.81 (s, 3H), 2.80 (q, *J* = 7.3 Hz, 2H), 1.44 (t, *J* = 7.3 Hz, 3H); ^{13}C NMR (101 MHz, $CDCl_3$): δ = 159.7, 133.5 (2C), 115.8, 114.0 (2C), 93.3, 77.3, 55.4, 30.2, 14.9; HRMS (ES): *m/z* calculated for $C_{11}H_{12}OS$: 192.0609, found 192.0602 $[M]^+$.

4-(Methoxyphenyl)ethynylphenylsulfide (1h):

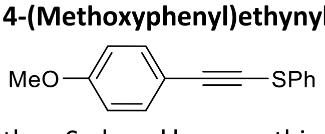

Following **GP1**, using 1-ethynyl-4-methoxybenzene **TA2** (264 mg, 2.0 mmol) in dry THF (10 mL), LiHMDS (2.2 mL, 1 M in THF, 1.1 mmol) and then *S*-phenyl benzenethiosulfonate (550 mg, 2.2 mmol) in dry THF (2 mL). Purification by flash column chromatography (10% EtOAc in hexane) afforded the alkynyl thioether **1h** as a colourless solid (410 mg, 85%); mp. 34-36 °C; IR (neat): *v* = 3006, 2836, 2169, 1600, 1505, 1473, 1287, 1245, 1180,

1107, 1022, 824, 745 cm^{-1}; ^{1}H NMR (300 MHz, $CDCl_3$): δ = 7.52-7.45 (m, 4H), 7.35 (app. t, *J* = 7.3 Hz, 2H), 7.22 (tt, *J* = 7.3, 1.2 Hz, 1H), 6.88 (d, *J* = 8.9 Hz, 2H), 3.83 (s, 3H); ^{13}C NMR (101 MHz, $CDCl_3$): δ = 160.2, 133.9 (2C), 133.6, 129.3 (2C), 126.5, 126.2 (2C), 115.1, 114.2 (2C), 98.1, 73.6, 55.5; HRMS (ES): *m/z* calculated for $C_{15}H_{12}OS$: 240.0609, found 240.0612 $[M]^+$.

4-(Methoxyphenyl)ethynylmethylsulfide (1i):

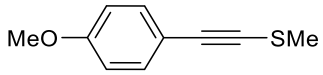

Following **GP1**, using 1-ethynyl-4-methoxybenzene **TA2** (0.66 g, 5.0 mmol) in dry THF (10 mL), *n*-BuLi (2.5 M in hexane, 2.20 mL, 5.5 mmol, 1.1 equiv.) and then *S*-methyl 4-methylbenzenesulfonothioate (1.29 g, 6.4 mmol, 1.3 equiv.) was added in a single portion. Purification by flash column chromatography (4% EtOAc in hexane) afforded the alkynyl thioether **1i** as a pale yellow oil (0.86 g, 96%); IR (neat): *v* = 2927, 2167, 1603, 1568, 1504, 1461, 1288, 1244, 1170, 1029 cm^{-1}; δ = 7.37 (d, *J* = 8.9 Hz, 2H), 6.82 (d, *J* = 8.9 Hz, 2H), 3.80 (s, 3H), 2.46 (s, 3H); ^{13}C NMR (101 MHz, $CDCl_3$): δ = 159.8, 133.5 (2C), 115.6, 114.0 (2C), 91.7, 79.1, 55.4, 19.6; HRMS (EI): *m/z* calculated for $C_{10}H_{10}OS$: 178.0452, found 178.0448 $[M]^+$.

(3,4-Dimethoxyphenyl)ethynylethylsulfide (1k):

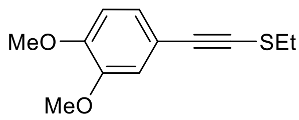

Following **GP1**, using 4-ethynyl-1,2-dimethoxybenzene **TA3** (486 mg, 3.0 mmol) in dry THF (15 mL), LiHMDS (3.3 mL, 1 M in THF, 1.1 mmol) and then *S*-ethyl benzenethiosulfonate (667 mg, 3.3 mmol) in dry THF (3 mL). Purification by flash column chromatography (5% EtOAc in hexane) afforded the alkynyl thioether **1k** as a pale-yellow liquid (600 mg, 90%); IR (neat): *v* = 2961, 2929, 2162, 1597, 1509, 1440, 1260, 1238, 1163, 1135, 1023, 960, 852, 806, 759 cm^{-1}; ^{1}H NMR (300 MHz, $CDCl_3$): δ = 7.04 (dd, *J* = 6.4, 1.9 Hz, 1H), 6.94 (d, *J* = 1.9 Hz, 1H), 6.78 (d, *J* = 8.3 Hz, 1H), 3.88 (s, 3H), 3.86 (s, 3H), 2.80 (q, *J* = 7.3 Hz, 2H), 1.44 (t, *J* = 7.3 Hz, 3H); ^{13}C NMR (101 MHz, $CDCl_3$): δ = 149.6, 148.7, 125.3, 115.8, 114.7, 111.1, 93.5, 77.4, 56.0 (2C), 30.2, 14.9; HRMS (ES): *m/z* calculated for $C_{12}H_{15}O_2S$: 223.0793, found 223.0790 $[M+H]^+$.

Mesitylethynyl(methyl)sulfide (1l):

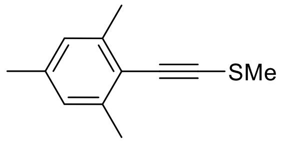

Following **GP1**, using 2-ethynyl-1,3,5-trimethylbenzene **TA4** (288 mg, 2.0 mmol) in dry THF (10 mL), LiHMDS (2.2 mL, 1 M in THF, 1.1 mmol) and then *S*-methyl benzenethiosulfonate (413 mg, 2.2 mmol) in dry THF (2 mL). Purification by flash column chromatography (3% EtOAc in hexane) afforded the alkynyl thioether **1l** as a pale-yellow liquid (320 mg, 84%); IR (neat): *v* = 2921, 2853, 2158, 1609, 1509, 1473, 1433, 1375, 1311, 1233, 1033, 975, 850, 725 cm^{-1}; ^{1}H NMR (300 MHz, $CDCl_3$): δ = 6.85 (s, 2H), 2.50 (s, 3H), 2.38 (s, 6H), 2.27 (s, 3H); ^{13}C NMR (101 MHz, $CDCl_3$): δ = 140.1 (2C), 137.5, 127.7 (2C), 120.3, 90.0, 87.7, 21.4, 21.1 (2C), 20.2; HRMS (EI): *m/z* calculated for $C_{12}H_{14}S$: 190.0816, found 190.0821 $[M]^+$.

***N,N*-Diethyl-4-((methylthio)ethynyl)aniline (1m)**

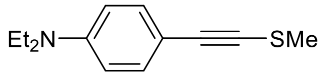

THF (10 mL) was added to 4-(2,2-dibromovinyl)-N,N-diethylaniline (0.53 g, 1.6 mmol) and was then cooled to -78 °C. After 15 minutes *n*BuLi (2.5 M in hexane, 0.63 mL, 1.6 mmol) was added over 5 minutes and the reaction was stirred for 40 minutes at -78 °C. A second portion of *n*BuLi (2.5 M in hexane, 0.63 mL, 1.6 mmol, 1.05 equiv.) was added over 5 minutes and then the reaction was stirred at -78 °C for 50 minutes. *S*-Methyl 4 -methylbenzenesulfonothioate (0.33 g, 1.6 mmol) was added as a solution in THF (3.3 mL) and the

reaction was then allowed to stir at r.t. for 40 minutes. After this time, the reaction was quenched with a saturated NH_4Cl solution (10 mL) and the product was extracted with Et_2O (2 × 10 mL). The combined organic layers were dried over Na_2SO_4, filtered and concentrated under reduced pressure to give a residue which was purified by silica gel flash column chromatography (5% EtOAc in hexane) to afford alkynyl thioether **1m** as pale yellow solid (0.23 g, 66%); mp 26-28 °C; IR (neat): *ν* = 2969, 2926, 2153, 1603, 1466, 1373, 1189, 814 cm^{-1}; ^1H NMR (300 MHz, $CDCl_3$): δ = 7.30 (d, *J* = 9.0 Hz, 2H), 6.56 (d, *J* = 9.0 Hz, 2H), 3.35 (q, *J* = 7.1 Hz, 4H), 2.44 (s, 3H), 1.16 (t, *J* = 7.1 Hz, 6H); ^{13}C NMR (101 MHz, $CDCl_3$): δ = 147.8, 133.8 (2C), 111.2 (2C), 109.0, 93.1, 77.2, 44.4 (2C), 19.9, 12.6 (2C); HRMS (EI): *m/z* calculated for $C_{13}H_{17}NS$: 219.1082, found 219.1081 $[M]^+$.

3-((Methylthio)ethynyl)-1-tosyl-1H-indole (1n)

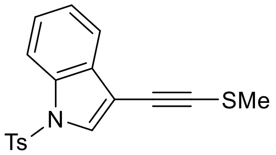

Following **GP1**, using 3-ethynyl-1-tosyl-1H-indole **TA5** (0.47 g, 1.6 mmol) in dry THF (16 mL), *n*-BuLi (2.5 M in hexane, 0.76 mL, 1.9 mmol, 1.2 equiv.) and then *S*-methyl 4-methylbenzenesulfonothioate (0.44 g, 2.2 mmol, 1.4 equiv.) was added in a single portion. After 2 h the reaction was quenched with a saturated NH_4Cl solution (10 mL) and the product was extracted with Et_2O (3 × 10 mL). The combined organic layers were dried over Na_2SO_4, filtered and concentrated under reduced pressure to give a residue which was purified by silica gel flash column chromatography (5% EtOAc in hexane) and then recrystallised from hexane-EtOAc to afford alkynyl thioether **1n** as pale yellow crystals (0.31 g, 56%); mp 129-131 °C; IR (neat): *ν* = 2973, 2901, 2216, 1519, 1370, 1348, 1125, 1096 cm^{-1}; ^1H NMR (300 MHz, $CDCl_3$): δ = 7.96 (d, *J* = 8.2 Hz, 1H), 7.77 (d, *J* = 8.4 Hz, 2H), 7.73 (s, 1H), 7.63 (d, *J* = 7.2 Hz, 1H), 7.38-7.21 (m, 4H), 2.49 (s, 3H), 2.34 (s, 3H); ^{13}C NMR (101 MHz, $CDCl_3$): δ = 145.5, 135.0, 134.3, 131.1, 130.1 (2C), 129.5, 127.1 (2C), 125.6, 123.9, 120.7, 113.7, 105.6, 85.3, 82.8, 21.7, 19.8; HRMS (ES): *m/z* calculated for $C_{18}H_{16}NO_2S_2$: 342.0622, found 342.0618 $[M+H]^+$.

Synthesis of Internal Alkynes

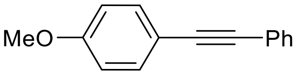

1-Methoxy-4-(phenylethynyl)benzene (4a):

The internal alkyne **4a** was synthesised using literature methods with spectroscopic data matching that reported.[13]

1-Methoxy-4-(prop-1-yn-1-yl)benzene (4b):

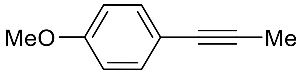

Following a modified **GP1**, using 1-ethynyl-4-methoxybenzene **TA2** (132 mg, 1.0 mmol) in dry THF (5 mL) and LiHMDS (2.2 mL, 1 M in THF, 1.1 mmol) before methyl iodide (0.11 mL, 2.0 mmol) was added dropwise. Purification by flash column chromatography (10% EtOAc in hexane) afforded the internal alkyne **4b** as a pale-yellow liquid (135 mg, 92%); IR (neat): *ν* = 2915, 2847, 2159, 1510, 1441, 1291, 1247, 1173, 1034, 833, 664 cm^{-1}; ^1H NMR (300 MHz, $CDCl_3$): δ = 7.32 (d, *J* = 8.8 Hz, 2H), 6.81 (d, *J* = 8.8 Hz, 2H), 3.79 (s, 3H), 2.03 (s, 3H); ^{13}C NMR (101 MHz, $CDCl_3$): δ = 159.1, 132.9 (2C), 116.3, 114.0 (2C), 84.2, 79.6, 55.4, 4.4; HRMS (ES): *m/z* calculated for $C_{10}H_{10}O$: 146.0732, found 146.0729 $[M]^+$.

Synthesis of Pyridinium-*N*-Aminides

Substituted pyridine *N*-aminides 2a(II-VII):[13,14]

The aminides were synthesised using literature methods to give spectroscopic data matching that reported: **II**[13,14]: 33%, **III**[13,14]: 46%, **IV**[13,14]: 47%, **V**[13,14]: 52%, **VI**[13,14]: 49%, **VII**[13]: 62%.

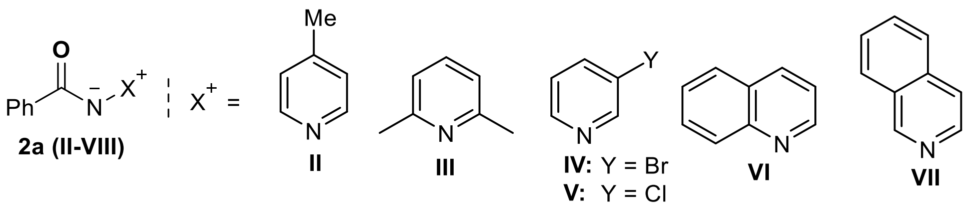

N-Acyl pyridinium-*N*-aminides:[13,14,15,16]

Synthesised according to literature procedures in the following yields: **2a**[13,15]: 76%, **2b**[13,15]: 74%, **2e**[13,15]: 93%, **2f**[16]: 84%, **2h**[16]: 61%, **2i**[16]: 78%, **2j**[16]: 84%, **2k**[16]: 71% , **2m**[16]: 86% with spectroscopic data matching that reported.

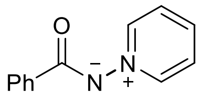

2a

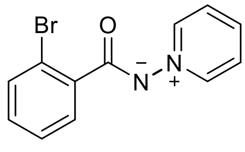

2b

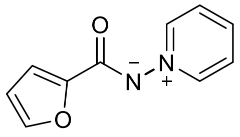

2e

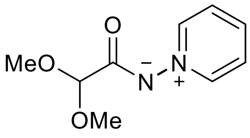

2f

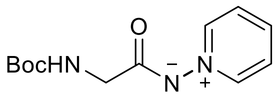

2h

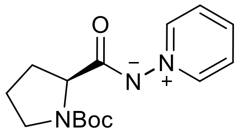

2i

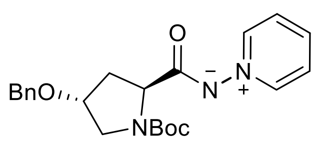

2j

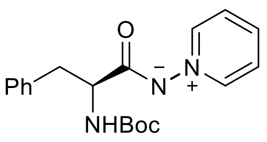

2k

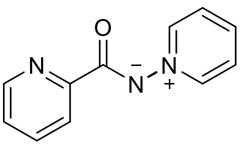

2m

General Procedure for the formation of *N*-acyl pyridinium *N*-aminides (GP2)

Following the literature procedure,[16] a mixture of *N*-aminopyridinium iodide (1.0 equiv.), K_2CO_3 (2.4 equiv.) and the corresponding methyl ester or acid chloride (1.2-2.0 equiv.) were stirred in MeOH (~0.1 M w.r.t. *N*-aminopyridinium iodide). The reaction mixture was allowed to stir at r.t. for 3 days. MeOH was removed under reduced pressure and the resulting residue was taken up in EtOAc or CH_2Cl_2-MeOH (9:1). The undissolved salts were removed by filtration through a pad of alumina and the filtrate was evaporated under reduced pressure. The resulting residue was purified by silica gel flash column chromatography.

(2-Naphthoyl)(pyridin-1-ium-1-yl)amide (2c):

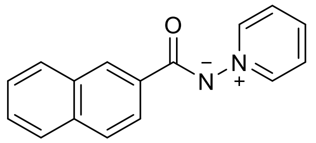

Following **GP2**, using *N*-aminopyridinium iodide (444 mg, 2.0 mmol), K_2CO_3 (662 mg, 4.8 mmol), MeOH (20 mL), and 2-naphthoyl chloride (762 mg, 4.0 mmol). Purification by flash column chromatography (5% to 10% MeOH/CH_2Cl_2) afforded aminide **2c** as a colourless solid (255 mg, 51%); mp. 116-118 °; IR (neat): *v* = 3056, 3027, 1619, 1573, 1556, 1472, 1324, 1253, 1163, 904, 771, 747, 686 cm^{-1}; ^1H NMR (300 MHz, $CDCl_3$): δ = 8.84 (d, *J* = 5.9 Hz, 2H), 8.69 (s, 1H), 8.26 (d, *J* = 8.4 Hz, 1H), 8.03-7.78 (m, 4H), 7.63 (t, *J* = 7.1 Hz, 2H), 7.56-7.40 (m, 2H); ^{13}C NMR (101 MHz, $CDCl_3$): δ = 170.8, 143.4 (2C), 136.9, 134.7, 134.6, 133.1, 129.1, 128.2, 127.7, 127.5, 126.7, 126.1 (2C), 125.9, 125.4; HRMS (ES): *m/z* calculated for $C_{16}H_{13}N_2O$: 249.1028, found 249.1030 $[M+H]^+$.

(1-Naphthoyl)(pyridin-1-ium-1-yl)amide (2d):

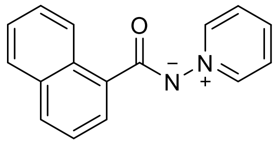

Following **GP2**, *N*-aminopyridinium iodide (444 mg, 2.0 mmol), K_2CO_3 (662 mg, 4.8 mmol), MeOH (20 mL) and 1-naphthol chloride (0.46 mL, 4.0 mmol). Purified by flash column chromatography (5% to 10% MeOH/CH_2Cl_2) to afforded aminide **2d** as a colourless solid (400 mg, 81%); mp. 142-144 °C; IR (neat): *v* = 3057, 1709, 1597, 1579, 1544, 1460, 1348, 1320. 1199, 953, 753, 740, 673 cm^{-1}; ^1H NMR (300 MHz, $CDCl_3$): δ = 8.87 (d, *J* = 5.5 Hz, 2H), 8.71 (d, *J* = 7.6 Hz, 1H), 7.95 (d, *J* = 6.7 Hz, 1H), 7.92-7.70 (m, 3H), 7.70-7.34 (m, 5H); ^{13}C NMR (101 MHz, $CDCl_3$): δ = 173.5, 143.3 (2C), 137.2, 136.4, 133.9, 131.2, 129.5, 128.2, 126.5, 126.4, 126.2, 126.1 (2C), 125.7, 125.1; HRMS (ES) *m/z*: calculated for $C_{16}H_{13}N_2O$: 249.1028, found 249.1036 $[M+H]^+$.

(2-Hydroxy-2-methylpropanoyl)(pyridin-1-ium-1-yl)amide (2g):

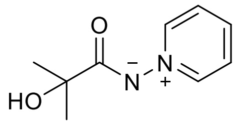

Following **GP2**, *N*-aminopyridinium iodide (444 mg, 2.0 mmol), K_2CO_3 (0.67 g, 4.8 mmol), MeOH (20 mL) and methyl 2-hydroxyisobutyrate (0.28 mL, 2.4 mmol). Purified by flash column chromatography (10% MeOH/CH_2Cl_2) to afforded aminide **2g** as an off-white solid (120 mg, 33%); mp. 110-112 °C; IR (neat): *v* = 3404, 3072, 2940, 1619, 1566, 1472, 1318, 1174 cm^{-1}; ^1H NMR (300 MHz, $CDCl_3$): δ = 8.69 (d, *J* = 5.6 Hz, 2H), 7.94 (tt, *J* = 7.7, 1.2 Hz, 1H), 7.67 (t, *J* = 7.4 Hz, 2H), 4.30 (s, 1H), 1.50 (s, 6H); ^{13}C NMR (101 MHz, $CDCl_3$): δ = 180.4, 143.2 (2C), 137.4, 126.2 (2C), 72.1, 28.7 (2C); HRMS (ES): *m/z* calculated for $C_9H_{13}N_2O_2$: 181.0977, found 181.0974 $[M+H]^+$.

(2-(Methoxycarbonyl)benzoyl)(pyridin-1-ium-1-yl)amide (2l):

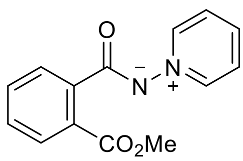

Monomethyl hydrogen phthalate (0.54 g, 3.0 mmol) and Et_3N (0.84 mL, 6.0 mmol, 2.0 equiv.) were added to CH_2Cl_2 (30 mL) and the reaction was cooled to 0 °C for 5 minutes. Ethyl chloroformate (0.32 mL, 3.3 mmol, 1.1 equiv.) was added and the reaction was stirred at 0 °C for 1 h. After this time, 1-aminopyridinium iodide (0.73 g, 3.3 mmol, 1.1 equiv.) and MeOH (6 mL) were added and the cooling bath was removed. After 18 h the reaction was poured into 1M NaOH (100 mL) and the layers were separated. The aqueous layer was washed twice with CH_2Cl_2 (2 × 50 mL). The combined organic layers were dried with Na_2SO_4 which was then filtered off. After removal of solvent under reduced pressure, the crude material was purified by flash column chromatography (10% MeOH in CH_2Cl_2) to afford aminide **2l** as a colourless solid (0.60 g, 78%); mp. 136-138 °C; IR (neat): ν = 3109, 1725, 1688, 1599, 1414, 1327, 1300, 1115 cm^{-1}; ^1H NMR (300 MHz, $CDCl_3$): δ = 8.87-8.84 (m, 2H), 7.92 (tt, *J* = 7.7, 1.3 Hz, 1H), 7.76 (td, *J* = 7.8, 1.2 Hz, 2H), 7.68 (t, *J* = 7.0 Hz, 2H), 7.51 (td, *J* = 7.5, 1.2 Hz, 1H), 7.40 (t, *J* = 7.5, 1.2 Hz, 1H), 3.88 (s, 3H); ^{13}C NMR (101 MHz, $CDCl_3$): δ = 172.9, 168.7, 143.2 (2C), 140.1, 137.2, 131.4,

130.5, 129.2, 128.5, 128.5, 126.3 (2C), 52.3; HRMS (ES): *m/z* calculated for $C_{14}H_{13}N_2O_3$: 257.0926, found 257.0925 $[M+H]^+$.

(3-Methoxy-3-oxopropanoyl)(pyridin-1-ium-1-yl)amide (2n):

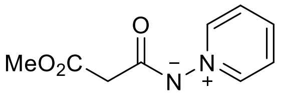

Following **GP2**, *N*-aminopyridinium iodide (1.78 g, 8.0 mmol), K_2CO_3 (2.65 g, 19.2 mmol), MeOH (60 mL) and dimethyl malonate (1.10 mL, 9.6 mmol). Purified by flash column chromatography (15% MeOH in CH_2Cl_2) to afford aminide **2n** as a colourless solid (0.63 g, 41%); mp. 120-122 °C; IR (neat): ν = 3124, 3074, 2941, 1741, 1584, 1471, 1338, 1259, 1127, 1016 cm^{-1}; ^1H NMR (300 MHz, $CDCl_3$): δ = 8.69-8.66 (m, 2H), 7.92 (tt, *J* = 7.7 Hz, 1.3 Hz, 1H), 7.65 (t, *J* = 7.0 Hz, 2H), 3.73 (s, 3H), 3.38 (s, 2H); ^{13}C NMR (101 MHz, $CDCl_3$): δ = 170.5, 170.3, 143.2 (2C), 137.6, 126.2 (2C), 52.2, 43.4; HRMS (ES): *m/z* calculated for $C_9H_{10}N_2O_3Na$: 217.0589, found 217.0580 $[M+Na]^+$.

5,5-Dimethyl-3-phenyl-1,4,2-dioxazole[17] (**7** 70%) was synthesised using a literature method to give spectroscopic data matching that reported.[17]

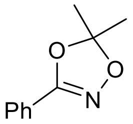

Survey of Reaction Conditions [a]

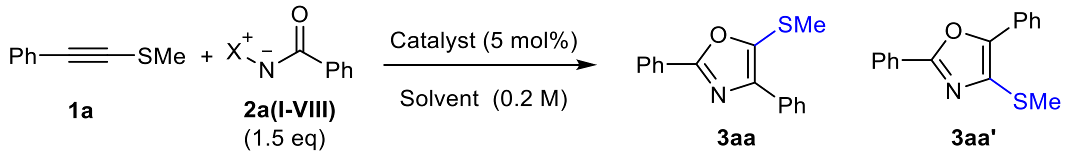

Entry	Cat	Solvent	2a	T (°C)	Time	Yield[b]	(3aa:3aa')[c]
1	$PicAuCl_2$	toluene	**I**	90 °C	20 h	22%	8.4:1
2	$PicAuCl_2$	1,4-dioxane	**I**	90 °C	24 h	25%	8.5:1
3	$PicAuCl_2$	*m*-xylene	**I**	120 °C	48 h	25%	8.0:1
4	$PicAuCl_2$	Chloro benzene	**I**	120 °C	24 h	32%	8.0:1
5	$PicAuCl_2$	1,2-DCB	**I**	90 °C	48 h	40%	10.0:1
6	$PicAuCl_2$	1,2-DCB	**I**	110 °C	30 h	60%	8.3:1
**7**	**$PicAuCl_2$**	**1,2-DCB**	**I**	**125 °C**	**24 h**	**72%**	**8.4:1**
8	DTBPAu.NCMe.SbF_6	1,2-DCB	**I**	125 °C	40 h	28%	4.5:1
9	$[Ir(cod)Cl]_2$	1,2-DCB	**I**	125 °C	24 h	NR	--
10[d]	--	1,2-DCB	**I**	125 °C	24 h	NR	--
11[e]	$PicAuCl_2$	1,2-DCB	**I**	125 °C	20 h	71%	8.0:1
12[f]	$PicAuCl_2$	1,2-DCB	**I**	125 °C	24 h	45%	7.6:1
13	$PicAuCl_2$	1,2-DCB	**II**	125 °C	24 h	30%	8.0:1
14	$PicAuCl_2$	1,2-DCB	**III**	125 °C	24 h	traces	--
**15**	**$PicAuCl_2$**	**1,2-DCB**	**IV**	**125 °C**	**24 h**	**70%**	**8.0:1**
16	$PicAuCl_2$	1,2-DCB	**V**	125 °C	24 h	51%	7.5:1
17	$PicAuCl_2$	1,2-DCB	**VI**	125 °C	24 h	32%	7.5:1
18	$PicAuCl_2$	1,2-DCB	**VII**	125 °C	24 h	15%	9.2:1

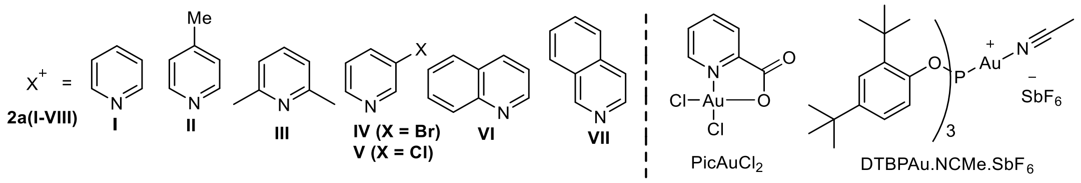

[a] All reactions were carried out on 0.1 mmol scale; [b] For entries 1 and 2 the yield was determined by ^1H NMR of the reaction mixture against a known quantity of internal standard. All other entries are isolated yields after flash chromatography; [c] Ratio based on ^1H NMR analysis after purification; [d] No catalyst; [e] Using 10 mol% of $PicAuCl_2$; [f] Using 1.2 equiv. of aminide. DCB: 1,2-dichlorobenzene.

Synthesis of 5-Thio-oxazoles

General Procedure for the formation of 5-thio-oxazoles (GP3)

A heat gun-dried reaction tube was charged with the corresponding pyridinium-*N*-aminide (1.5-3.0 equiv.), dichloro(2-pyridinecarboxylato)gold (5 or 10 mol%) and solid alkynyl thioether (1.0 equiv.) under an argon atmosphere before 1,2-dichlorobenzene (0.2 M with respect to the alkynyl thioether) was added. In cases when the alkynyl thioether was an oil/liquid it was prepared as a solution in 1,2-dichlorobenzene (0.2 M) and then added to the aminide and catalyst. The mixture was stirred at 125 °C until the reaction was either complete or appeared to be proceeding no further (determined by TLC). Heating was removed and the reaction mixture allowed to cool down to room temperature and then the reaction mixture was subjected to silica gel flash column chromatography.

2,4-Diphenyl-5-(methylthio)oxazole (3aa):

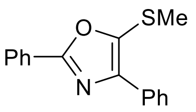

Following **GP3**, using alkynyl thioether **1a** (29.6 mg, 0.2 mmol), aminide **2a** (59.4 mg, 0.3 mmol) and gold catalyst (3.9 mg, 0.01 mmol) for 24 h. Purification by flash column chromatography (3% EtOAc in hexane) afforded oxazole **3aa** as a pale yellow solid (38.4 mg, 72%); mp. 42-44 °C; IR (neat): ν = 2987, 2923, 1552, 1487, 1446, 1340, 1068, 977, 775, 713, 687 cm^{-1}; *Mixture of regioisomers (8.4:1) observed by* ^{1}H NMR (300 MHz, $CDCl_3$): δ = 8.22-8.05 and 8.03-7.93 (m, 4H), 7.54-7.43 (m, 5H), 7.37 (tt, *J* = 7.3, 1.3 Hz, 1H), 2.63 and 2.52 (s, 3H); ^{13}C NMR (101 MHz, $CDCl_3$): δ = 162.5, 142.6, 140.2, 131.4, 130.8, 128.9 (2C), 128.6 (2C), 128.3, 127.4, 127.3 (2C), 126.7 (2C), 18.7; HRMS (ES): *m/z* calculated for $C_{16}H_{14}NOS$: 268.0796, found 268.0790 $[M+H]^+$. *Single crystals were grown from a mixture of **3aa** and **3aa'** in hexane. ^{1}H NMR analysis of the bulk crystals showed enhanced regioisomeric ratio of 20:1.*

2,4-Diphenyl-5-(ethylthio)oxazole (3ba):

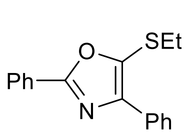

Following **GP3**, using alkynyl thioether **1b** (32.4 mg, 0.2 mmol), aminide **2a** (59.4 mg, 0.3 mmol) and gold catalyst (3.9 mg, 0.01 mmol) for 24 h. Purification by flash column chromatography using 3% EtOAc in hexane afforded oxazole **3ba** as a pale-yellow liquid (39.2 mg, 70%); IR (neat): ν = 3059, 2965, 2926, 1606, 1552, 1488, 1446, 1340, 1258, 1069, 977, 777, 716, 690 cm^{-1}; *Mixture of regioisomers (6.5:1) observed by* ^{1}H NMR (300 MHz, $CDCl_3$): δ = 8.34-8.00 and 8.09-8.01 (m, 4H), 7.60-7.43 (m, 5H), 7.37 (tt, *J* = 7.3, 1.3 Hz, 1H), 3.14 and 2.95 (q, *J* = 7.4 Hz, 2H), 1.38 and 1.34 (t, *J* = 7.4 Hz, 3H); ^{13}C NMR (101 MHz, $CDCl_3$): δ = 162.6, 143.9, 139.1, 131.4, 130.8, 128.9 (2C), 128.5 (2C), 128.3, 127.3 (2C), 126.7 (2C), 125.6, 30.5, 15.3; HRMS (ES): *m/z* calculated for $C_{17}H_{16}NOS$: 282.0953, found 282.0960 $[M+H]^+$.

2,4-Diphenyl-5-(isopropylthio)oxazole (3ca):

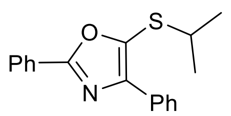

Following **GP3**, using alkynyl thioether **1c** (35.2 mg, 0.2 mmol), aminide **2a** (59.4 mg, 0.3 mmol) and gold catalyst (3.9 mg, 0.01 mmol) for 24 h. Purification by flash column chromatography using 3% EtOAc in hexane afforded oxazole **3ca** as a pale-yellow liquid (38.3 mg, 65%); IR (neat): ν = 2971, 2901, 1550, 1487, 1446, 1341, 1230, 1150, 1067, 974, 780, 711, 682 cm^{-1}; *Mixture of regioisomers (4.5:1) observed by* ^{1}H NMR (300 MHz, $CDCl_3$): δ = 8.33-8.04 (m, 4H), 7.56-7.42 (m, 5H), 7.36 (tt, *J* = 7.3, 1.3 Hz, 1H), 3.71 and 3.42 (sept, *J* = 6.7 Hz, 1H), 1.38 and 1.34 (d, *J* = 6.7 Hz, 6H); ^{13}C NMR (101 MHz, $CDCl_3$): δ = 162.8, 144.8, 139.0, 131.5, 130.8, 128.9 (2C), 128.5 (2C), 128.4, 127.4 (2C), 126.7 (2C), 125.8, 41.2, 23.6 (2C); HRMS (ES): *m/z* calculated for $C_{18}H_{18}NOS$: 298.1104, found 298.1109 $[M+H]^+$.

2,4-Diphenyl-5-(phenylthio)oxazole (3da):

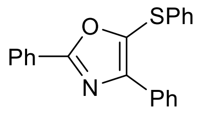

Following **GP3**, using alkynyl thioether **1d** (42.0 mg, 0.2 mmol), aminide **2a** (59.4 mg, 0.3 mmol) and gold catalyst (3.9 mg, 0.01 mmol) for 24 h. Purification by flash column chromatography using 3% EtOAc in hexane afforded oxazole **3da** as a colourless solid (40.2 mg, 61%); mp. 60-62 °C; IR (neat): ν = 2974, 2901, 1606, 1480, 1444, 1148, 1066, 1025, 978, 743, 714, 683 cm^{-1}; *mixture of regioisomers (Due to overlapping resonances in the aromatic region the regioisomeric ratio of 4.8:1 is based on ^{1}H NMR analysis of the crude reaction mixture when converting **3da** into 2,4-diphenyl-5-methyl oxazole **5a**),* ^{1}H NMR (300 MHz, $CDCl_3$): δ = 8.28-8.10 (m, 4H), 7.56-7.40 (m, 6H), 7.33-7.16 (m, 5H); ^{13}C NMR (101 MHz, $CDCl_3$): δ = 163.7, 146.5, 136.0, 134.9, 131.1, 130.9, 129.5 (2C), 129.2, 128.9 (2C), 128.8, 128.6 (2C), 127.7, 127.4 (2C), 126.9 (3C), 126.3; HRMS (ES): *m/z* calculated for $C_{21}H_{16}NOS$: 330.0953, found 330.0957 $[M+H]^+$.

5-(Benzylthio)-2,4-diphenyloxazole (3ea):

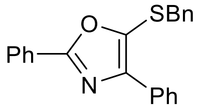

Following **GP3**, using alkynyl thioether **1e** (44.8 mg, 0.2 mmol), aminide **2a** (59.4 mg, 0.3 mmol) and gold catalyst (3.9 mg, 0.01 mmol) for 30 h. Purification by flash column chromatography using 3% EtOAc in hexane afforded oxazole **3ea** as a colourless solid (35.0 mg, 51%); mp. 32-34 °C; IR (neat): ν = 3031, 2922, 1603, 1547, 1485, 1444, 1338, 1239, 1068, 978, 920, 777, 764, 716, 687 cm^{-1}; *mixture of regioisomers (6.3:1) observed by* ^{1}H NMR (300 MHz, $CDCl_3$): δ = 8.24-8.04 (m, 2H), 8.03-7.82 (m, 2H), 7.55-7.46 (m, 3H), 7.44-7.31 (m, 3H), 7.30-7.08 (m, 5H), 4.34 and 4.10 (s, 2H); ^{13}C NMR (101 MHz, $CDCl_3$): δ = 162.9, 144.8, 138.4, 136.9, 131.2, 130.8, 129.1 (2C), 128.9 (2C), 128.7 (2C), 128.4 (2C), 127.7, 127.3 (2C), 126.7 (2C), 126.5, 125.7, 40.8; HRMS (ES): *m/z* calculated for $C_{22}H_{18}NOS$: 344.1109, found 344.1100 $[M+H]^+$.

5-(Ethylthio)-4-(4-methoxyphenyl)-2-phenyloxazole (3ga):

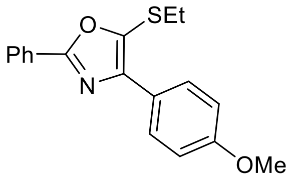

Following **GP3**, using alkynyl thioether **1g** (38.4 mg, 0.2 mmol), aminide **1a** (59.4 mg, 0.3 mmol) and gold catalyst (7.8 mg, 0.02 mmol) for 24 h Purification by flash column chromatography using toluene afforded oxazole **3ga** as a colourless solid (48.5 mg, 78%); mp: 50-52 °C; IR (neat): *ν* = 2987, 2945, 1616, 1546, 1482, 1238, 1190, 1037, 972, 830, 812, 762, 743, 725 cm^{-1}; *mixture of regioisomers (21.0:1) observed by* ^{1}H NMR (400 MHz, $CDCl_3$): δ = 8.20-8.08 (m, 4H), 7.52-7.45 (m, 3H), 6.99 (d, *J* = 9.0 Hz, 2H), 3.86 (s, 3H), 2.91 (q, *J* = 7.4 Hz, 2H), 1.32 (t, *J* = 7.4 Hz, 3H); ^{13}C NMR (101 MHz, $CDCl_3$): δ = 162.5, 159.7, 144.0, 137.8, 130.7, 128.8 (2C), 128.7 (2C), 127.4, 126.6 (2C), 124.0, 113.9 (2C), 55.4, 30.5, 15.3; HRMS (ES): *m/z* calculated for $C_{18}H_{18}NO_2S$: 312.1058, found 312.1055 $[M+H]^+$.

*The reaction with 5 mol% of gold catalyst was sluggish. After 48 h, purification afforded oxazole **3ga** as a colourless solid (39.8 mg, 64%) with the same level of regioselectivity.*

*Carefully washing this compound with hexane afforded **3ga** as a single regioisomer, as determined by ^{1}H NMR analysis. This pure regiosomer was used to grow to single crystals (2% EtOAc in hexane) which were employed in single crystal X-ray diffraction analysis.*

4-(4-Methoxyphenyl)-2-phenyl-5-(phenylthio)oxazole (3ha):

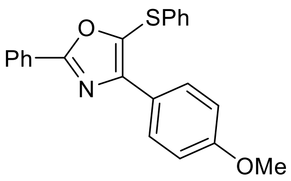

Following **GP3**, using alkynyl thioether **1h** (48.0 mg, 0.2 mmol), aminide **2a** (59.4 mg, 0.3 mmol) and gold catalyst (7.8 mg, 0.02 mmol) for 24 h. Purification by flash column chromatography using toluene afforded oxazole **3ha** as a pale-yellow solid (48.0 mg, 67%); mp: 70-72 °C; IR (neat): *ν* = 3026, 2924, 1607, 1578, 1549, 1499, 1444, 1302, 1250, 1176, 1146, 965, 840, 736, 689 cm^{-1}; *mixture of regioisomers (15.0:1) observed by* ^{1}H NMR (300 MHz, $CDCl_3$): δ = 8.17 and 8.07 (d, *J* = 8.9 Hz, 4H), 7.53-7.44 (m, 3H), 7.38-7.25 (m, 4H), 7.24-7.18 (m, 1H), 6.99 (d, *J* = 8.9 Hz, 2H), 3.85 (s, 3H); ^{13}C NMR (101 MHz, $CDCl_3$): δ = 163.6, 160.1, 146.6, 135.2, 134.5, 131.0, 129.5, 128.9 (2C), 128.8 (2C), 127.3 (2C), 127.2, 126.9 (2C), 126.8, 126.7, 123.4, 114.0 (2C), 55.4; HRMS (ES): *m/z* calculated for $C_{22}H_{18}NO_2S$: 360.1058, found 360.1062 $[M+H]^+$.

4-(4-Methoxyphenyl)-5-(methylthio)-2-phenyloxazole (3ia):

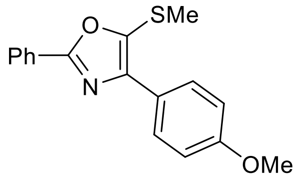

Following **GP3**, using alkynyl thioether **1i** (35.6 mg, 0.2 mmol), aminide **2a** (59.4 mg, 0.3 mmol) and gold catalyst (7.8 mg, 0.02 mmol) for 24 h Purification by flash column chromatography using toluene afforded oxazole **3ia** as a colourless solid (43.5 mg, 73%); mp: 75-77 °C; IR (neat): *v* = 2954, 2925, 2236, 1609, 1498, 1444, 1246, 1174, 1034, 977, 830, 779, 734, 710, 690 cm^{-1}; *mixture of regioisomers (26.0:1) observed by* ^{1}H NMR (300 MHz, $CDCl_3$): δ = 8.18-8.03 (m, 4H), 7.53-7.42 (m, 3H), 7.00 (d, *J* = 8.9 Hz, 2H), 3.86 (s, 3H), 2.49 (s, 3H); ^{13}C NMR (101 MHz, $CDCl_3$): δ = 162.3, 159.7, 142.7, 138.9, 130.7, 128.9 (2C), 128.6 (2C), 127.4, 126.6 (2C), 124.0, 114.0 (2C), 55.4, 18.9; HRMS (ES): *m/z* calculated for $C_{17}H_{16}NO_2S$: 298.0902, found 298.0900 $[M+H]^+$.

*The reaction with 5 mol% of gold catalyst was sluggish. After 48 h, purification afforded oxazole **3ia** as a colourless solid (35.0 mg, 59%) with the same level of regioselectivity*.

2-(2-Bromophenyl)-5-(ethylthio)-4-phenyloxazole (3bb):

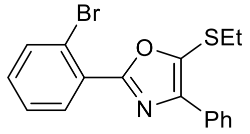

Following **GP3**, using alkynyl thioether **1b** (32.4 mg, 0.2 mmol), aminide **2b** (83.0 mg, 0.3 mmol) and gold catalyst (3.9 mg, 0.01 mmol) for 24 h. Purification by flash column chromatography using 5% EtOAc in hexane afforded oxazole **3bb** as a colourless solid (39.0 mg, 54%); mp. 32-34 °C; IR (neat): ν = 2961, 2919, 1686, 1602, 1567, 1446, 1258, 1077, 1027, 978, 769, 730, 693 cm^{-1}; *mixture of regioisomers (5.0:1) observed by* ^{1}H NMR (300 MHz, $CDCl_3$): δ = 8.17 (d, *J* = 7.2 Hz, 2H), 8.08 and 8.03 (dd, *J* = 6.3, 1.6 Hz, 1H), 7.73 (dd, *J* = 7.0, 1.0 Hz, 1H), 7.52-7.28 (m, 5H), 3.15 and 2.99 (q, *J* = 7.4 Hz, 2H), 1.39 and 1.35 (t, *J* = 7.4 Hz, 3H); ^{13}C NMR (101 MHz, $CDCl_3$): δ = 161.1, 142.9, 140.0, 134.6, 131.5 (2C), 131.2, 128.5 (2C), 128.3, 127.5, 127.3 (2C), 125.7, 121.2, 30.3, 15.4; HRMS (ES): *m/z* calculated for $C_{17}H_{15}NOS^{79}Br$: 360.0058, found 360.0051 $[M+H]^+$.

2-(2-Bromophenyl)-5-(ethylthio)-4-(4-methoxyphenyl)oxazole (3gb):

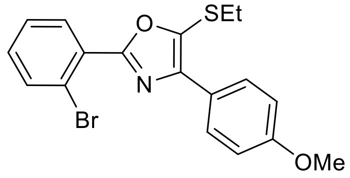

Following **GP3**, using alkynyl thioether **1g** (38.4 mg, 0.2 mmol), aminide **2b** (83.0 mg, 0.3 mmol) and gold catalyst (7.8 mg, 0.02 mmol) for 24 h. Purification by flash column chromatography using toluene afforded oxazole **3gb** as a pale-yellow liquid (55.3 mg, 71%); IR (neat): *v* = 2967, 2925, 1611, 1566, 1496, 1248, 1176, 1034, 978, 834, 818, 766, 739, 726 cm^{-1}; *mixture of regioisomers (10.0:1) observed by* ^{1}H NMR (300 MHz, $CDCl_3$): δ = 8.13 (d, *J* = 8.9 Hz, 2H), 8.02 (dd, *J* = 6.1, 1.7 Hz, 1H), 7.72 (dd, *J* = 7.0, 1.0 Hz, 1H), 7.42 (app. td, *J* = 7.5, 1.1 Hz, 1H), 7.30 (app. td, *J* = 7.7, 1.8 Hz, 1H), 6.99 (d, *J* = 8.9 Hz, 2H), 3.86 (s, 3H), 2.96 (q, *J* = 7.4 Hz, 2H), 1.34 (t, *J* = 7.4 Hz, 3H); ^{13}C NMR (101 MHz, $CDCl_3$): δ = 161.0, 159.7, 143.1, 138.6, 134.6, 131.5 (2C), 128.6 (2C), 128.4, 127.5, 123.9, 121.2, 113.9 (2C), 55.4, 30.4, 15.3; HRMS (ES): *m/z* calculated for $C_{18}H_{16}NO_2S^{79}BrNa$: 411.9983, found 411.9984 $[M+Na]^+$.

5-(Methylthio)-2-(naphthalen-2-yl)-4-phenyloxazole (3ac):

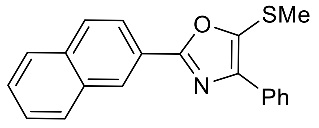

Following **GP3**, using alkynyl thioether **1a** (29.6 mg, 0.2 mmol), aminide **2c** (74.4 mg, 0.3 mmol) and gold catalyst (3.9 mg, 0.01 mmol) for 24 h. Purification by flash column chromatography using 5% EtOAc in hexane afforded oxazole **3ac** as a pale-yellow solid (39.5 mg, 62%); mp: 86-88 °C; IR (neat): ν = 3054, 2918, 1606, 1581, 1541, 1486, 1444, 1196, 1070, 982, 903, 756, 731, 693 cm^{-1}; *mixture of regioisomers (8.0:1) observed by* ^{1}H NMR (300 MHz, $CDCl_3$): δ = 8.63 and 8.61 (s, 1H), 8.26-

8.15 (m, 3H), 8.00-7.86 (m, 3H), 7.59-7.47 (m, 4H), 7.39 (tt, *J* = 7.4, 1.3 Hz, 1H), 2.68 and 2.57 (s, 3H); ^{13}C NMR (101 MHz, $CDCl_3$): δ = 162.5, 142.8, 140.4, 134.4, 133.1, 131.3, 128.9, 128.7, 128.6 (2C), 128.4, 128.0, 127.5, 127.3 (2C), 126.9, 126.6, 124.6, 123.5, 18.7; HRMS (ES): *m/z* calculated for $C_{20}H_{16}NOS$: 318.0953, found 318.0960 $[M+H]^+$.

5-(Ethylthio)-4-(4-methoxyphenyl)-2-(naphthalen-2-yl)oxazole (3gc):

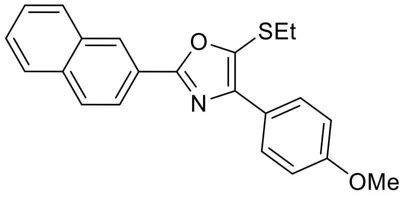

Following **GP3**, using alkynyl thioether **1g** (38.4 mg, 0.2 mmol), aminide **2c** (74.4 mg, 0.3 mmol) and gold catalyst (3.9 mg, 0.01 mmol) for 36 h. Purification by flash column chromatography using toluene afforded oxazole **3gc** as a pale-yellow solid (63.6 mg, 88%); mp: 71-73 °C; IR (neat): *v* = 2957, 2928, 1606, 1547, 1495, 1247, 1176, 1032, 977, 837, 818, 751, 661 cm^{-1}; *mixture of regioisomers (20.0:1) observed by* ^{1}H NMR (300 MHz, $CDCl_3$): δ = 8.62 (s, 1H), 8.28-8.15 (m, 3H), 8.00-7.92 (m, 2H), 7.92-7.83 (m, 1H), 7.59-7.51 (m, 2H), 7.02 (d, *J* = 8.9 Hz, 2H), 3.87 (s, 3H), 2.96 (q, *J* = 7.4 Hz, 2H), 1.36 (t, *J* = 7.4 Hz, 3H); ^{13}C NMR (101 MHz, $CDCl_3$): δ = 162.6, 159.7, 144.2, 138.0, 134.3, 133.1, 128.9, 128.7 (3C), 128.0, 127.4, 126.9, 126.6, 124.6, 124.0, 123.5, 113.9 (2C), 55.4, 30.6, 15.3; HRMS (ES): *m/z* calculated for $C_{22}H_{20}NO_2S$: 362.1215, found 362.1227 $[M+H]^+$.

5-(Ethylthio)-2-(naphthalen-1-yl)-4-phenyloxazole (3bd):

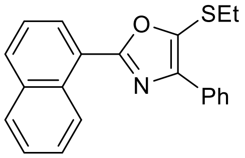

Following **GP3**, using alkynyl thioether **1b** (32.4 mg, 0.2 mmol), aminide **2d** (74.4 mg, 0.3 mmol) and gold catalyst (3.9 mg, 0.01 mmol) for 22 h. Purification by flash column chromatography using 5% EtOAc in hexane afforded oxazole **3bd** as a pale-yellow liquid (46.3 mg, 70%); IR (neat): ν = 2988, 2924, 1607, 1554, 1487, 1448, 1316, 1170, 925, 765, 686 cm^{-1}; *mixture of regioisomers (4.7:1) observed by* ^{1}H NMR (300 MHz, $CDCl_3$): δ = 9.48 and 9.39 (d, *J* = 8.5 Hz, 1H), 8.38-8.24 and 8.14-8.03 (m, 3H), 8.00 (d, *J* = 8.2 Hz, 1H), 7.93 (d, *J* = 8.1 Hz, 1H), 7.75-7.66 (m, 1H), 7.63-7.47 (m, 4H), 7.40 (tt, *J* = 7.4, 1.2 Hz, 1H), 3.24 and 3.01 (q, *J* = 7.4 Hz, 2H), 1.45 and 1.38 (t, *J* = 7.4 Hz, 3H); ^{13}C NMR (101 MHz, $CDCl_3$): δ = 162.5, 143.5, 139.0, 134.1, 131.7, 131.5, 130.3, 128.7, 128.6 (2C), 128.3, 128.1, 127.8, 127.3 (2C), 126.4 (2C), 125.0, 123.6, 30.4, 15.4; HRMS (ES): *m/z* calculated for $C_{21}H_{18}NOS$: 332.1103, found 332.1109 $[M+H]^+$.

5-(Ethylthio)-4-(4-methoxyphenyl)-2-(naphthalen-1-yl)oxazole (3gd):

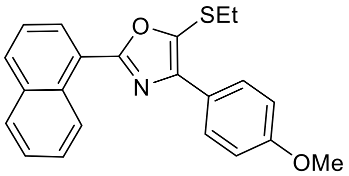

Following **GP3**, using alkynyl thioether **1g** (38.4 mg, 0.2 mmol), aminide **2d** (74.4 mg, 0.3 mmol) and gold catalyst (3.9 mg, 0.01 mmol) for 36 h. Purification by flash column chromatography using toluene afforded oxazole **3gd** as a pale-yellow solid (60.6 mg, 84%); mp: 32-34 °C; IR (neat): *v* = 3148, 2924, 1600, 1589, 1345, 1234, 1151, 1089, 960, 891, 796, 738, 694 cm^{-1}; *mixture of regioisomers (13.0:1) observed by* ^{1}H NMR (300 MHz, $CDCl_3$): δ = 9.47 (d, *J* = 8.7 Hz, 1H), 8.37-8.21 (m, 3H), 7.98 (d, *J* = 8.2 Hz, 1H), 7.92 (d, *J* = 8.2 Hz, 1H), 7.75-7.66 (m, 1H), 7.63-7.54 (m, 2H), 7.05 (d, *J* = 9.0 Hz, 2H), 3.89 (s, 3H), 2.98 (q, *J* = 7.4 Hz, 2H), 1.37 (t, *J* = 7.4 Hz, 3H); ^{13}C NMR (101 MHz, $CDCl_3$): δ = 162.5, 159.7, 143.6, 137.6, 134.1, 131.6, 130.2, 128.7 (3C), 128.1, 127.7, 126.43, 126.41, 125.0, 124.1, 123.7, 113.9 (2C), 55.4, 30.5, 15.4; HRMS (ES): *m/z* calculated for $C_{22}H_{19}NO_2SNa$: 384.1034, found 384.1029 $[M+Na]^+$.

2-(Furan-2-yl)-5-(methylthio)-4-phenyloxazole (3ae):

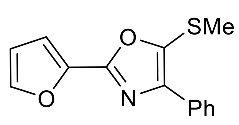

Following **GP3**, using alkynyl thioether **1a** (29.6 mg, 0.2 mmol), aminide **2e** (56.4 mg, 0.3 mmol) and gold catalyst (3.9 mg, 0.01 mmol) for 40 h. Purification by flash column chromatography using 5% EtOAc in hexane afforded oxazole **3ae** as a pale-yellow liquid (32.0 mg, 62%); IR (neat): ν = 2978, 2924, 1632, 1619, 1537, 1445, 1229, 1176, 1067, 1011, 976, 897, 744, 691 cm^{-1}; *mixture of regioisomers (5.7:1) observed by* ^{1}H NMR (300 MHz, $CDCl_3$): δ = 8.17-8.05 and 7.97-7.89 (m, 2H), 7.59 (dd, *J* = 1.6, 0.7 Hz, 1H), 7.49-7.41 (m, 2H), 7.36 (tt, *J* = 7.3, 1.3 Hz, 1H), 7.11 (dd, *J* = 3.5, 0.6 Hz, 1H), 6.57 (dd, *J* = 3.5, 1.8 Hz, 1H), 2.62 and 2.50 (s, 3H); ^{13}C NMR (101 MHz, $CDCl_3$): δ = 155.2, 144.8, 142.8, 142.4, 130.9, 128.8, 128.6 (2C), 127.3 (2C), 125.5, 112.3, 112.1, 18.7; HRMS (ES): *m/z* calculated for $C_{14}H_{12}NO_2S$: 258.0589, found 258.0584 $[M+H]^+$.

2-(Furan-2-yl)-5-(isopropylthio)-4-phenyloxazole (3ce):

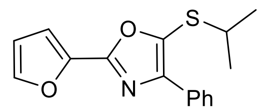

Following **GP3**, using alkynyl thioether **1c** (35.2 mg, 0.2 mmol), aminide **2e** (56.4 mg, 0.3 mmol) and gold catalyst (3.9 mg, 0.01 mmol) for 48 h. Purification by flash column chromatography using 5% EtOAc in hexane afforded oxazole **3ce** as a pale yellow liquid (29.2 mg, 51%); IR (neat): ν = 2971, 2988, 1581, 1550, 1481, 1445, 1382, 1341, 1066, 977, 744, 714, 683 cm^{-1}; *mixture of regioisomers (3.8:1) observed by* ^{1}H NMR (300 MHz, $CDCl_3$): δ = 8.28-8.14 and 8.08-8.02 (m, 2H), 7.60 (dd, *J* = 1.7, 0.7 Hz, 1H), 7.50-7.39 (m, 2H), 7.35 (tt, *J* = 7.3, 1.3 Hz, 1H), 7.11 (dd, *J* = 3.5, 0.7 Hz, 1H), 6.57 (dd, *J* = 3.5, 1.8 Hz, 1H), 3.72 and 3.40 (septet, *J* = 6.7 Hz, 1H), 1.35 and 1.32 (d, *J* = 6.7 Hz, 6H); ^{13}C NMR (101 MHz, $CDCl_3$): δ = 155.5, 144.9, 144.5, 142.9, 131.0, 128.5, 128.4 (2C), 127.5 (2C), 125.9, 112.4, 112.1, 41.2, 23.5 (2C); HRMS (ES): *m/z* calculated for $C_{16}H_{16}NO_2S$: 286.0902, found 286.0908 $[M+H]^+$.

2-(Furan-2-yl)-4-(4-methoxyphenyl)-5-(methylthio)oxazole (3ie):

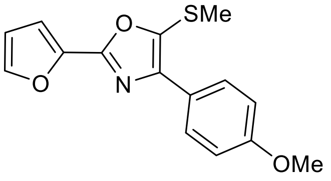

Following **GP3**, using alkynyl thioether **1i** (35.6 mg, 0.2 mmol), aminide **2e** (75.2 mg, 0.4 mmol) and gold catalyst (7.8 mg, 0.02 mmol) for 48 h. Purification by flash column chromatography using 20% EtOAc in hexane afforded oxazole **3ie** as a brown solid (22.9 mg, 40%); IR (neat): *v* = 2923, 1634, 1608, 1525, 1498, 1337, 1301, 1242, 1174, 1030, 967, 897, 831, 757, 736 cm^{-1}; *mixture of regioisomers (7.9:1) observed by* ^{1}H NMR (300 MHz, $CDCl_3$): δ = 8.07 and 7.88 (d, *J* = 9.0 Hz, 2H), 7.58 (dd, *J* = 1.7, 0.7 Hz, 1H), 7.09 and 7.06 (dd, *J* = 3.5, 0.7 Hz, 1H), 6.98 (d, *J* = 9.0 Hz, 2H), 6.55 (dd, *J* = 3.5, 1.8 Hz, 1H), 3.85 (s, 3H), 2.57 and 2.47 (s, 3H); ^{13}C NMR (101 MHz, $CDCl_3$): δ = 159.8, 155.1, 144.8, 142.8, 142.5, 138.6, 128.7 (2C), 123.5, 113.9 (2C), 112.2, 112.0, 55.4, 18.9; HRMS (ES): *m/z* calculated for $C_{15}H_{13}NO_3SNa$: 310.0514, found 310.0515 $[M+Na]^+$.

The reaction with 5 mol% of gold catalyst and 1.5 eq. of aminide was sluggish. After 48 h, purification (20% EtOAc in hexane) afforded oxazole ***3ia*** *as a brown solid (16.4 mg, 28%) with the same level of regioselectivity.*

2-(Dimethoxymethyl)-5-(ethylthio)-4-(4-methoxyphenyl)oxazole (3gf):

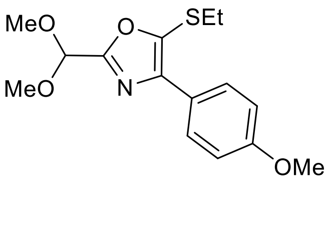

Following **GP3**, using alkynyl thioether **1g** (38.4 mg, 0.2 mmol), aminide **2f** (58.8 mg, 0.3 mmol) and gold catalyst (3.9 mg, 0.02 mmol) for 24 h. Purification by flash column chromatography using toluene afforded oxazole **3gf** as a pale-yellow liquid (27.8 mg, 45%); IR (neat): *v* = 2930, 2834, 1612, 1566, 1498, 1451, 1247, 1175, 1061, 967, 834, 795 cm^{-1};

mixture of regioisomers (15.2:1) observed by ^{1}H NMR (300 MHz, $CDCl_3$): δ = 8.06 (d, *J* = 9.0 Hz, 2H), 6.94 (d, *J* = 9.0 Hz, 2H), 5.50 (s, 1H), 3.83 (s, 3H), 3.48 (s, 6H), 2.86 (q, *J* = 7.4 Hz, 2H), 1.25 (t, *J* = 7.4 Hz, 3H); ^{13}C NMR (101 MHz, $CDCl_3$): δ = 160.5, 159.8, 142.5, 138.8, 128.7 (2C), 123.4, 113.8 (2C), 97.1, 55.4, 53.8 (2C), 30.2, 15.2; HRMS (ES): *m/z* calculated for $C_{15}H_{19}NO_4SNa$: 332.0932, found 332.0927 $[M+Na]^+$.

2-(Dimethoxymethyl)-4-(4-methoxyphenyl)-5-(phenylthio)oxazole (3hf):

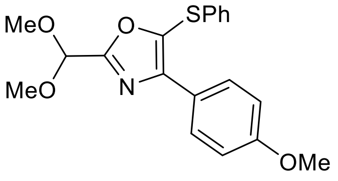

Following **GP3**, using alkynyl thioether **1h** (48.0 mg, 0.2 mmol), aminide **2f** (58.8 mg, 0.3 mmol) and gold catalyst (7.8 mg, 0.02 mmol) for 24 h. Purification by flash column chromatography using toluene afforded oxazole **3hf** as a pale-yellow liquid (28.8 mg, 40%); IR (neat): *v* = 2934, 2835, 1611, 1582, 1498, 1440, 1249, 1175, 1063, 968, 834, 738, 688 cm^{-1}; *mixture of regioisomers (11.4:1) observed by* ^{1}H NMR (300 MHz, $CDCl_3$): δ = 8.04 and 7.96 (d, *J* = 9.0 Hz, 2H), 7.25-7.13 (m, 5H), 6.89 (d, *J* = 9.0 Hz, 2H), 5.51 and 5.48 (s, 1H), 3.80 and 3.79 (s, 3H), 3.46 and 3.45 (s, 6H); ^{13}C NMR (101 MHz, $CDCl_3$): δ = 161.6, 160.2, 145.2, 135.9, 134.6, 129.5 (2C), 128.9 (2C), 127.7 (2C), 127.0, 122.9, 114.0 (2C), 97.0, 55.4, 53.8 (2C); HRMS (ES): *m/z* calculated for $C_{19}H_{20}NO_4S$: 358.1113, found 358.1110 $[M+H]^+$.

5-(Ethylthio)-2-(propan-2-ol)-(4-methoxyphenyl)oxazole (3gg):

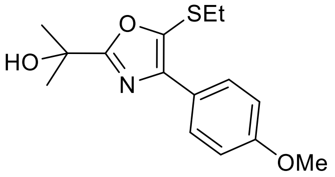

Following **GP3**, using alkynyl thioether **1g** (38.4 mg, 0.2 mmol), aminide **2g** (54.0 mg, 0.3 mmol) and gold catalyst (3.9 mg, 0.01 mmol) for 24 h. Purification by flash column chromatography using toluene afforded oxazole **3gg** as a pale-yellow liquid (24.1 mg, 41%); IR (neat): *v* = 3360, 2981, 2930, 1743, 1612, 1500, 1455, 1247, 1174, 1077, 1032, 973, 834, 757 cm^{-1}; *mixture of regioisomers (28.5:1) observed by* ^{1}H NMR (300 MHz, $CDCl_3$): δ = 8.03 (d, *J* = 8.9 Hz, 2H), 6.95 (d, *J* = 8.9 Hz, 2H), 3.84 (s, 3H), 3.03 (s, 1H), 2.84 (q, *J* = 7.4 Hz, 2H), 1.66 (s, 6H), 1.26 (t, *J* = 7.4 Hz, 3H); ^{13}C NMR (101 MHz, $CDCl_3$): δ = 169.8, 159.8, 142.5, 138.0, 128.6 (2C), 123.7, 113.9 (2C), 69.8, 55.4, 30.4, 28.7 (2C), 15.3; HRMS (ES): *m/z* calculated for $C_{15}H_{19}NO_3SNa$: 316.0983, found 316.0976 $[M+Na]^+$.

4-Butyl-5-(methylthio)-2-phenyloxazole (3ja):

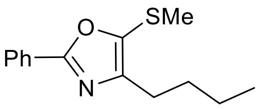

Following **GP3**, using alkynyl thioether **1j** (51.2 mg, 0.4 mmol), aminide **2a** (118.8 mg, 0.6 mmol) and gold catalyst (7.8 mg, 0.02 mmol) for 36 h. Purification by flash column chromatography using 2% EtOAc in hexane afforded oxazole **3ja** as a pale-yellow liquid (25.0 mg, 25%); IR (neat): v = 2956, 2927, 1631, 1577, 1478, 1343, 1155, 1024, 961, 749, 699 cm^{-1}; *mixture of regioisomers (3.2:1) observed by* ^{1}H NMR (300 MHz, $CDCl_3$): δ = 8.10-7.98 (m, 2H), 7.49-7.40 (m, 3H), 2.79 and 2.63 (t, *J* = 7.3 Hz, 2H), 2.42 and 2.39 (s, 3H), 1.67 (app. quint., *J* = 7.6 Hz, 2H), 1.39 (app. sext., *J* = 7.8 Hz, 2H), 0.97 and 0.95 (t, *J* = 7.3 Hz, 3H); ^{13}C NMR (101 MHz, $CDCl_3$): δ = 162.8, 146.8, 140.5, 130.6, 128.8 (2C), 127.6, 126.5 (2C), 31.2, 26.3, 22.5, 19.8, 14.0; HRMS (ES): *m/z* calculated for $C_{14}H_{18}NOS$: 248.1109, found 248.1103 $[M+H]^+$.

4-Butyl-5-(methylthio)-2-(naphthalen-2-yl)oxazole (3jc):

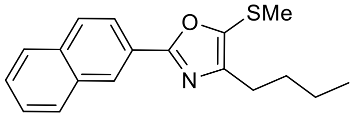

Following **GP3**, using alkynyl thioether **1j** (25.6 mg, 0.2 mmol), aminide **2c** (74.4 mg, 0.3 mmol) and gold catalyst (3.9 mg, 0.01 mmol) for 36 h. Purification by flash column chromatography using 3% EtOAc in hexane afforded oxazole **3jc** as a pale-yellow liquid (18.2 mg, 31%);

IR (neat): ν = 2956, 2926, 1719, 1651, 1578, 1463, 1189, 1130, 1078, 967, 861, 819, 752 cm^{-1}; *mixture of regioisomers (3.5:1) observed by* ^{1}H NMR (300 MHz, $CDCl_3$): δ = 8.55 and 8.50 (s, 1H), 8.13 (dd, *J* = 6.8, 1.8 Hz, 1H), 7.95-7.82 (m, 3H), 7.57-7.49 (m, 2H), 2.83 and 2.66 (t, *J* = 7.5 Hz, 2H), 2.46 and 2.43 (s, 3H), 1.71 (app. quint, *J* = 7.7 Hz, 2H), 1.43 (app. sext, *J* = 7.6 Hz, 2H), 0.98 and 0.97 (t, *J* = 7.3 Hz, 3H); ^{13}C NMR (101 MHz, $CDCl_3$): δ = 163.0, 147.0, 140.7, 134.3, 133.2, 128.9, 128.7, 128.0, 127.4, 126.9, 126.5, 124.9, 123.4, 31.3, 26.4, 22.5, 19.8, 14.0; HRMS (ES): *m/z* calculated for $C_{18}H_{20}NOS$: 298.1266, found 298.1254 [M+H]$^+$.

4-(3,4-Dimethoxyphenyl)-5-(ethylthio)-2-phenyloxazole (3ka):

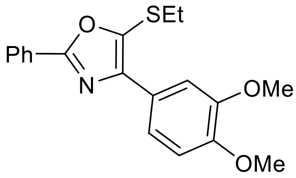

Following **GP3**, using alkynyl thioether **1k** (44.4 mg, 0.2 mmol), aminide **2a** (59.4 mg, 0.3 mmol) and gold catalyst (7.8 mg, 0.02 mmol) for 24 h. Purification by flash column chromatography using 10% EtOAc in hexane afforded oxazole **3ka** as a pale-yellow liquid (47.8 mg, 70%); IR (neat): *ν* = 2969, 2928, 1551, 1502, 1448, 1251, 1225, 1133, 1025, 861, 765, 722, 689 cm^{-1}; *mixture of regioisomers (13.5:1) observed by* ^{1}H NMR (300 MHz, $CDCl_3$): δ = 8.18-8.07 (m, 2H), 7.87-7.74 (m, 2H), 7.52-7.42 (m, 3H), 6.95 (d, *J* = 8.3 Hz, 1H), 3.99 (s, 3H), 3.93 (s, 3H), 2.92 (q, *J* = 7.4 Hz, 2H), 1.32 (t, *J* = 7.4 Hz, 3H); ^{13}C NMR (101 MHz, $CDCl_3$): δ = 162.5, 149.2, 148.8, 143.8, 137.9, 130.7, 128.9 (2C), 127.3, 126.6 (2C), 124.2, 120.1, 111.0, 110.3, 56.03, 55.99, 30.5, 15.4; HRMS (ES): *m/z* calculated for $C_{19}H_{20}NO_3S$: 342.1164; found 342.1163 [M+H]$^+$.

4-(3,4-Dimethoxyphenyl)-5-(ethylthio)-2-(naphthalen-2-yl)oxazole (3kc):

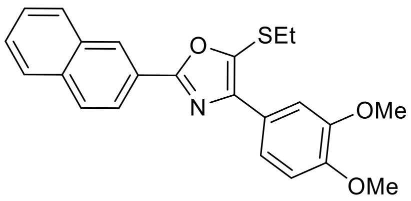

Following **GP3**, using alkynyl thioether **1k** (44.4 mg, 0.2 mmol), aminide **2c** (74.4 mg, 0.3 mmol) and gold catalyst (3.9 mg, 0.01 mmol) for 22 h. Purification by flash column chromatography using 20% EtOAc in hexane afforded oxazole **3kc** as a pale-yellow liquid (61.0 mg, 78%); IR (neat): *ν* = 2987, 2928, 1685, 1609, 1525, 1503, 1449, 1251, 1224, 1125, 1083, 989, 936, 868, 815, 767, 711 cm^{-1}; *mixture of regioisomers (16.1:1) observed by* ^{1}H NMR (300 MHz, $CDCl_3$): δ = 8.62 (s, 1H), 8.22 (dd, *J* = 6.9, 1.7 Hz, 1H), 7.99-7.90 (m, 2H), 7.89-7.78 (m, 3H), 7.57-7.50 (m, 2H), 6.96 (d, *J* = 8.3 Hz, 1H), 4.01 (s, 3H), 3.94 (s, 3H), 2.96 (q, *J* = 7.4 Hz, 2H), 1.35 (t, *J* = 7.4 Hz, 3H); ^{13}C NMR (101 MHz, $CDCl_3$): δ = 162.6, 149.2, 148.8, 144.0, 138.2, 134.4, 133.1, 128.8, 128.7, 128.0, 127.5, 126.9, 126.7, 124.5, 124.1, 123.5, 120.2, 111.0, 110.4, 56.03, 55.97, 30.5, 15.4; HRMS (ES): *m/z* calculated for $C_{23}H_{22}NO_3S$: 392.1320; found 392.1316 [M+H]$^+$.

***tert*-Butyl-5-(ethylthio)-4-[(4-methoxyphenyl)oxazol-2-yl)methyl]carbamate (3gh):**

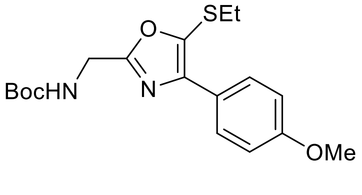

Following **GP3**, using alkynyl thioether **1g** (38.4 mg, 0.2 mmol), aminide **2h** (75.3 mg, 0.3 mmol) and gold catalyst (3.9 mg, 0.01 mmol) for 24 h. Purification by flash column chromatography using 30% EtOAc in hexane afforded oxazole **3gh** as a pale-yellow solid (52.4 mg, 72%); mp. 62-64 °C; IR (neat): *ν* = 3675, 3358, 2973, 2925, 1686, 1611, 1572, 1524, 1497, 1247, 1154, 1081, 1051, 1037, 937, 867, 832, 785 cm^{-1}; *mixture of regioisomers (23.0:1) observed by* ^{1}H NMR (300 MHz, $CDCl_3$): δ = 8.01 (d, *J* = 8.9 Hz, 2H), 6.94 (d, *J* = 8.9 Hz, 2H), 5.29 (br s, 1H), 4.45 (d, *J* = 5.3 Hz, 2H), 3.83 (s, 3H), 2.82 (q, *J* = 7.4 Hz, 2H), 1.47 (s, 9H), 1.25 (t, *J* = 7.4 Hz, 3H); ^{13}C NMR (101 MHz, $CDCl_3$): δ = 162.3, 159.7, 155.7, 142.7, 138.2, 128.5 (2C), 123.6, 113.9 (2C), 80.2, 55.4, 38.6, 30.3, 28.4 (3C), 15.2; HRMS (ES): *m/z* calculated for $C_{18}H_{24}N_2O_4SNa$: 387.1354, found 387.1349 [M+Na]$^+$.

***tert*-Butyl [(4-(4-methoxyphenyl)-5-(phenylthio)oxazol-2-yl)methyl]carbamate (3hh):**

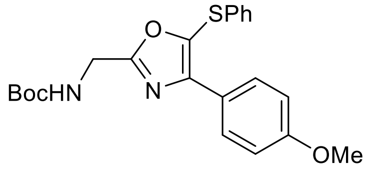

Following **GP3**, using alkynyl thioether **1h** (48.0 mg, 0.2 mmol), aminide **2h** (75.3 mg, 0.3 mmol) and gold catalyst (3.9 mg, 0.01 mmol) for 24 h Purification by flash column chromatography using 30% EtOAc in hexane afforded oxazole **3hh** as a pale-yellow solid (64.1 mg, 78%); IR (neat): *v* = 3371, 2990, 2968, 2928, 1683, 1610, 1499, 1439, 1280, 1247, 1172, 974, 840, 734, 684 cm^{-1}; *mixture of regioisomers (10.3:1) observed by* ^{1}H NMR (300 MHz, $CDCl_3$): δ = 8.02 and 7.93 (d, *J* = 8.9 Hz, 2H), 7.32-7.15 (m, 5H), 6.93 (d, *J* = 8.9 Hz, 2H), 5.24 (br s, 1H), 4.53 (d, *J* = 5.3 Hz, 2H), 3.82 (s, 3H), 1.46 (s, 9H); ^{13}C NMR (101 MHz, $CDCl_3$): δ = 163.5, 160.1, 155.6, 145.3, 135.2, 134.8, 129.5 (2C), 128.7 (2C), 127.6 (2C), 126.9, 123.1, 114.0 (2C), 80.3, 55.4, 38.7, 28.4 (3C); HRMS (ES): *m/z* calculated for $C_{22}H_{24}N_2O_4SNa$: 435.1354, found 435.1347 $[M+Na]^+$.

tert-Butyl ((4-(4-methoxyphenyl)-5-(methylthio)oxazol-2-yl)methyl)carbamate (3ih):

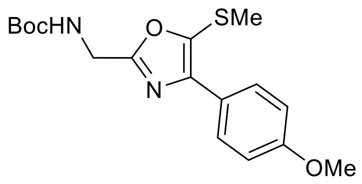

Following **GP3**, using alkynyl thioether **1i** (541.1 mg, 3.0 mmol), aminide **2h** (1158.7 mg, 4.6 mmol) and gold catalyst (12.0 mg, 0.06 mmol, 2 mol%) for 8 h. Purification by flash column chromatography (35% EtOAc in hexane) afforded oxazole **3ih** as a pale yellow solid (617.6 mg, 58%); mp. 88-90 °C; IR (neat): ν = 3360, 2935, 1688, 1523, 1498, 1244, 1267, 1163, 1077 cm^{-1}; *mixture of rotamers observed by* ^{1}H NMR (300 MHz, $CDCl_3$): δ = 7.97 (d, *J* = 8.9 Hz, 2H), 6.95 (d, *J* = 8.9 Hz, 2H), 5.22 and 5.02 (br s, 1H), 4.45 (br d, *J* = 5.3 Hz, 2H), 3.84 (s, 3H), 2.41 (s, 3H), 1.48 (s, 9H); ^{13}C NMR (101 MHz, $CDCl_3$): δ = 162.1, 159.7, 155.7, 141.5, 128.4 (2C), 123.6, 114.4, 114.0 (2C), 80.3, 55.4, 38.6, 28.5 (3C), 18.7; HRMS (ES): *m/z* calculated for $C_{17}H_{22}N_2O_4SNa$: 373.1198, found 373.1202 $[M+Na]^+$.

***tert*-Butyl [(4-(3,4-dimethoxyphenyl)-5-(ethylthio)oxazol-2-yl)methyl]carbamate (3kh):**

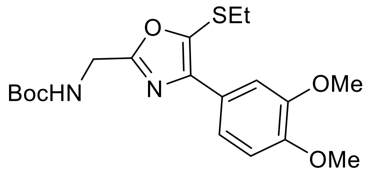

Following **GP3**, using alkynyl thioether **1k** (44.4 mg, 0.2 mmol), aminide **1h** (75.3 mg, 0.3 mmol) and gold catalyst (3.9 mg, 0.01 mmol) for 24 h. Purification by flash column chromatography using 30% EtOAc in hexane afforded oxazole **3kh** as a pale-yellow liquid (60.0 mg, 76%); IR (neat): *v* = 2957, 2930, 1588, 1544, 1501, 1463, 1252, 1225, 1132, 1026, 862, 818, 755, 730 cm^{-1}; *mixture of regioisomers (50.0:1) observed by* ^{1}H NMR (300 MHz, $CDCl_3$): δ = 7.70-7.60 (m, 2H), 6.89 (d, *J* = 8.2 Hz, 1H), 5.30 (br s, 1H), 4.44 (d, *J* = 5.3 Hz, 2H), 3.92 (s, 3H), 3.89 (s, 3H), 2.82 (q, *J* = 7.4 Hz, 2H), 1.45 (s, 9H), 1.24 (t, *J* = 7.4 Hz, 3H); ^{13}C NMR (101 MHz, $CDCl_3$): δ = 162.3, 155.6, 149.1, 148.8, 142.6, 138.3, 123.8, 119.9, 111.0, 110.1, 80.2, 56.0, 55.9, 38.5, 30.3, 28.4 (3C), 15.3; HRMS (ES): *m/z* calculated for $C_{19}H_{26}N_2O_5SNa$: 417.1460; found 417.1466 $[M+Na]^+$.

***tert*-Butyl(*S*)-1-[(5-(ethylthio)-4-(4-methoxyphenyl)oxazol-2-yl]-2-phenylethylcarbam-ate (3gk):**

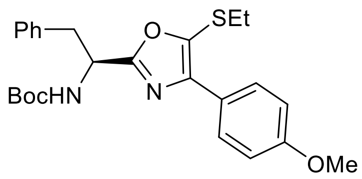

Following **GP3**, using alkynyl thioether **1g** (38.4 mg, 0.2 mmol), aminide **2k** (102.0 mg, 0.3 mmol) and gold catalyst (3.9 mg, 0.01 mmol) for 20 h. Purification by flash column chromatography using 20-30% EtOAc in hexane afforded oxazole **3gk** as a pale-yellow liquid (53.0 mg, 58%); $[\alpha]_D^{20}$ = -29.6° (*c* = 1.0, $CHCl_3$); IR (neat): *v* = 3675, 3340, 2973, 2927, 1714, 1612, 1500, 1455, 1367, 1250, 1175, 1077, 1055, 973, 836, 732, 701 cm^{-1}; *mixture of regioisomer (19.1:1) and rotamers (2.3:1) observed by* ^{1}H NMR (300 MHz, $CDCl_3$):

δ = 8.00 and 7.81 (d, *J* = 8.7 Hz, 2H), 7.30-7.18 (m, 3H), 7.14-7.04 (m, 2H), 6.95 (d, *J* = 8.9 Hz, 2H), 5.32-4.95 (m, 2H), 3.84 (s, 3H), 3.25 (d, *J* = 5.8 Hz, 2H), 2.75 (q, *J* = 7.4 Hz, 2H), 1.44 (s, 9H), 1.20 (t, *J* = 7.4 Hz, 3H); ^{13}C NMR (101 MHz, $CDCl_3$): *one quaternary aromatic resonance could not be identified* δ = 164.5, 159.7, 155.0, 142.8, 136.1, 129.5 (2C), 128.6 (4C), 127.0, 123.7, 113.9 (2C), 80.1, 55.4, 50.5, 40.5, 30.4, 28.4 (3C), 15.2; HRMS (ES): *m/z* calculated for $C_{25}H_{30}N_2O_4SNa$: 477.1824, found 477.1829 $[M+Na]^+$.

***tert*-Butyl (*S*)-2-[5-(methylthio)-4-phenyloxazol-2-yl]pyrrolidine-1-carboxylate (3ai):**

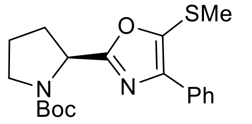

Following **GP3**, using alkynyl thioether **1a** (29.6 mg, 0.2 mmol), aminide **2i** (87.3 mg, 0.3 mmol) and gold catalyst (3.9 mg, 0.01 mmol) for 1 h. Purification by flash column chromatography using 30% EtOAc in hexane afforded oxazole **3ai** as a colourless liquid (39.7 mg, 55%); $[\alpha]_D^{20}$ = -80.6° (*c* = 2.0, $CHCl_3$); IR (neat): ν = 2974, 2926, 1697, 1481, 1391, 1366, 1250, 1162, 1117, 1076, 978, 772, 696 cm^{-1}; *mixture of regioisomers (12.4:1) and rotamers (2.5:1) observed by* ^{1}H NMR (300 MHz, $CDCl_3$): δ = 8.02 and 7.83 (d, *J* = 7.4 Hz, 2H), 7.53-7.27 (m, 3H), 5.12-4.80 (m, 1H), 3.79-3.37 (m, 2H), 2.52 and 2.41 (s, 3H), 2.37-2.21 (m, 1H), 2.20-2.02 (m, 2H), 2.01-1.89 (m, 1H), 1.46 and 1.30 (s, 9H); ^{13}C NMR (101 MHz, $CDCl_3$): δ = 166.5, 154.1, 141.4, 131.3, 128.5 (2C), 128.2, 127.1 (2C), 125.3, 80.0, 55.3, 46.5, 32.5, 28.4 (3C), 23.8, 18.6; HRMS (ES): *m/z* calculated for $C_{19}H_{24}N_2O_3SNa$: 383.1405, found 383.1392 $[M+Na]^+$.

***tert*-Butyl (2*S*,4*R*)-[(4-benzyloxy)-2-(5-(methylthio)-4-phenyloxazol-2-yl]pyrrolidine-1-carboxylate (3aj):**

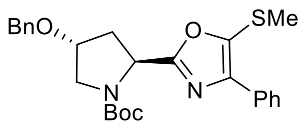

Following **GP3**, using alkynyl thioether **1a** (29.6 mg, 0.2 mmol), aminide **2j** (119.0 mg, 0.3 mmol) and gold catalyst (3.9 mg, 0.01 mmol) for 1 h. Purification by flash column chromatography using 30% EtOAc in hexane afforded oxazole **3aj** as a colourless liquid (55.0 mg, 59%); $[\alpha]_D^{20}$ = -41.6° (*c* = 0.5, $CHCl_3$); IR (neat): ν = 2975, 2928, 1697, 1392, 1365, 1159, 1089, 977, 770, 695 cm^{-1}; *mixture of regioisomers (22.0:1) and rotamers (2.6:1) observed by* ^{1}H NMR (300 MHz, $CDCl_3$): δ = 8.01 and 7.83 (d, *J* = 7.5 Hz, 2H), 7.52-7.27 (m, 8H), 5.14 and 5.04 (t, *J* = 7.8 Hz, 1H), 4.56 (ABq, *J* = 12.3 Hz, 2H), 4.29 (br s, 1H), 3.89-3.59 (m, 2H), 2.61-2.47 (m, 1H), 2.42 (s, 3H), 2.37-2.25 (m, 1H), 1.45 and 1.29 (s, 9H); ^{13}C NMR (101 MHz, $CDCl_3$): *one quaternary aromatic resonance could not be identified* δ = 166.0, 154.1, 141.5, 137.9, 131.2, 128.63 (5C), 128.60, 128.3, 128.0, 127.8, 127.1, 80.3, 76.0, 71.2, 54.2, 51.7, 38.5, 38.3 (3C), 18.6; HRMS (ES): *m/z* calculated for $C_{26}H_{30}N_2O_4SNa$: 489.1824, found 489.1815 $[M+Na]^+$.

***tert*-Butyl(*S*)-2-[4-(4-methoxyphenyl)-5-(methylthio)oxazol-2-yl]pyrrolidine-1-carbox-ylate (3ii):**

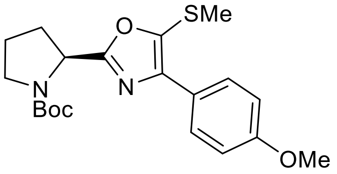

Following **GP3**, using alkynyl thioether **1i** (35.6 mg, 0.2 mmol), aminide **2i** (87.3 mg, 0.3 mmol) and gold catalyst (3.9 mg, 0.01 mmol) for 2 h. Purification by flash column chromatography using 30-40% EtOAc in hexane afforded oxazole **3ii** as a pale-yellow liquid (56.2 mg, 72%); $[\alpha]_D^{20}$ = -82.4° (*c* =0.5, $CHCl_3$); IR (neat): *v* = 2974, 2931, 1695, 1612, 1499, 1389, 1365, 1248, 1159, 1116, 1032, 977, 835, 770, 731 cm^{-1}; *mixture of regioisomers (18.6:1) and rotamers (2.6:1) observed by* ^{1}H NMR (300 MHz, $CDCl_3$): δ = 7.96 and 7.79 (d, *J* = 8.6 Hz, 2H), 6.94 (d, *J* = 8.6 Hz, 2H), 5.04-4.80 (m, 1H), 3.82 (s, 3H), 3.71-3.59 (m, 1H), 3.58-3.41 (m, 1H), 2.38 (s, 3H), 2.33-2.19 (m, 1H), 2.18-2.04 (m, 2H), 1.99-1.87 (m, 1H), 1.45 and 1.29 (s, 9H); ^{13}C NMR (101 MHz, $CDCl_3$): δ = 166.3, 159.6, 154.0, 141.5, 138.1, 128.4 (2C), 123.8, 113.9 (2C), 79.9, 55.4, 55.3, 46.5, 32.5, 28.3 (3C), 23.8, 18.8; HRMS (ES): *m/z* calculated for $C_{20}H_{27}N_2O_4S$: 391.1692, found 391.1686 $[M+H]^+$.

***tert*-Butyl(2*S*,4*R*)-[4-(benzyloxy)-2-(5-(ethylthio)-4-(4-methoxyphenyl)oxazol-2-yl]-pyrrolidine-1-carboxylate (3gj):**

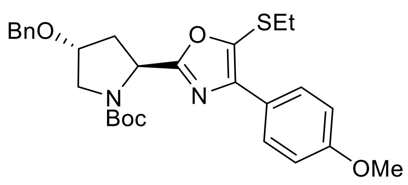

Following **GP3**, using alkynyl thioether **1g** (38.4 mg, 0.2 mmol), aminide **2j** (119.0 mg, 0.3 mmol) and gold catalyst (3.9 mg, 0.01 mmol) for 2 h. Purification by flash column chromatography using 30% EtOAc in hexane afforded oxazole **3gj** as a pale-yellow liquid (67.6 mg, 66%); $[\alpha]_D^{20}$ = -50.0° (*c* = 1.0, $CHCl_3$); IR (neat): *v* = 2973, 2928, 1697, 1611, 1499, 1391, 1365, 1248, 1159, 1079, 975, 836, 737, 698 cm^{-1}; *mixture of regioisomers (20.0:1) and rotamers (2.6:1) observed by* ^1H NMR (300 MHz, $CDCl_3$): δ = 8.02 and 7.86 (d, *J* = 8.9 Hz, 2H), 7.40-7.27 (m, 5H), 6.95 (d, *J* = 8.6 Hz, 2H), 5.12 and 5.03 (t, *J* = 7.5 Hz, 1H), 4.56 (ABq, *J* = 12.2 Hz, 2H), 4.29 (br s, 1H), 3.84 (s, 3H), 3.83-3.69 (m, 2H), 2.81 (q, *J* = 7.3 Hz, 2H), 2.60-2.43 (m, 1H), 2.36-2.21 (m, 1H), 1.44 and 1.29 (s, 9H), 1.25 (t, *J* = 7.3 Hz, 3H); ^{13}C NMR (101 MHz, $CDCl_3$): *one quaternary aromatic resonance could not be identified* δ = 166.0, 159.7, 154.1, 142.8, 137.9, 128.6 (4C), 127.9, 127.8 (2C), 123.8, 113.9 (2C), 80.2, 76.0, 71.2, 55.3, 54.1, 51.6, 38.4, 30.4, 28.3 (3C), 15.2; HRMS (ES): *m/z* calculated for $C_{28}H_{34}N_2O_5SNa$: 533.2086, found 533.2095 $[M+Na]^+$.

***tert*-Butyl (*S*)-2-[4-(3,4-dimethoxyphenyl)-5-(ethylthio)oxazol-2-yl]pyrrolidine-1-carbo-xylate (3ki):**

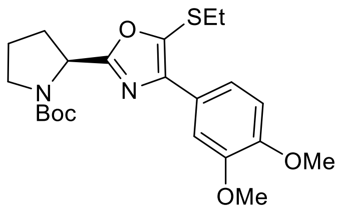

Following **GP3**, using alkynyl thioether **1k** (88.8 mg, 0.4 mmol), aminide **2i** (174.6 mg, 0.6 mmol) and gold catalyst (7.8 mg, 0.02 mmol) for 2 h. Purification by flash column chromatography using 30% EtOAc in hexane afforded oxazole **3ki** as a pale-yellow liquid (125.1 mg, 72%); $[\alpha]_D^{20}$ = –80.8° (*c* = 1.0, $CHCl_3$); IR (neat): *v* = 2972, 2931, 1695, 1590, 1504, 1453, 1389, 1253, 1159, 1118, 1025, 895, 859, 765, 734 cm^{-1}; *rotamers (2.2:1) observed by* ^1H NMR (300 MHz, $CDCl_3$): δ = 7.73-7.58 (m, 2H), 6.89 (d, *J* = 8.2 Hz, 1H), 5.04-4.82 (m, 1H), 3.92 (s, 3H), 3.89 (s, 3H), 3.73-3.60 (m, 1H), 3.57-3.42 (m, 1H), 2.81 (q, *J* = 7.4 Hz, 2H), 2.40-2.21 (m, 1H), 2.18-2.03 (m, 2H), 1.98-1.89 (m, 1H), 1.44 and 1.29 (s, 9H), 1.23 (t, *J* = 7.1 Hz, 3H); ^{13}C NMR (101 MHz, $CDCl_3$): δ = 166.4, 154.0, 149.1, 148.8, 142.6, 137.2, 124.1, 119.9, 111.0, 110.2, 79.9, 56.0 (2C), 55.1, 46.5, 32.5, 30.3, 28.3 (3C), 23.7, 15.3; HRMS (ES): *m/z* calculated for $C_{22}H_{30}N_2O_5SNa$: 457.1773; found 457.1764 $[M+Na]^+$.

***tert*-Butyl (2*S*,4*R*)-[4-(benzyloxy)-2-(4-(3,4-dimethoxyphenyl)-5-(ethylthio)oxazol-2-yl]-pyrrolidine-1-carboxylate (3kj):**

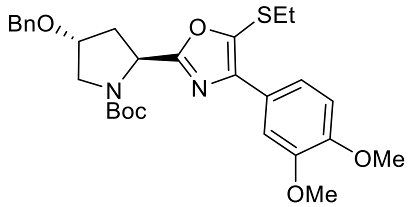

Following **GP3**, using alkynyl thioether **1k** (88.8 mg, 0.4 mmol), aminide **2j** (238.2 mg, 0.6 mmol) and gold catalyst (7.8 mg, 0.02 mmol) for 2 h. Purification by flash column chromatography using 30% EtOAc in hexane afforded oxazole **3kj** as a pale-yellow liquid (173.5 mg, 80%); $[\alpha]_D^{20}$= –43.2 ° (*c* = 0.5, $CHCl_3$); IR (neat): *v* = 2973, 2918, 1609, 1594, 1547, 1483, 1442, 1381, 1321, 1155, 1097, 1068, 1047, 1023, 954, 762, 710, 688, 673 cm^{-1}; *rotamers (2.3:1) observed by* ^1H NMR (300 MHz, $CDCl_3$): δ = 7.73-7.61 (m, 2H), 7.39-7.27 (m, 5H), 6.90 (d, *J* = 8.0 Hz, 1H), 5.12 and 5.03 (t, *J* = 7.6 Hz, 1H), 4.54 (ABq, *J* = 12.3 Hz, 2H), 4.27 (br s, 1H), 3.93 (s, 3H), 3.90 (s, 3H), 3.84-3.65 (m, 2H), 2.81 (q, *J* = 7.4 Hz, 2H), 2.60-2.41 (m, 1H), 2.38-2.19 (m, 1H), 1.43 and 1.29 (s, 9H), 1.24 (t, *J* = 7.4 Hz, 3H); ^{13}C NMR (101 MHz, $CDCl_3$): *one quaternary aromatic resonance could not be identified* δ = 165.8, 154.0, 149.1, 148.8, 142.7, 137.8, 128.5 (3C), 127.8, 127.7, 124.0, 119.9, 111.0, 110.2, 80.2, 75.9, 71.0, 55.9

(2C), 54.1, 51.6, 38.4, 30.3, 28.2 (3C), 15.2; HRMS (ES) *m/z* calculated for $C_{29}H_{37}N_2O_6S$: 541.2372; found 541.2373 $[M+H]^+$.

5-(Methylthio)-2-(naphthalen-2-yl)-4-(2,4,6-trimethylphenyl)oxazole (3lc):

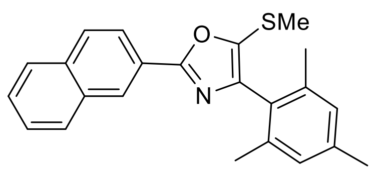

Following **GP3**, using alkynyl thioether **1l** (38.0 mg, 0.2 mmol), aminide **2c** (74.4 mg, 0.3 mmol) and gold catalyst (7.8 mg, 0.02 mmol) for 24 h. Purification by flash column chromatography using 30% EtOAc in hexane afforded oxazole **3lc** as a pale-yellow solid (35.4 mg, 49%); mp. 52-54 °C; IR (neat): *ν* = 2924, 1700, 1628, 1573, 1535, 1409, 1352, 1280, 1163, 1091, 1060, 925, 813, 748, 717, 664 cm^{-1}; ^1H NMR (300 MHz, $CDCl_3$): δ = 8.63 (s, 1H), 8.20 (dd, *J* = 7.0, 1.6 Hz, 1H), 8.00-7.84 (m, 3H), 7.60-7.50 (m, 2H), 6.97 (s, 2H), 2.42 (s, 3H), 2.34 (s, 3H), 2.21 (s, 6H); ^{13}C NMR (101 MHz, $CDCl_3$): *one quaternary aromatic resonance could not be identified* δ = 163.2, 143.5, 142.1, 138.6, 137.7, 134.4, 133.2, 128.9, 128.8, 128.4 (2C), 128.0, 127.6, 127.5, 126.9, 126.5, 124.8, 123.4, 21.3, 20.4 (2C), 18.5; HRMS (ES): *m/z* calculated for $C_{23}H_{22}NOS$: 360.1422; found 360.1418 $[M+H]^+$.

tert-Butyl ((4-(4-(diethylamino)phenyl)-5-(methylthio)oxazol-2-yl)methyl)carbamate (3mh)

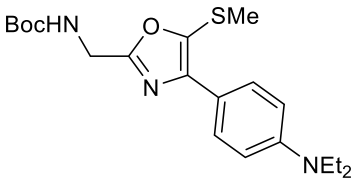

Following **GP3**, using alkynyl thioether **1m** (109.8 mg, 0.5 mmol), aminide **2h** (188.0 mg, 0.8 mmol) and gold catalyst (10.0 mg, 0.03 mmol) for 45 minutes. Purification by flash column chromatography (30% EtOAc in hexane) afforded oxazole **3mh** as a brown oil (149.3 mg, 76%); IR (neat): ν = 3353, 2972, 2929, 1706, 1612, 1504, 1359, 1265, 1158; ^1H NMR (300 MHz, $CDCl_3$): δ = 7.89 (d, *J* = 9.0 Hz, 2H), 6.71 (d, *J* = 9.0 Hz, 2H), 5.24 and 5.00 (br s, 1H), 4.44 (d, *J* = 5.1 Hz, 2H), 3.39 (q, *J* = 7.1 Hz, 4H), 2.39 (s, 3H), 1.48 (s, 9H), 1.18 (t, *J* = 7.1 Hz, 6H); ^{13}C NMR (101 MHz, $CDCl_3$): δ = 161.8, 155.6, 147.7, 142.4, 137.6, 128.3 (2C), 117.8, 111.3 (2C), 80.1, 44.4 (2C), 38.6, 28.4 (3C), 18.9, 12.7 (2C); HRMS (ES): *m/z* calculated for $C_{20}H_{30}N_3O_3S$: 392.2008, found 392.2006 $[M+H]^+$.

5-(Methylthio)-2-phenyl-4-(1-tosyl-1H-indol-3-yl)oxazole (3na)

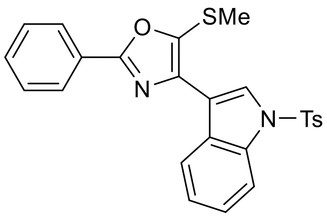

Following **GP3**, using alkynyl thioether **1n** (34.1 mg, 0.1 mmol), aminide **2a** (59.5 mg, 0.3 mmol) and gold catalyst (4.0 mg, 0.01 mmol) for 4.5 h. A pre-chromatographic filtration through a short pad of silica, eluting with EtOAc was used. Purification by flash column chromatography (10% EtOAc in hexane) afforded oxazole **3na** as an off white solid (39.8 mg, 87%); mp 148-150 °C; IR (neat): ν = 3053, 2923, 1615, 1593, 1445, 1368, 1168; ^1H NMR (300 MHz, $CDCl_3$): δ = 8.45-8.43 (m, 1H), 8.21 (s, 1H), 8.16-8.13 (m, 2H), 8.05-8.02 (m, 1H), 7.81 (d, *J* = 8.4 Hz, 2H), 7.51-7.48 (m, 3H), 7.41-7.31 (m, 2H), 7.22 (d, *J* = 8.1 Hz, 2H), 2.52 (s, 3H), 2.33 (s, 3H); ^{13}C NMR (101 MHz, $CDCl_3$): δ = 162.9, 145.2, 140.5, 138.2, 135.2, 135.2, 130.9, 130.1 (2C), 129.5, 129.0 (2C), 127.3, 127.1 (2C), 126.6 (2C), 125.2, 124.8, 123.9, 122.8, 113.6, 113.6, 21.7, 18.7; HRMS (ES): *m/z* calculated for $C_{25}H_{20}N_2O_3S_2Na$: 483.0813, found 483.0817 $[M+Na]^+$.

2-(2-Bromophenyl)-5-(methylthio)-4-(1-tosyl-1H-indol-3-yl)oxazole (3nb)

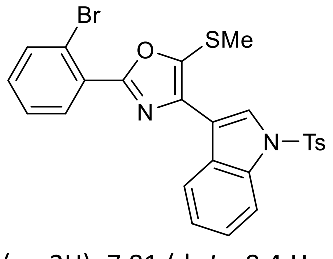

Following **GP3**, using alkynyl thioether **1n** (34.3 mg, 0.1 mmol), aminide **2b** (78.0 mg, 0.3 mmol) and gold catalyst (3.9 mg, 0.01 mmol) for 3 h. Purification by flash column chromatography (18% EtOAc in hexane) afforded oxazole **3nb** as colourless crystals (43.2 mg, 80%); mp 142-144 °C; IR (neat): ν = 2934, 1690, 1444, 1366, 1166, 1136, 1102; ^{1}H NMR (300 MHz, $CDCl_3$): δ = 8.50-8.48 (m, 1H), 8.21 (s, 1H), 8.07-8.02 (m, 2H), 7.81 (d, *J* = 8.4 Hz, 2H), 7.75 (dd, *J* = 8.0, 1.1 Hz, 1H), 7.44 (app td, *J* = 7.5, 1.2 Hz, 1H), 7.40-7.29 (m, 3H), 7.22 (d, *J* = 8.1 Hz, 2H), 2.56 (s, 3H), 2.33 (s, 3H); ^{13}C NMR (101 MHz, $CDCl_3$): δ = 161.2, 145.2, 141.2, 137.1, 135.2, 135.1, 134.9, 131.5, 131.2, 130.0 (2C), 129.4, 128.0, 127.6, 127.0 (2C), 125.2, 124.7, 123.9, 122.9, 121.1, 114.4, 113.5, 21.7, 18.4; HRMS (ES): *m/z* calculated for $C_{25}H_{20}N_2O_3S_2{}^{79}Br$: 539.0099, found 539.0095 $[M+H]^+$.

tert-Butyl ((5-(methylthio)-4-(1-tosyl-1H-indol-3-yl)oxazol-2-yl)methyl)carbamate (3nh)

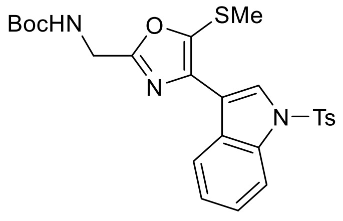

Following **GP3**, using alkynyl thioether **1n** (34.3 mg, 0.1 mmol), aminide **2h** (75.8 mg, 0.3 mmol) and gold catalyst (4.0 mg, 0.01 mmol) for 1.5 h. Purification by flash column chromatography (30% EtOAc in hexane) afforded oxazole **3nh** as a white solid (48.7 mg, 94%); mp 104-106 °C; IR (neat): ν = 3415, 2981, 1692, 1594, 1511, 1367, 1166, 1136, 1102, 997; ^{1}H NMR (300 MHz, $CDCl_3$): δ = 8.25 (d, *J* = 7.5 Hz, 1H), 8.12 (s, 1H), 8.02-7.99 (m, 1H), 7.78 (d, *J* = 8.4 Hz, 2H), 7.38-7.28 (m, 2H), 7.20 (d, *J* = 8.1 Hz, 2H), 5.17 (br s, 1H), 4.49 (d, *J* = 5.7 Hz, 2H), 2.44 (s, 3H), 2.33 (s, 3H), 1.49 (s, 9H); ^{13}C NMR (101 MHz, $CDCl_3$): δ = 162.7, 155.7, 145.2, 141.0, 136.9, 135.1, 135.1, 130.0 (2C), 129.3, 127.0 (2C), 125.2, 124.8, 123.7, 122.6, 113.5, 113.3, 80.4, 38.6, 28.5 (3C), 21.7, 18.5; HRMS (ES): *m/z* calculated for $C_{25}H_{27}N_3O_5S_2Na$: 536.1314, found 536.1318 $[M+Na]^+$.

Methyl 2-(5-(methylthio)-4-(1-tosyl-1H-indol-3-yl)oxazol-2-yl)benzoate (3nl)

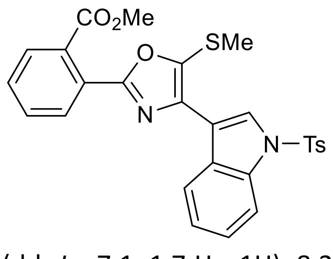

Following **GP3**, using alkynyl thioether **1n** (33.6 mg, 0.1 mmol), aminide **2l** (77.4 mg, 0.3 mmol) and gold catalyst (4.0 mg, 0.01 mmol) for 6 h. Purification by flash column chromatography (30% EtOAc in hexane) afforded oxazole **3nl** as a pale yellow oil (38.5 mg, 74%); IR (neat): ν = 2926, 1728, 1597, 1445, 1371, 1289, 1172, 1133, 1103; *mixture of regioisomers (18.3:1) observed by* ^{1}H NMR (300 MHz, $CDCl_3$): δ = 8.39 (dd, *J* = 7.1, 1.7 Hz, 1H), 8.22 (s, 1H), 8.02 (d, *J* = 7.3 Hz, 2H), 7.82 (d, *J* = 8.4 Hz, 2H), 7.70 (d, *J* = 7.2, 1.5 Hz, 1H), 7.63-7.52 (m, 2H), 7.40-7.29 (m, 2H), 7.23 (d, *J* = 8.1 Hz, 2H), 3.88 (s, 3H), 2.51 (s, 3H), 2.34 (s, 3H); ^{13}C NMR (101 MHz, $CDCl_3$): δ = 169.0, 161.5, 145.2, 141.2, 137.9, 135.2, 135.1, 132.2, 131.0, 130.5, 130.1 (2C), 129.4, 129.1, 129.1, 127.0 (2C), 125.9, 125.2, 124.7, 123.8, 123.0, 113.5, 113.4, 52.9, 21.7, 18.5; HRMS (ES): *m/z* calculated for $C_{27}H_{23}N_2O_5S_2$: 519.1048, found 519.1053 $[M+H]^+$.

5-(Methylthio)-2-(pyridin-2-yl)-4-(1-tosyl-1H-indol-3-yl)oxazole (3nm)

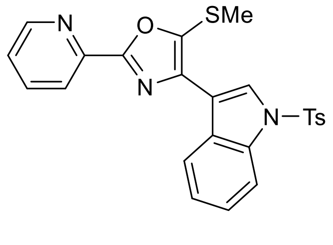

Following **GP3**, using alkynyl thioether **1n** (34.3 mg, 0.1 mmol), aminide **2m** (59.9 mg, 0.3 mmol) and gold catalyst (3.9 mg, 0.01 mmol) for 8 h. Purification by flash column chromatography (50% EtOAc in hexane) afforded oxazole **3nm** as an off-white solid (22.4 mg, 48%); mp 125-126 °C; IR (neat): ν = 2924, 1588, 1439, 1375, 1169, 1136, 1080, 997; ^{1}H NMR (300 MHz, $CDCl_3$): δ = 8.79-8.77 (m, 1H), 8.43-8.40 (m, 1H),

8.27 (br d, *J* = 7.9 Hz, 1H), 8.24 (s, 1H), 8.05-8.02 (m, 1H), 7.89-7.80 (m, 3H), 7.42-7.33 (m, 3H), 7.22 (br d, *J* = 8.1 Hz, 2H), 2.55 (s, 3H), 2.33 (s, 3H); ^{13}C NMR (101 MHz, $CDCl_3$): δ = 161.5, 150.2, 146.0, 145.3, 142.5, 138.2, 137.1, 135.2, 135.1, 130.1 (2C), 129.3, 127.1 (2C), 125.2, 125.1, 125.0, 123.8, 122.8, 122.5, 113.6, 113.3, 21.7, 18.5; HRMS (ES): *m/z* calculated for $C_{24}H_{20}N_3O_3S_2$: 462.0946, found 462.0947 $[M+H]^+$.

Methyl 2-(5-(methylthio)-4-(1-tosyl-1H-indol-3-yl)oxazol-2-yl)acetate (3nn)

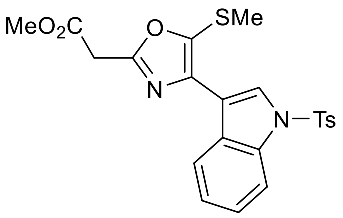

Following **GP3**, using alkynyl thioether **1n** (34.2 mg, 0.1 mmol), aminide **2n** (58.3 mg, 0.3 mmol) and gold catalyst (4.0 mg, 0.01 mmol) for 8 h. A pre-chromatographic filtration through a short pad of silica, eluting with EtOAc was used. Purification by flash column chromatography (30% EtOAc in hexane) afforded oxazole **3nn** as a colourless oil (34.7 mg, 76%); IR (neat): ν = 2981, 1693, 1595, 1511, 1367, 1166, 1136; ^{1}H NMR (300 MHz, $CDCl_3$): δ = 8.27-8.24 (m, 1H), 8.13 (s, 1H), 8.00 (d, *J* = 7.7 Hz, 1H), 7.79 (d, *J* = 8.4 Hz, 2H), 7.38-7.28 (m, 2H), 7.21 (d, *J* = 8.1 Hz, 2H), 3.92 (s, 2H), 3.77 (s, 3H), 2.45 (s, 3H), 2.33 (s, 3H); ^{13}C NMR (101 MHz, $CDCl_3$): δ = 167.8, 158.9, 145.2, 141.4, 137.2, 135.2, 135.1, 130.0 (2C), 129.3, 127.0 (2C), 125.2, 124.8, 123.8, 122.6, 113.5, 113.3, 52.9, 35.0, 21.7, 18.4; HRMS (ES): *m/z* calculated for $C_{22}H_{21}N_2O_5S_2$: 457.0892, found 457.0896 $[M+H]^+$.

Reactions of Cycloaddition Adducts

General Procedure for nickel catalysed C-S activation (GP4)

MeMgCl (3.0 M solution in THF, 3 equiv.) was added dropwise for 5 min to a stirred mixture of thio-oxazole (1 equiv.) and Ni(dppp)Cl_2 (10 mol%) in THF under an argon atmosphere. The reaction was stirred for 10 min at room temperature and then heated to reflux overnight. The reaction mixture was quenched with water, extracted with diethyl ether, washed with brine, dried over Na_2SO_4, filtered and the solvent was removed under reduced pressure. The resulting residue was purifed by silica gel flash column chromatography affording the methyl substituted oxazoles.

Following **GP4**, using *2,4-diphenyl-5-(phenylthio)oxazole* ***(3da)*** (131.6 mg, 0.4 mmol), Ni(dppp)Cl_2 (21.0 mg, 10 mol%) in THF (4.0 mL) and MeMgCl (0.4 mL, 1.2 mmol). Purification by flash column chromatography (5% then 8% EtOAc in hexane) allowed separation of the regioisomers **5a** (60.0 mg, 64%) and **5a’** (16.0 mg, 17%).

Following **GP4**, using *2,4-diphenyl-5-(methylthio)oxazole* ***(3aa)*** (53.5 mg, 0.2 mmol), Ni(dppp)Cl_2 (10.5 mg, 10 mol%) and MeMgCl (0.2 mL, 0.6 mmol). Purification by flash column chromatography (5% then 8% EtOAc in hexane) allowed separation of the regioisomers **5a** (33.8 mg, 72%) and **5a’** (3.0 mg, 6%).

2,4-Diphenyl-5-methyloxazole 5a (*Major isomer*):

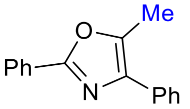

The spectroscopic data match those reported for this regioisomer in the literature.[18] IR (neat): *v* = 3056, 2922, 1597, 1557, 1496, 1447, 1326, 1206, 1081, 1014, 963, 775, 712, 689 cm^{-1}; ^{1}H NMR (300 MHz, $CDCl_3$): δ = 8.09 (d, *J* = 7.9 Hz, 2H), 7.75 (d, *J* = 8.3 Hz, 2H), 7.52-7.41 (m, 5H), 7.33 (t, *J* = 7.2 Hz, 1H), 2.62 (s, 3H); ^{13}C NMR (101 MHz, $CDCl_3$): δ = 159.5, 144.1, 136.1, 132.6, 130.1, 128.8 (2C), 128.7 (2C), 127.8, 127.4, 126.9 (2C), 126.3 (2C), 12.1; HRMS (ES): *m/z* calculated for $C_{16}H_{14}NO$: 236.1075; found 236.1072 $[M+H]^+$.

2,5-Diphenyl-4-methyloxazole 5a′ (*Minor isomer*):

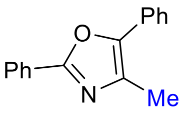

The spectroscopic data match those reported for this regioisomer in the literature.[19] mp. 68-70 °C (Lit.[19b] 69-72 °C); IR (neat): *v* = 3055, 2918, 1593, 1547, 1483, 1442, 1381, 1130, 1097, 1068, 953, 777, 762, 709, 687, 671 cm^{-1}; ^{1}H NMR (300 MHz, $CDCl_3$): δ = 8.10 (d, *J* = 7.9 Hz, 2H), 7.69 (d, *J* = 7.3 Hz, 2H), 7.53-7.41 (m, 5H), 7.34 (t, *J* = 7.4 Hz, 1H), 2.51 (s, 3H); ^{13}C NMR (101 MHz, $CDCl_3$): δ = 159.5, 145.6, 133.5, 130.3, 129.3, 128.92 (2C), 128.90 (2C), 127.7, 127.6, 126.3 (2C), 125.5 (2C), 13.7; HRMS (ES): *m/z* calculated for $C_{16}H_{14}NO$: 236.1075; found 236.1077 $[M+H]^+$.

4-(4-Methoxyphenyl)-5-methyl-2-phenyloxazole (5b):

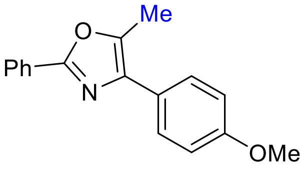

Following **GP4**, using *5-(ethylthio)-4-(4-methoxyphenyl)-2-phenyloxazole **3ga*** (56.2 mg, 0.2 mmol), Ni(dppp)Cl_2 (10.5 mg, 10 mol%) and 3.0 M solution of MeMgBr (0.2 mL, 0.6 mmol) was refluxed for 15 h. Purification by flash chromatography (3-5% EtOAc in hexane) afforded oxazole **5b** as a colourless solid (38.0 mg, 72%); mp. 74-76 °C; IR (neat): *v* = 3054, 2956, 2834, 1603, 1541, 1508, 1440, 1300, 1291, 1245, 1166, 1108, 1022, 958, 832, 812, 771, 748, 683 cm^{-1}; ^{1}H NMR (300 MHz, $CDCl_3$): δ = 8.07 (d, *J* = 7.9 Hz, 2H), 7.67 (d, *J* = 8.8 Hz, 2H), 7.50-7.38 (m, 3H), 6.99 (d, *J* = 8.8 Hz, 2H), 3.85 (s, 3H), 2.59 (s, 3H); ^{13}C NMR (101 MHz, $CDCl_3$): δ = 159.3, 159.0, 143.1, 135.9, 130.0, 128.8 (2C), 128.2 (2C), 127.9, 126.2 (2C), 125.2, 114.2 (2C), 55.4, 12.0; HRMS (ES): *m/z* calculated for $C_{17}H_{16}NO_2$: 266.1181; found 266.1173 $[M+H]^+$.

General procedure for Boc-deprotection of thio-oxazoles 3ki and 3kj (GP5):[16]

The Boc-pyrrolidine derived oxazoles **3ki** and **3kj** (0.15 mmol) were treated with TFA (0.3 mL) in CH_2Cl_2 (3 mL) at 0 °C. After stirring for 2 hrs at room temperature, the reaction mixture was slowly poured into satd. $NaHCO_3$(aq) solution. The organic layer was extracted with CH_2Cl_2 (3 ×10 mL) and the combined organic layers were washed with brine, dried over Na_2SO_4, filtered and the solvent was removed *in vacuo*. The resulting residue was purified by silica gel flash chromatography affording the pyrrolidine-derived oxazoles (**6a** and **6b**).

(*S*)-4-(3,4-Dimethoxyphenyl)-5-(ethylthio)-2-(pyrrolidin-2-yl)oxazole (6a):

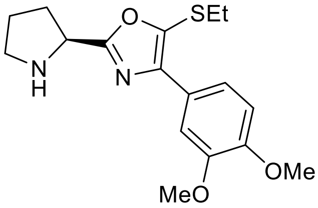

Following **GP5**, using the Boc-pyrrolidine derived oxazole **3ki** (65.1 mg, 0.15 mmol). Purification by flash column chromatography using 10% MeOH in EtOAc afforded pyrrolidine derived oxazole **6a** as a pale yellow liquid (41.2 mg, 82%); $[\alpha]_D^{21}$ = -20.8° (*c* = 0.5, $CHCl_3$); IR (neat): *v* = 3328, 2960, 2930, 1589, 1563, 1503, 1462, 1252, 1223, 1175, 1126, 1025, 911, 860, 764, 729 cm^{-1}; ^{1}H NMR (300 MHz, $CDCl_3$): δ = 7.74-7.61 (m, 2H), 6.89 (d, *J* = 8.3 Hz, 1H), 4.34 (t, *J* = 6.0 Hz, 1H), 3.94 (s, 3H), 3.90 (s, 3H), 3.25-3.12 (m, 1H), 3.06-2.96 (m, 1H), 2.83 (q, *J* = 7.4 Hz, 2H), 2.37 (br s, 1H), 2.24-2.05 (m, 2H), 1.99-1.80 (m, 2H), 1.25 (t, *J* = 7.4 Hz, 3H); ^{13}C NMR (101 MHz, $CDCl_3$): δ = 167.5, 149.1, 148.8, 142.5, 137.8, 124.1, 120.0, 111.0, 110.3, 56.02, 55.97, 55.93, 47.0, 31.0, 30.3, 25.5, 15.3; HRMS (ES): *m/z* calculated for $C_{17}H_{23}N_2O_3S$: 335.1429; found 335.1425 $[M+H]^+$.

2-[(*2S*,*4R*)-4-(Benzyloxy)pyrrolidin-2-yl]-4-(3,4-dimethoxyphenyl)-5-(ethylthio)oxazole (6b):

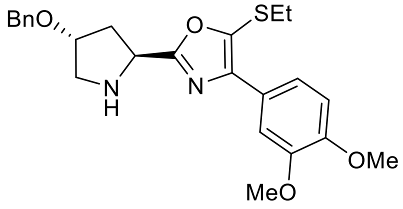

Following **GP5**, using the Boc-pyrrolidine derived oxazole **3kj** (81.0 mg, 0.15 mmol). Purification by flash column chromatography using 3% MeOH in EtOAc afforded pyrrolidine derived oxazole **6b** as a colourless liquid (48.1 mg, 73%); $[\alpha]_D^{21}$ = –28.0° (c = 1.0, $CHCl_3$); IR (neat): v = 3339, 2928, 1590, 1566, 1504, 1453, 1253, 1223, 1176, 1077, 1025, 910, 861, 765, 729, 697 cm^{-1}; ^{1}H NMR (300 MHz, $CDCl_3$): δ = 7.74-7.62 (m, 2H), 7.38-7.33 (m, 3H), 7.32-7.27 (m, 2H), 6.90 (d, J = 8.3 Hz, 1H), 4.59 (t, J = 7.7 Hz, 1H), 4.53 (ABq, J = 11.9 Hz, 2H), 4.32-4.23 (m, 1H), 3.94 (s, 3H), 3.91 (s, 3H), 3.30-3.14 (m, 2H), 2.84 (q, J = 7.4 Hz, 2H), 2.48-2.36 (m, 2H), 2.35-2.26 (m, 1H), 1.25 (t, J = 7.4 Hz, 3H); ^{13}C NMR (101 MHz, $CDCl_3$): δ = 167.1, 149.1, 148.8, 142.6, 138.3, 137.9, 128.6 (2C), 127.8, 127.7 (2C), 124.1, 120.0, 110.9, 110.3, 79.8, 71.1, 56.02, 55.97, 54.8, 52.8, 37.5, 30.3, 15.3; HRMS (ES): m/z calculated for $C_{24}H_{29}N_2O_4S$: 441.1848; found 441.1853 $[M+H]^+$.

4-(Methylthio)-2,5-diphenyloxazole (3aa')

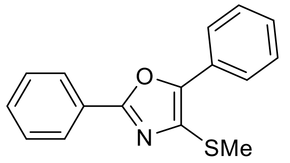

Alkynyl thioether **1a** (54.8 mg, 0.37 mmol) was added to dioxazole **7** (81.5 mg, 0.46 mmol, 1.2 equiv.). 1,2-Dichlorobenzene (4.0 mL) was added followed by Tf_2NH (5.9 mg, 0.02 mmol) and the reaction was stirred at 125 °C for 3.5 h and then allowed to cool to r.t. The crude reaction mixture was purified by flash column chromatography (40% hexane in toluene: complete separation from the dioxazole was not possible) to give oxazole **3aa'** as a pale yellow oil (13.5 mg, 14%). IR (neat): ν = 3065, 2923, 1696, 1600, 1558, 1485, 1449, 774; ^{1}H NMR (300 MHz, $CDCl_3$): δ = 8.14-8.10 (m, 2H), 7.99-7.95 (m, 2H), 7.51-7.45 (m, 5H), 7.34 (tt, J = 7.4 Hz, 1.3 Hz, 1H), 2.63 (s, 3H); ^{13}C NMR (101 MHz, $CDCl_3$): δ = 160.0, 147.3, 132.0, 130.7, 128.9 (2C), 128.8 (2C), 128.4, 128.2, 127.2, 126.6 (2C), 125.5 (2C), 16.8; HRMS (EI): m/z calculated for $C_{16}H_{13}NOS$: 267.0718, found 267.0720 $[M]^+$.

Stability Studies of Chiral Pyrrolidine Derived Aminides

To explore the possibility of degradation and/or epimerization/racemisation of the chiral pyrrolidine derived aminides (**2i** and **2j**) they were independently heated to 125 °C in 1,2-DCB for 3 h in the presence of $PicAuCl_2$. For **2j** there was no indication of epimerisation from analysis of the ^{1}H NMR. Both aminides were reisolated in high yield (**2i**, 96%; **2j**, 92%) returning the same optical rotations.

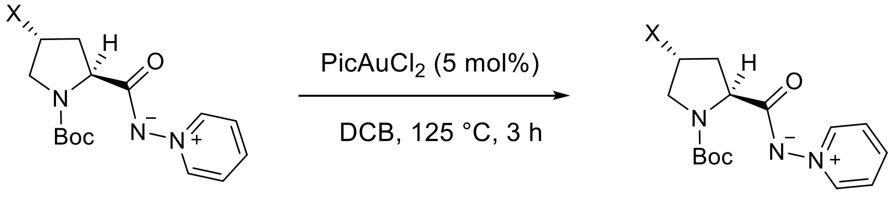

2j (X = OBn): **$[\alpha]_D^{21}$ = -83.2°** X = OBn: 92% (Rec.) **$[\alpha]_D^{20}$ = -82.4°**
2i (X = H): **$[\alpha]_D^{21}$ = -137.6°** X = H: 96% (Rec.) **$[\alpha]_D^{20}$ = -134.8°**

Single Crystal X-Ray Data Analysis

General: Suitable crystals were selected and a dataset was measured on a Bruker SMART 6000 diffractometer ($\lambda_{Cu-K\alpha}$ = 1.5418 Å or $\lambda_{Mo-K\alpha}$ = 0.71073 Å). The data collection was driven by SMART and processed by SAINTPLUS and an absorption correction was applied using SADABS. The structures were solved using ShelXS-9742 and refined by a full-matrix least-squares procedure on F2 in ShelXL-

97. All non-hydrogen atoms were refined with anisotropic displacement parameters. The N-bound hydrogen atom was located in the electron density and the position refined freely. All remaining hydrogen atoms were added at calculated positions and refined by use of a riding model and the isotropic displacement parameters for all hydrogen atoms are based on the equivalent isotropic displacement parameter (Ueq) of the parent atom. Figures were produced using OLEX2.

2,4-Diphenyl-5-(methylthio)oxazole (3aa-CCDC-1537012):
$C_{16}H_{13}NOS$, *M*r = 267.33, crystal dimensions: 0.20 x 0.18 x 0.11 mm, monoclinic, space group: *P2₁/n*, *a* =17.3013(3), *b* = 8.55085(13), *c* = 18.6856(3)Å, α = 90°, β = 107.3895 (19)°, γ = 90°, *V* =2638.02(8)Å^3, *Z* = 8, ρ_{calcd}= 1.346g/cm^3, μ = 2.091 mm^{-1}, $F_{000} = 1120.0$, $\lambda_{Cu-K\alpha}$ = 1.54184 Å, *T* = 99.97(14) K, 2θ_{max} =148.824°, 10334 reflections measured, 5246 independent reflections (R_{int} = 0.0263), R_1 = 0.0349 (observed reflections), *wR*= 0.0961 (all data), largest diff. peak and hole: 0.40 and -0.26 e.Å^{-3}.

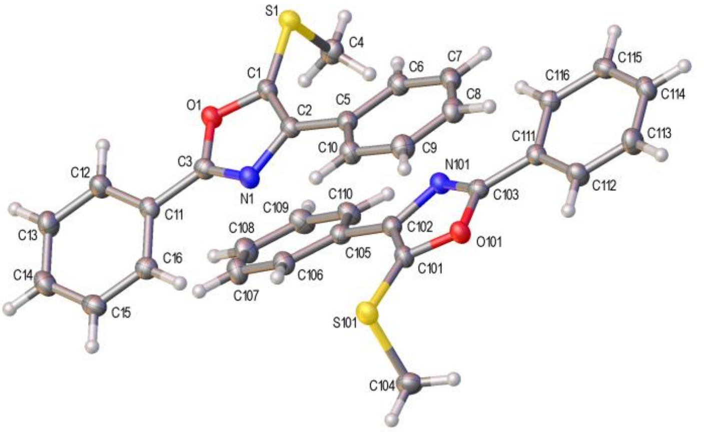

Figure 1: Crystal structure with ellipsoids drawn at the 50% probability level (The structure contains crystallographically two independent molecules).

5-(Ethylthio)-4-(4-methoxyphenyl)-2-phenyloxazole (3ga-CCDC-1537013):
$C_{18}H_{17}NO_2S$, *M*r = 311.38, crystal dimensions: 0.38 x 0.13 x 0.054 mm, triclinic, space group: *P-1*, *a* =8.2479(4), *b* = 10.0753(4), *c* = 10.9958(5) Å, α = 114.193(4)°, β = 109.745(4)°, γ = 91.909(4)°, *V* =768.63(6) Å^3, *Z* = 2, ρ_{calcd}= 1.345g/cm^3, μ = 0.217 mm^{-1}, $F_{000} = 328.0$, $\lambda_{Mo-K\alpha}$ = 0.71073 Å, *T* = 100(10) K, 2θ_{max} =52.75°, 5757 reflections measured, 3144 independent reflections (R_{int} = 0.0233), R_1 = 0.0435 (observed reflections), *wR*= 0.1065 (all data), largest diff. peak and hole: 0.29 and -0.29 e.Å^{-3}.

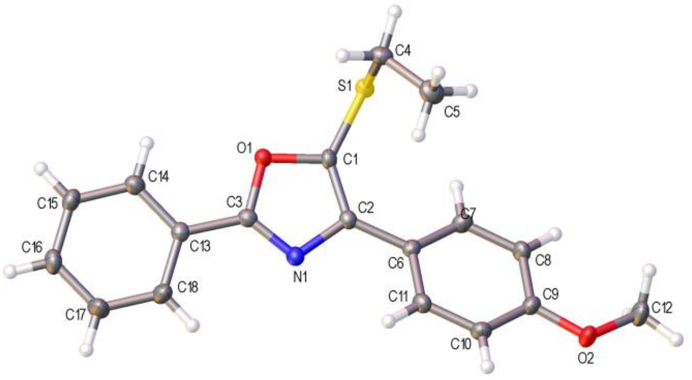

Figure 2: Crystal structure with ellipsoids drawn at the 50% probability level.

5-(Methylthio)-2-(naphthalen-2-yl)-4-(2,4,6-trimethylphenyl)oxazole (3lc-CCDC-1537014): $C_{23}H_{21}NOS$, *M*r = 359.47, crystal dimensions: 0.33 x 0.19 x 0.09 mm, monoclinic, space group: *P2/n*, *a* =14.2578(3), *b* = 8.43491(17), *c* = 15.6214(4) Å, *α* = 90°, *β* = 102.717(2)°, *γ* = 90°, *V* =1832.59(7) Å^3, *Z* = 4, ρ_{calcd}= 1.303g/cm^3, μ = 0.188 mm^{-1}, F_{000} = 760.0, $\lambda_{Mo-K\alpha}$ = 0.71073 Å, *T* = 100(10) K, $2\theta_{max}$ =52.75°, 18953 reflections measured, 3753 independent reflections (R_{int} = 0.0388), R_1 = 0.0386 (observed reflections), *wR*= 0.0996 (all data), largest diff. peak and hole: 0.34 and -0.33 e.Å^{-3}.

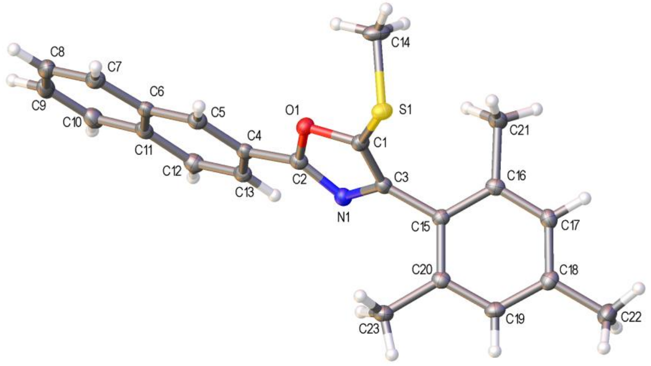

Figure 3: Crystal structure with ellipsoids drawn at the 50% probability level.

References

[1] H. E. Gottlieb, V. Kotlyar and A. Nudelman, *J. Org. Chem.,* 1997, **62**, 7512-7515.
[2] W. Zheng, F. Zheng, Y. Hong and L. Hu, *Heteroatom Chem.,* 2012**, 23***,* 105-110.
[3] E. A. Zaburdaeva and V. A. Dodonov, *Russ. Chem. Bull., Int. Ed.*, 2011, **60**, 185-187.
[4] Y.-L. Zhao, L. Liu, W. Zhang, C.-H. Sue, Q. Li, O. S. Miljanić, O. M. Yaghi and J. F. Stoddart, *Chem. – Eur. J.*, 2009, **15**, 13356-13380.
[5] N. Zhou, L. Wang, D. Thompson and Y. Zhao, *Tetrahedron*, 2011, **67**, 125-143.
[6] Z. Fang, Y. Song, T. Sarkar, E. Hamel, W. E. Fogler, G. E. Agoston, P. E. Fanwick and M. Cushman, *J. Org. Chem*., 2008, **73**, 4241-4244.
[7] L. I. Dixon, M. A. Carroll, T. J. Gregson, G. J. Ellames, R. W. Harrington and W. Clegg, *Eur. J. Org. Chem*., 2013, 2334-2345.
[8] Y. Li, H. Zou, J. Gong, J. Xiang, T. Luo, J. Quan, G. Wang and Z. Yang, *Org. Lett.*, 2007, **9**, 4057-4060.
[9] R. Wortmann, C. Glania, P. Krämer, R. Matschiner, J. Jens Wolff, S. Kraft, B. Treptow, E. Barbu, D. Längle and G. Görlitz, *Chem. – Eur. J.*, 1997, **3**, 1765-1773.
[10] K. Fujiki, N. Tanifuji, Y. Sasaki and T. Yokoyama, *Synthesis*, 2002, 343-348.
[11] V. Girijavallabhan, C. Alvarez and F. G. Njoroge, *J. Org. Chem.,* 2011, **76**, 6442-6446.
[12] (a) H. W. Pinnick, M. A. Reynolds, R. T. McDonald Jr. and W. D. Brewster, *J. Org. Chem*., 1980, **45**, 931-932; (b) M. S. Chen, N Pabagaran, N. A. Labenz and M. C. White, *J. Am. Chem. Soc*., 2005, **127**, 6970-6971.
[13] E. Chatzopoulou and P. W. Davies, *Chem. Commun*., 2013, **49**, 8617-8619.
[14] C. Legault and A. B. Charette, *J. Org. Chem.,* 2003, **68**, 7119-7122.
[15] P. W. Davies, A. Cremonesi and L. Dumitrescu, *Angew. Chem., Int. Ed.,* 2011, **50**, 8931-8935.
[16] A. D. Gillie, R. J. Reddy and P. W. Davies, *Adv. Synth. Cat.,* 2016, **358**, 226-239.
[17] V. Bizet and C. Bolm, *Eur. J. Org. Chem*., 2015, 2854-2860.
[18] For 2,4-diphenyl-5-methyloxazole (**5a**), see: F.-J. Zheng, H.-X. Lin and Z.-P. Zhan, *J. Org. Chem*., 2009, **74**, 3148-3151.
[19] For 2,5-diphenyl-4-methyloxazole (**5a'**), see: (a) M. Keni and J. J. Tepe*, J. Org. Chem*., 2005, **70**, 4211-4213; (b) W.-C. Gao, R.-L. Wang and C. Zhang, *Org. Biomol. Chem.,* 2013*,* **11***,* 7123-7128.

^{1}H NMR and ^{13}C NMR Spectra of New Compounds

Methyl 4-[(methylthio)ethynyl]benzoate (1f) in $CDCl_3$ ^{1}H-NMR and ^{13}C-NMR (UDEFT)

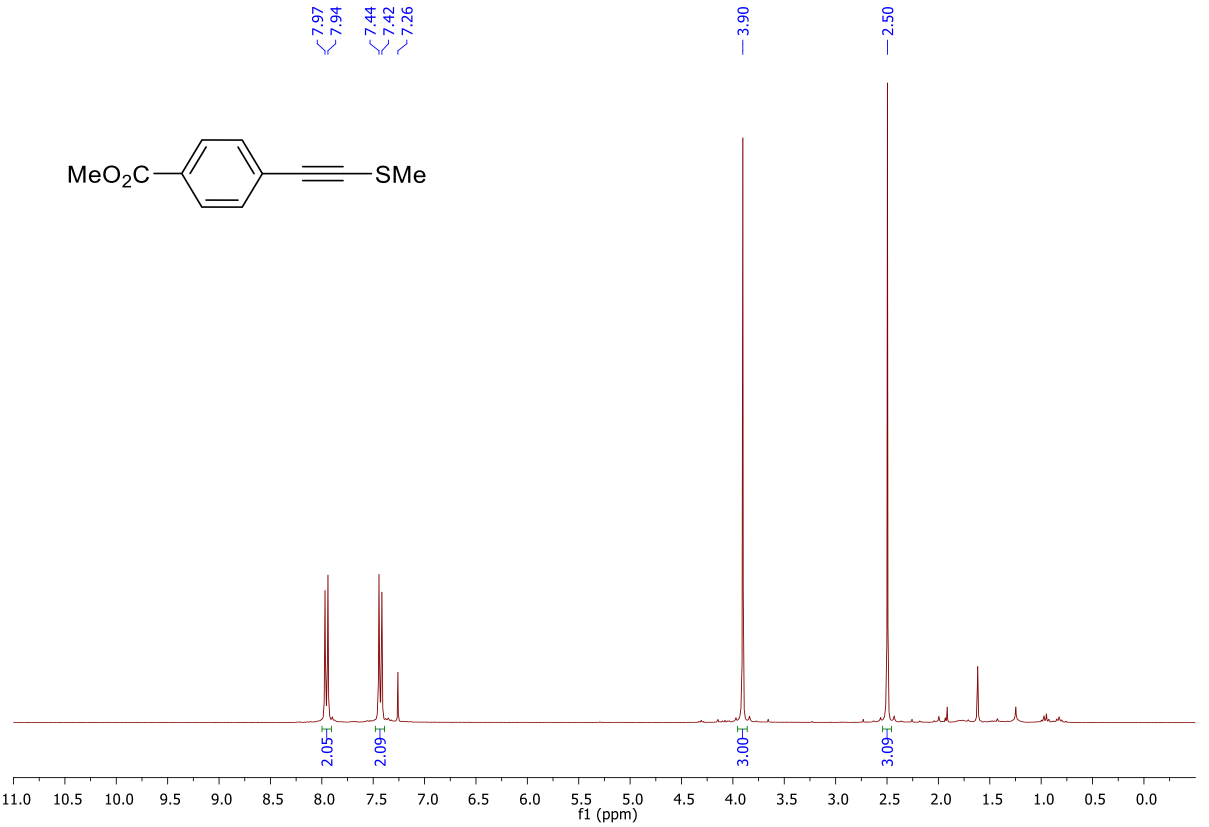

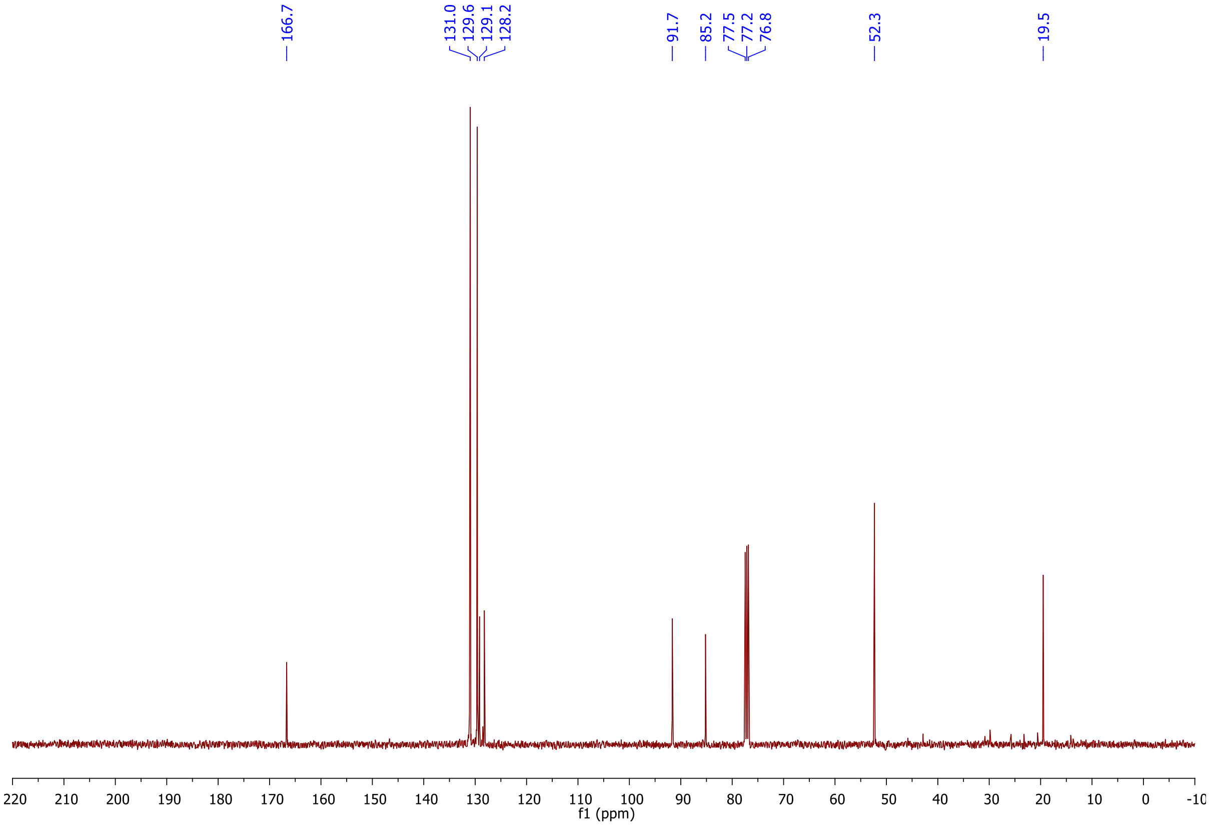

Ethyl(4-methoxyphenyl)ethynylsulfide (1g) in $CDCl_3$ ^{1}H-NMR and ^{13}C-NMR ((Pendant)

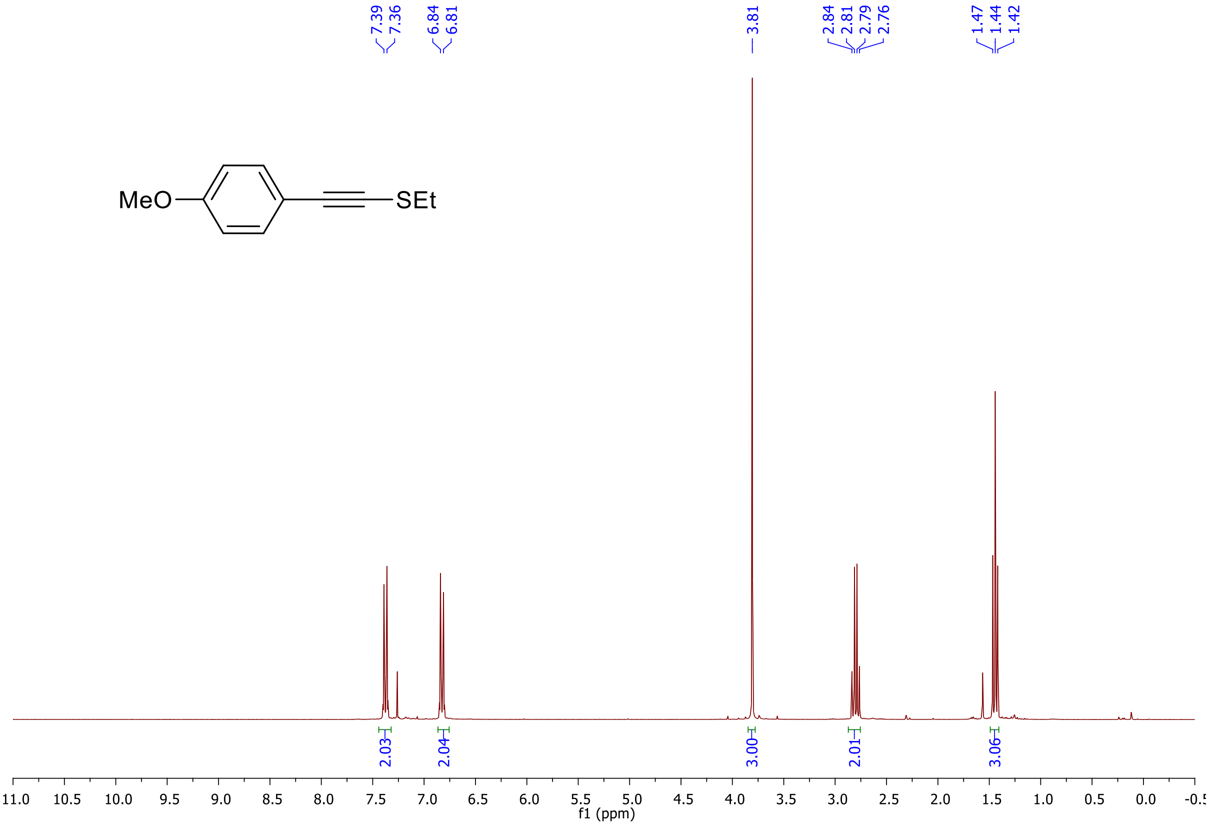

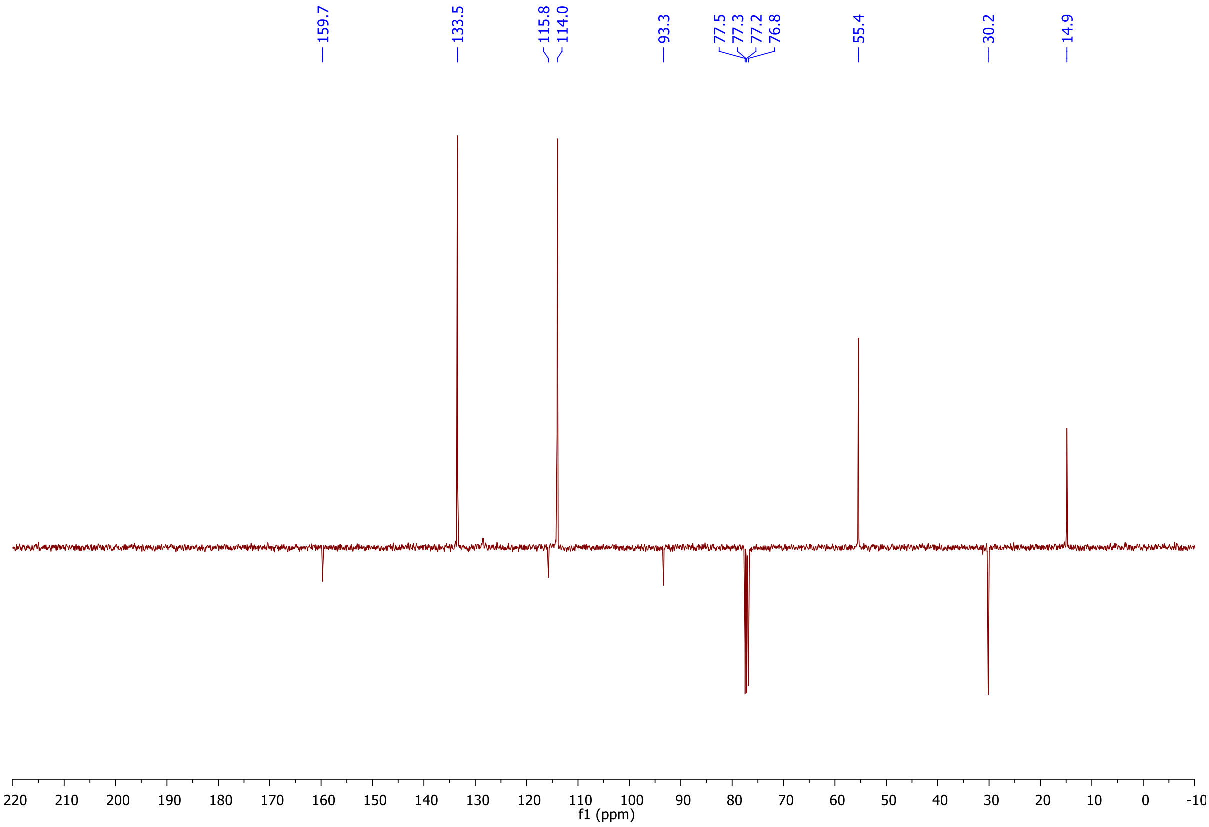

4-(Methoxyphenyl)ethynylphenylsulfide (1h) in $CDCl_3$ ^1H-NMR and ^{13}C-NMR (Pendant)

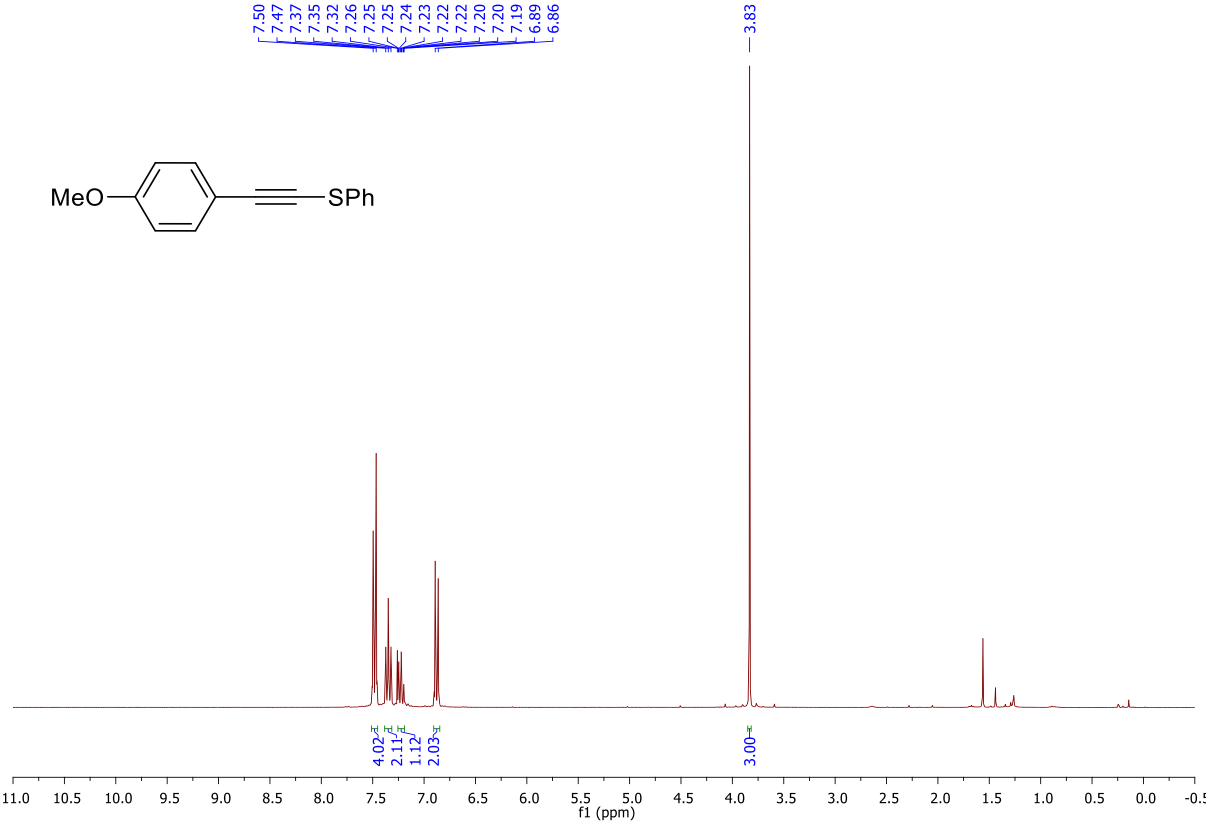

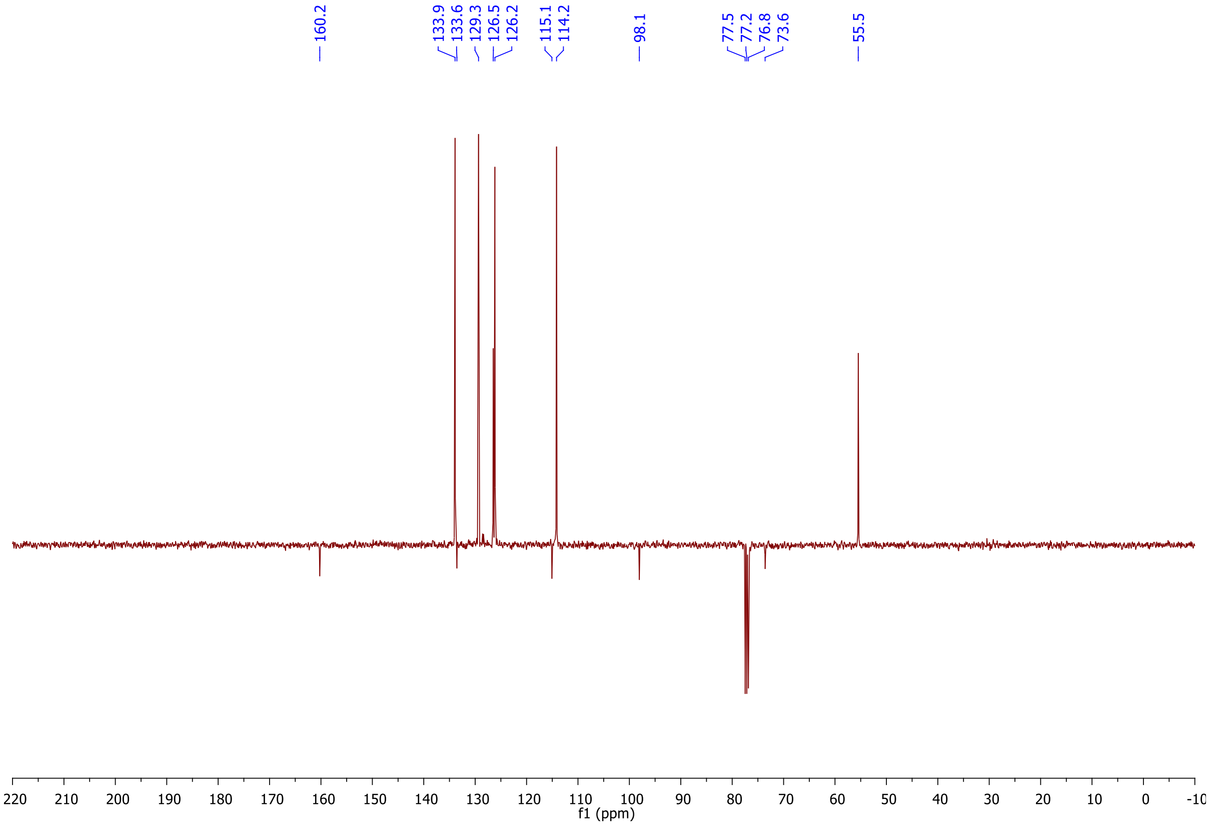

((4-Methoxyphenyl)ethynyl)(methyl)sulfane (1i) in $CDCl_3$ ^{1}H-NMR and ^{13}C-NMR (JMOD)

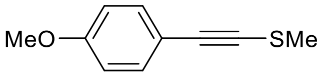

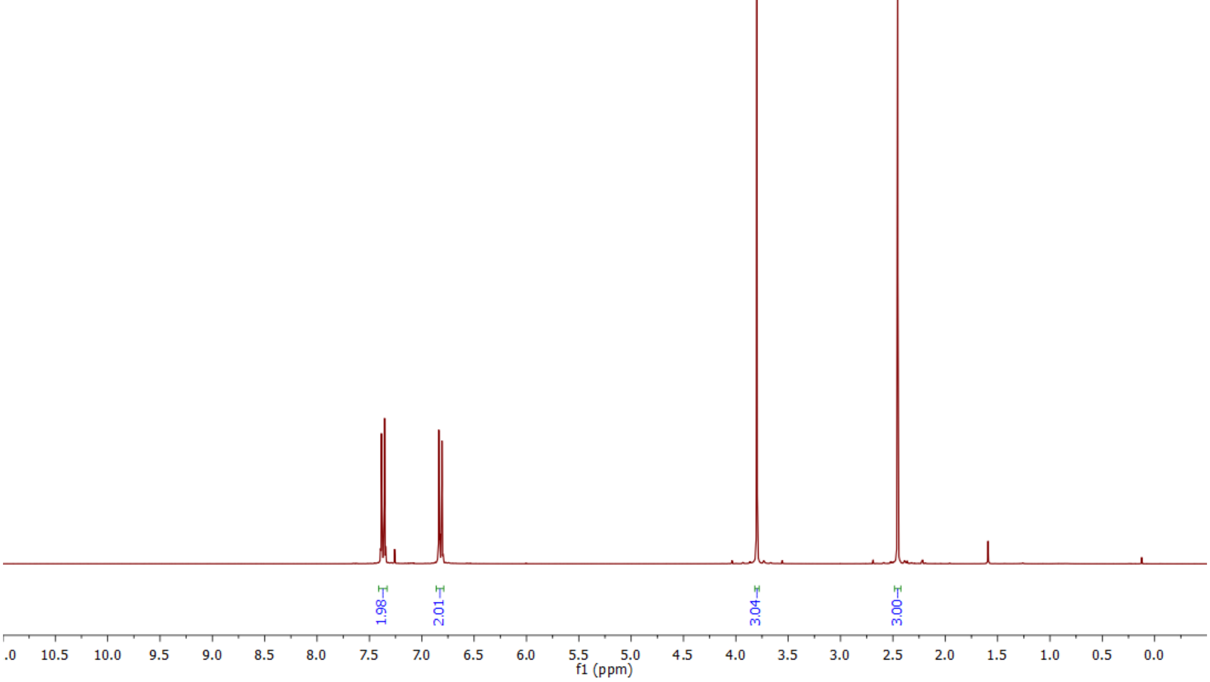

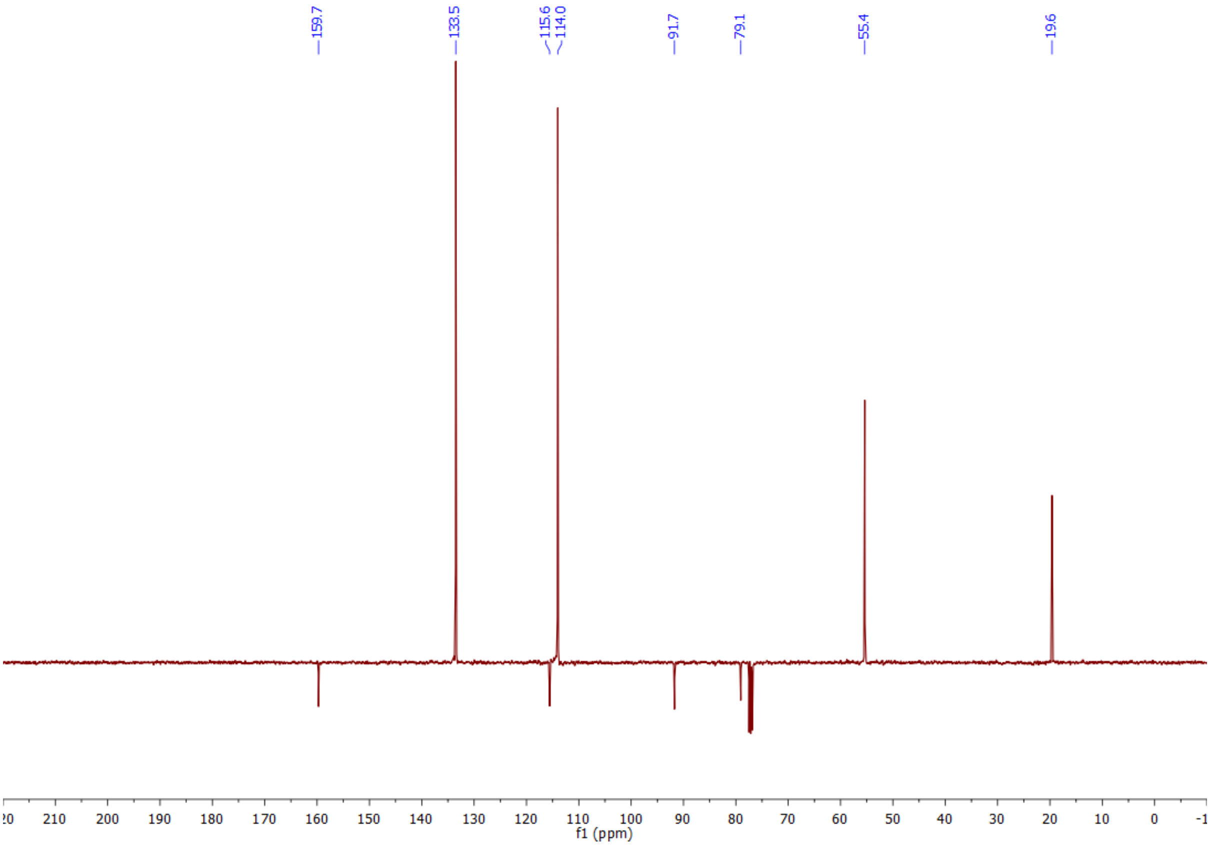

(3,4-Dimethoxyphenyl)ethynylethylsulfide (1k) in $CDCl_3$ ^1H-NMR and ^{13}C-NMR (UDEFT)

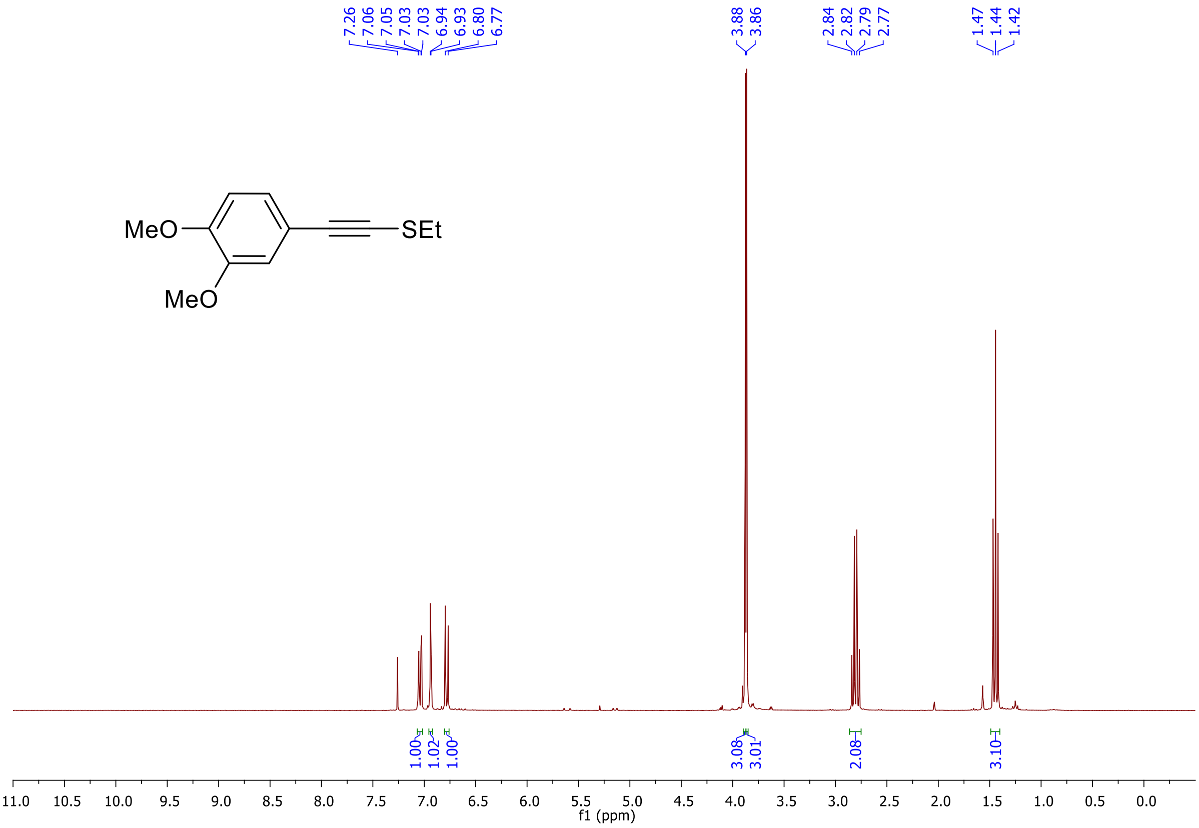

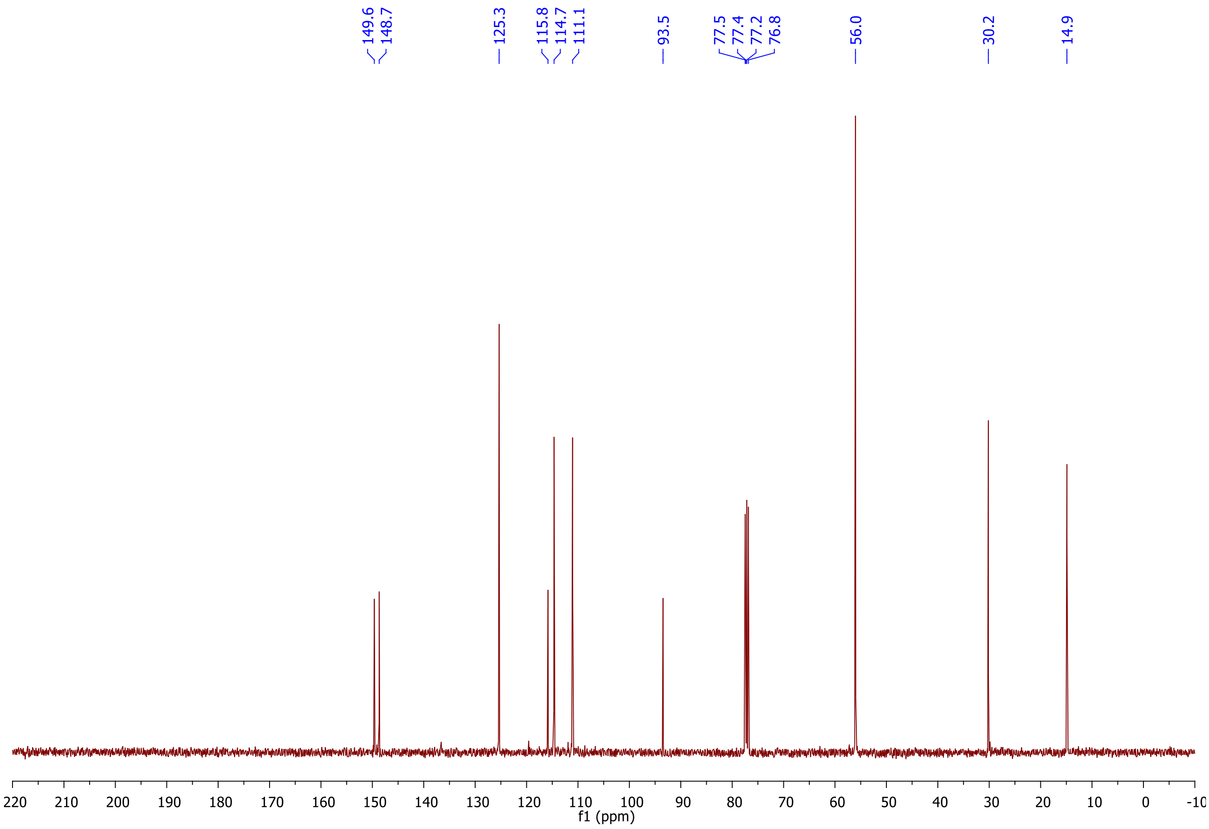

Mesitylethynyl(methyl)sulfide (1l) in $CDCl_3$ ^{1}H-NMR and ^{13}C-NMR (Pendant)

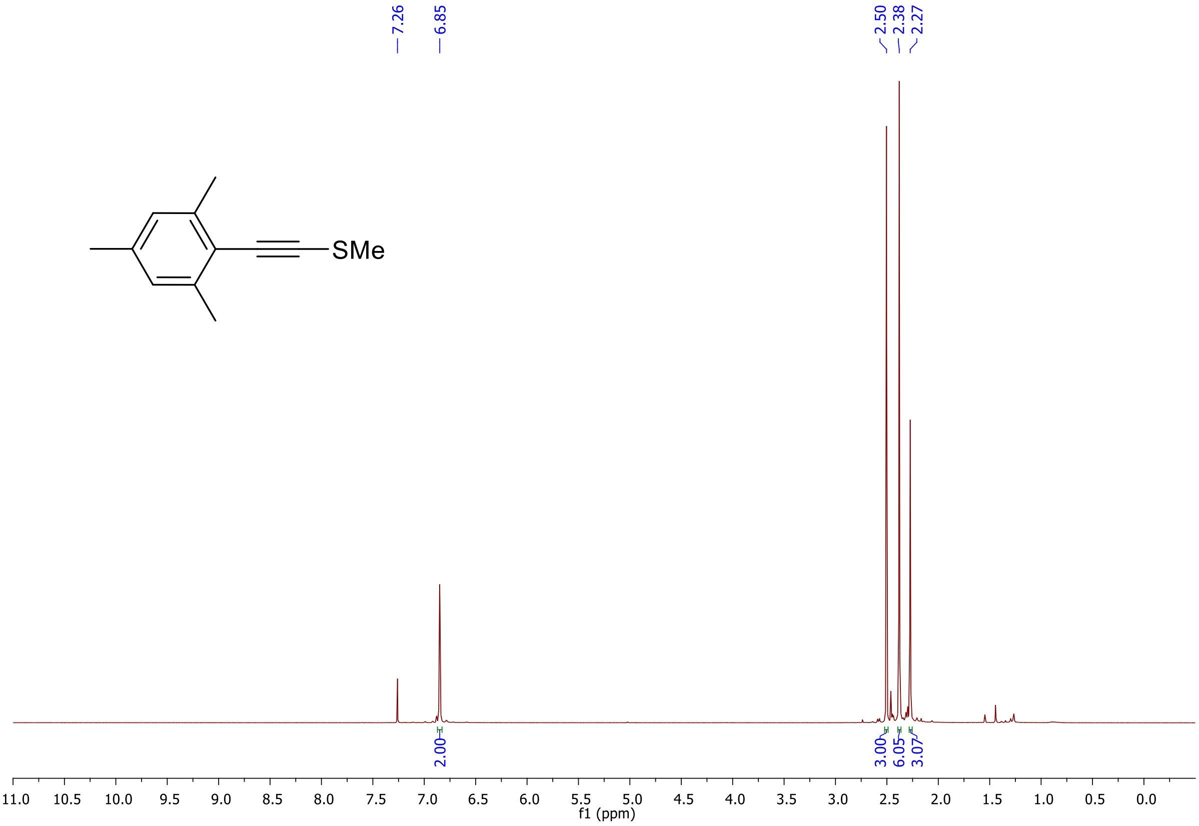

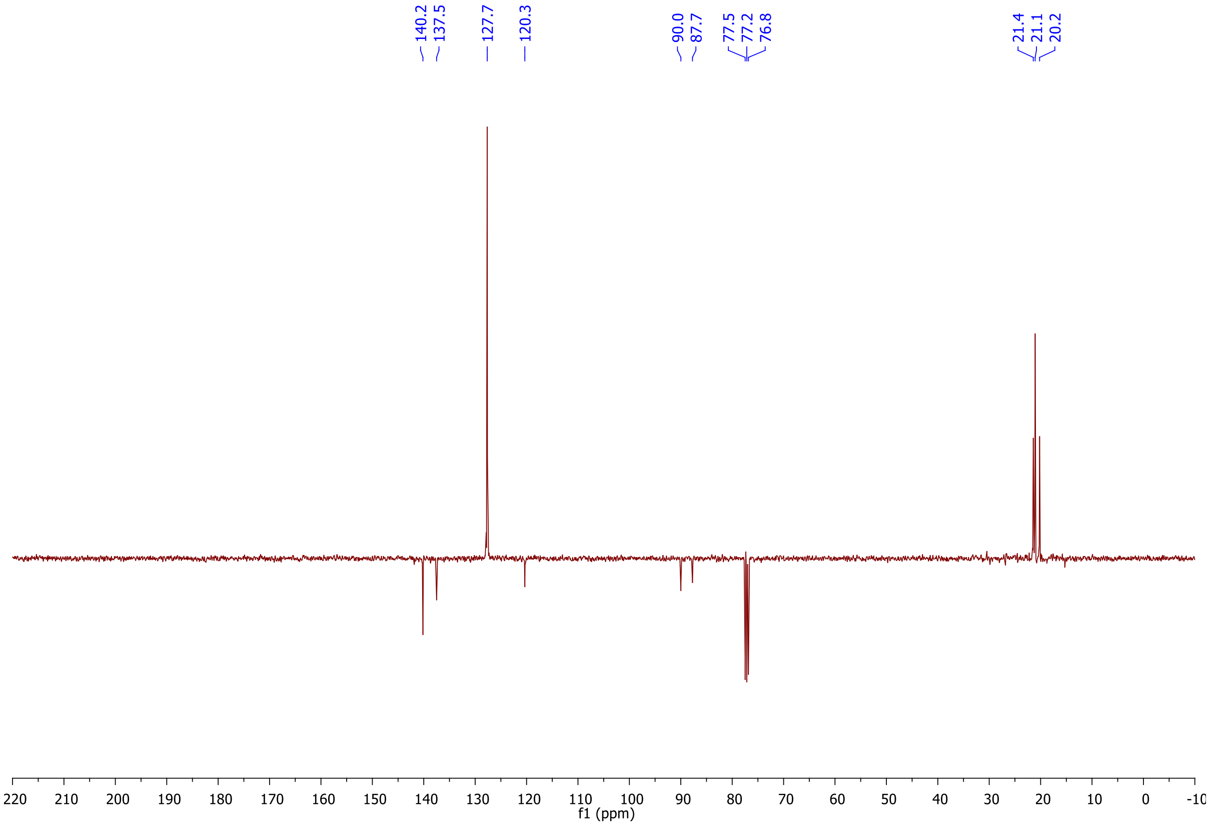

***N,N*-Diethyl-4-((methylthio)ethynyl)aniline (1m)** in $CDCl_3$ ^{1}H-NMR and ^{13}C-NMR (JMOD)

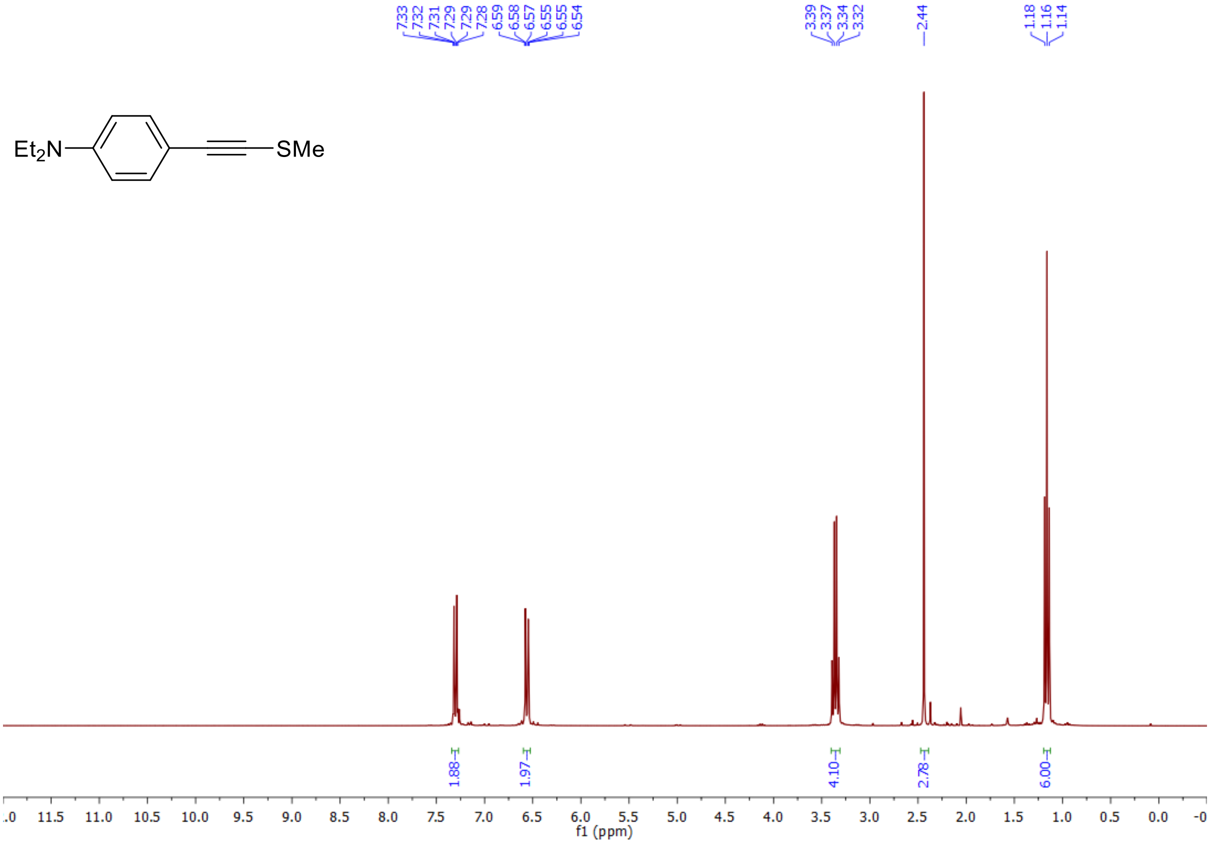

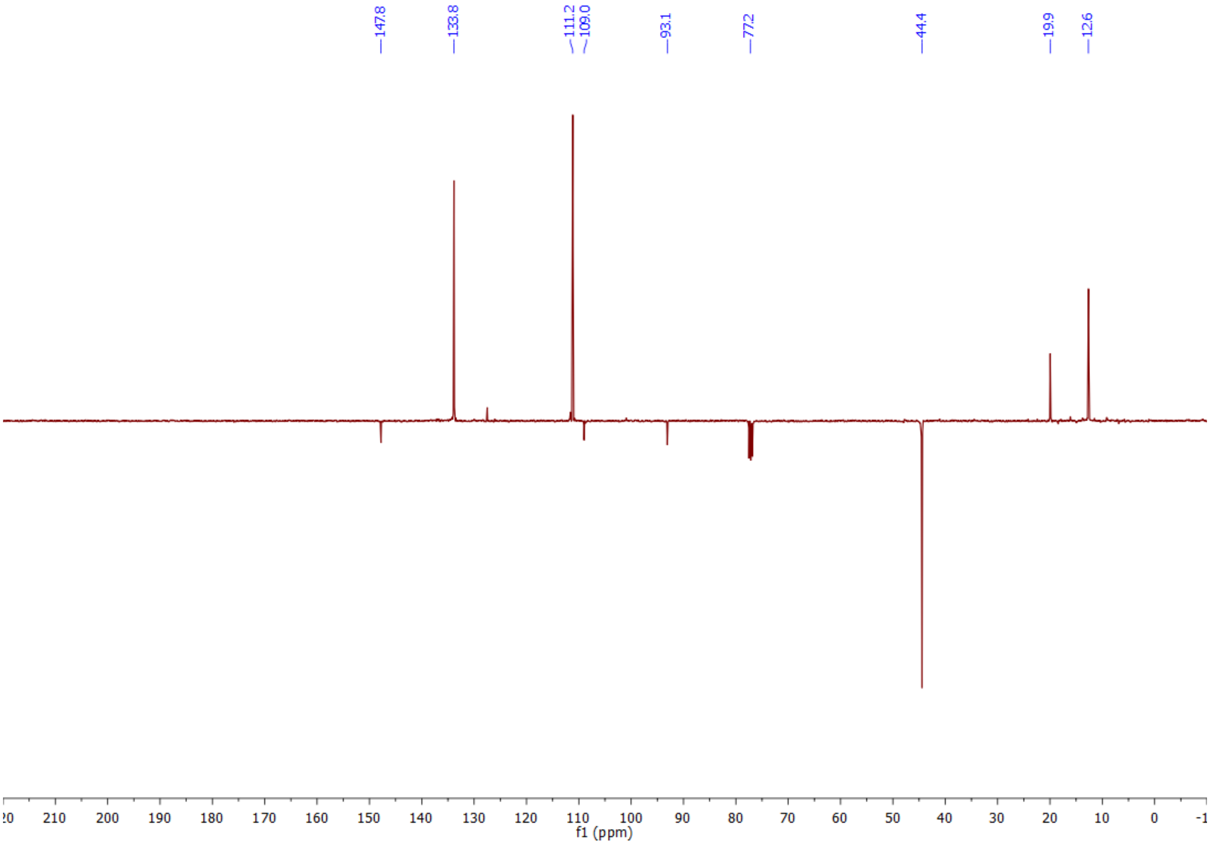

3-((Methylthio)ethynyl)-1-tosyl-1H-indole (1n) in $CDCl_3$ ^{1}H-NMR and ^{13}C-NMR (JMOD)

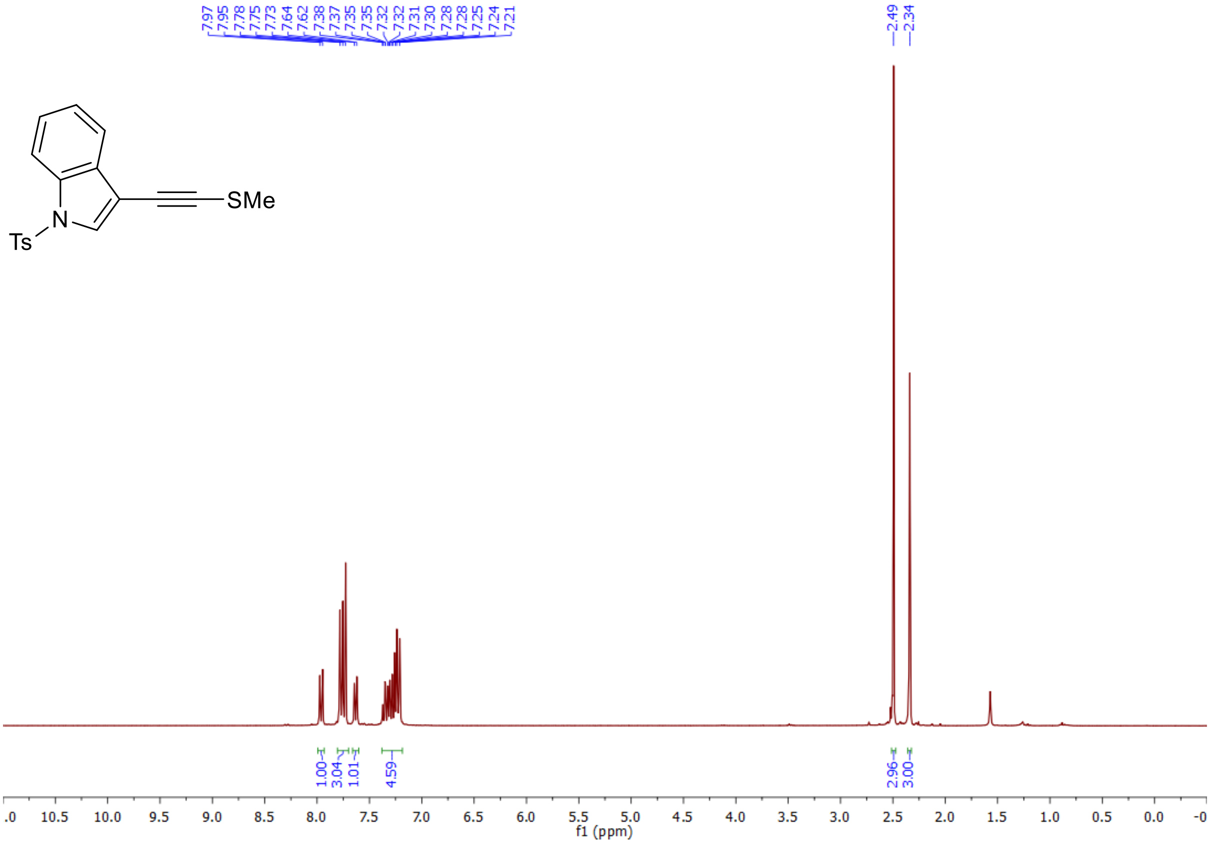

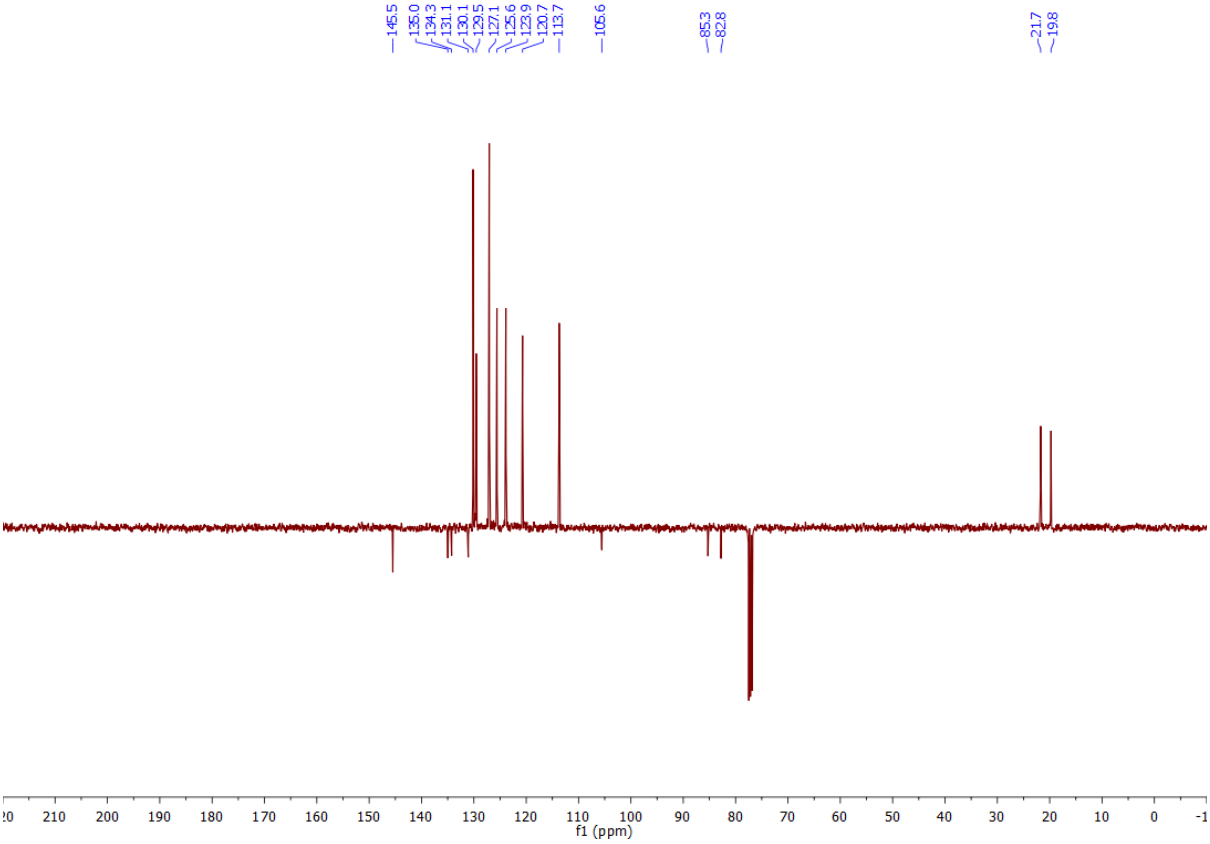

1-Methoxy-4-(prop-1-yn-1-yl)benzene (4b) in $CDCl_3$ ^1H-NMR and ^{13}C-NMR (UDEFT)

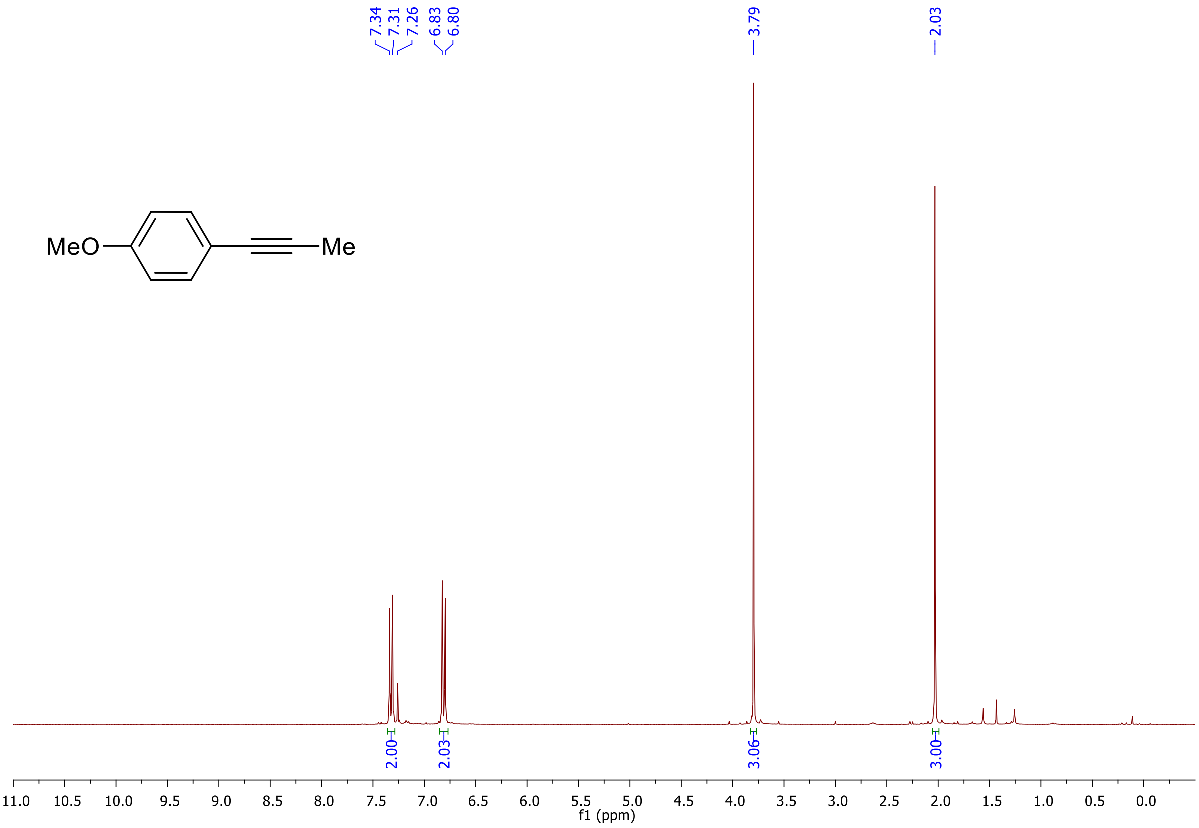

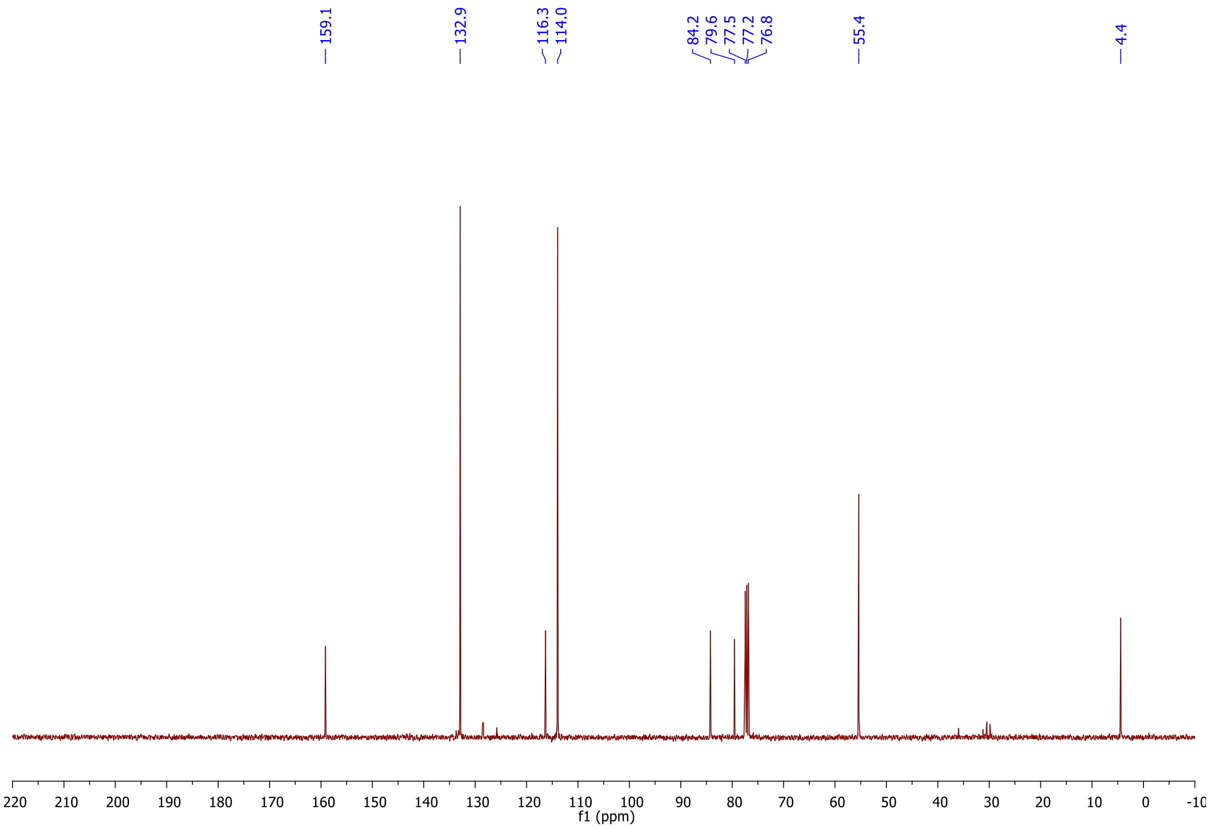

(2-Naphthoyl)(pyridin-1-ium-1-yl)amide (2c) in $CDCl_3$ ^{1}H-NMR and ^{13}C-NMR (UDEFT)

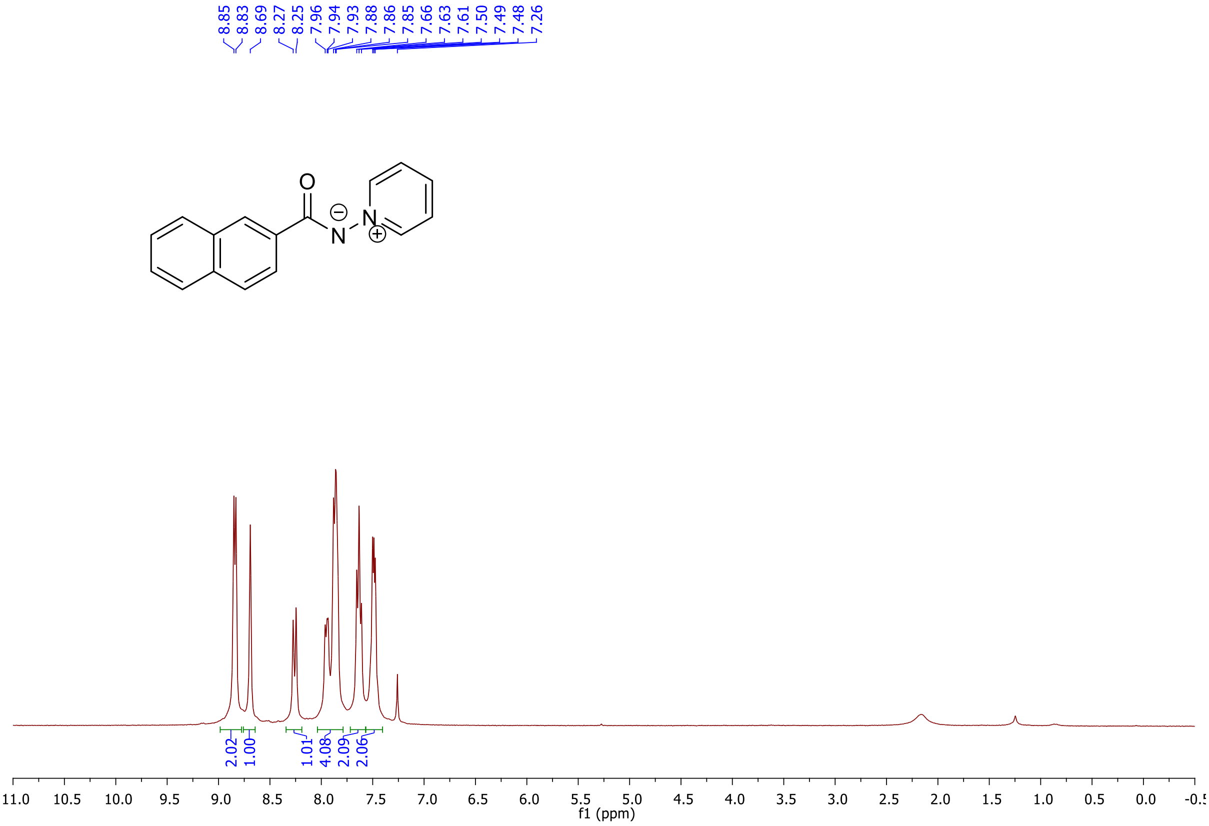

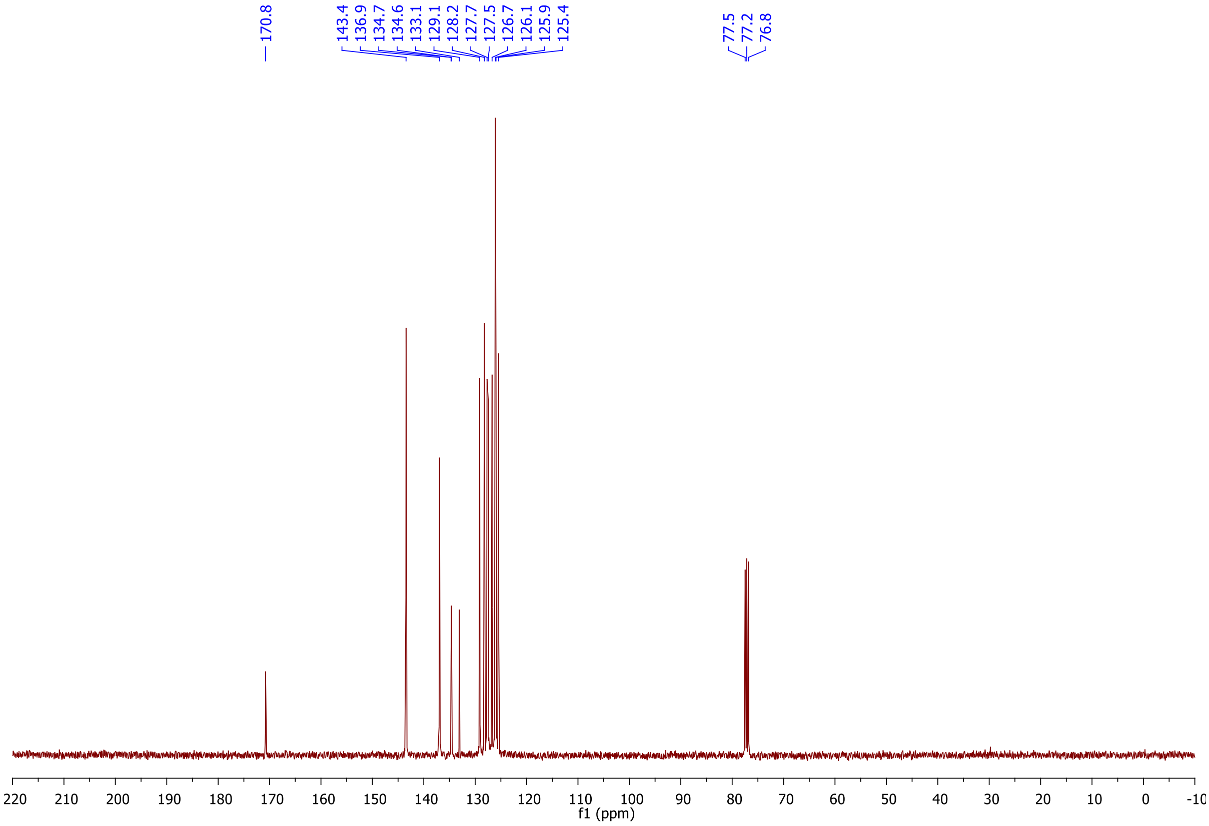

(1-Naphthoyl)(pyridin-1-ium-1-yl)amide (2d) in $CDCl_3$ ^1H-NMR and ^{13}C-NMR (UDEFT)

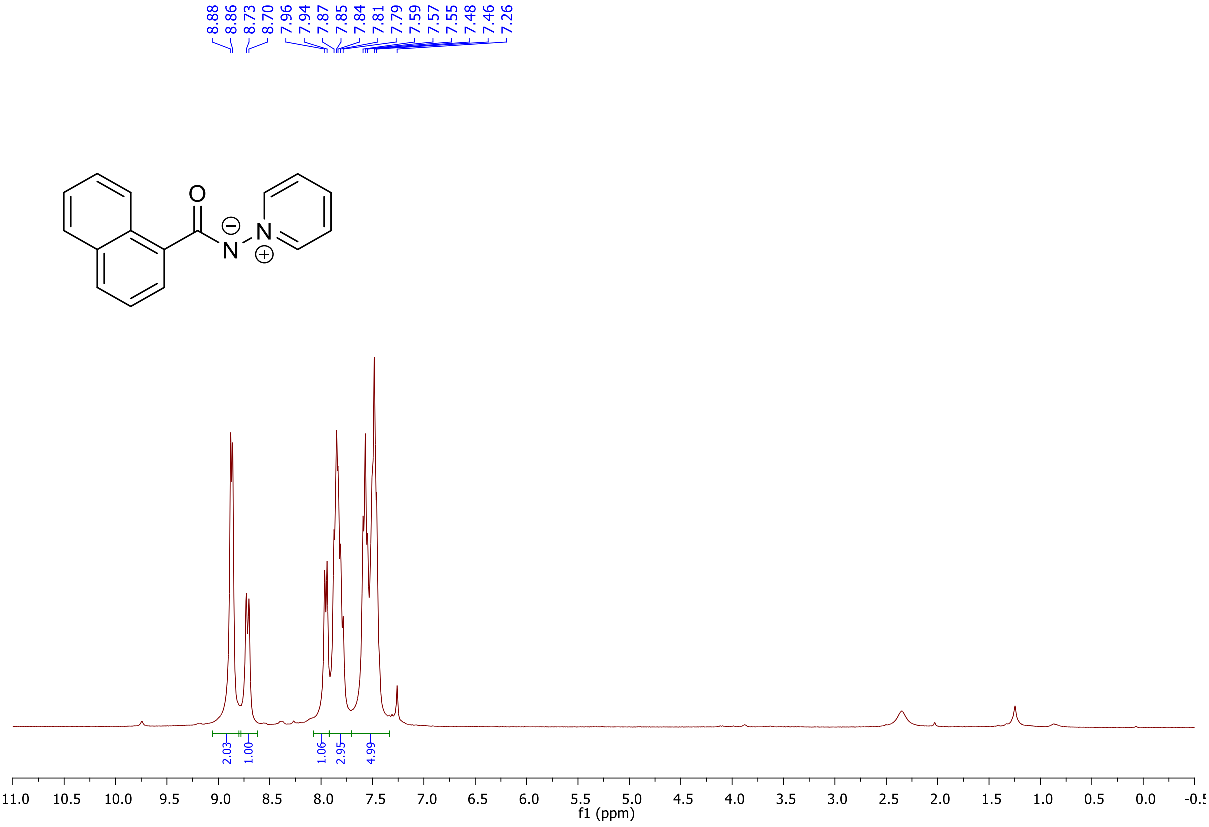

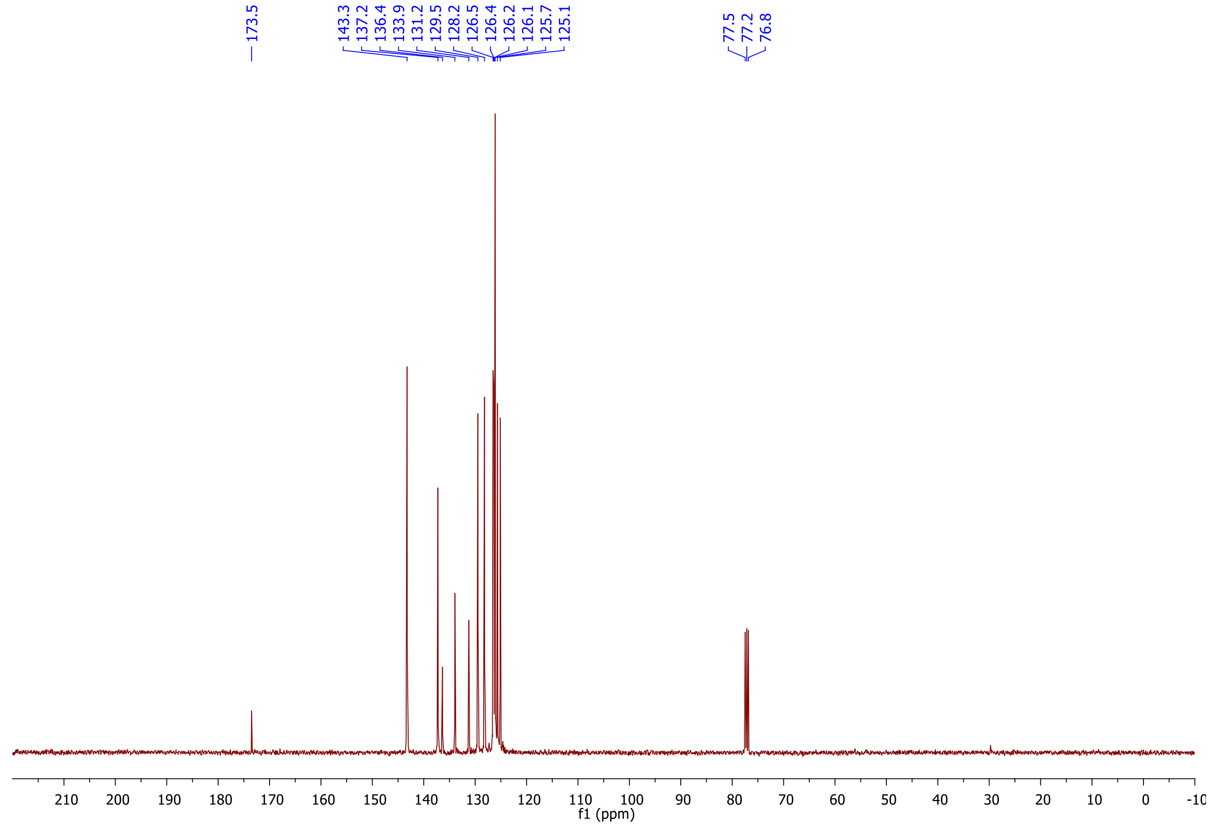

(2-Hydroxy-2-methylpropanoyl)(pyridin-1-ium-1-yl)amide (2g) in $CDCl_3$ ^{1}H-NMR and ^{13}C-NMR (Pendant)

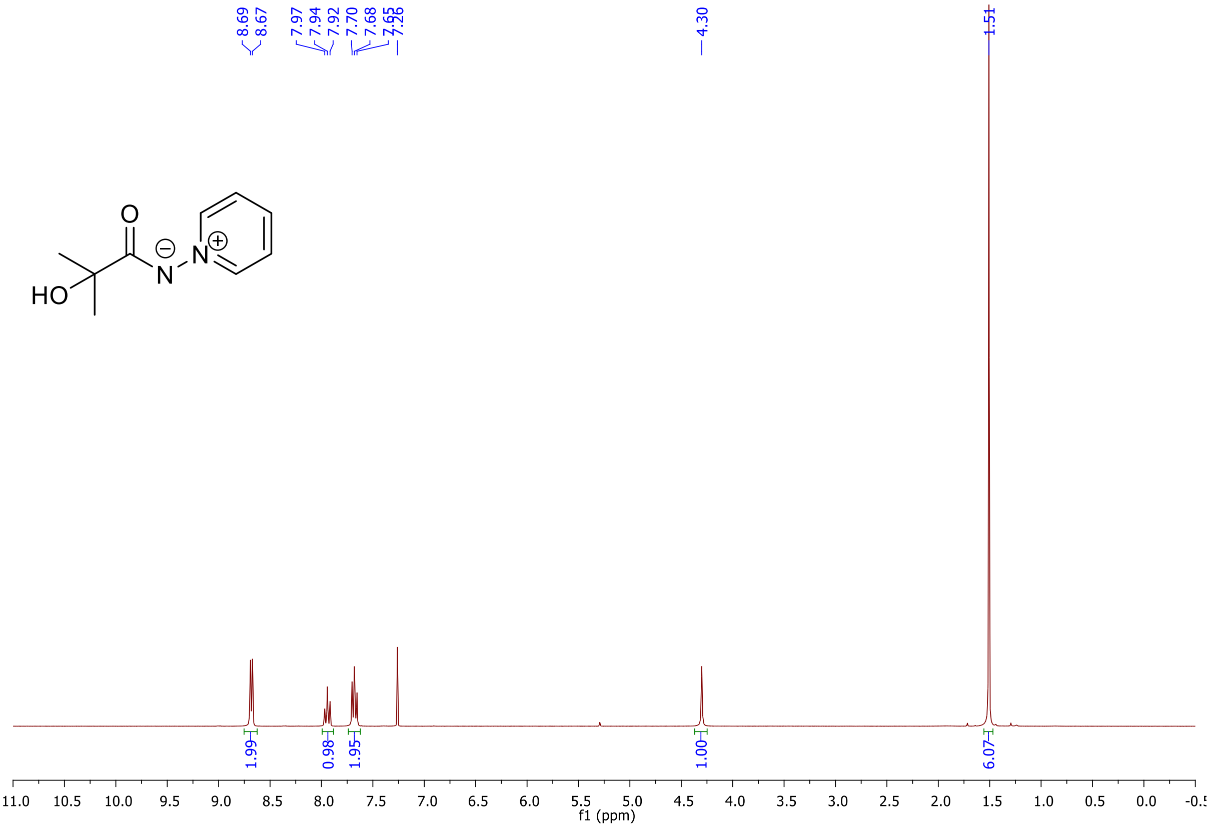

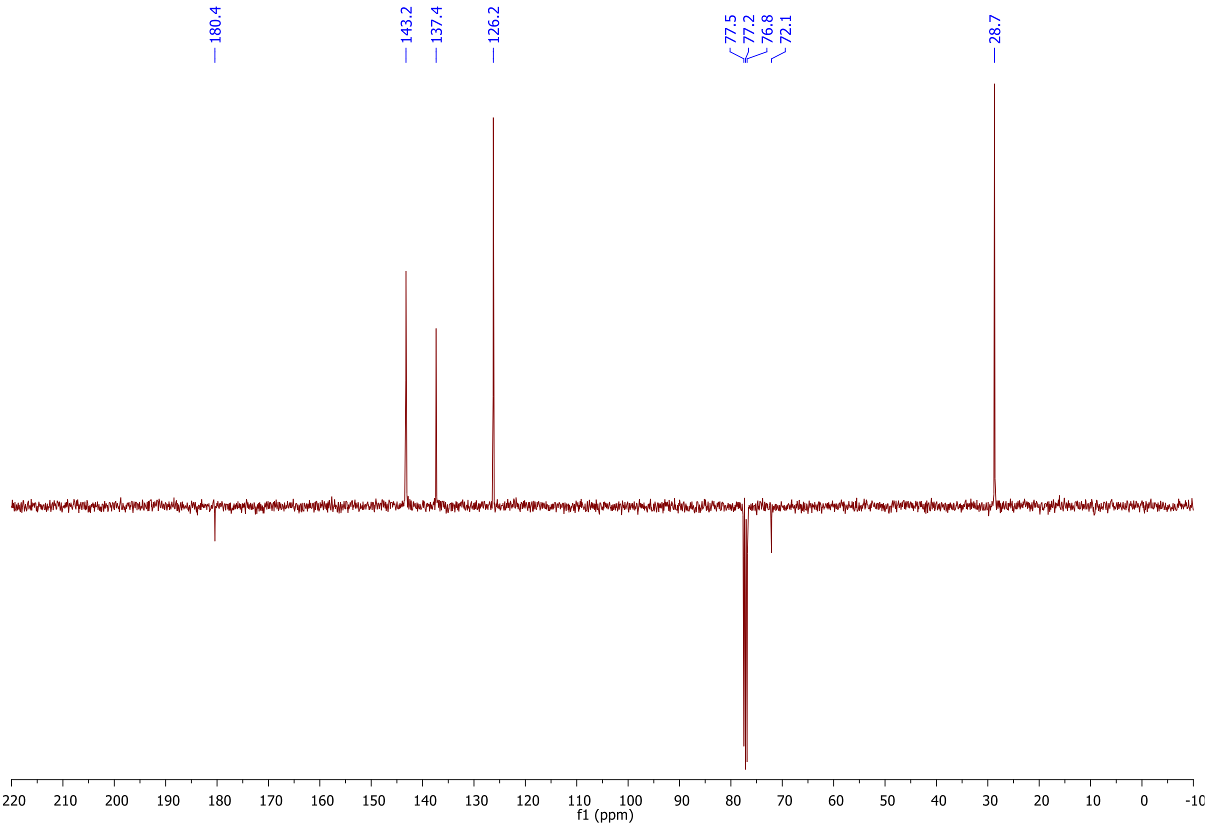

(3-Methoxy-3-oxopropanoyl)(pyridin-1-ium-1-yl)amide (2n) in $CDCl_3$ ^1H-NMR and ^{13}C-NMR (JMOD)

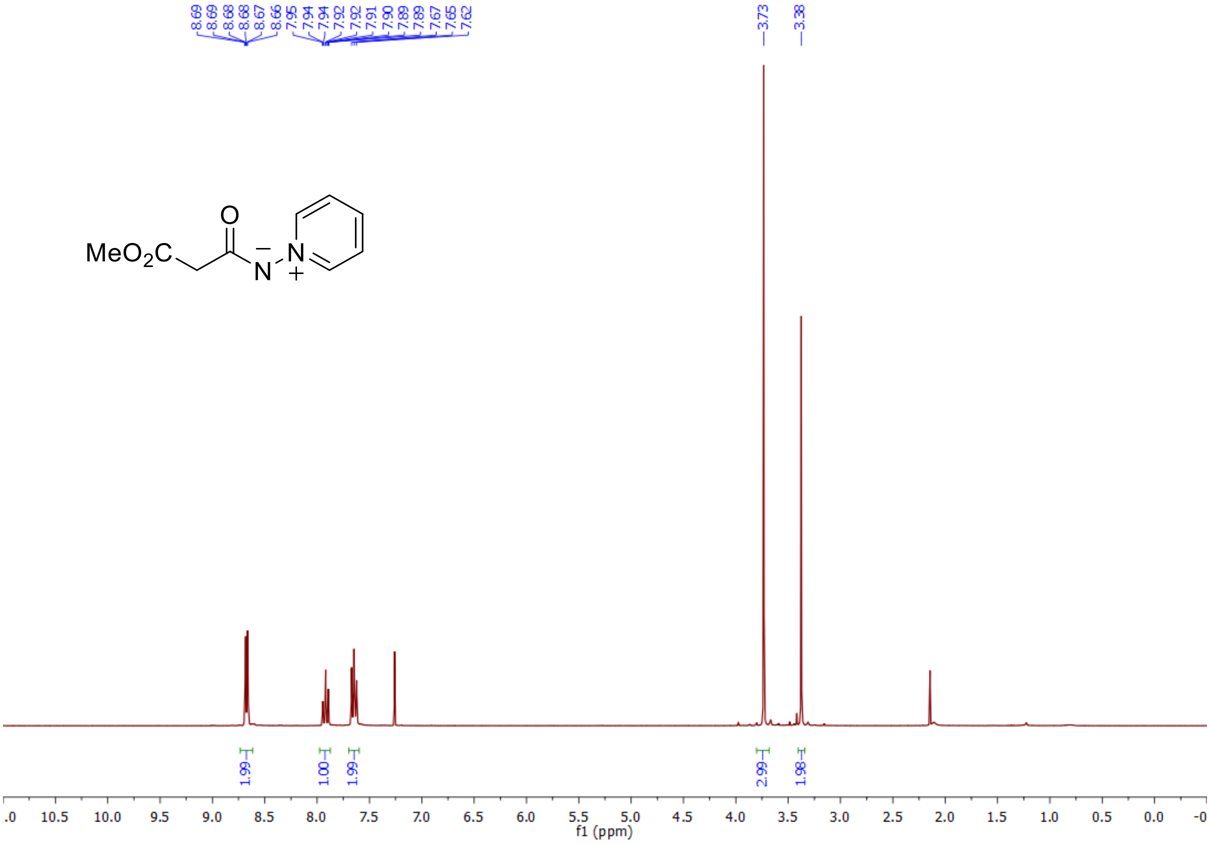

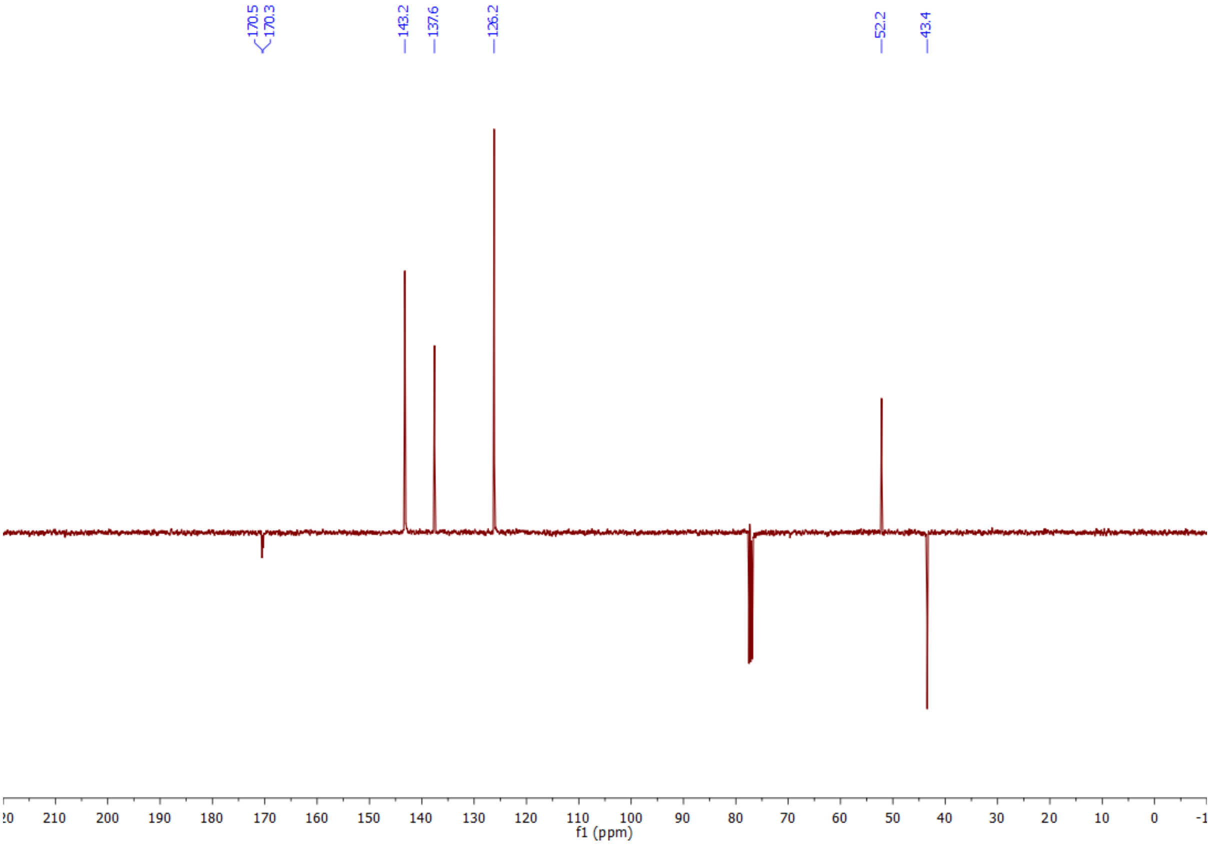

(2-(Methoxycarbonyl)benzoyl)(pyridin-1-ium-1-yl)amide (2l) in $CDCl_3$ ^{1}H-NMR and ^{13}C-NMR (JMOD)

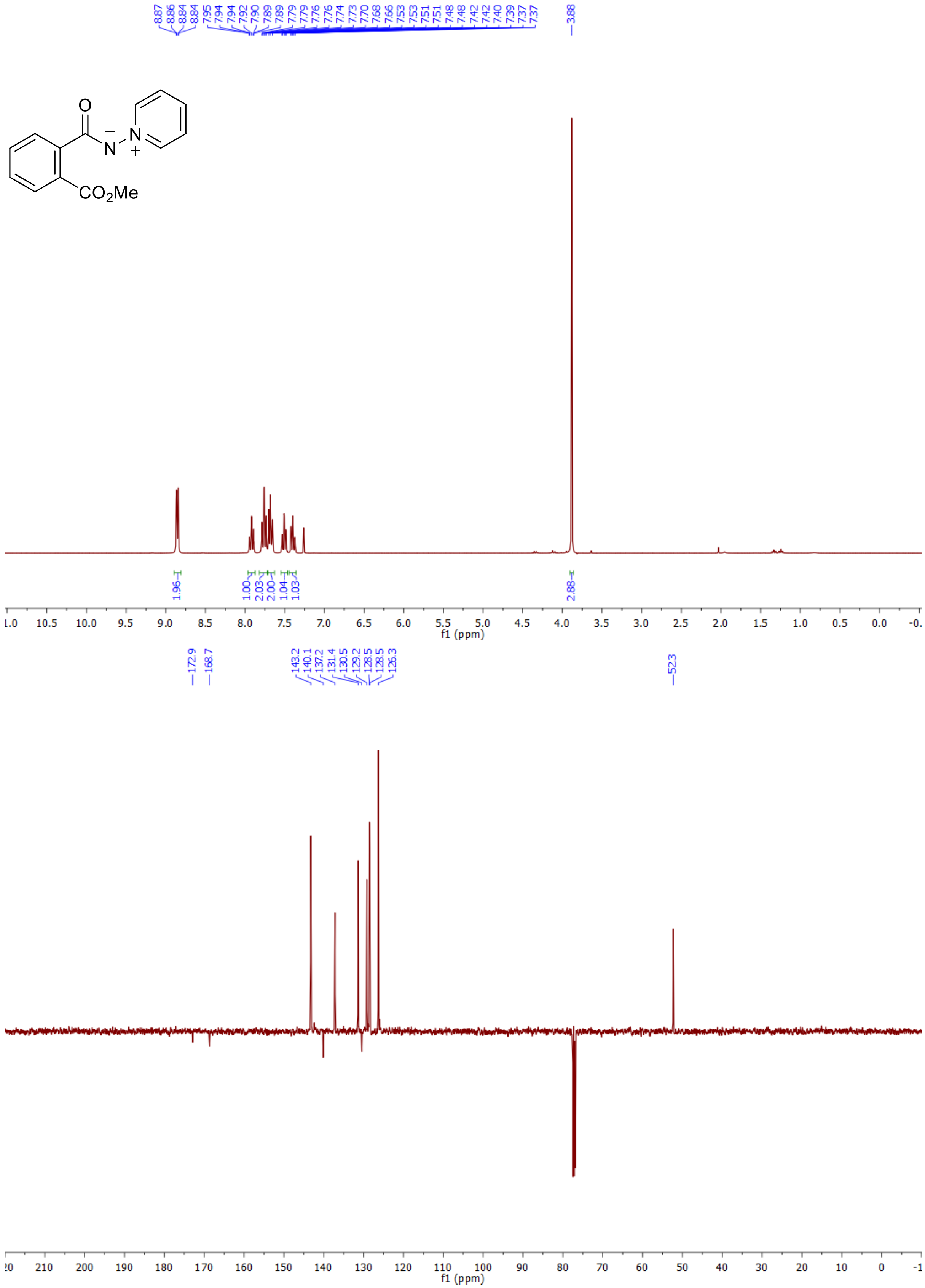

2,4-Diphenyl-5-(methylthio)oxazole (3aa-*8.4:1*) in $CDCl_3$ ^{1}H-NMR and ^{13}C-NMR (UDEFT)

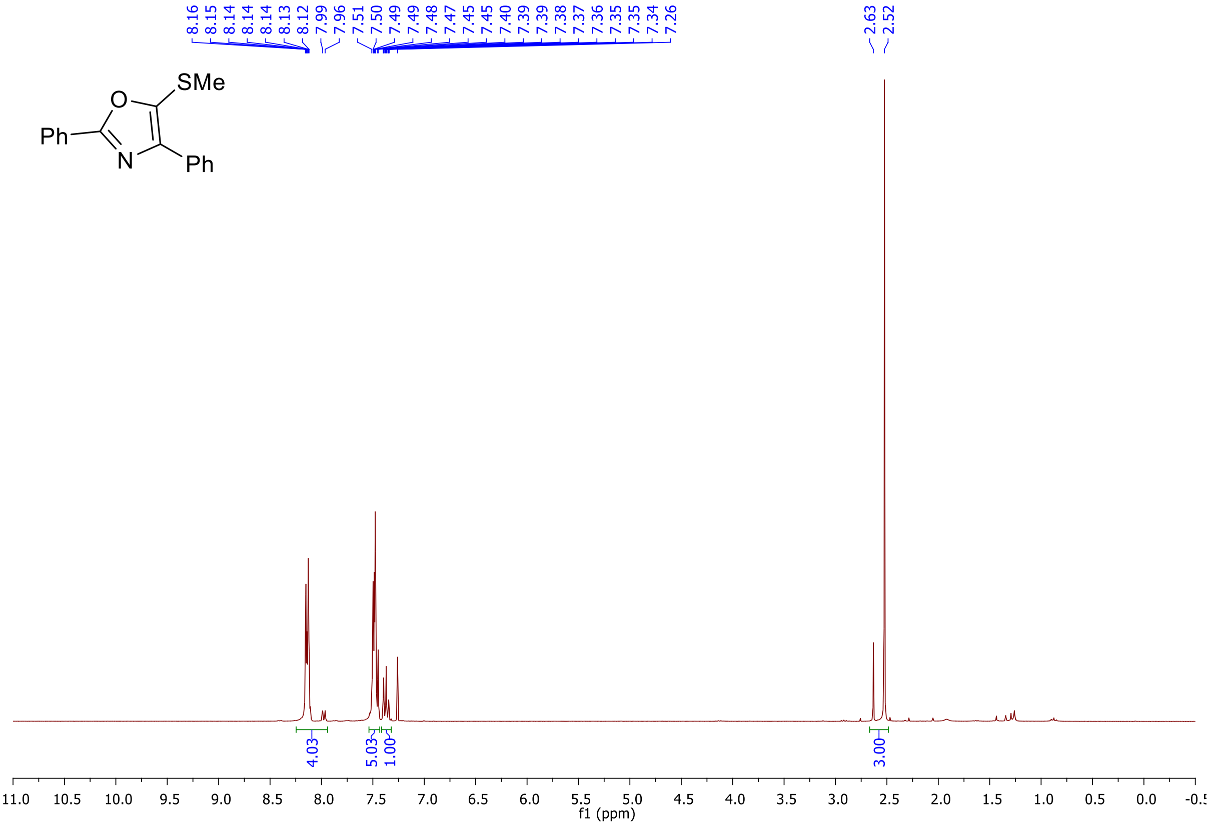

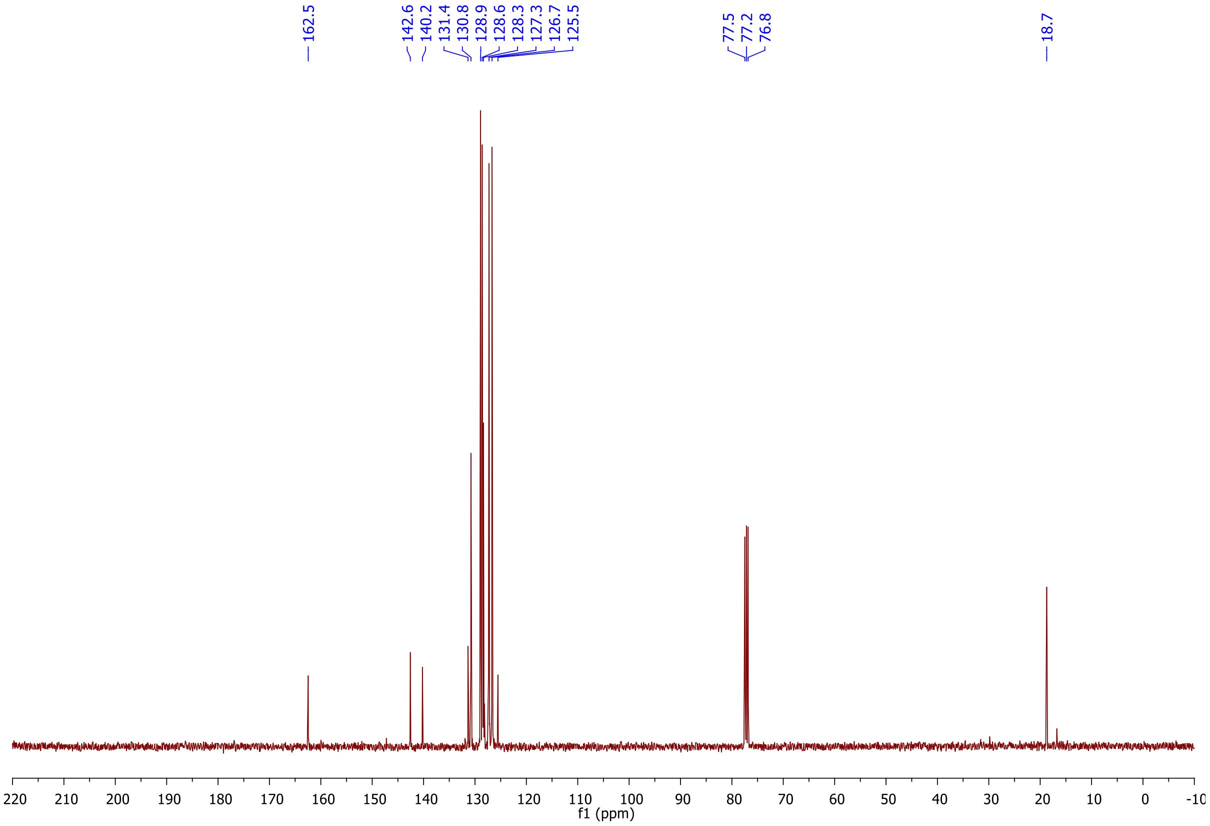

2,4-Diphenyl-5-(methylthio)oxazole (3aa-after crystallization-*20.0:1*) in $CDCl_3$ ^{1}H-NMR and ^{13}C-NMR (UDEFT)

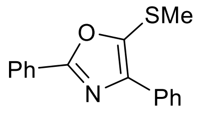

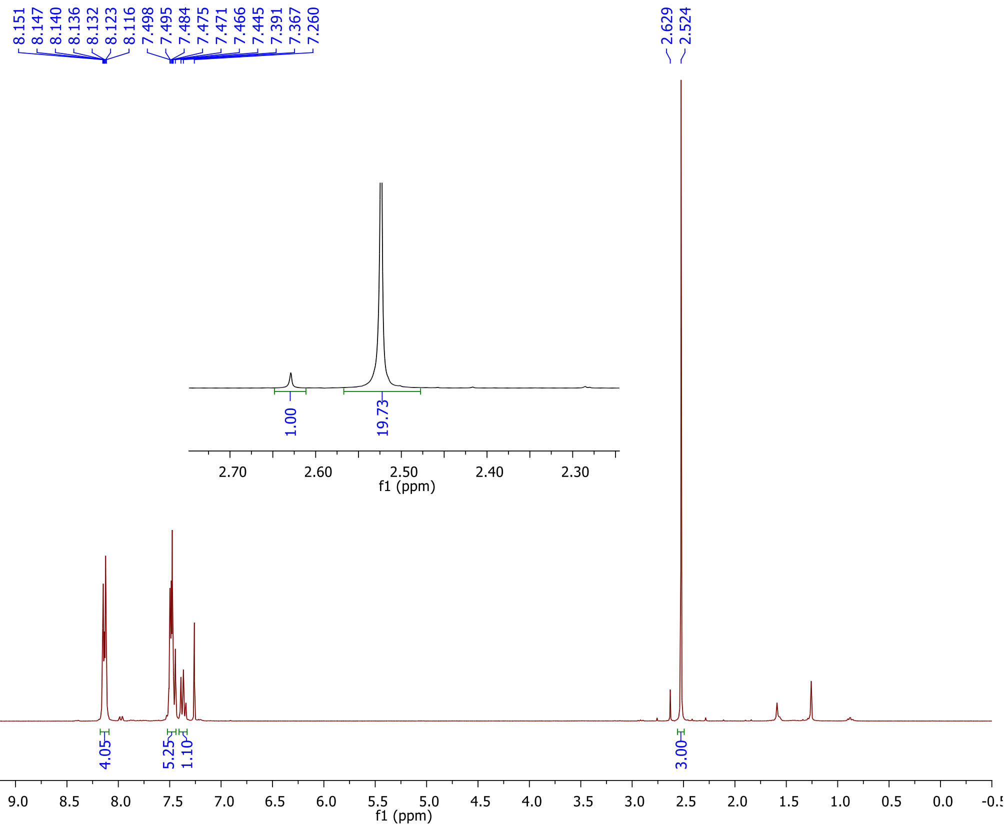

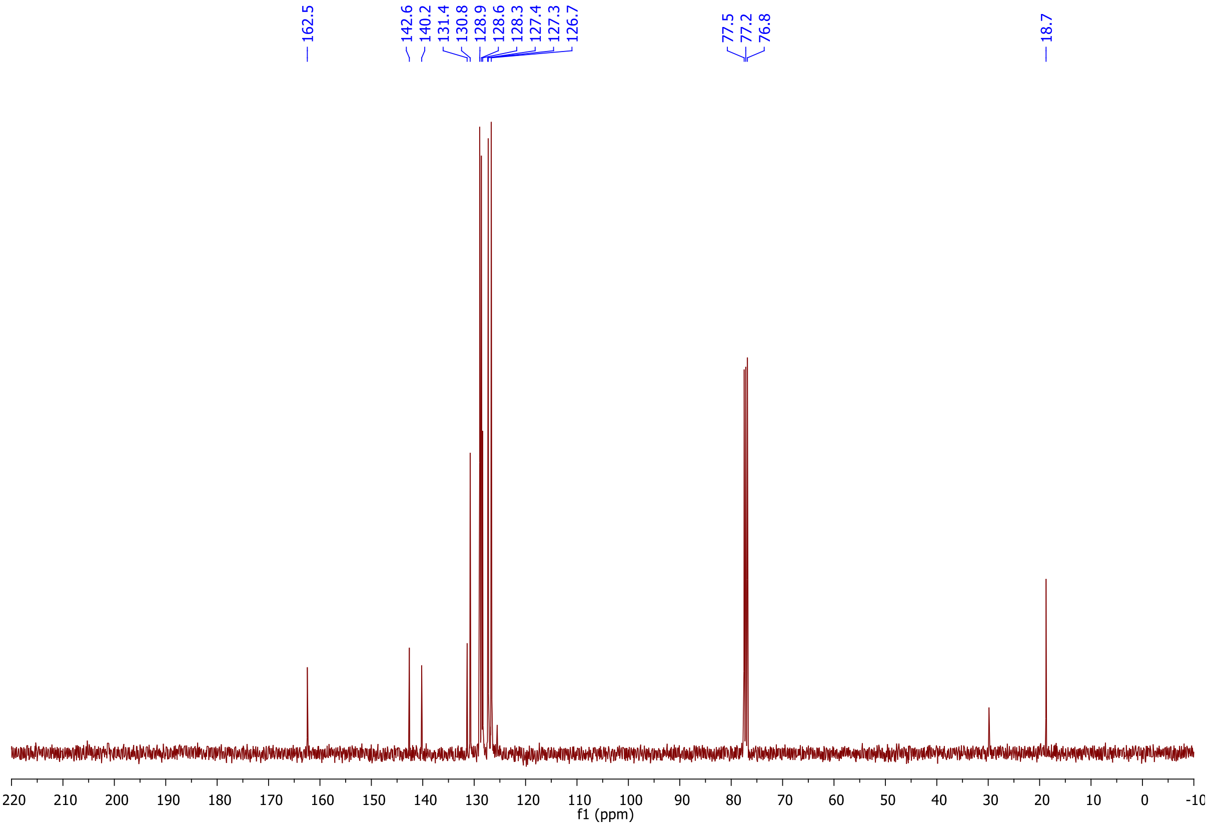

2,4-Diphenyl-5-(ethylthio)oxazole (3ba-*6.5:1*) in $CDCl_3$ ^{1}H-NMR and ^{13}C-NMR (Pendant)

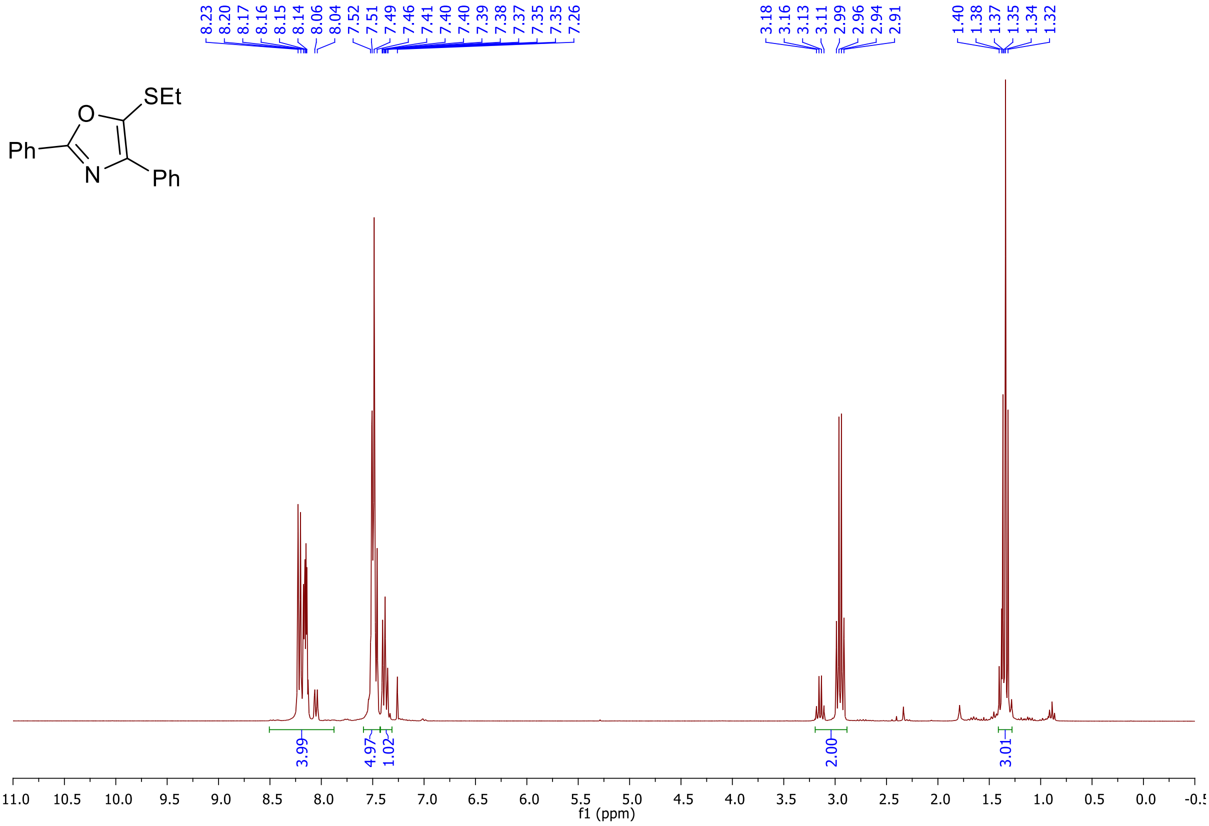

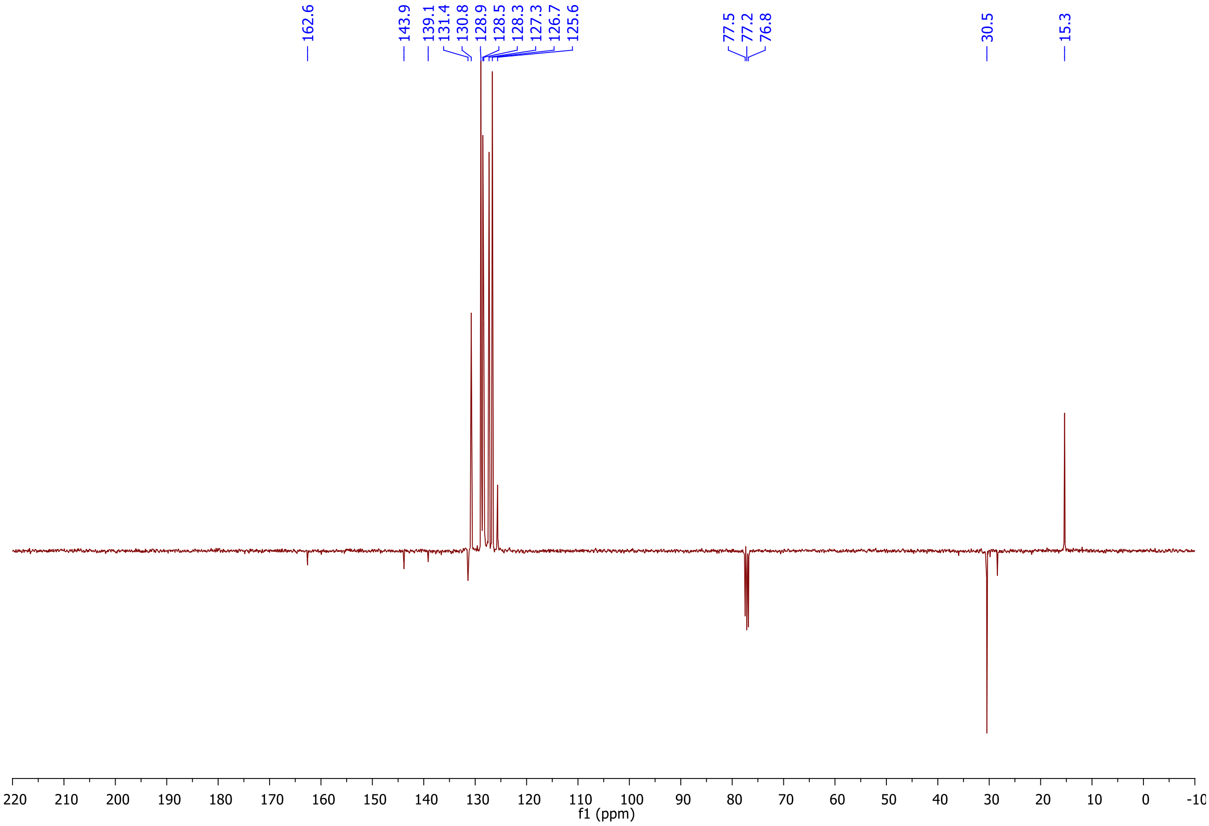

2,4-Diphenyl-5-(isopropylthio)oxazole (3ca-*4.5:1*) in $CDCl_3$ ^{1}H-NMR and ^{13}C-NMR (Pendant)

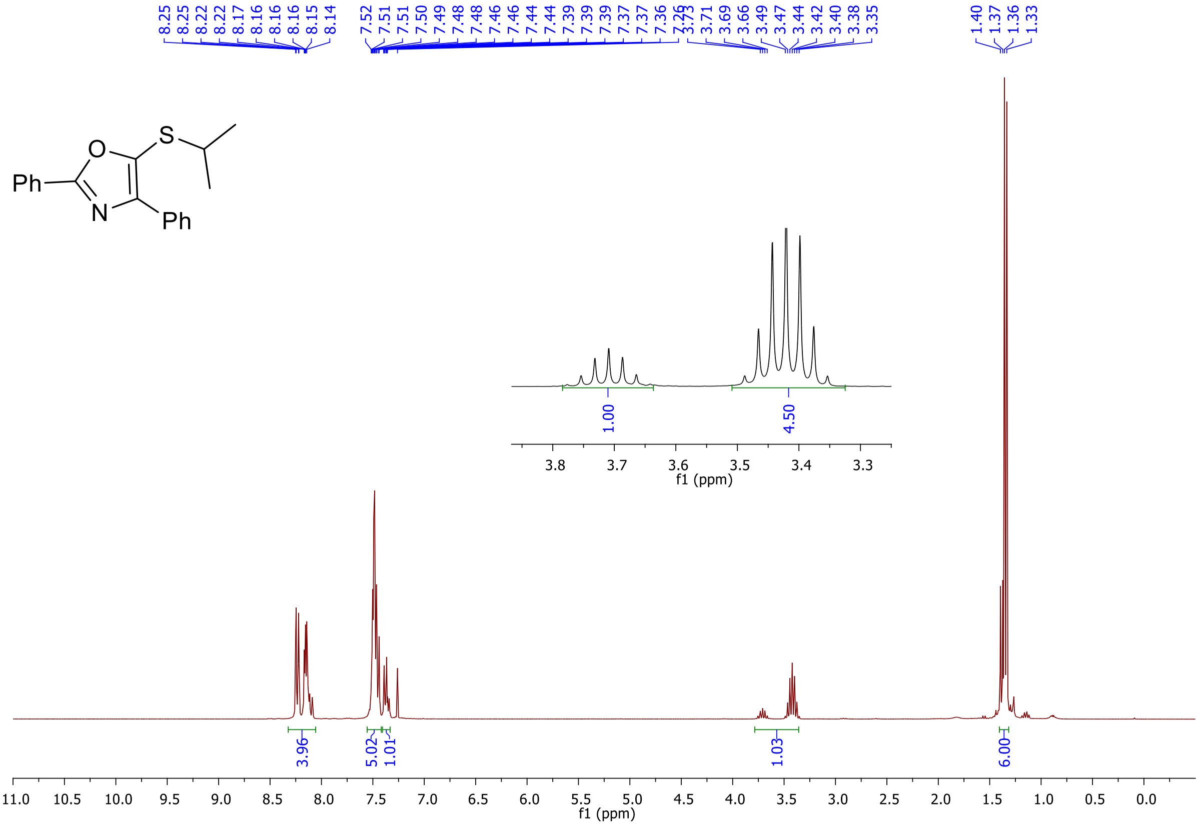

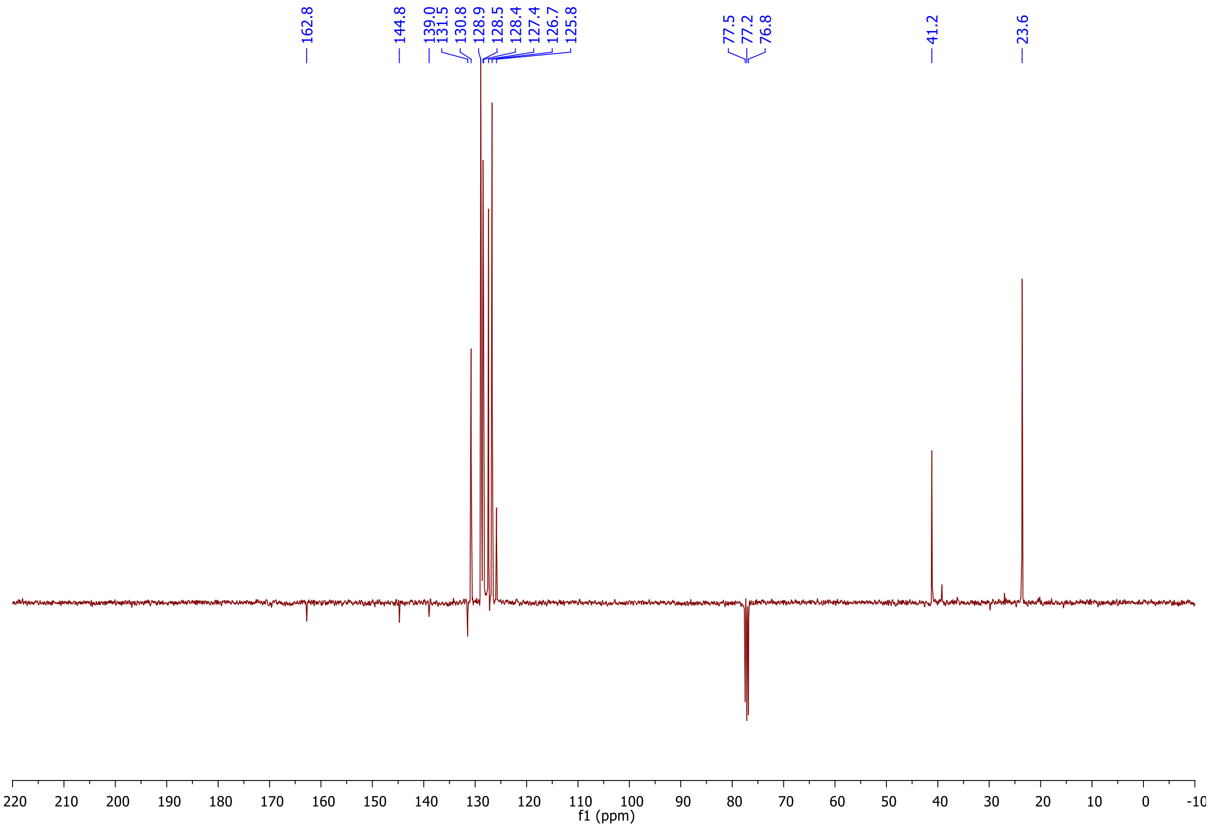

2,4-Diphenyl-5-(phenylthio)oxazole (3da-*4.8:1*) in $CDCl_3$ ^1H-NMR and ^{13}C-NMR (UDEFT)

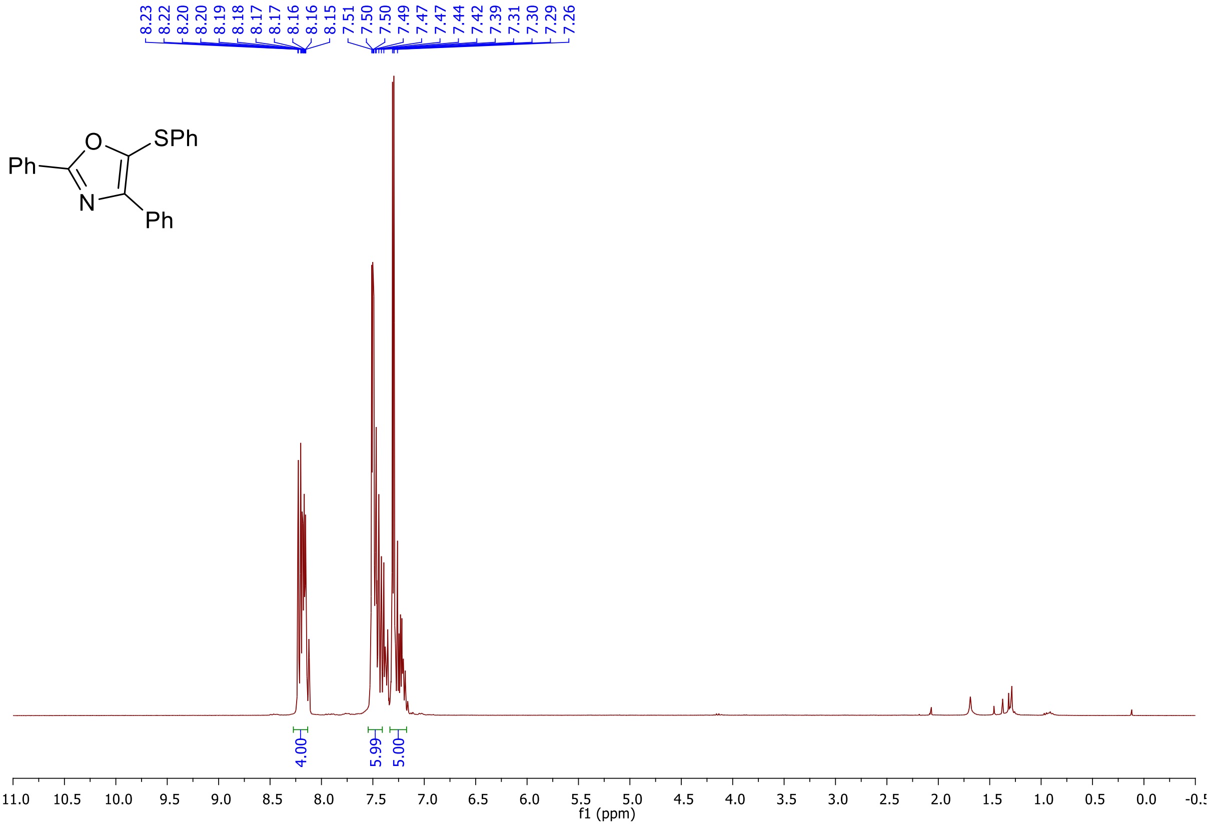

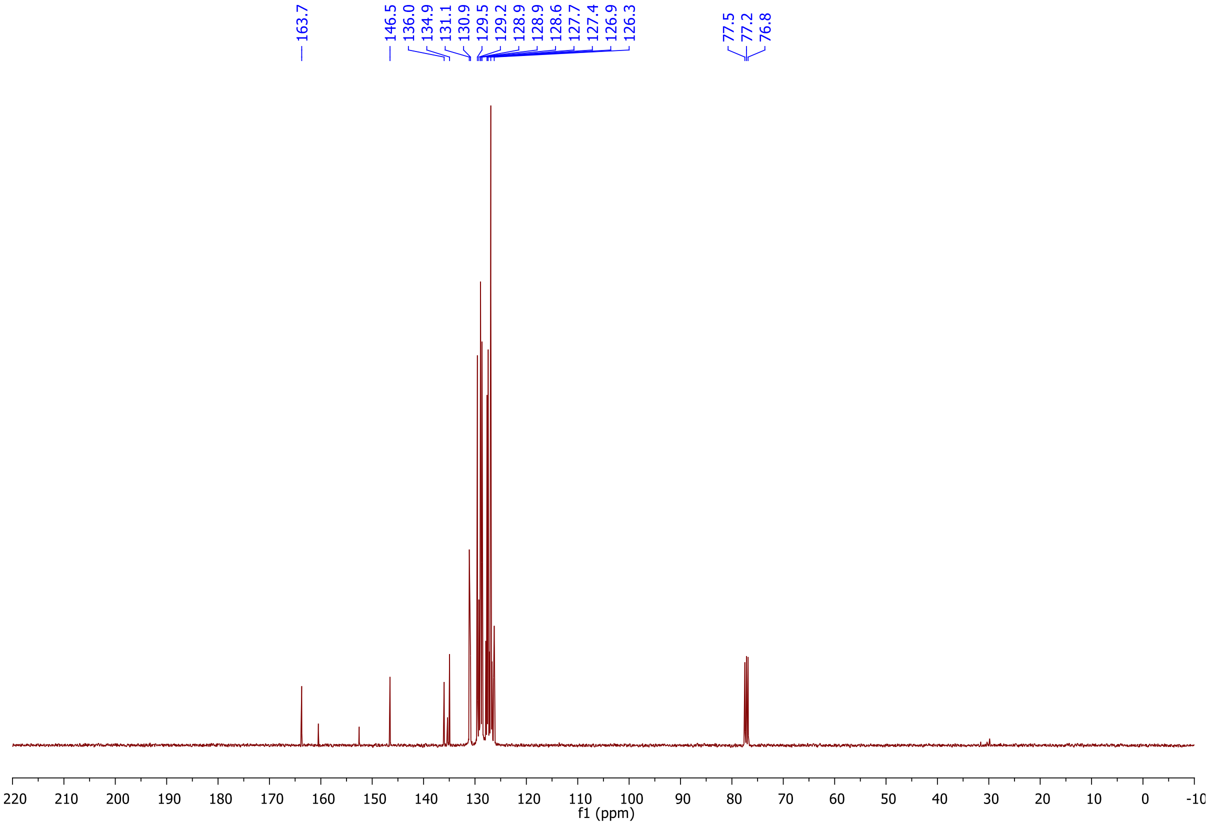

5-(Benzylthio)-2,4-diphenyloxazole (3ea-*6.3:1*) in $CDCl_3$ ^1H-NMR and ^{13}C-NMR (UDEFT)

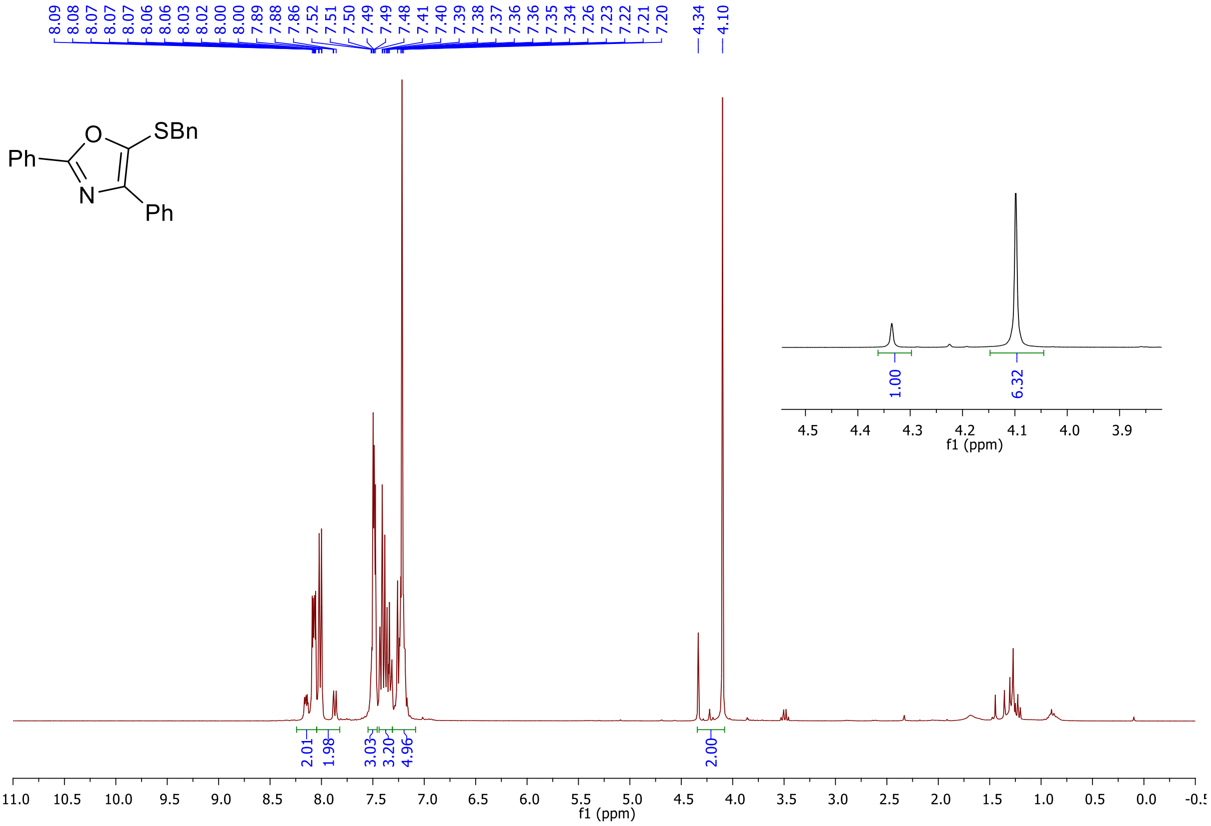

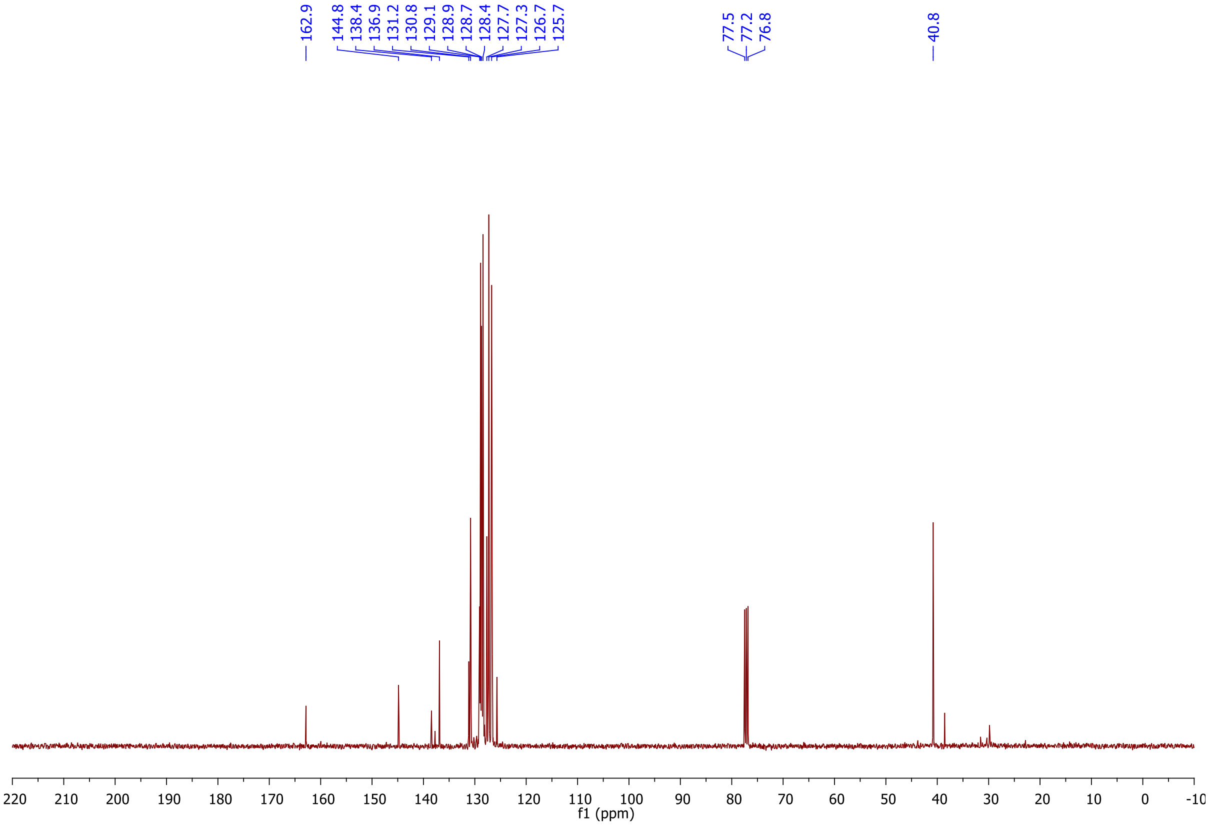

5-(Ethylthio)-4-(4-methoxyphenyl)-2-phenyloxazole (3ga-*21.0:1*) in $CDCl_3$ ^1H-NMR and ^{13}C-NMR (Pendant)

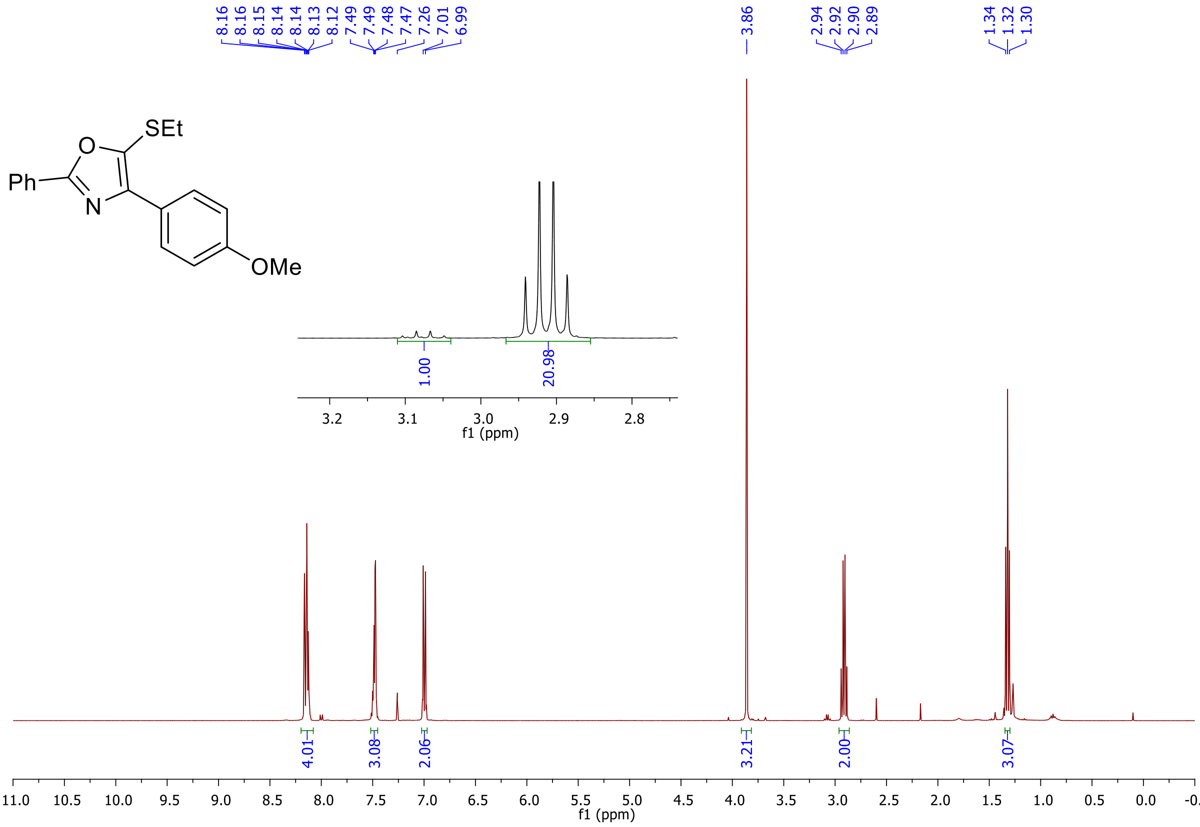

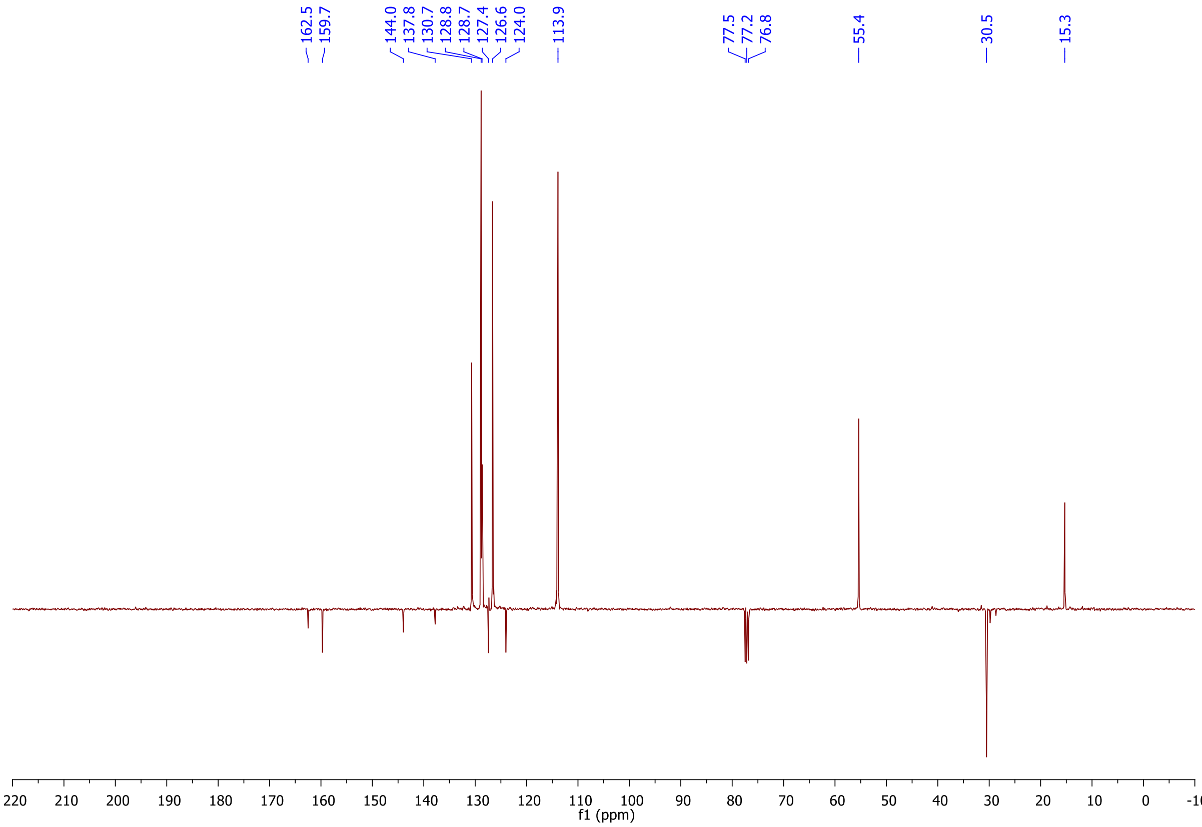

5-(Ethylthio)-4-(4-methoxyphenyl)-2-phenyloxazole (3ga-after crystallization) in $CDCl_3$ ^{1}H-NMR

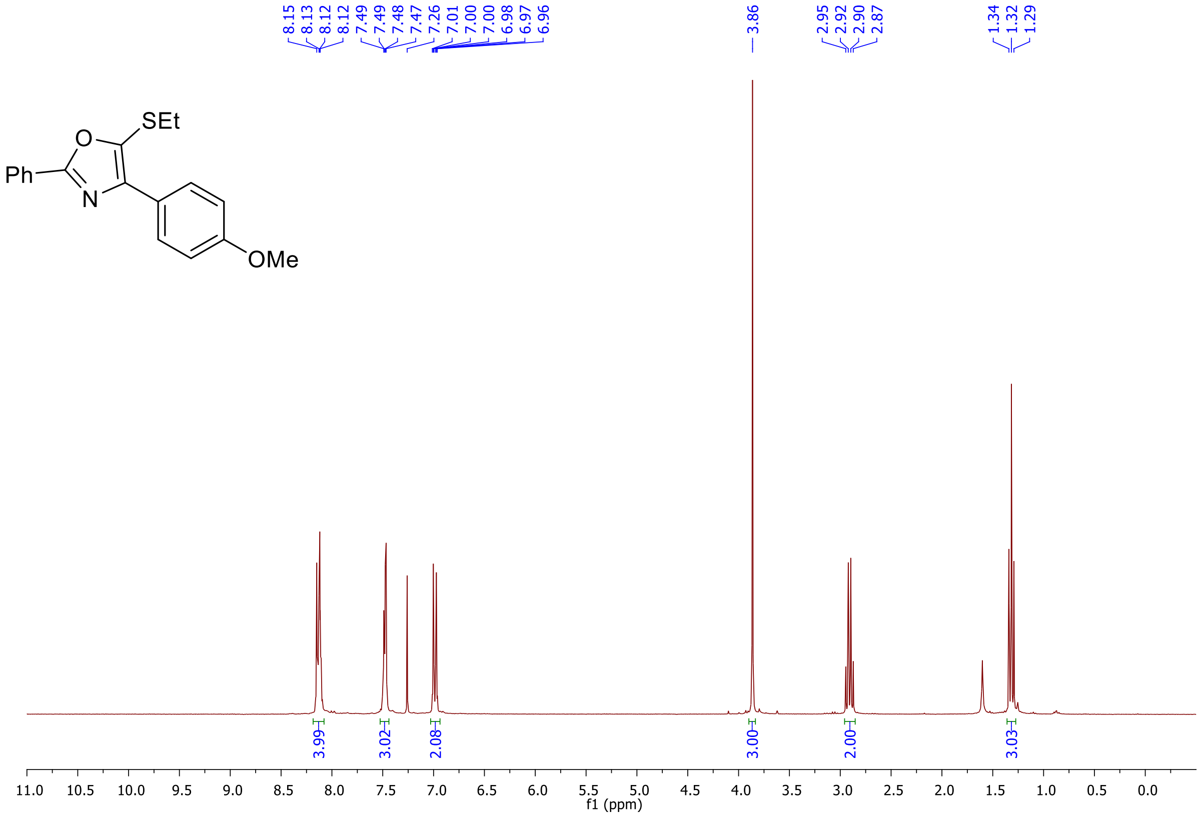

3ga (>21:1) ***Vs*** **(>99:1)** in $CDCl_3$ ^{1}H-NMR

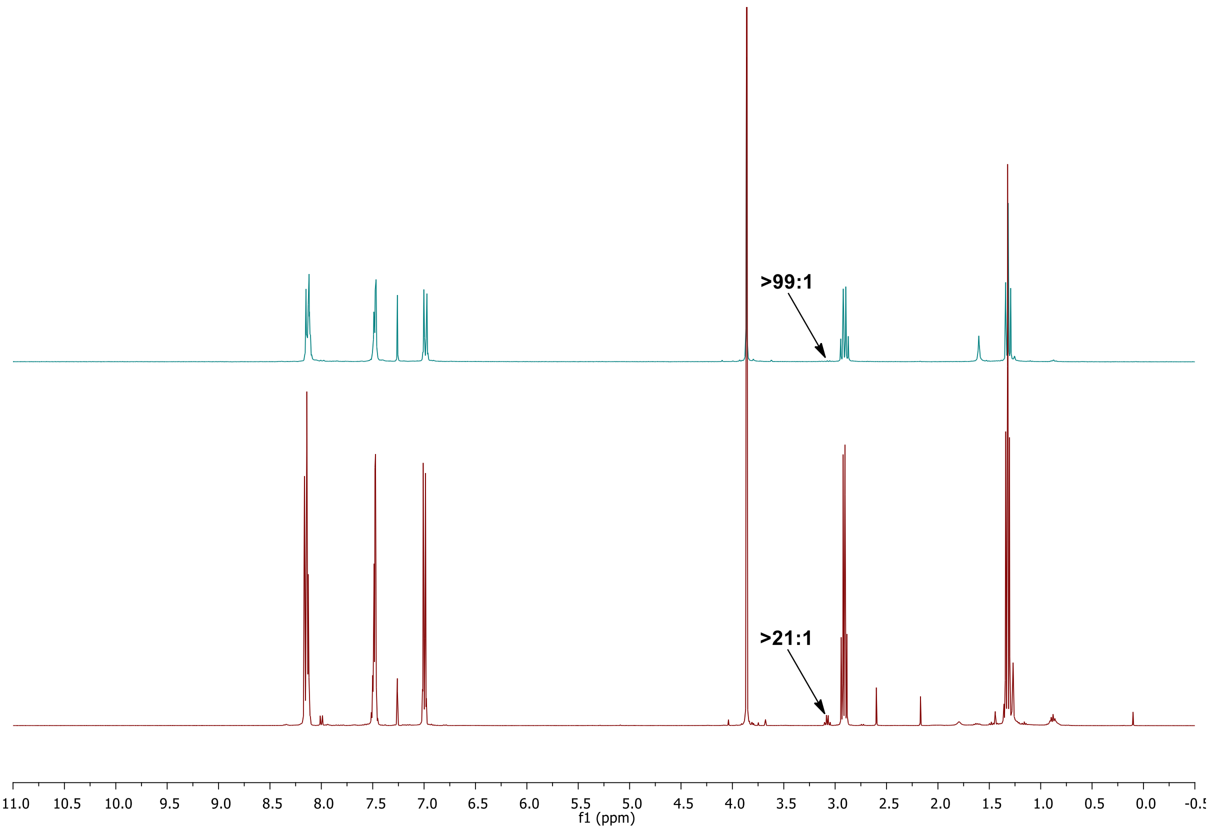

4-(4-Methoxyphenyl)-2-phenyl-5-(phenylthio)oxazole (3ha-*15.0:1*) in $CDCl_3$ ^{1}H-NMR and ^{13}C-NMR (Pendant)

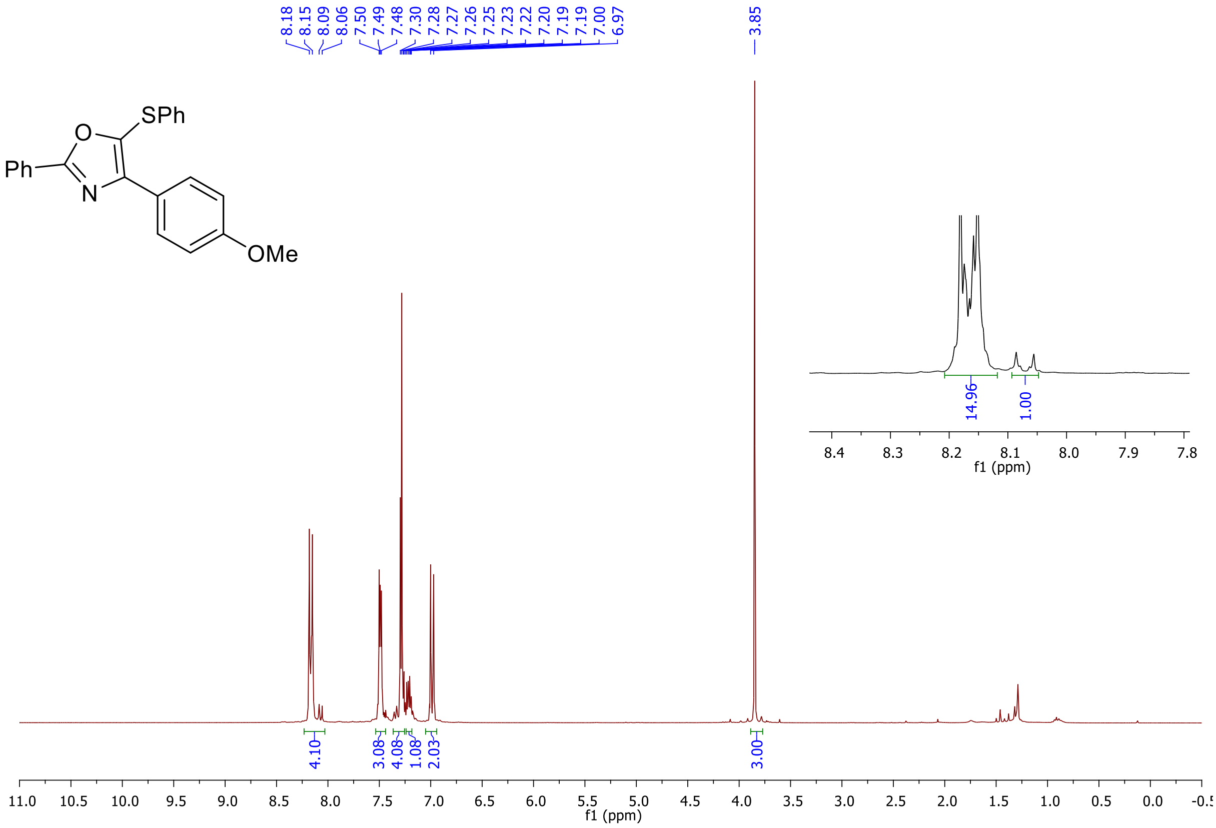

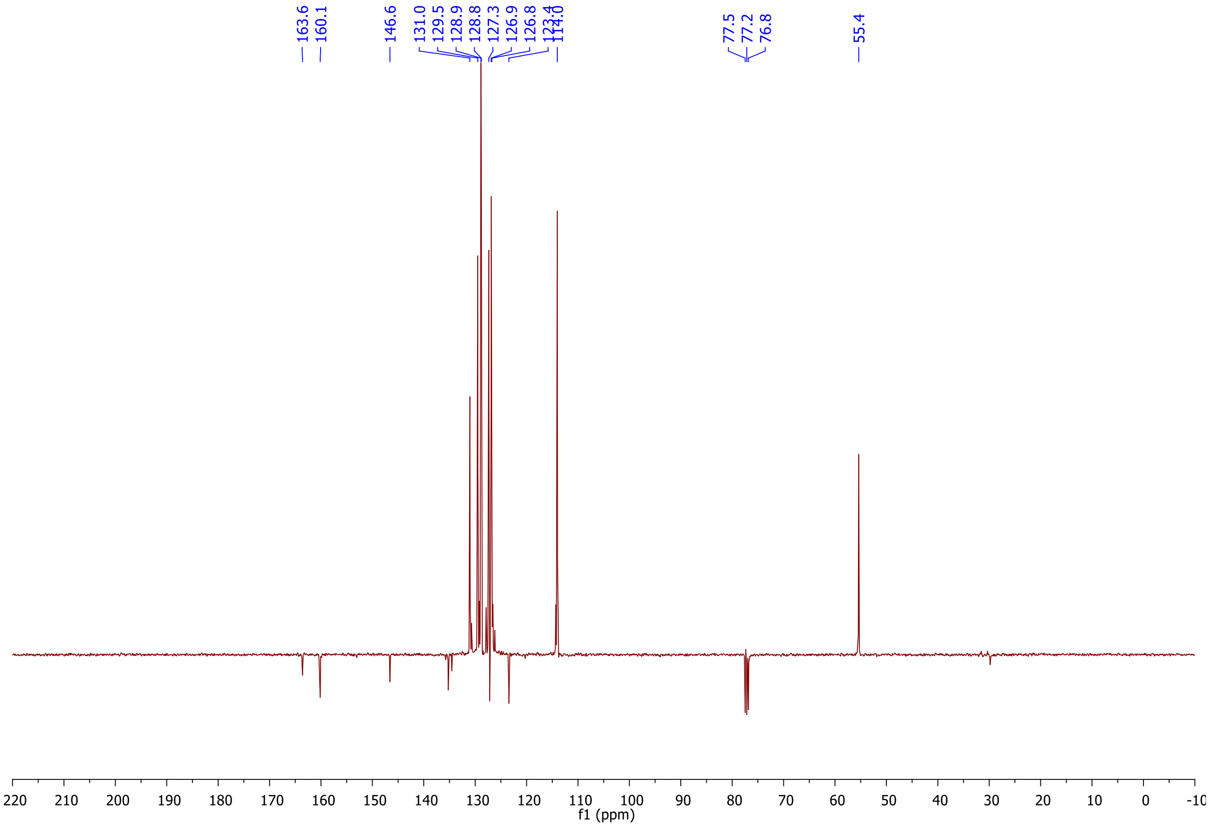

4-(4-Methoxyphenyl)-5-(methylthio)-2-phenyloxazole (3ia-*26.0:1*) in $CDCl_3$ ^{1}H-NMR and ^{13}C-NMR (Pendant)

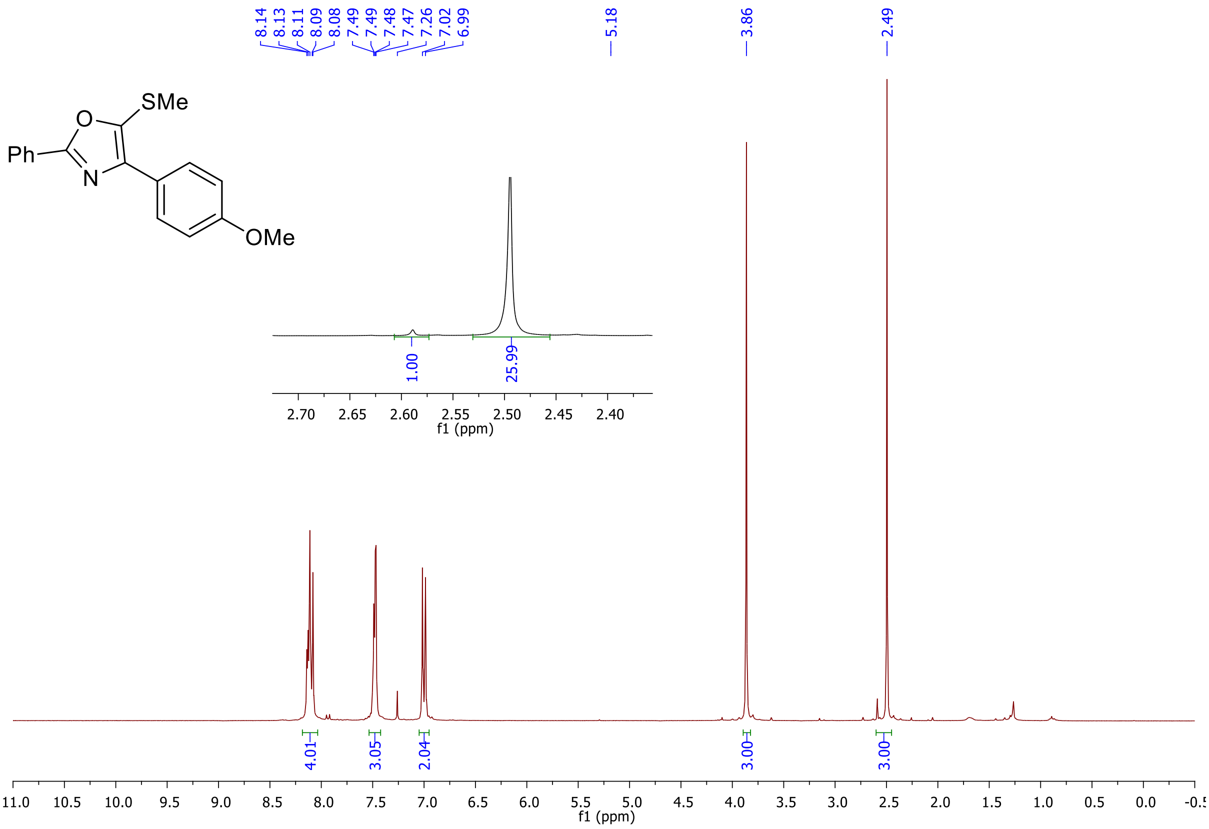

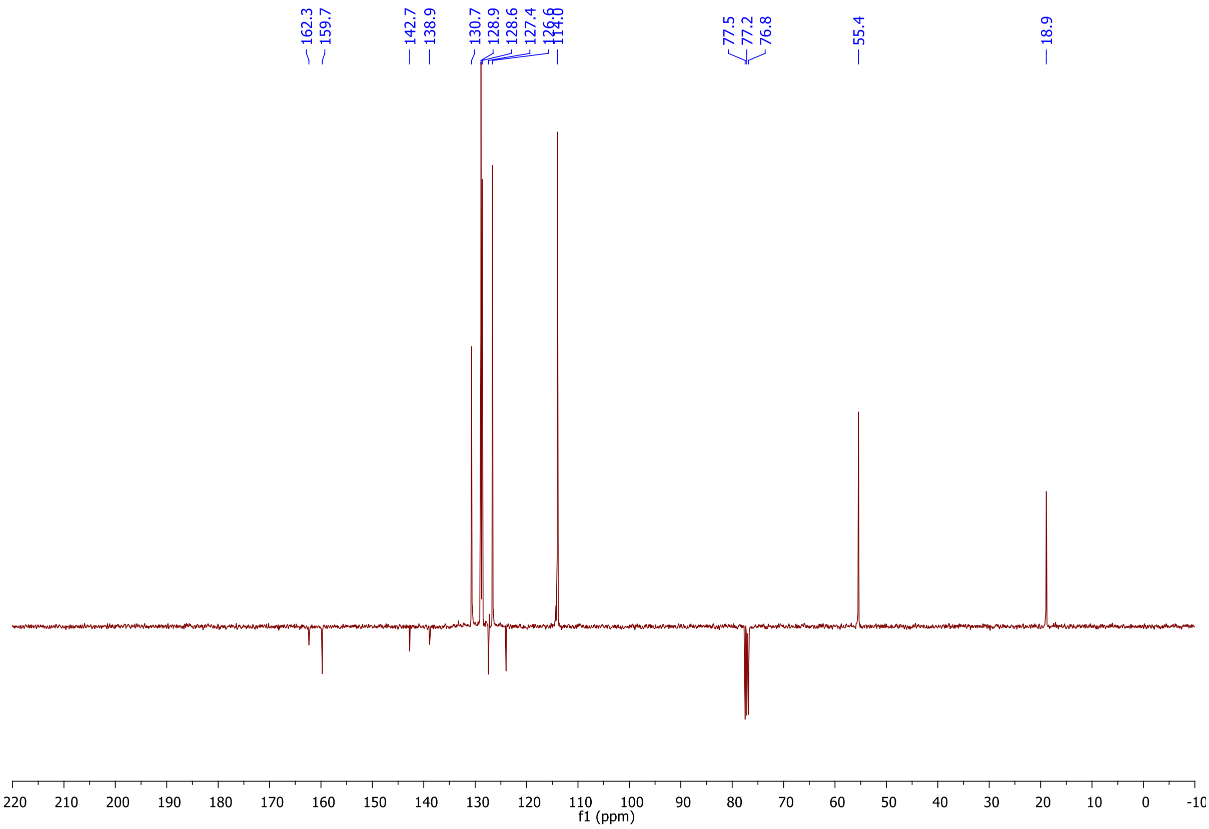

2-(2-Bromophenyl)-5-(ethylthio)-4-phenyloxazole (3bb-*5.0:1*) in $CDCl_3$ ^1H-NMR and ^{13}C-NMR (Pendant)

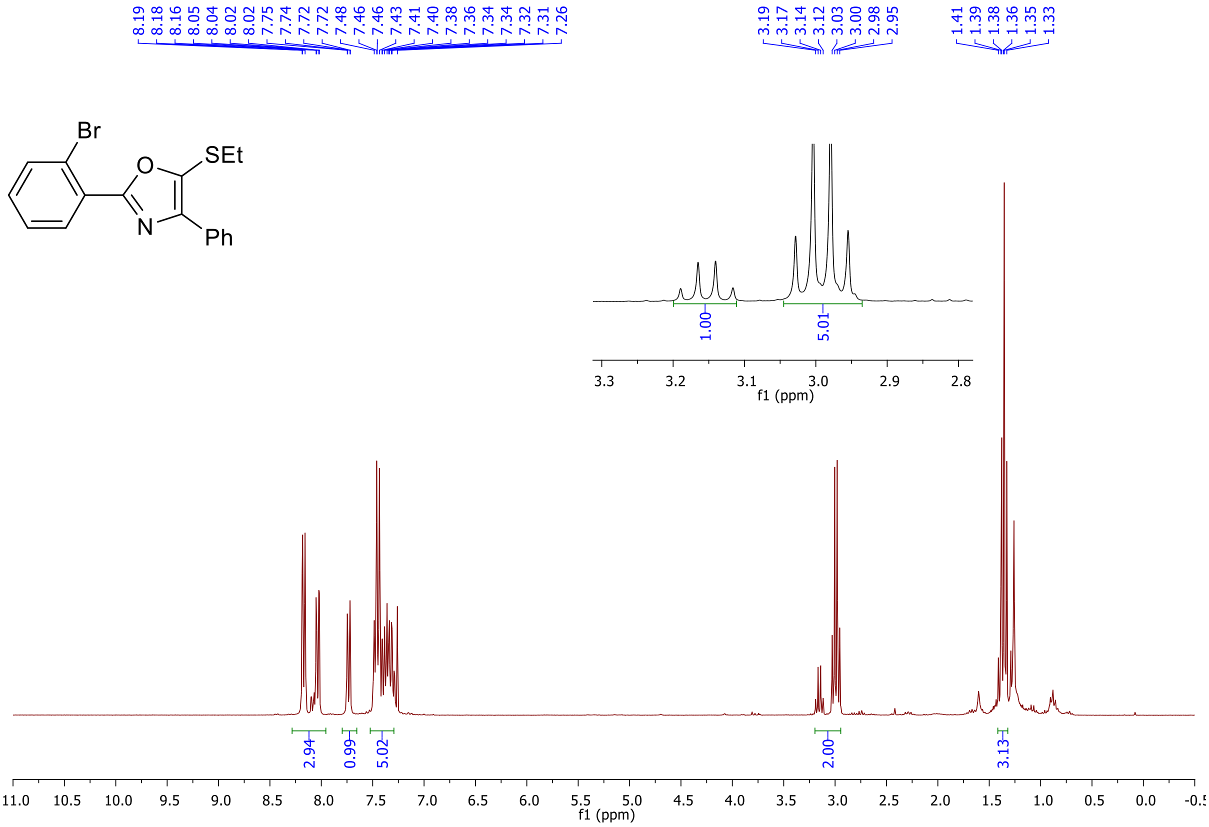

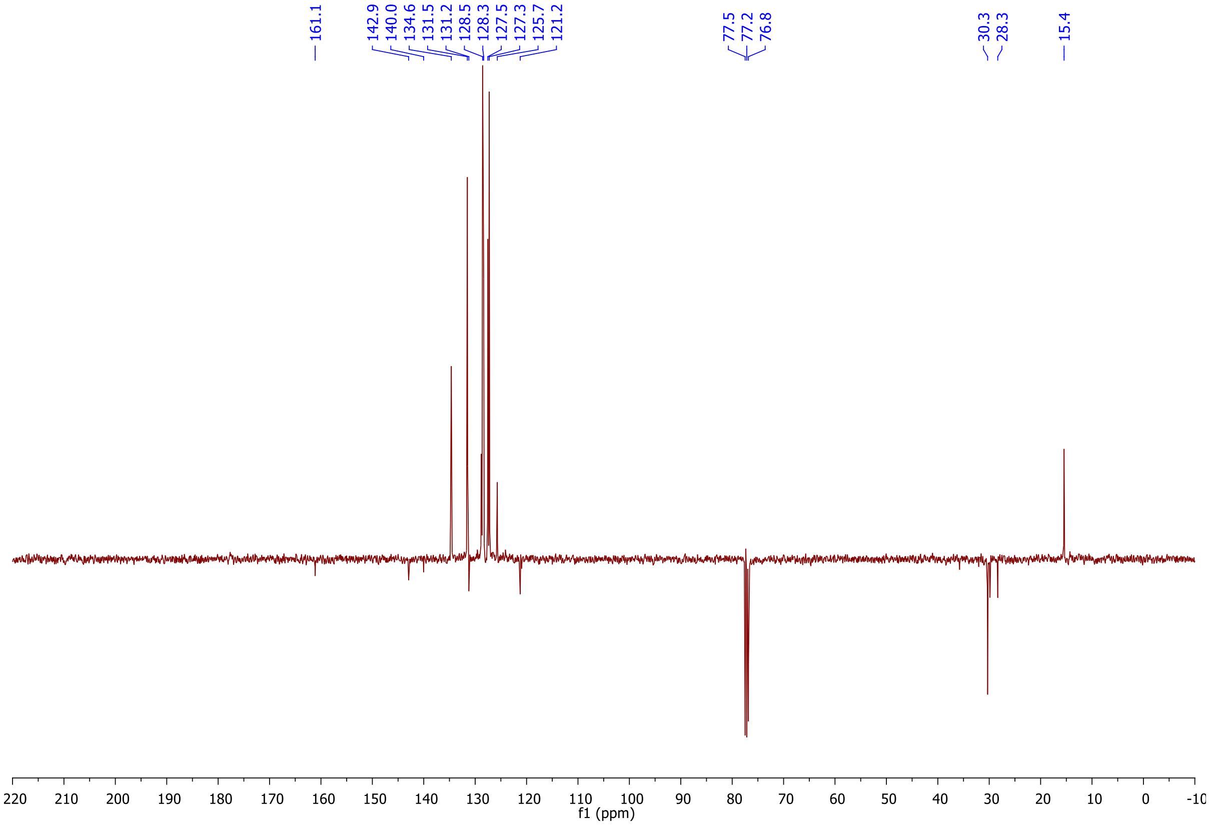

2-(2-Bromophenyl)-5-(ethylthio)-4-(4-methoxyphenyl)oxazole (3gb-*10.0:1*) in $CDCl_3$ ^{1}H-NMR and ^{13}C-NMR (Pendant)

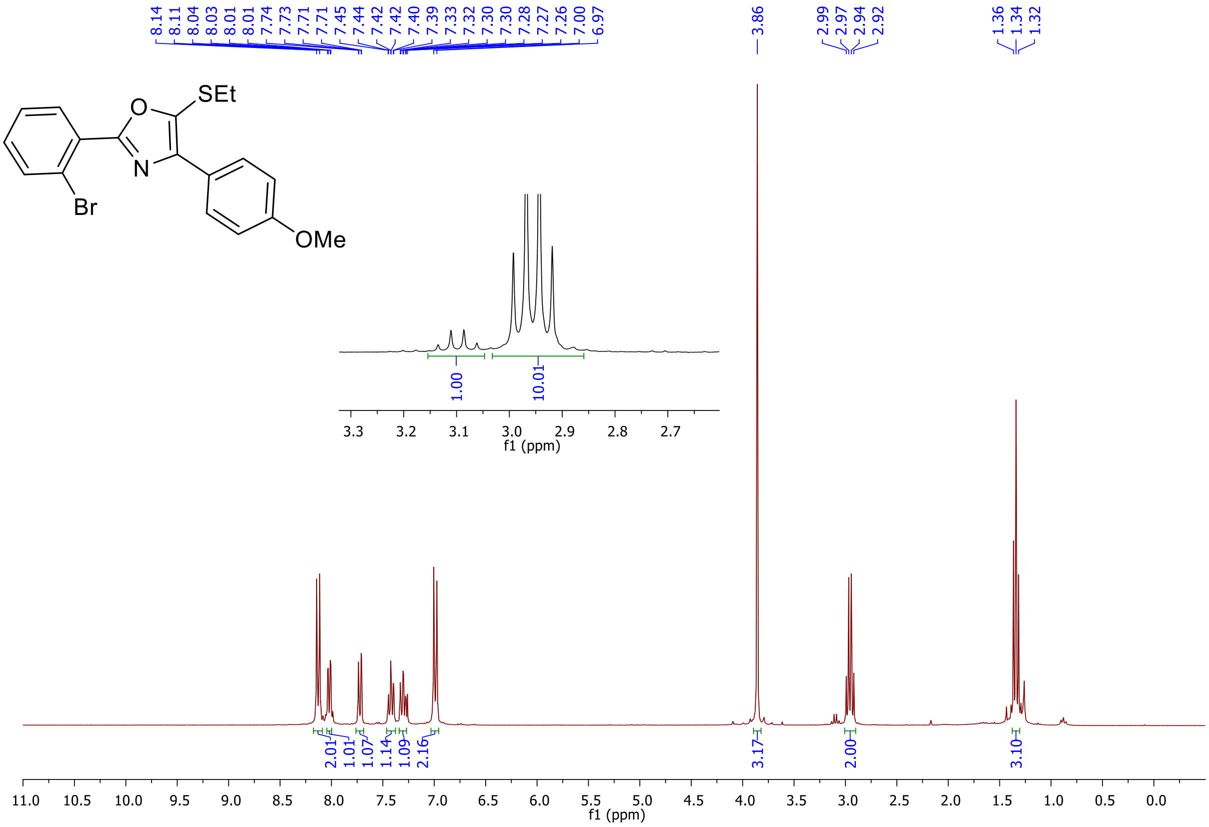

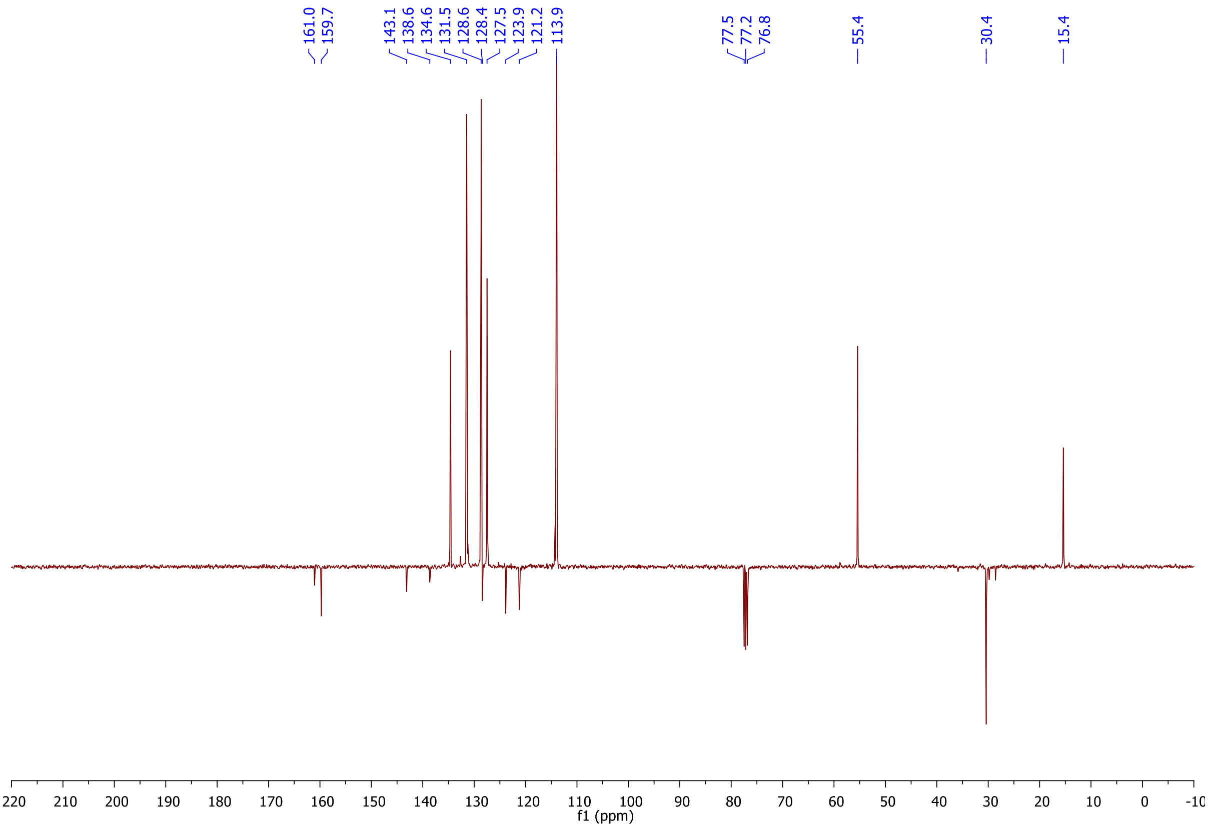

5-(Methylthio)-2-(naphthalen-2-yl)-4-phenyloxazole (3ac-*8.0:1*) in $CDCl_3$ ^{1}H-NMR and ^{13}C-NMR (Pendant)

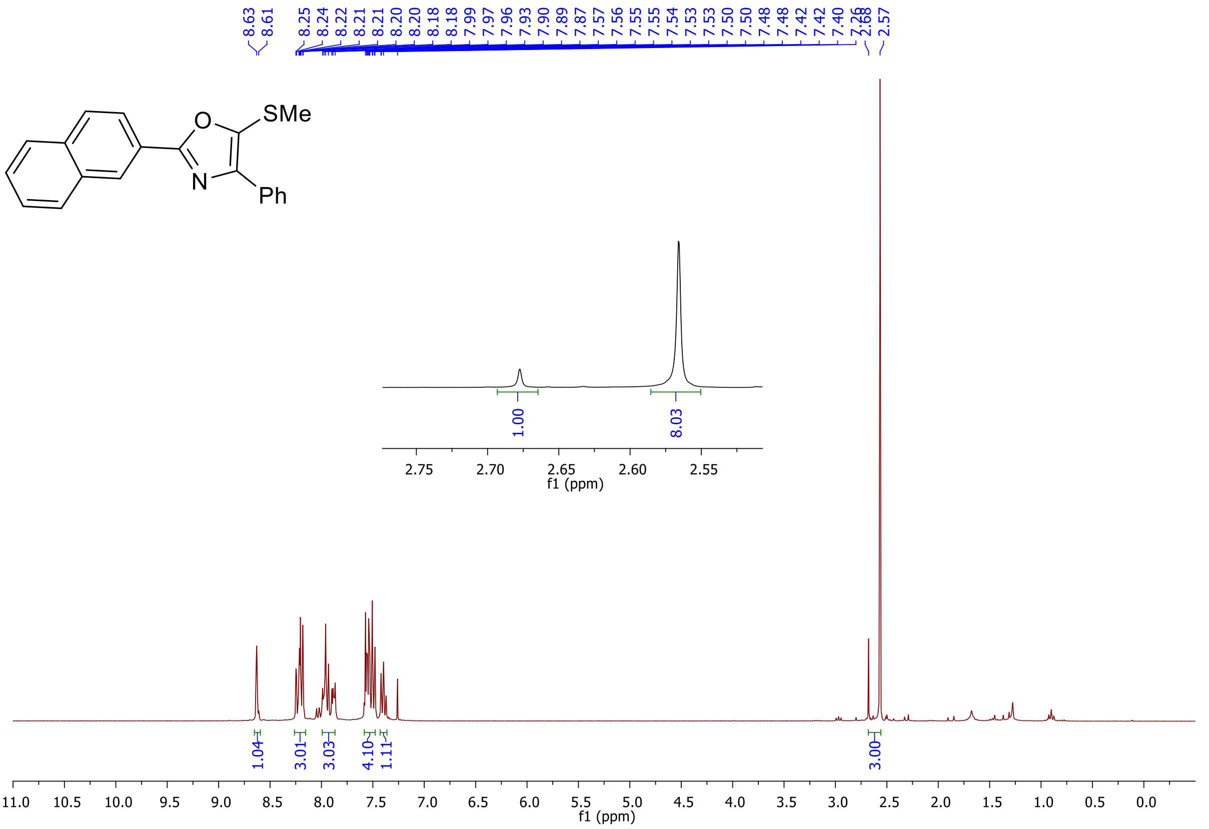

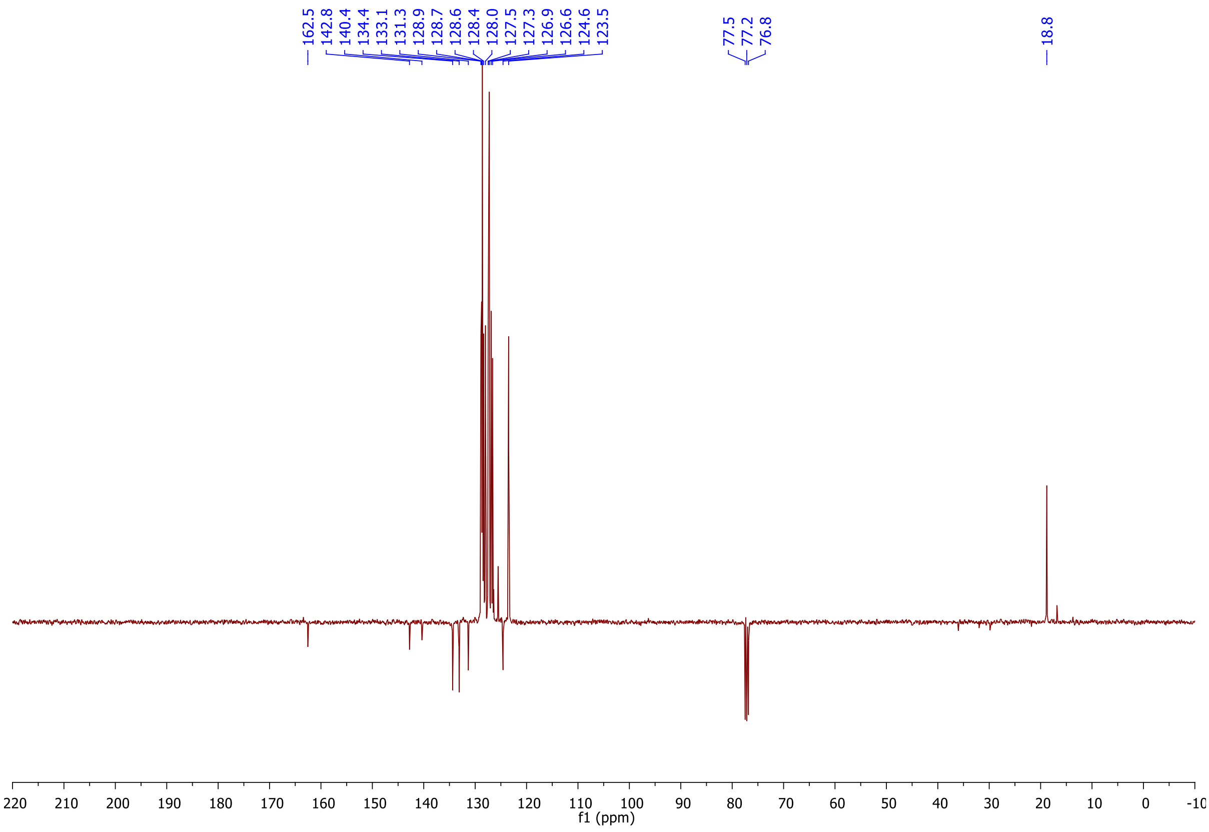

5-(Ethylthio)-4-(4-methoxyphenyl)-2-(naphthalen-2-yl)oxazole (3gc-*20.0:1*) in $CDCl_3$ ^{1}H-NMR and ^{13}C-NMR (Pendant)

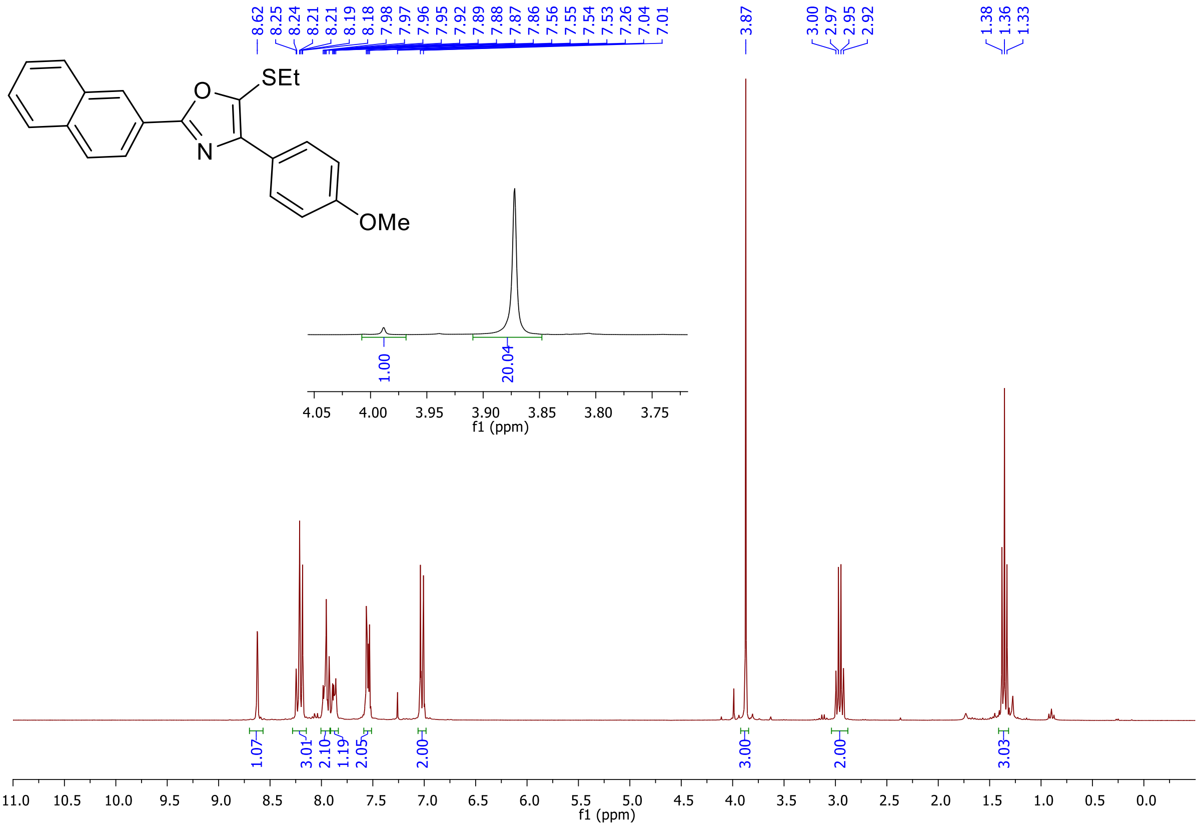

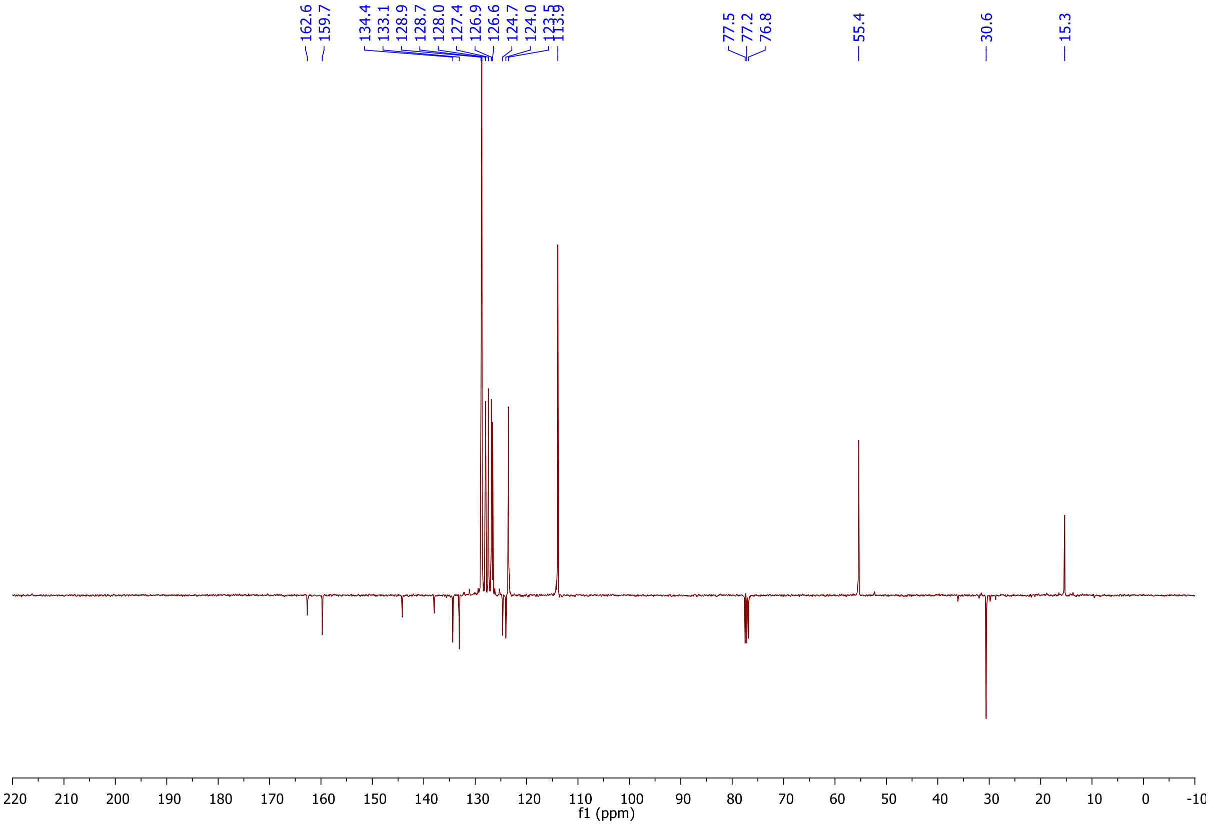

5-(Ethylthio)-2-(naphthalen-1-yl)-4-phenyloxazole (3bd-*4.7:1*) in $CDCl_3$ ^{1}H-NMR and ^{13}C-NMR (Pendant)

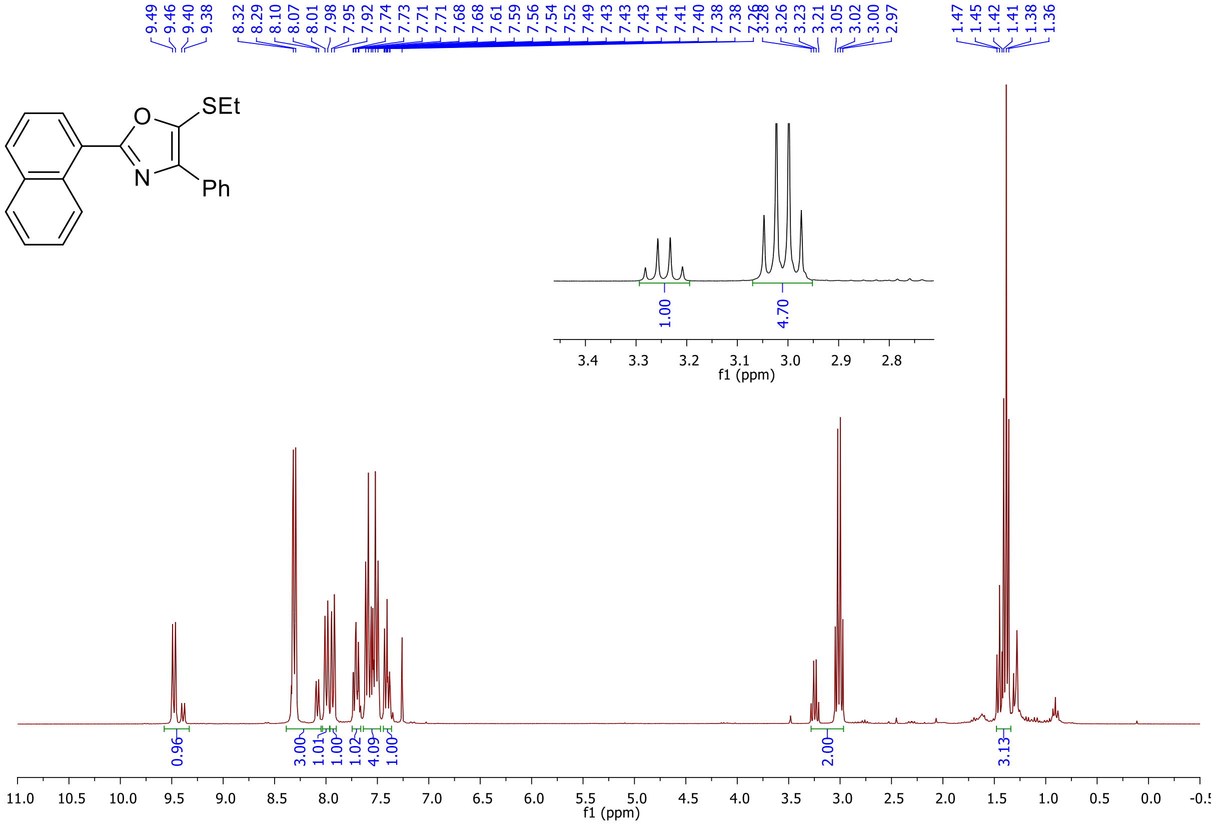

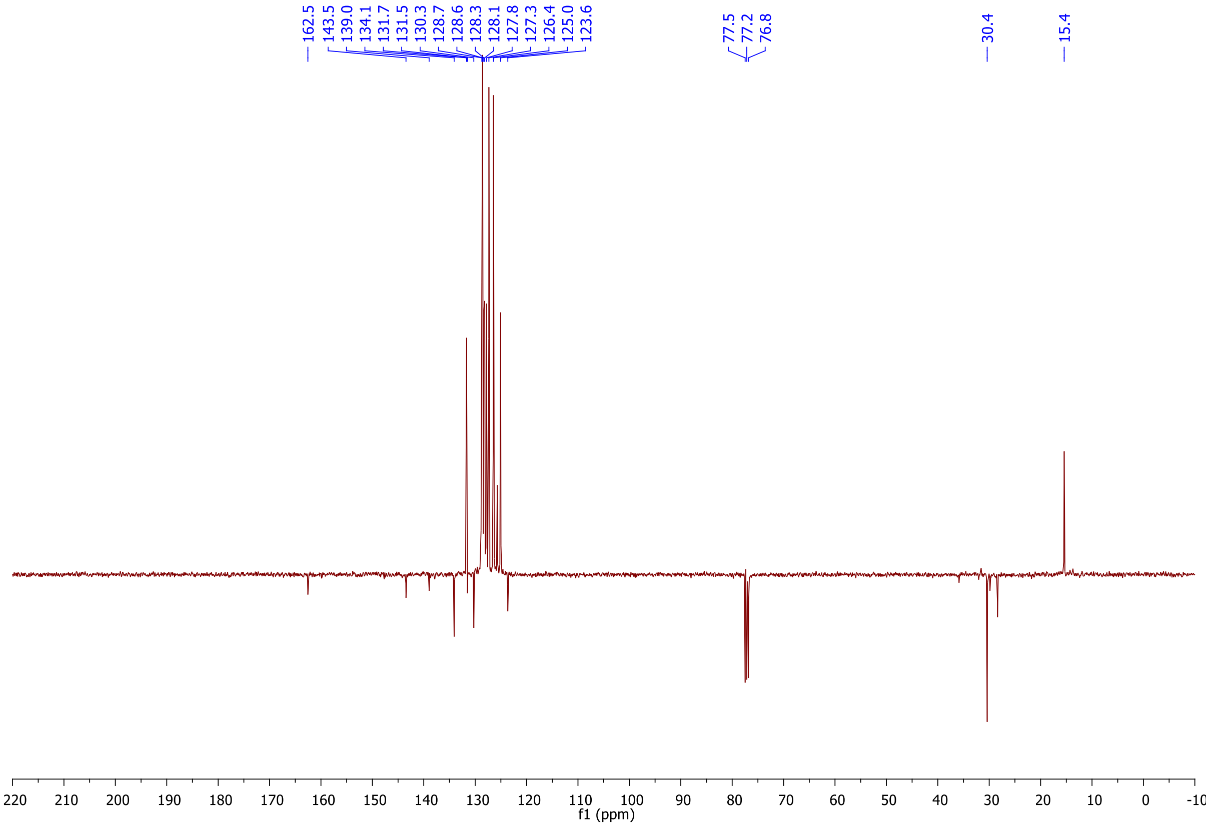

5-(Ethylthio)-4-(4-methoxyphenyl)-2-(naphthalen-1-yl)oxazole (3gd-*13.0:1*) in $CDCl_3$ ^1H-NMR and ^{13}C-NMR (Pendant)

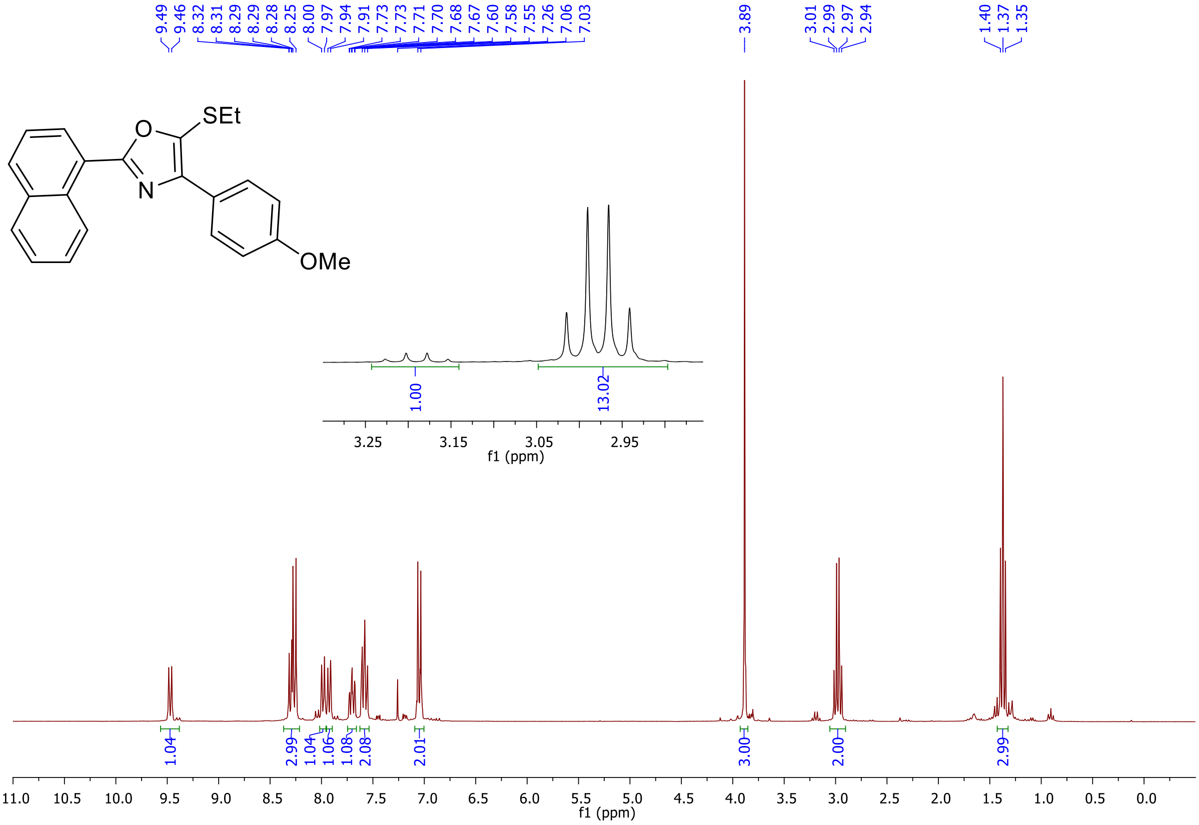

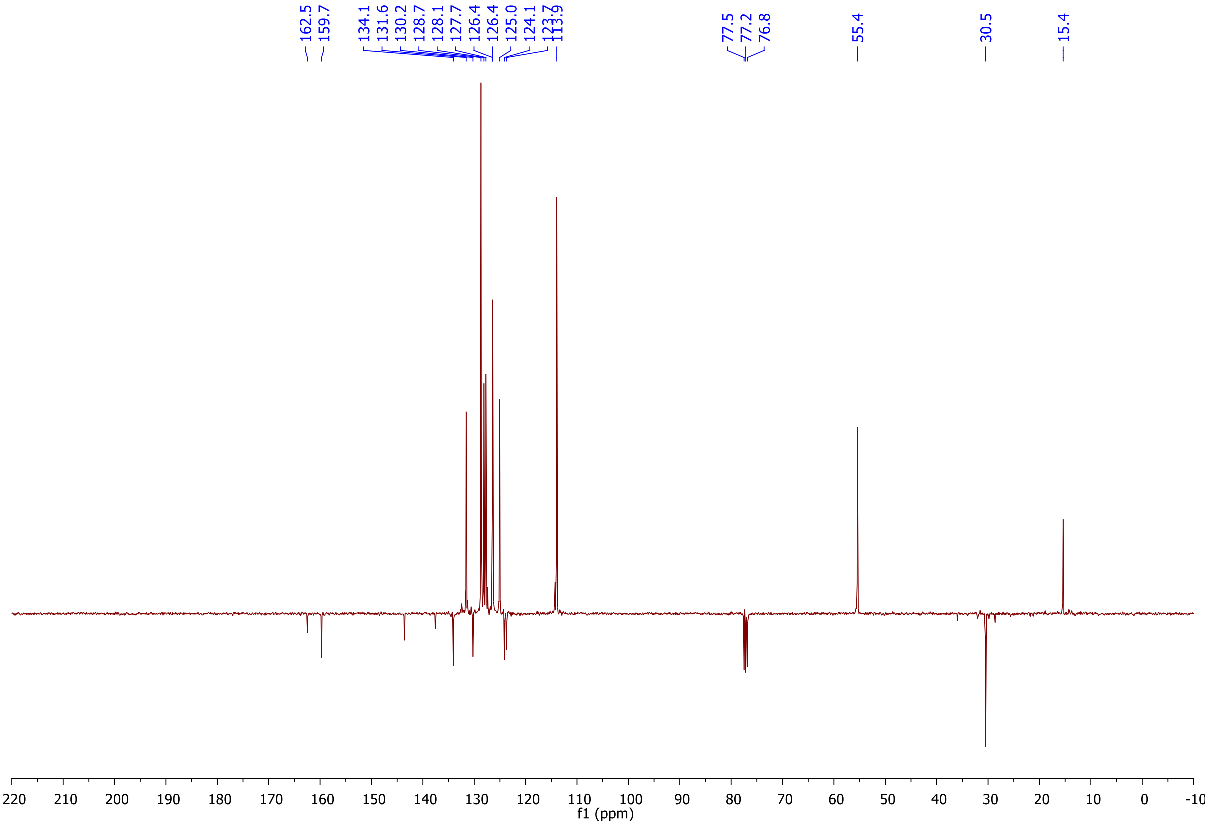

2-(Furan-2-yl)-5-(methylthio)-4-phenyloxazole (3ae-*5.7:1*) in $CDCl_3$ ^1H-NMR and ^{13}C-NMR (UDEFT)

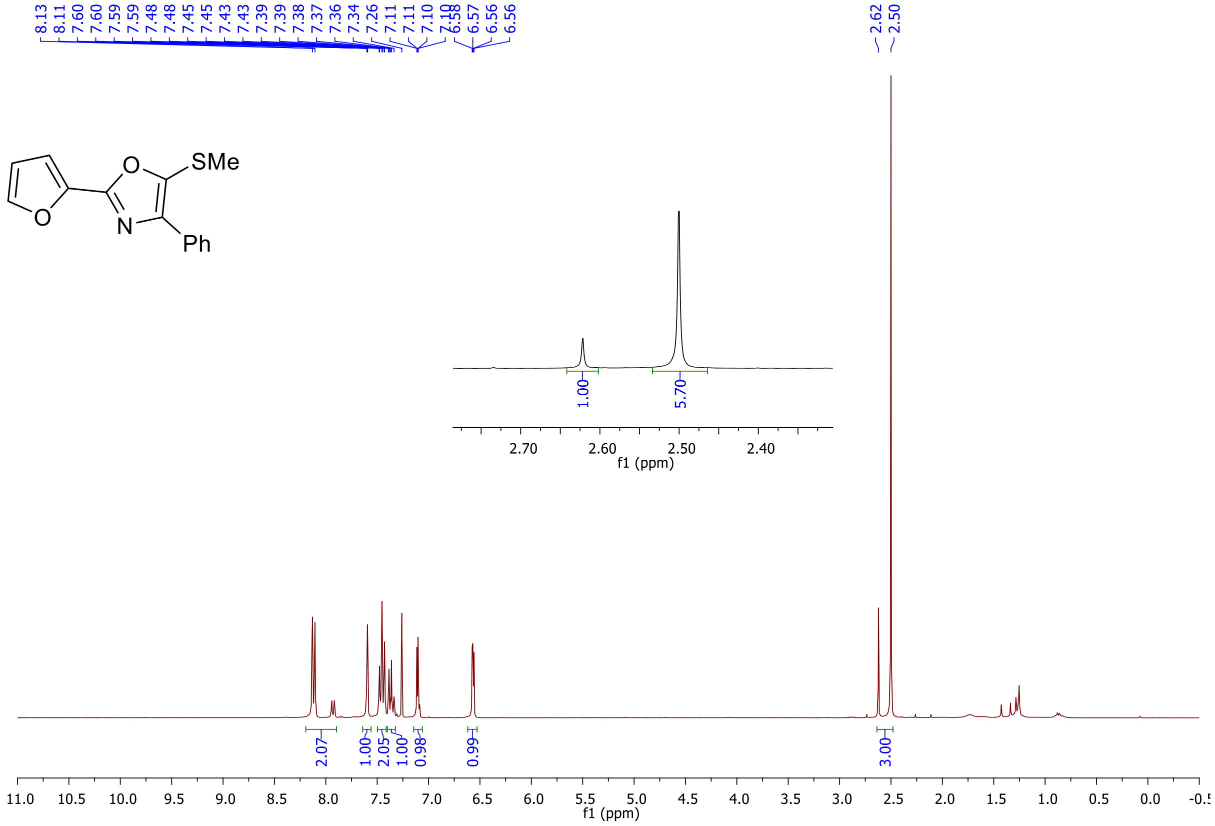

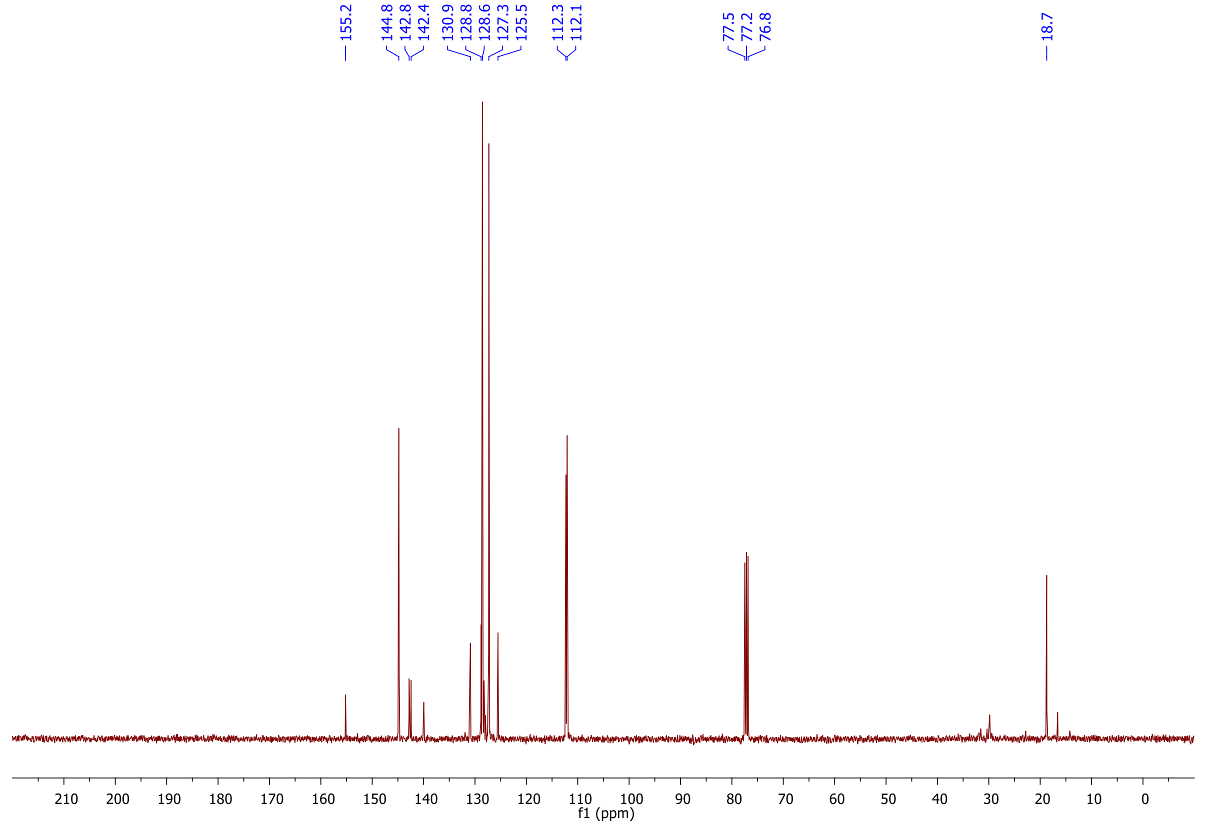

2-(Furan-2-yl)-5-(isopropylthio)-4-phenyloxazole (3ce-*3.8:1*) in $CDCl_3$ ^1H-NMR and ^{13}C-NMR (UDEFT)

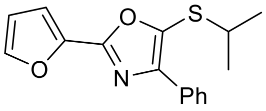

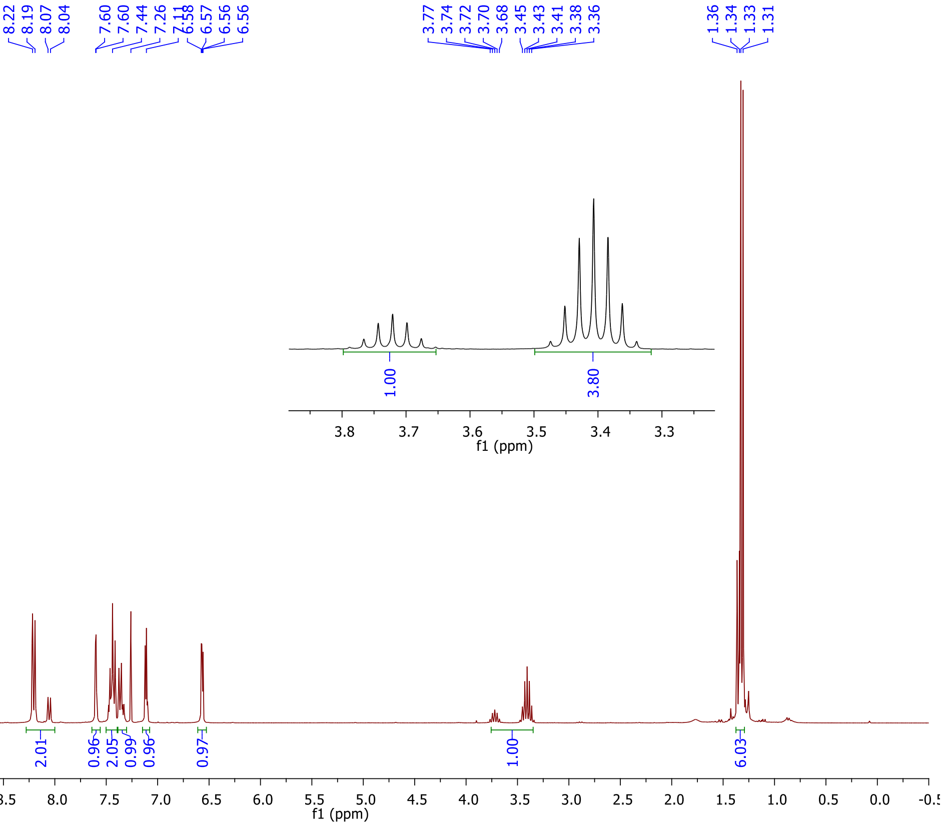

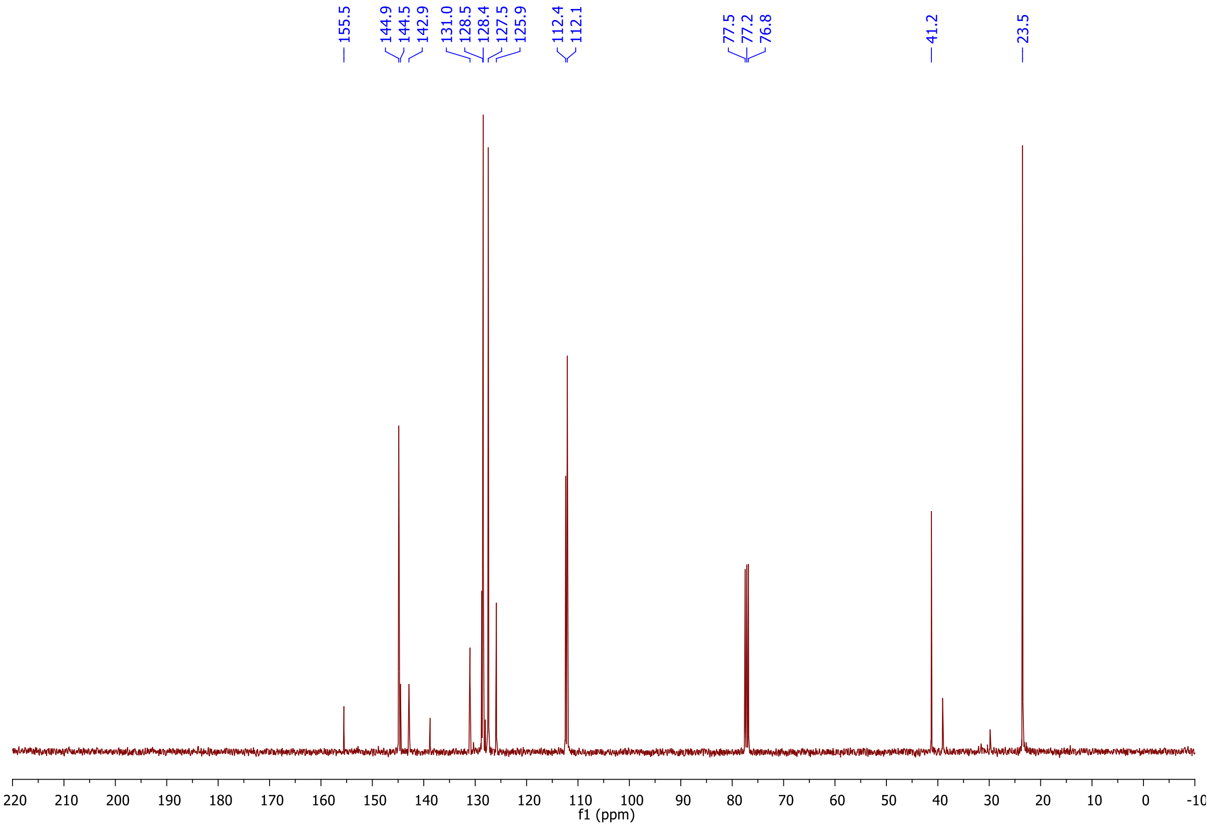

2-(Furan-2-yl)-4-(4-methoxyphenyl)-5-(methylthio)oxazole (3ie-*7.9:1*) in $CDCl_3$ ^{1}H-NMR and ^{13}C-NMR (Pendant)

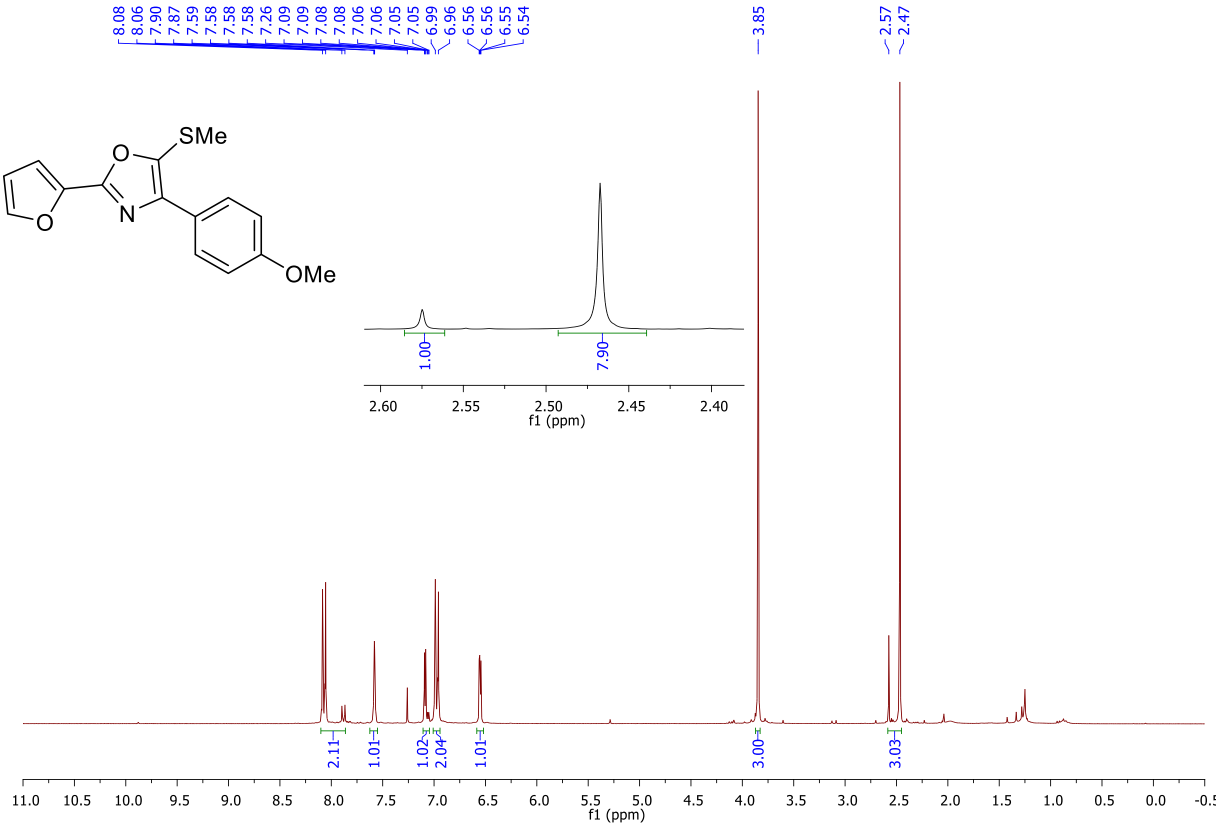

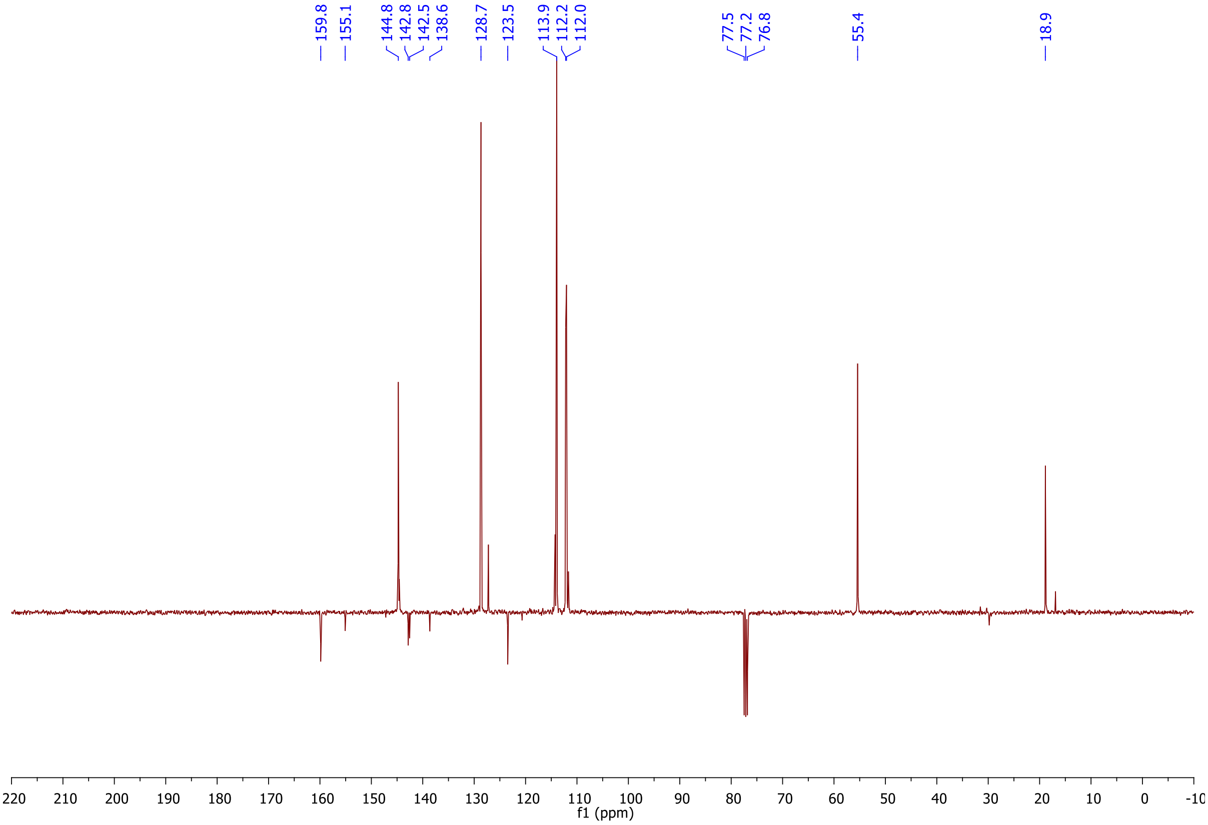

2-(Dimethoxymethyl)-5-(ethylthio)-4-(4-methoxyphenyl)oxazole (3gf-*15.2:1*) in $CDCl_3$ ^{1}H-NMR and ^{13}C-NMR (Pendant)

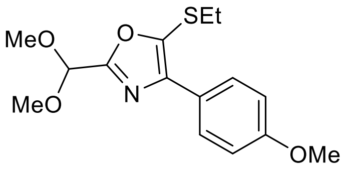

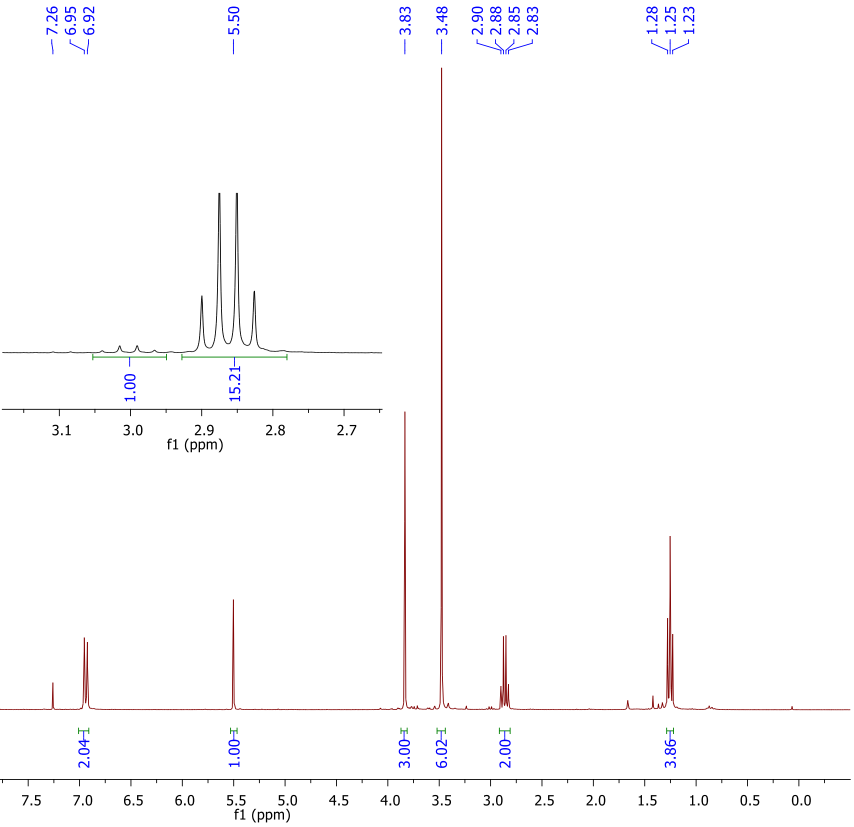

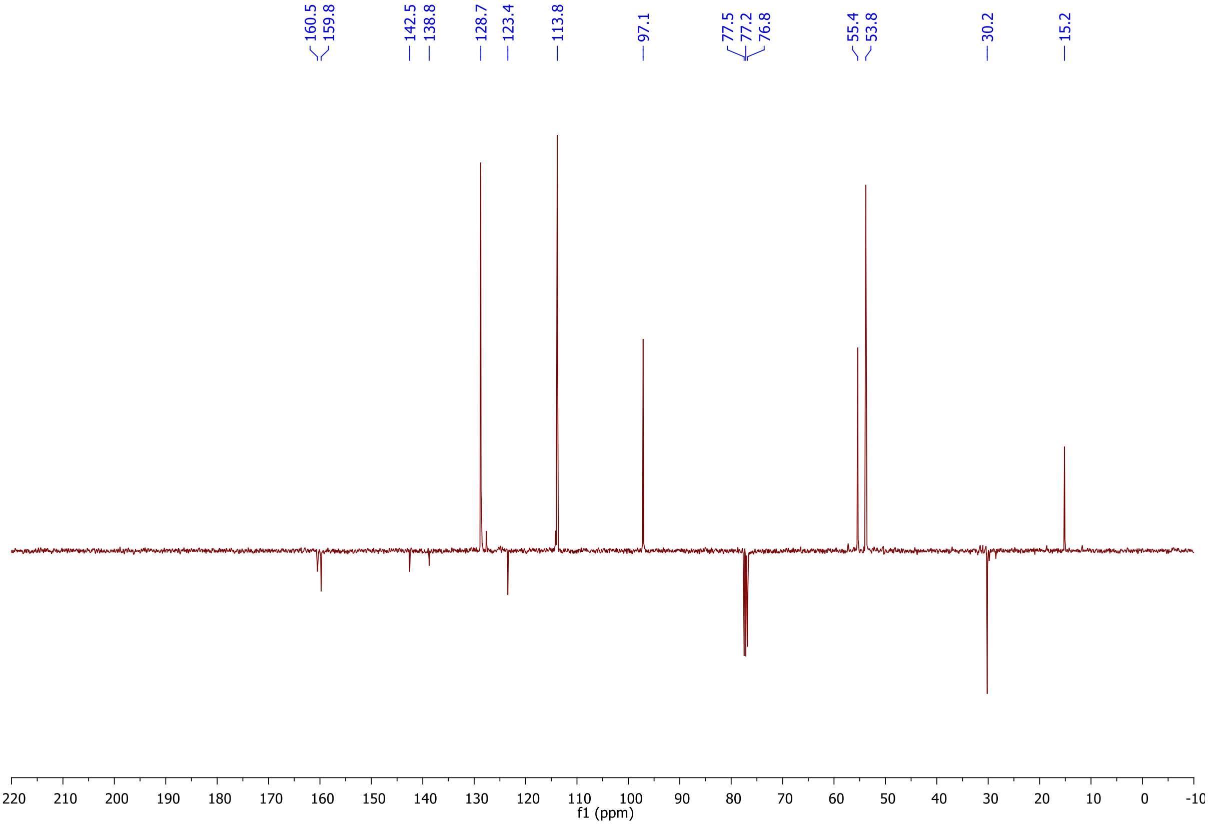

2-(Dimethoxymethyl)-4-(4-methoxyphenyl)-5-(phenylthio)oxazole (3hf-*11.4:1*) in $CDCl_3$ ^{1}H-NMR and ^{13}C-NMR (Pendant)

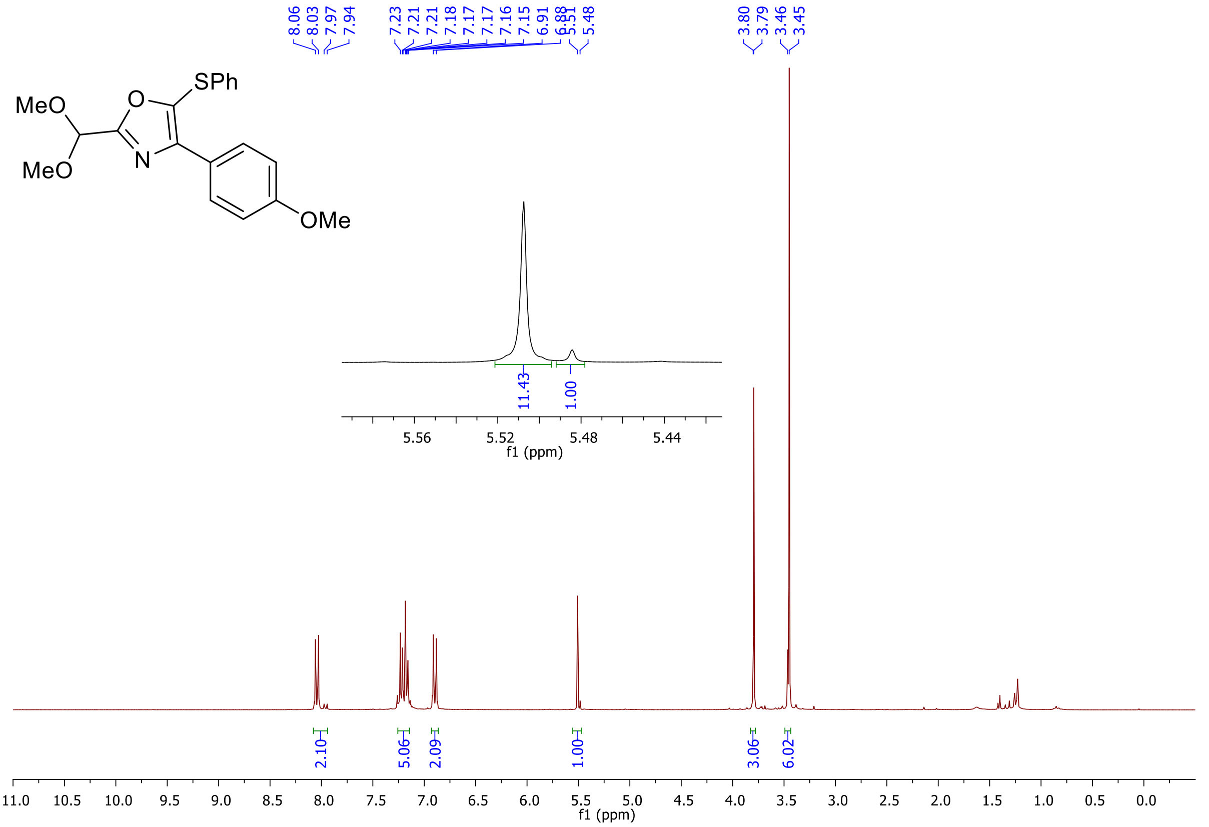

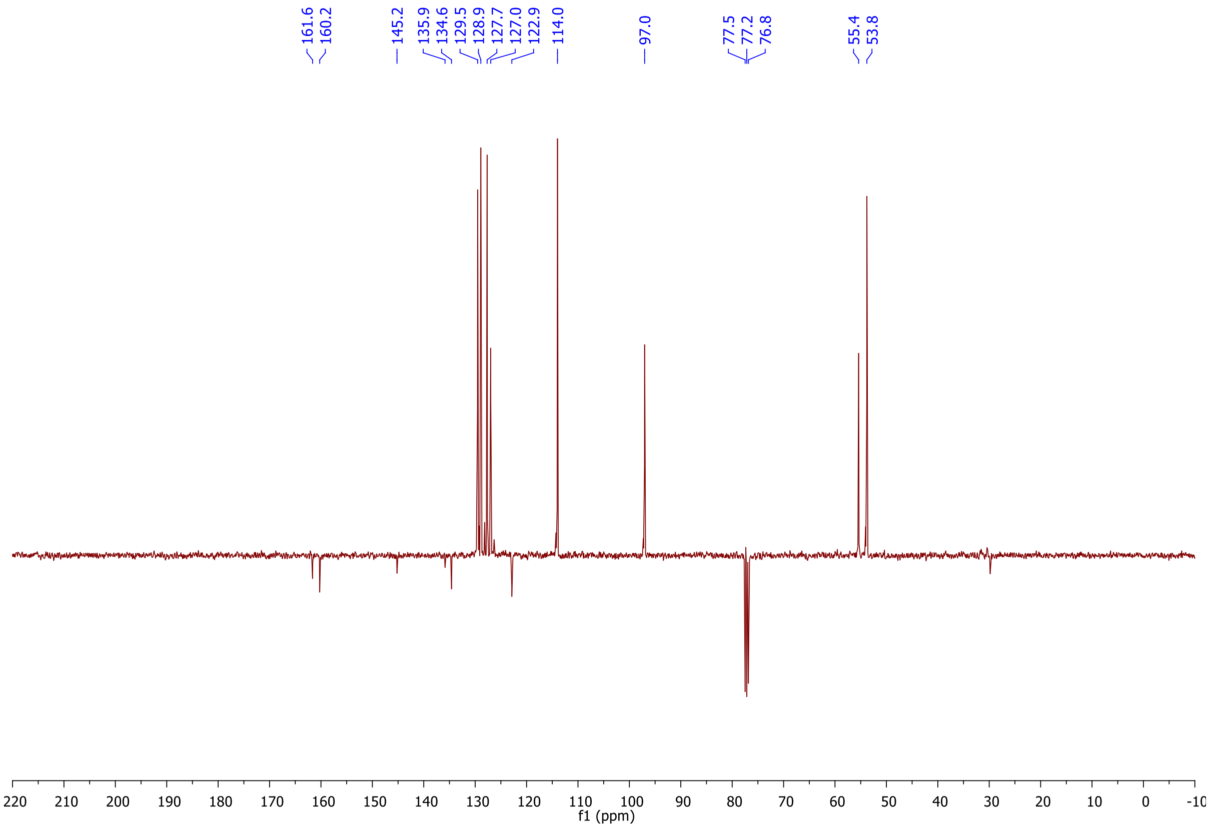

5-(Ethylthio)-2-(propan-2-ol)-(4-methoxyphenyl)oxazole (3gg-*28.5:1*) in $CDCl_3$ ^{1}H-NMR and ^{13}C-NMR (UDEFT)

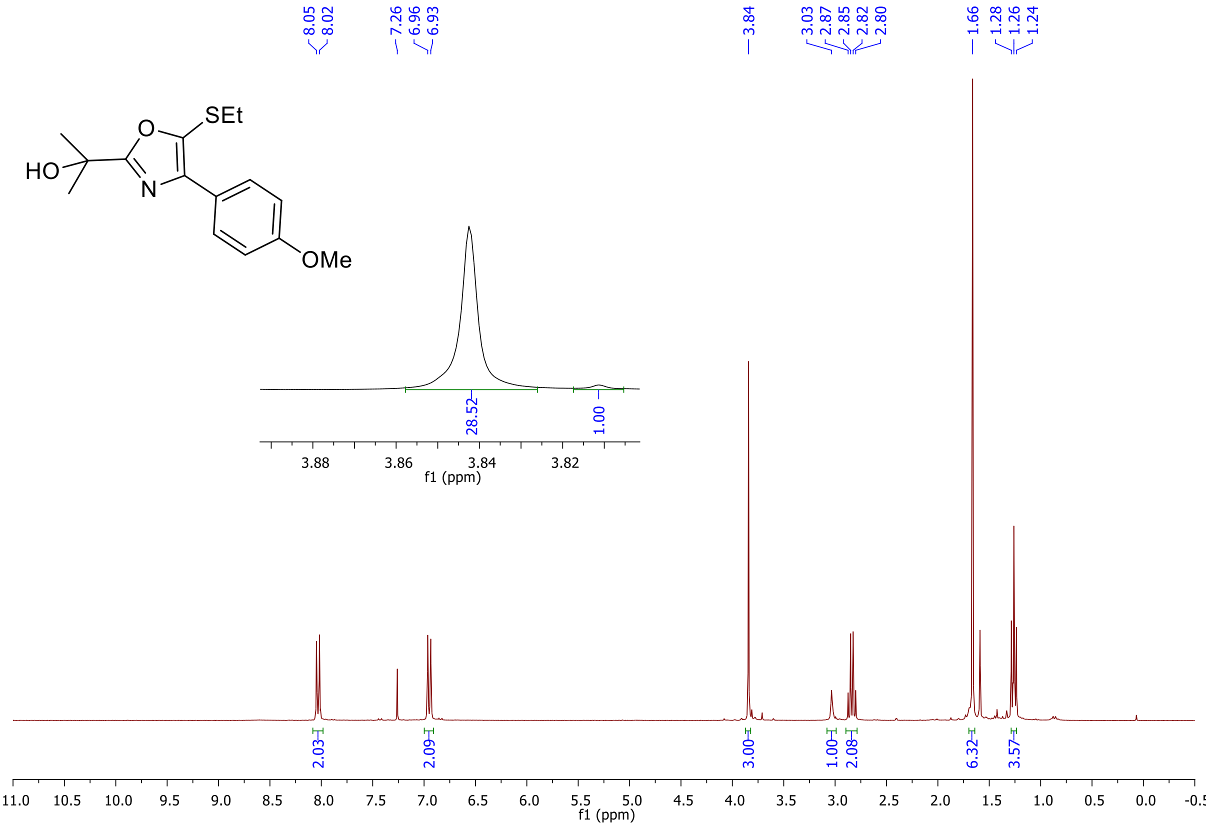

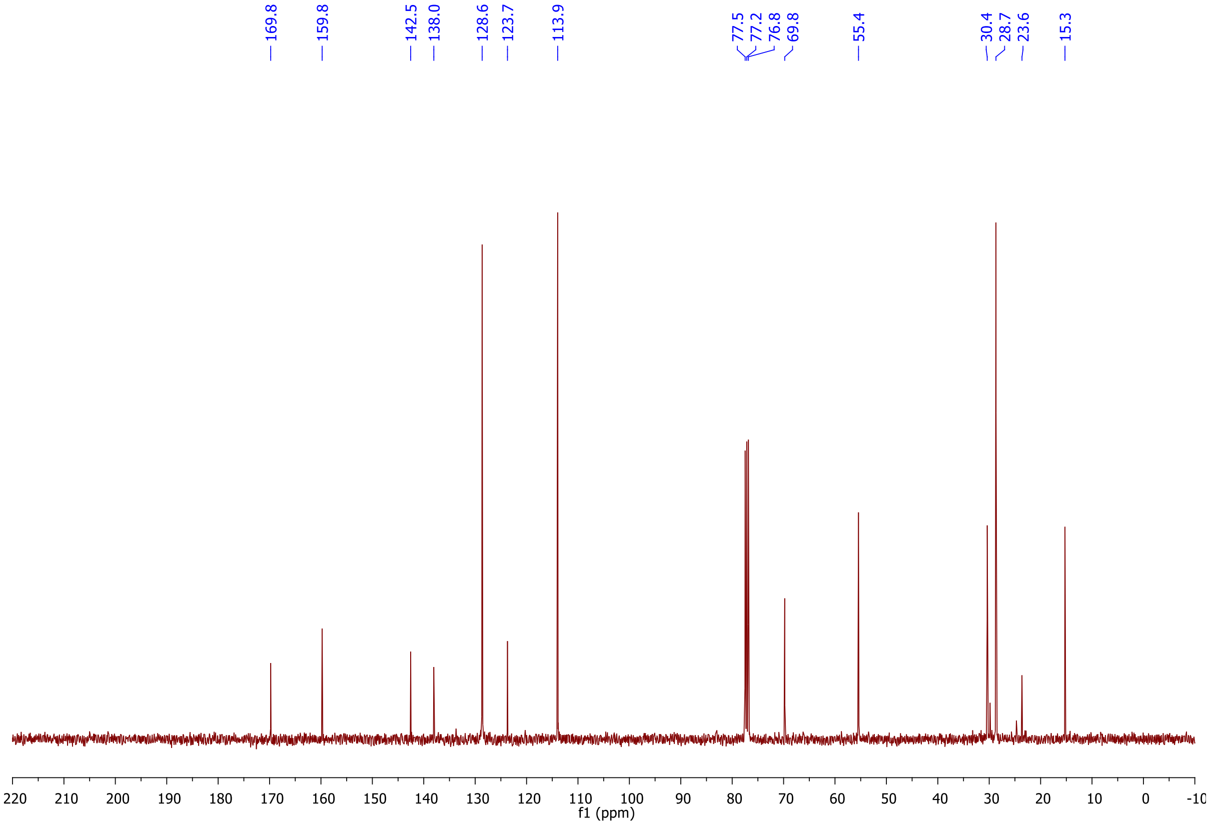

4-Butyl-5-(methylthio)-2-phenyloxazole (3ja-*3.2:1*) in $CDCl_3$ ^{1}H-NMR and ^{13}C-NMR (UDEFT)

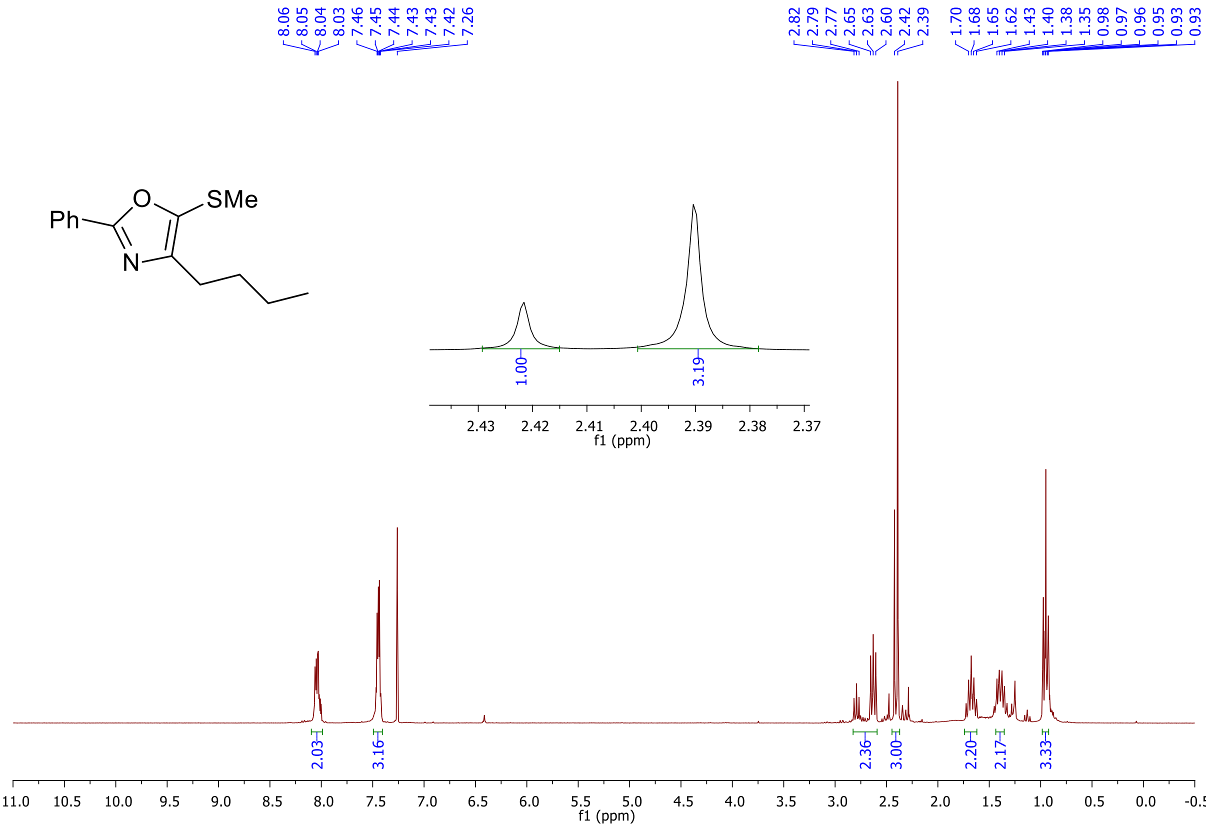

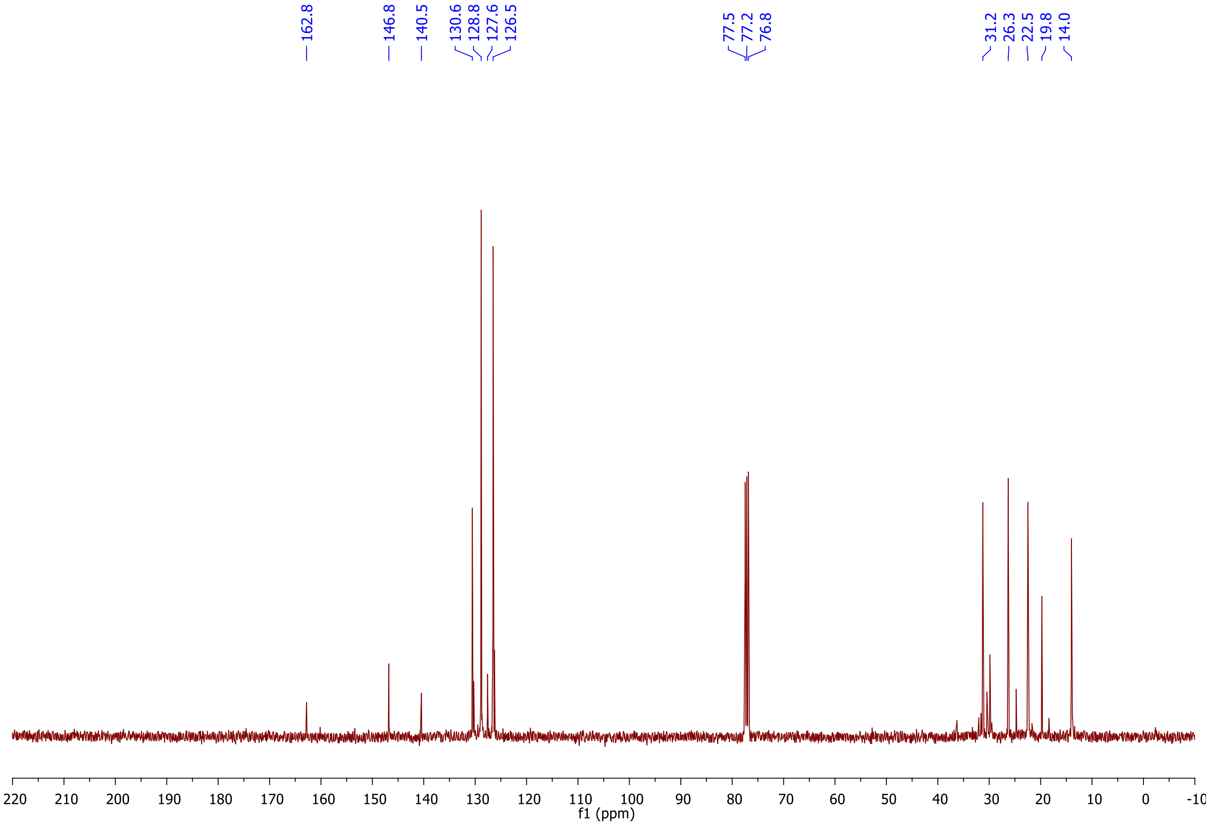

4-Butyl-5-(methylthio)-2-(naphthalen-2-yl)oxazole (3jc-*3.5:1*) in $CDCl_3$ ^{1}H-NMR and ^{13}C-NMR (UDEFT)

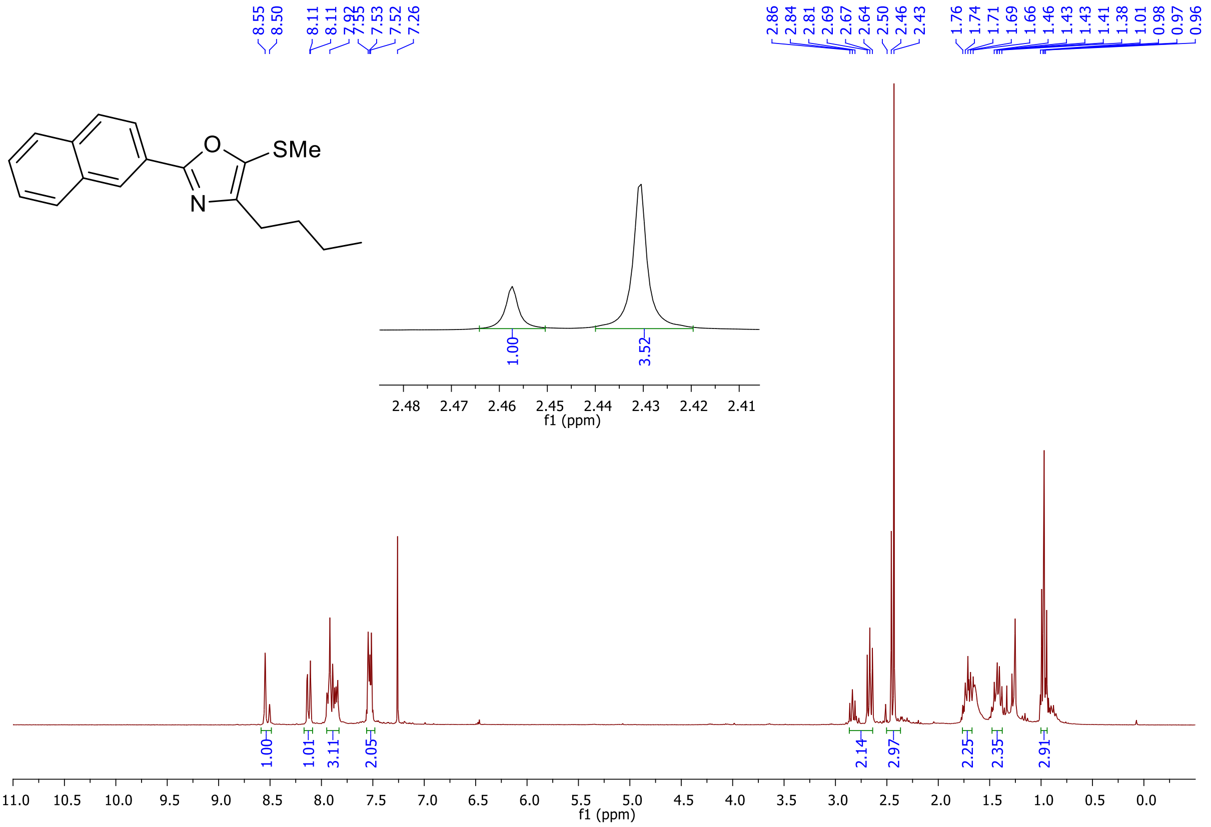

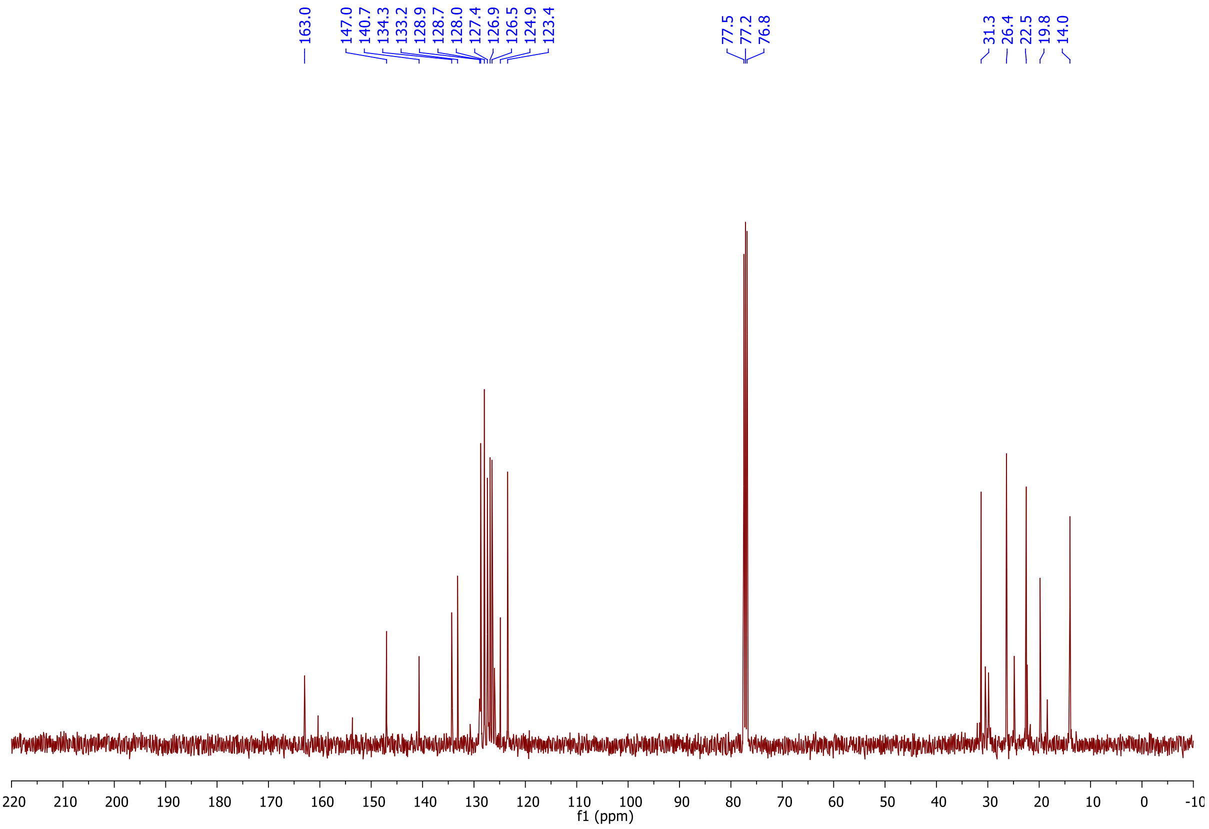

4-(3,4-Dimethoxyphenyl)-5-(ethylthio)-2-phenyloxazole (3ka-*13.5:1*) in $CDCl_3$ ^1H-NMR and ^{13}C-NMR (Pendant)

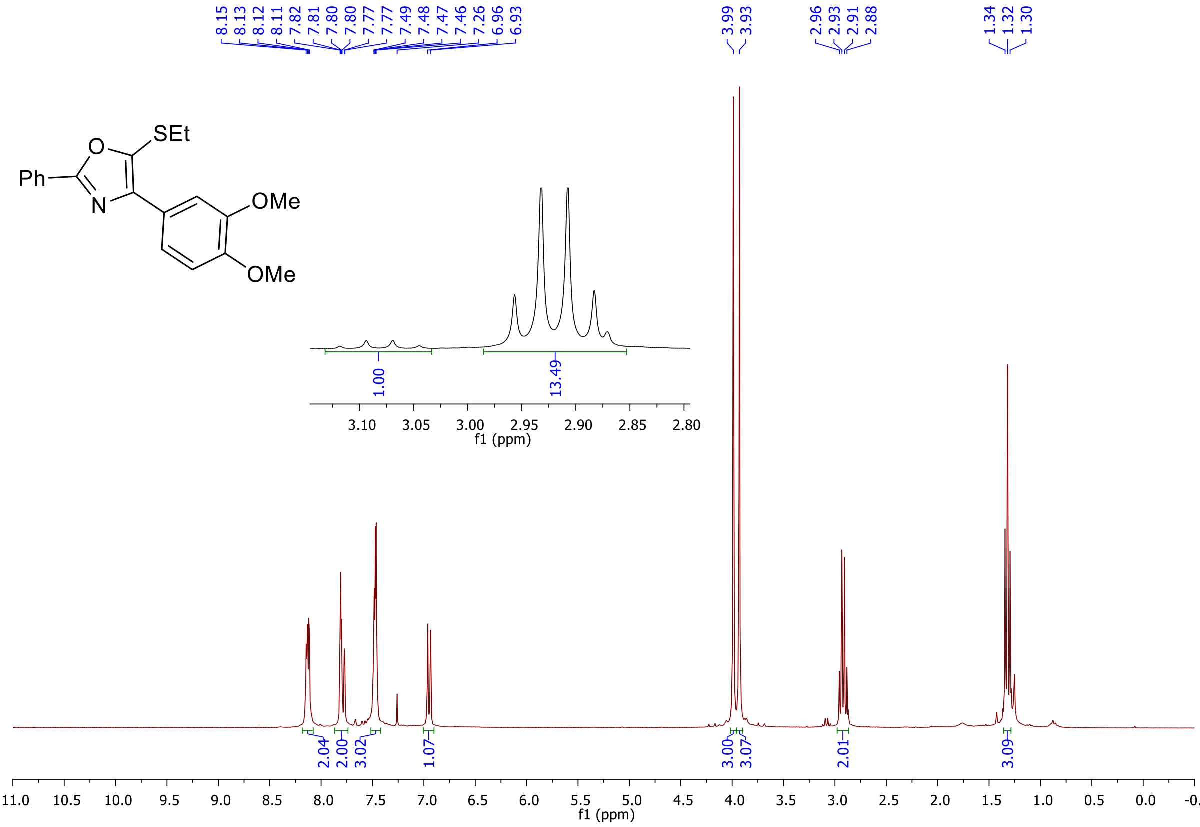

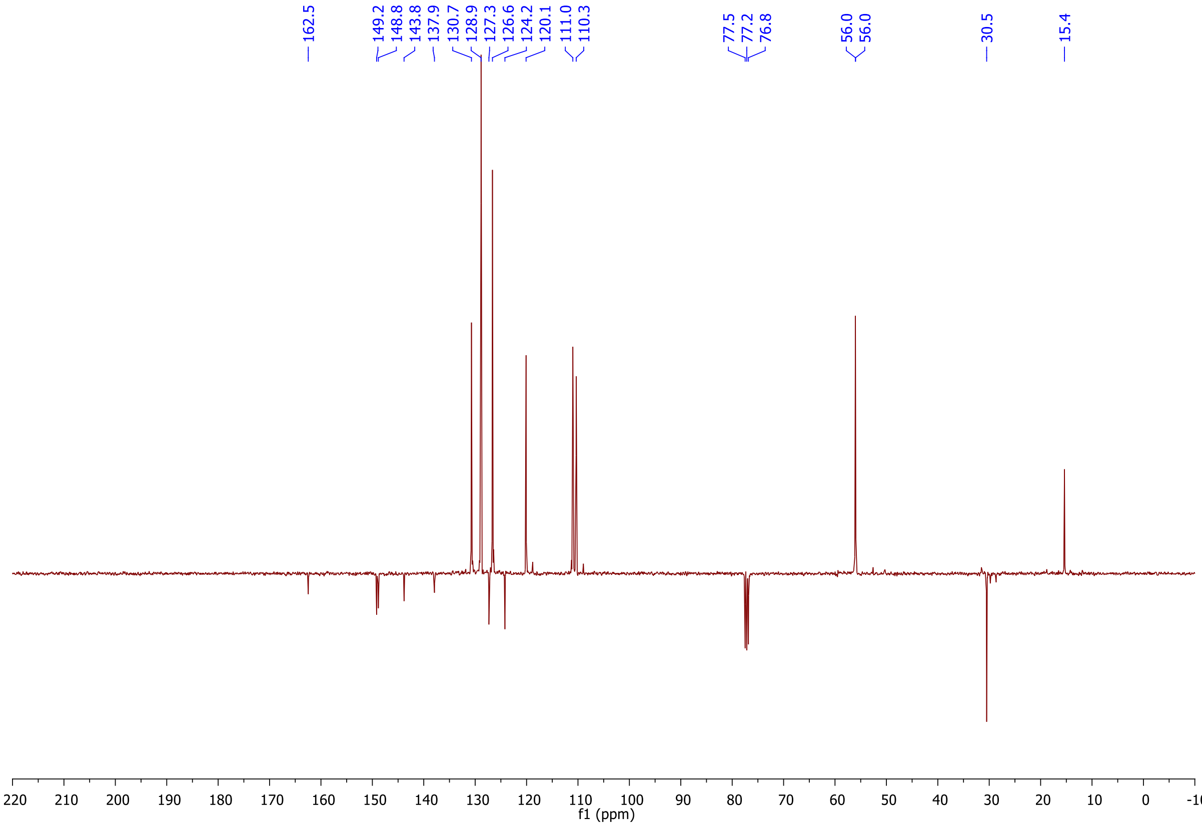

4-(3,4-Dimethoxyphenyl)-5-(ethylthio)-2-(naphthalen-2-yl)oxazole (3kc-*16.1:1*) in $CDCl_3$ ^{1}H-NMR and ^{13}C-NMR (Pendant)

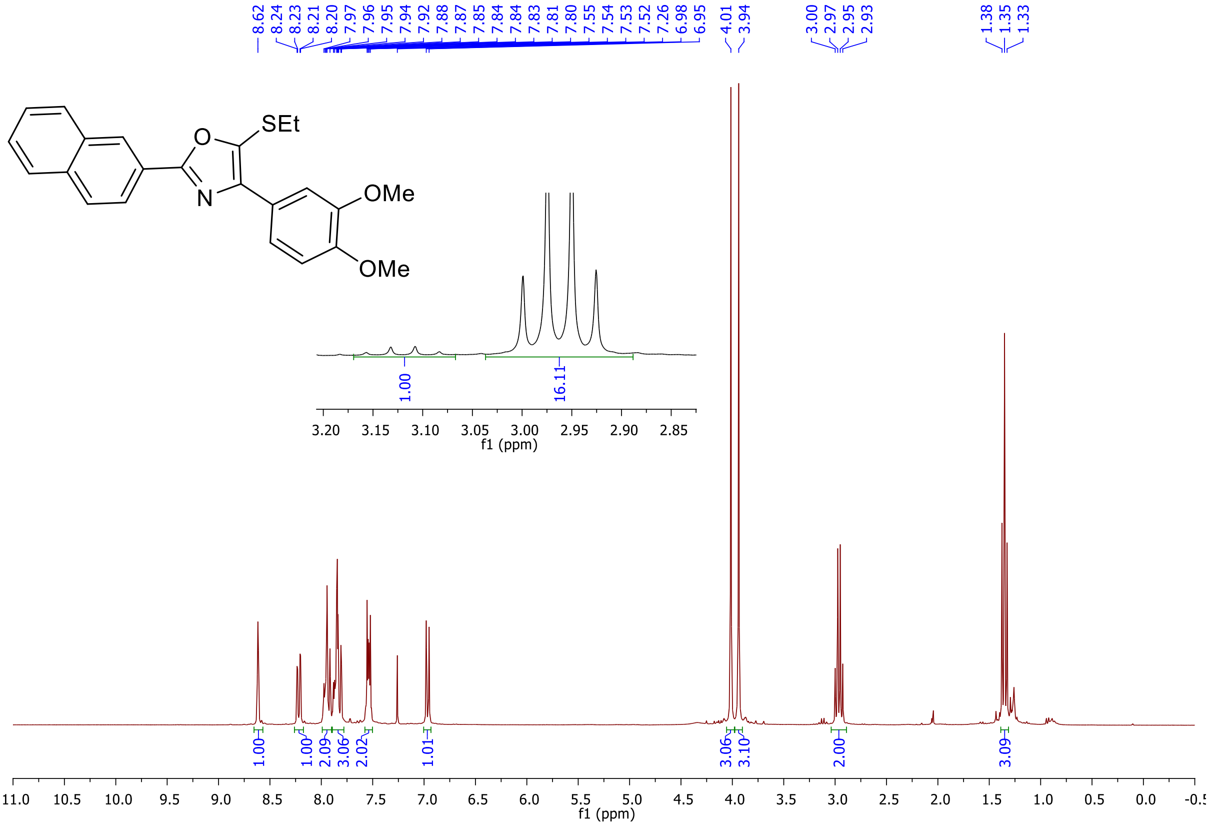

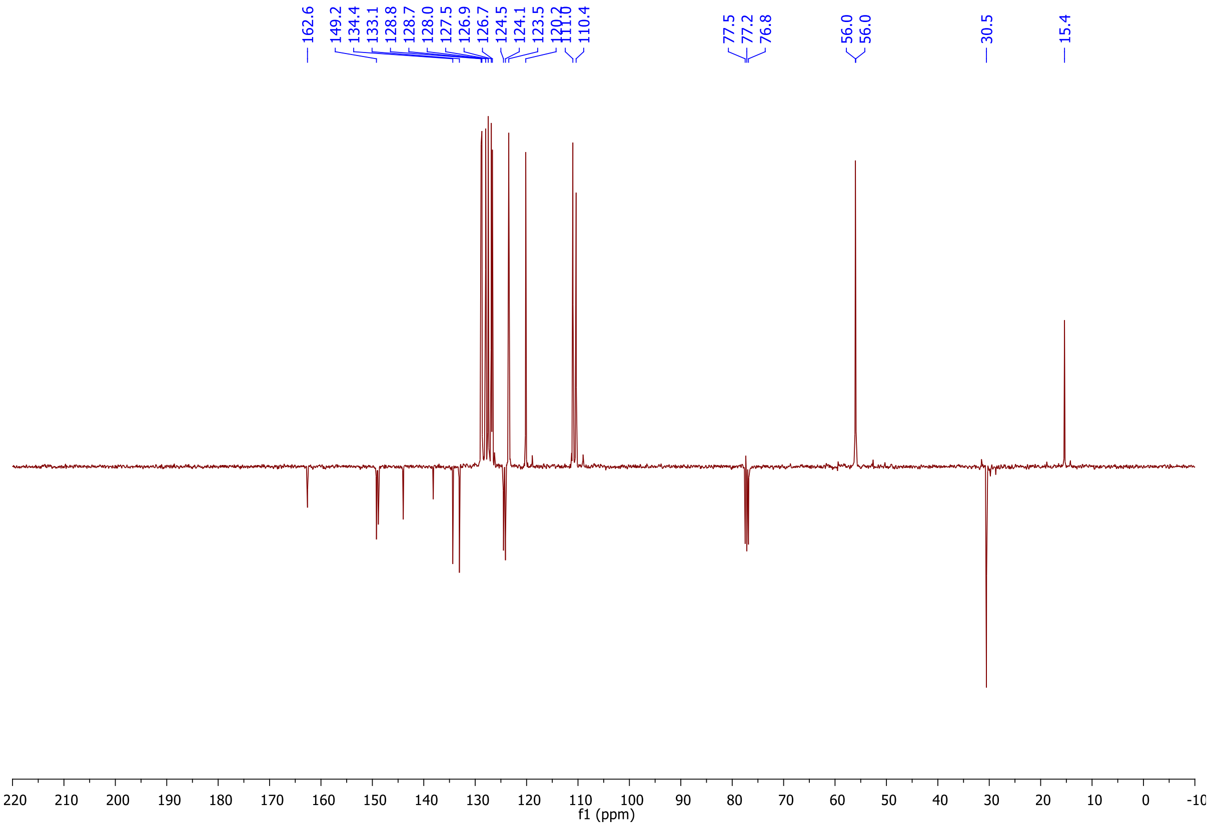

***tert*-Butyl5-(ethylthio)-4-[(4-methoxyphenyl)oxazol-2-yl)methyl]carbamate (3gh-*23.0:1*)** in $CDCl_3$
^{1}H-NMR and ^{13}C-NMR (Pendant)

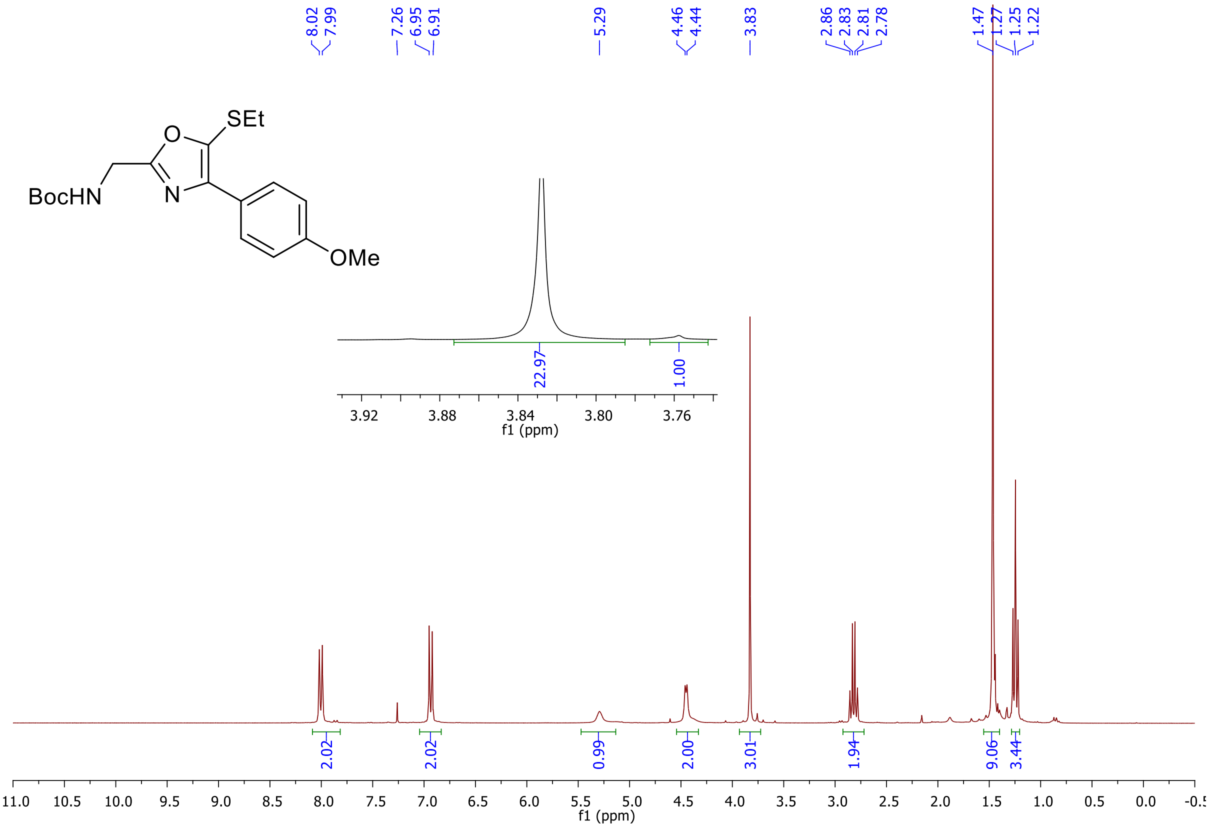

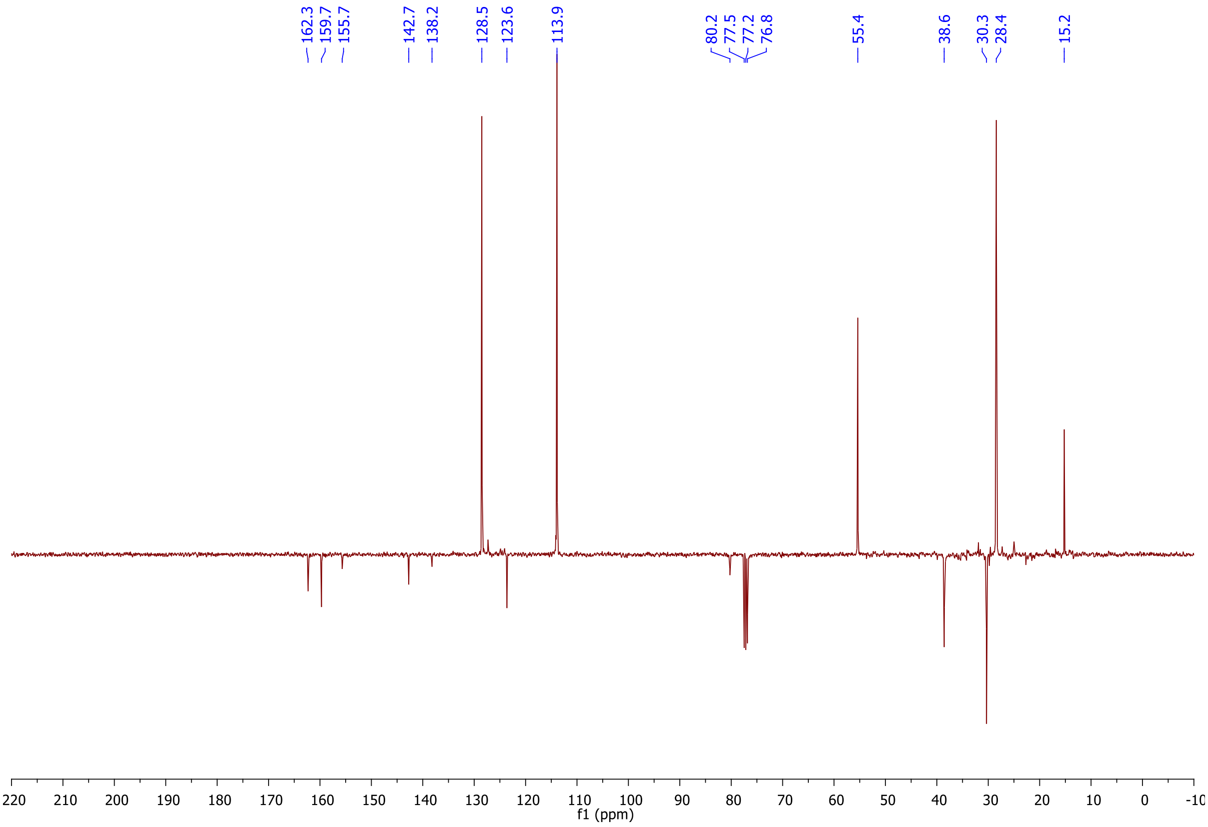

***tert*-Butyl [(4-(4-methoxyphenyl)-5-(phenylthio)oxazol-2-yl)methyl]carbamate (3hh-*10.3:1*)** in $CDCl_3$
^{1}H-NMR and ^{13}C-NMR (Pendant)

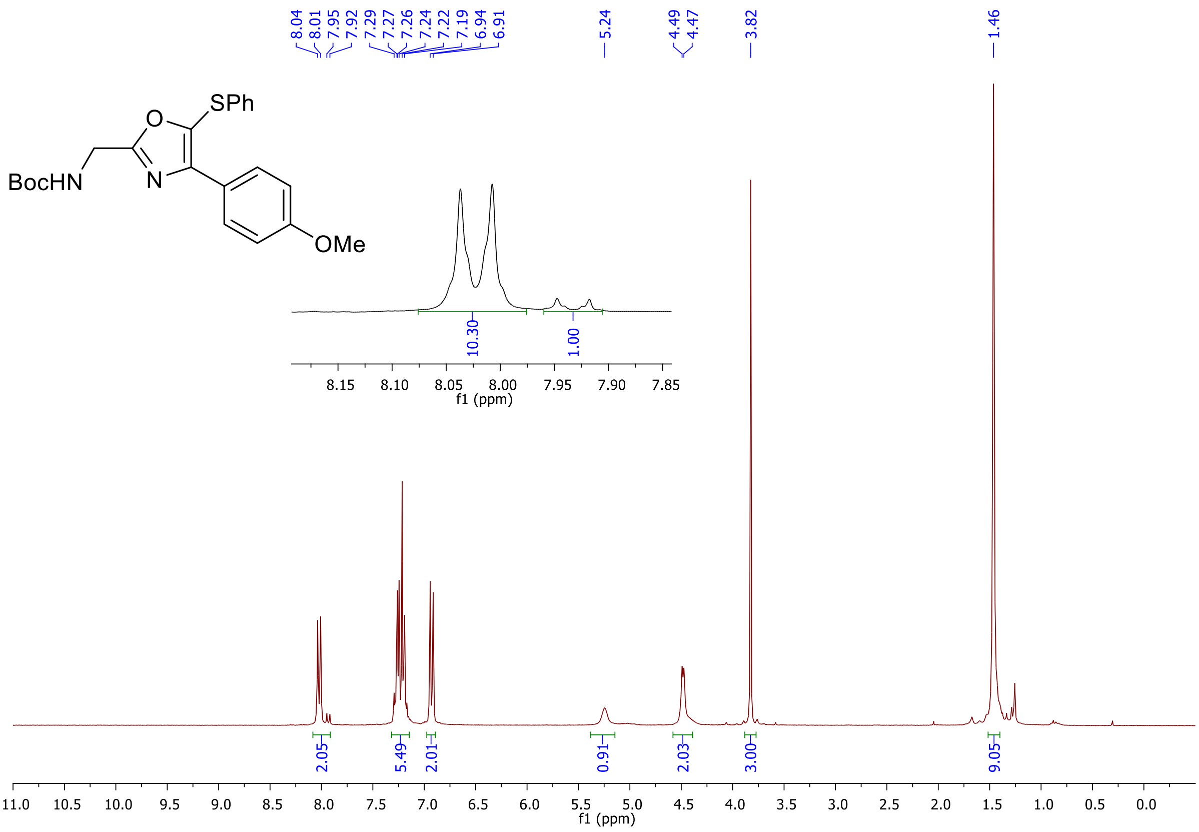

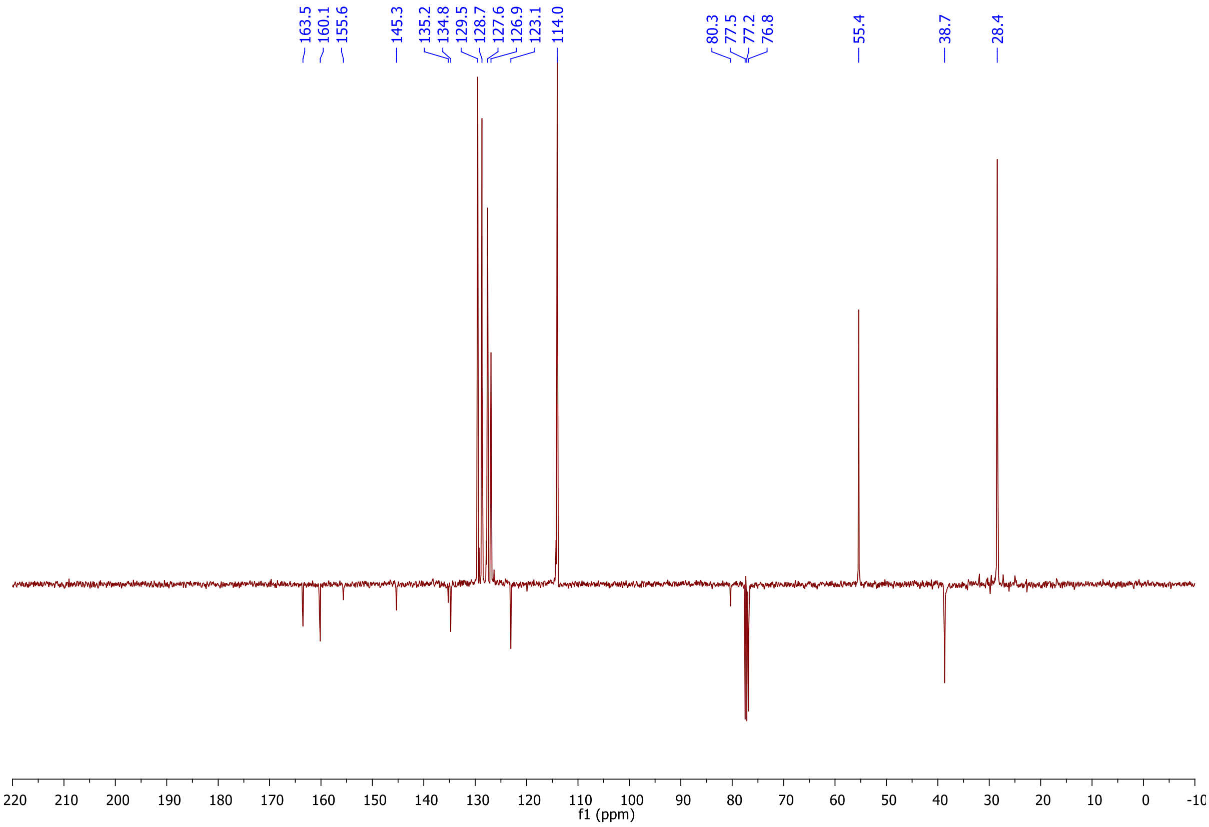

tert-Butyl ((4-(4-methoxyphenyl)-5-(methylthio)oxazol-2-yl)methyl)carbamate (3ih) in $CDCl_3$ ^{1}H-NMR and ^{13}C-NMR (UDEFT)

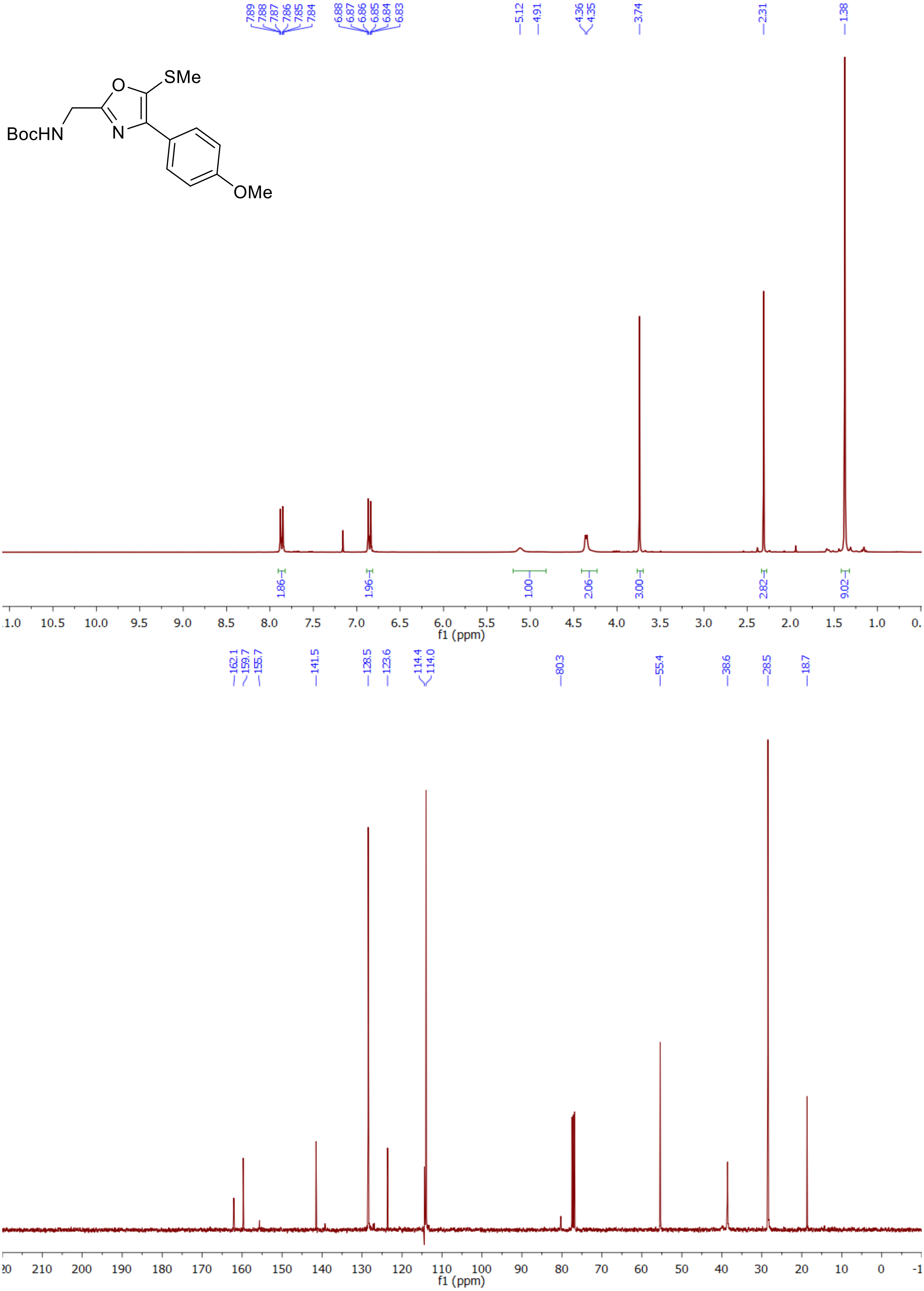

***tert*-Butyl [(4-(3,4-dimethoxyphenyl)-5-(ethylthio)oxazol-2-yl)methyl]carbamate (3kh-*50.0:1*)** in $CDCl_3$ ^1H-NMR and ^{13}C-NMR (Pendant)

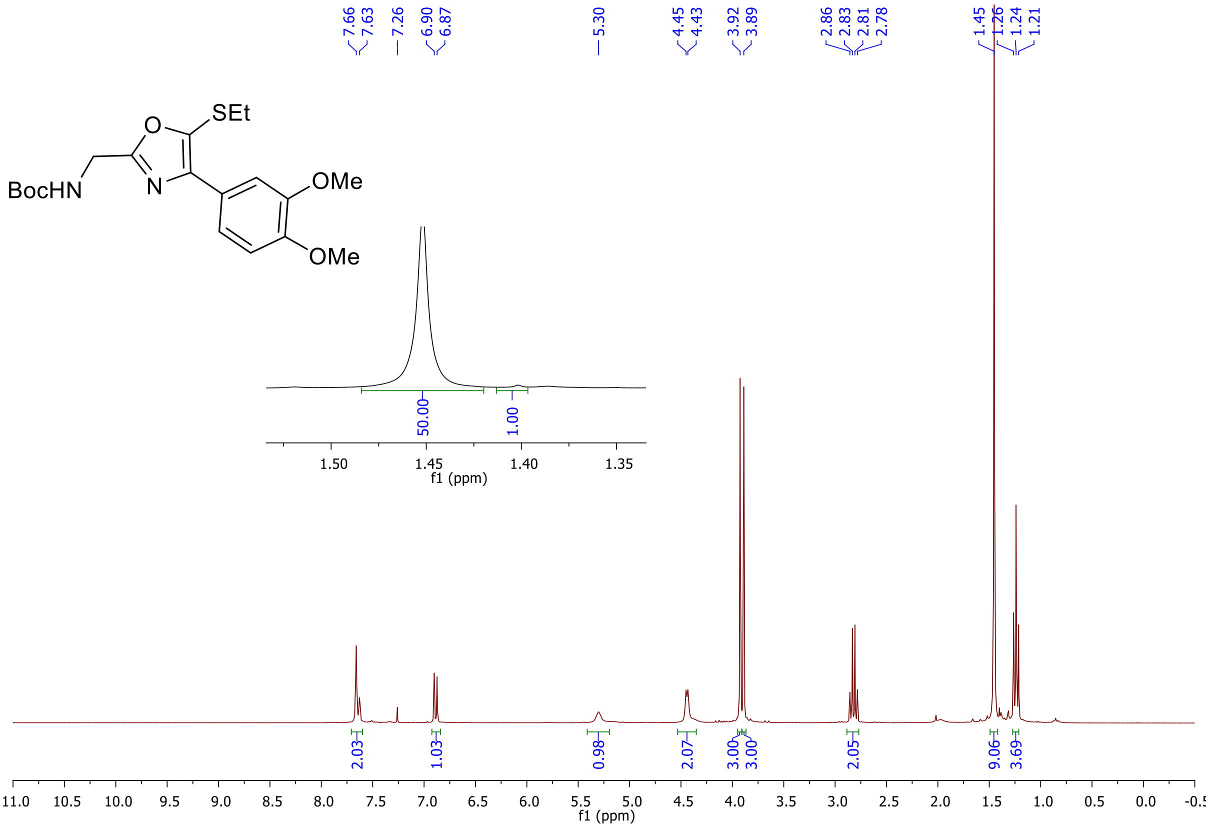

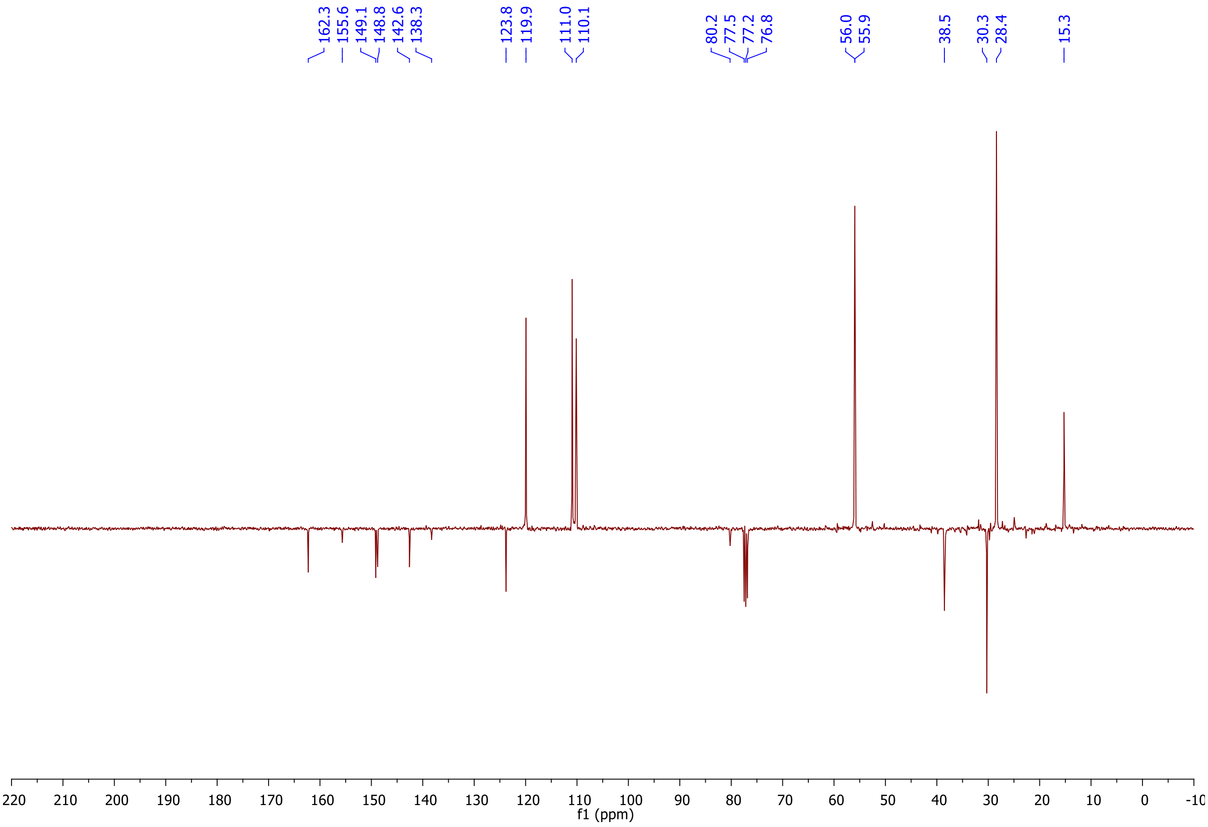

***tert*-Butyl(*S*)-1-[(5-(ethylthio)-4-(4-methoxyphenyl)oxazol-2-yl]-2-phenylethylcarbam-ate (3gk-*19.1:1*)** in $CDCl_3$ ^{1}H-NMR and ^{13}C-NMR (Pendant)

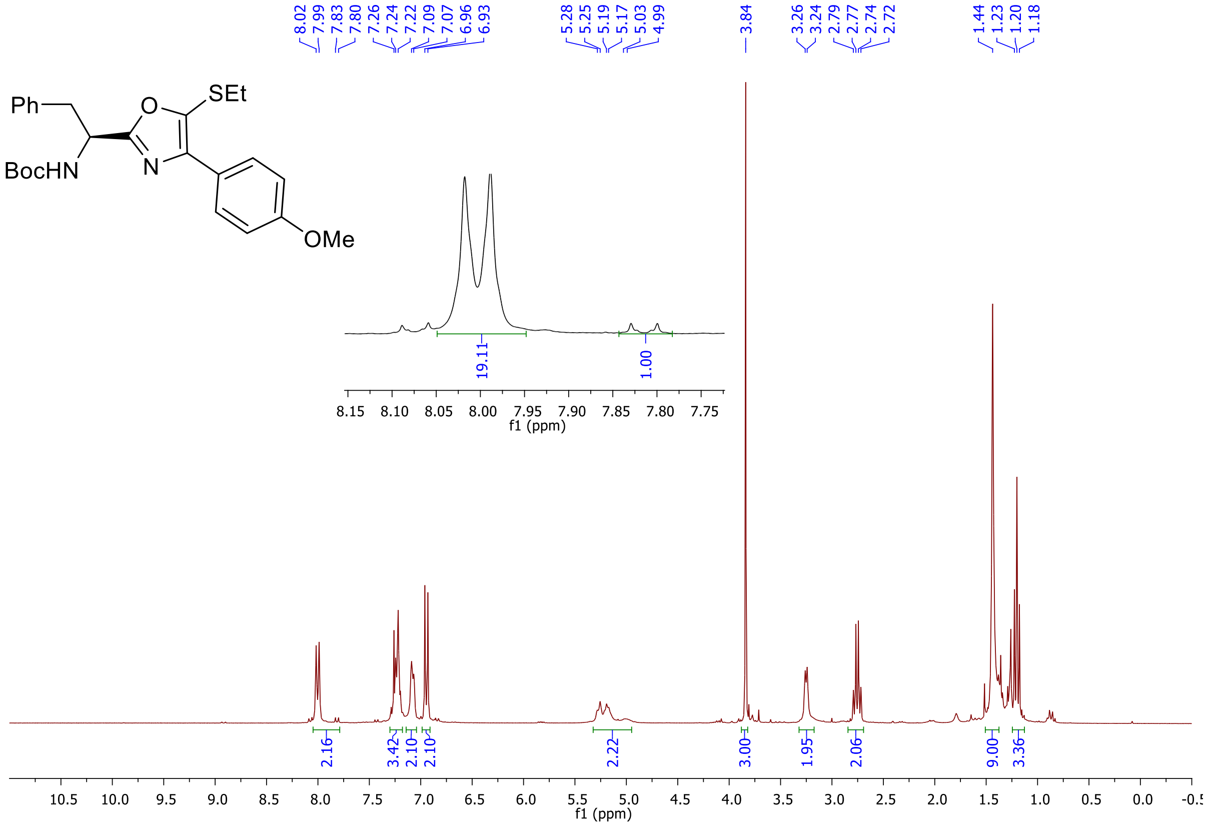

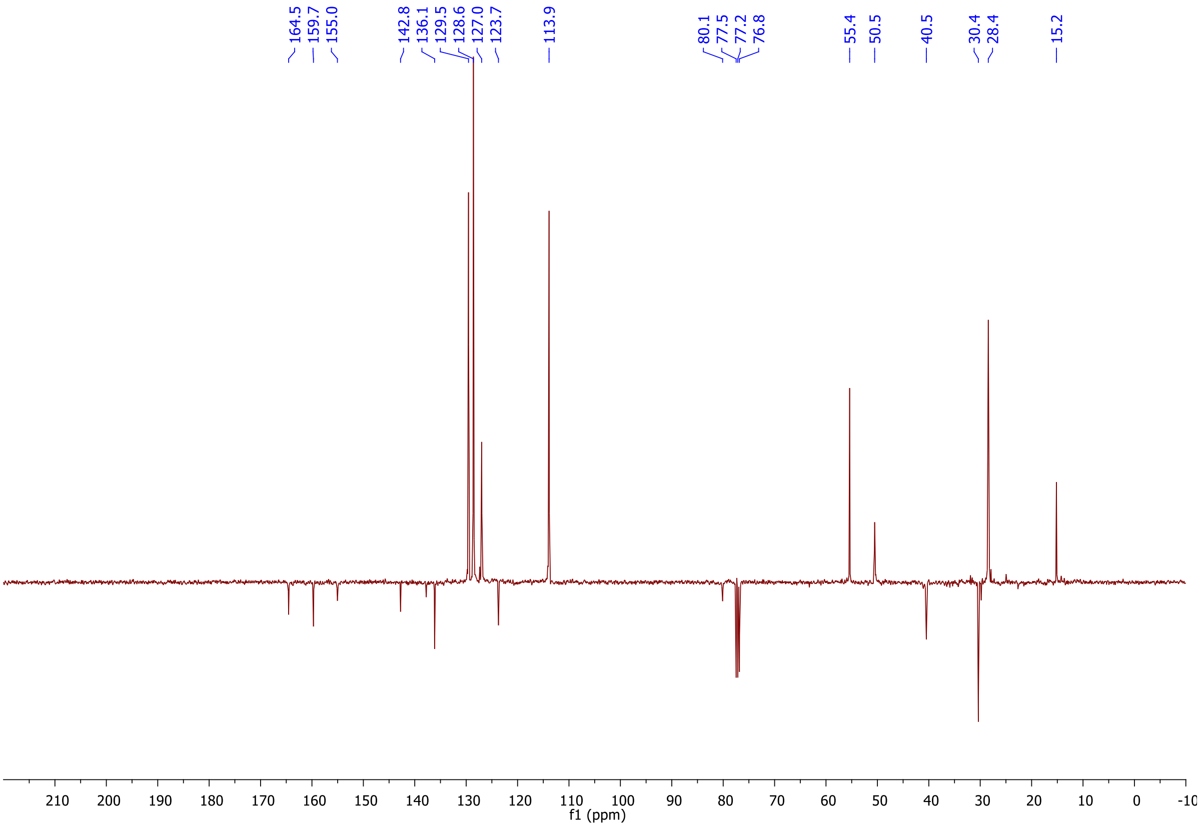

***tert*-Butyl (*S*)-2-[5-(methylthio)-4-phenyloxazol-2-yl]pyrrolidine-1-carboxylate (3ai-*12.4:1*)** in $CDCl_3$
^{1}H-NMR and ^{13}C-NMR (UDEFT)

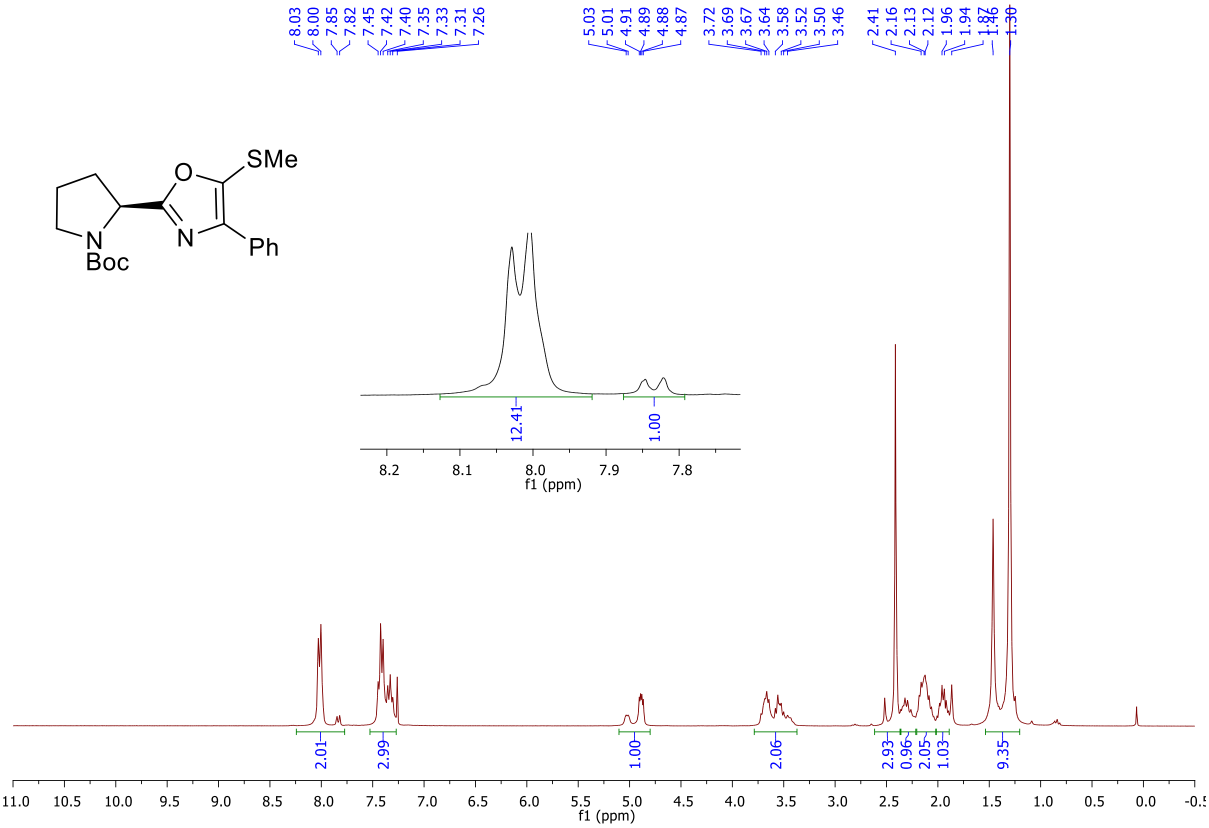

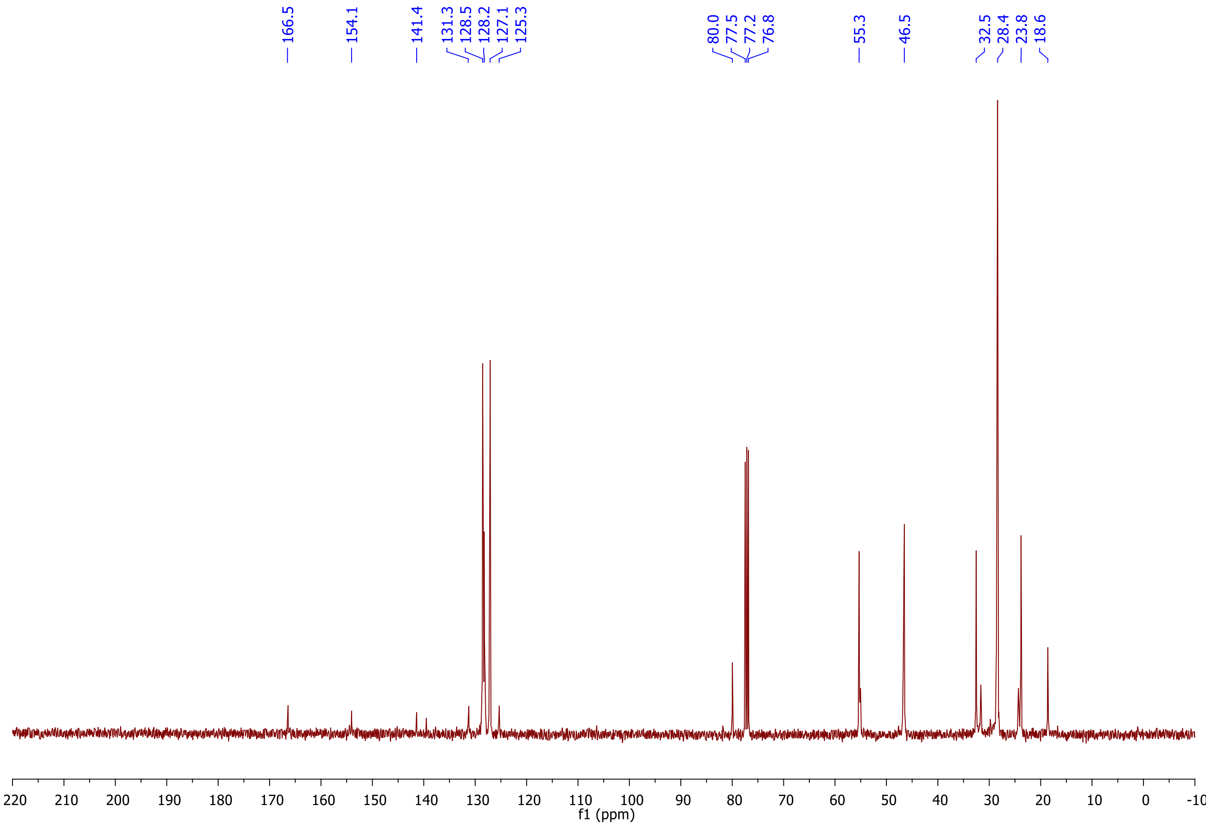

***tert*-Butyl (2*S*,4*R*)-[(4-benzyloxy)-2-(5-(methylthio)-4-phenyloxazol-2-yl]pyrrolidine-1-carboxylate (3aj-*20.0:1*)** in $CDCl_3$ ^{1}H-NMR and ^{13}C-NMR (UDEFT)

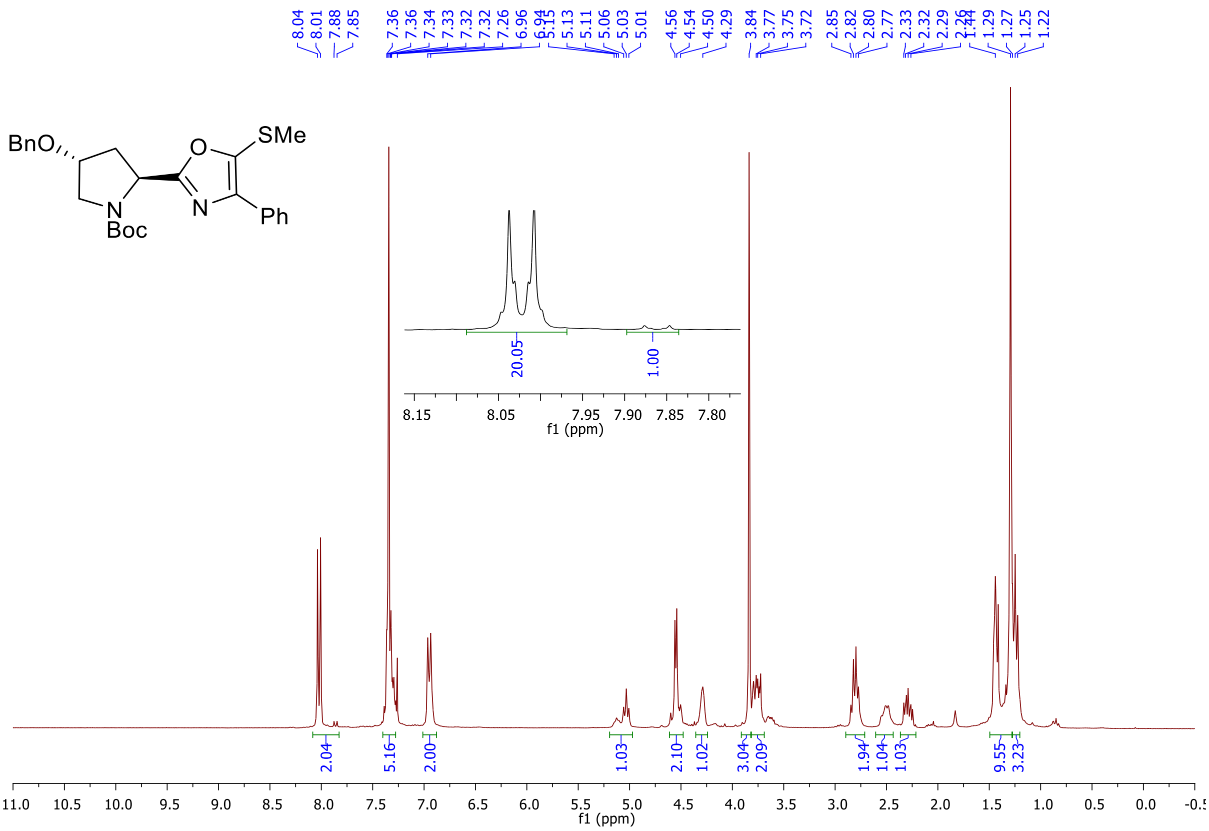

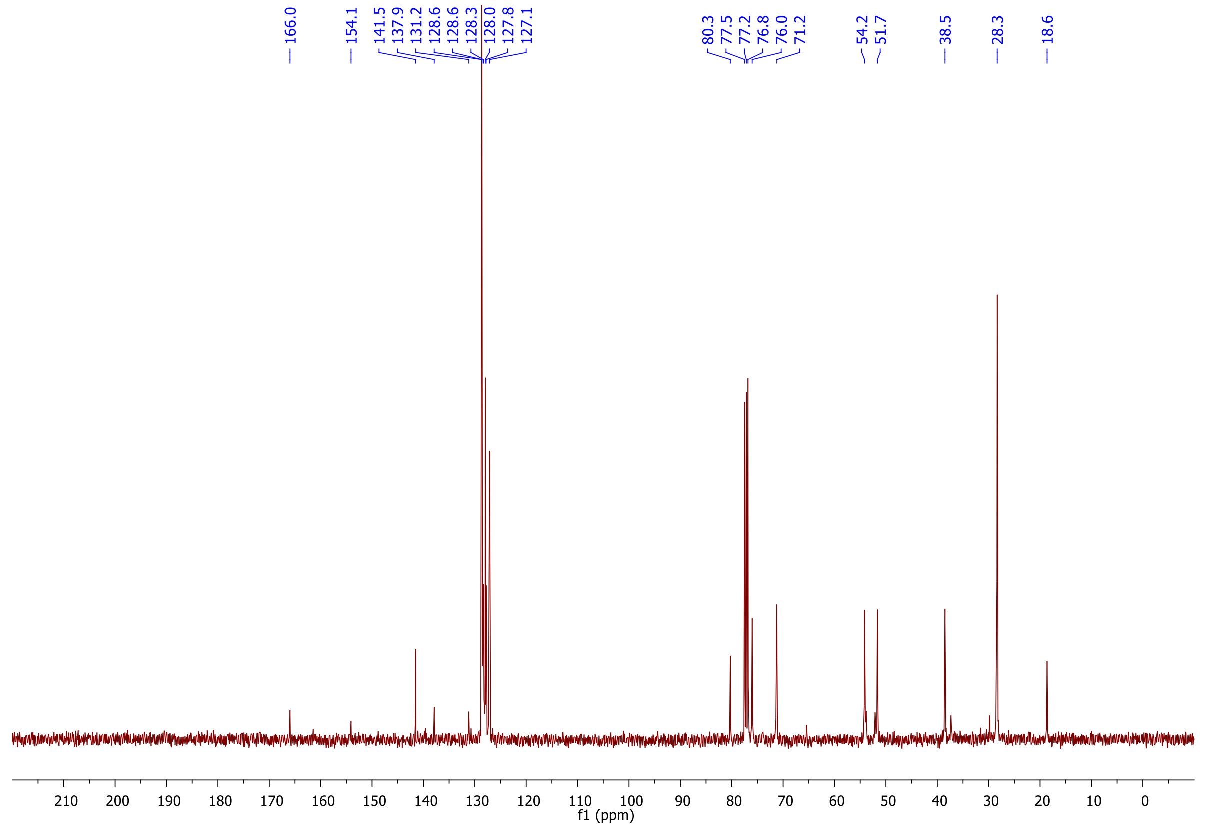

***tert*-Butyl(*S*)-2-[4-(4-methoxyphenyl)-5-(methylthio)oxazol-2-yl]pyrrolidine-1-carbox-ylate (3ii-18.6:1)** in $CDCl_3$ ^1H-NMR and ^{13}C-NMR (UDEFT)

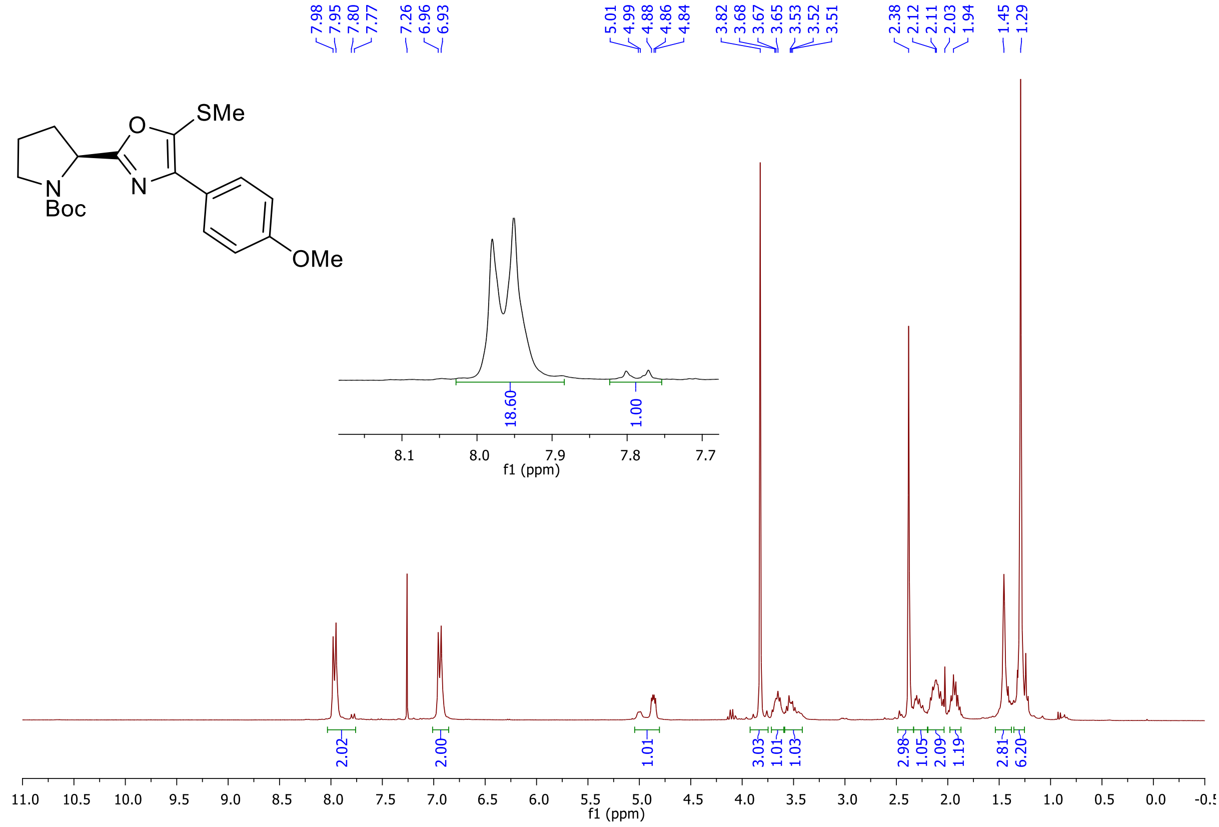

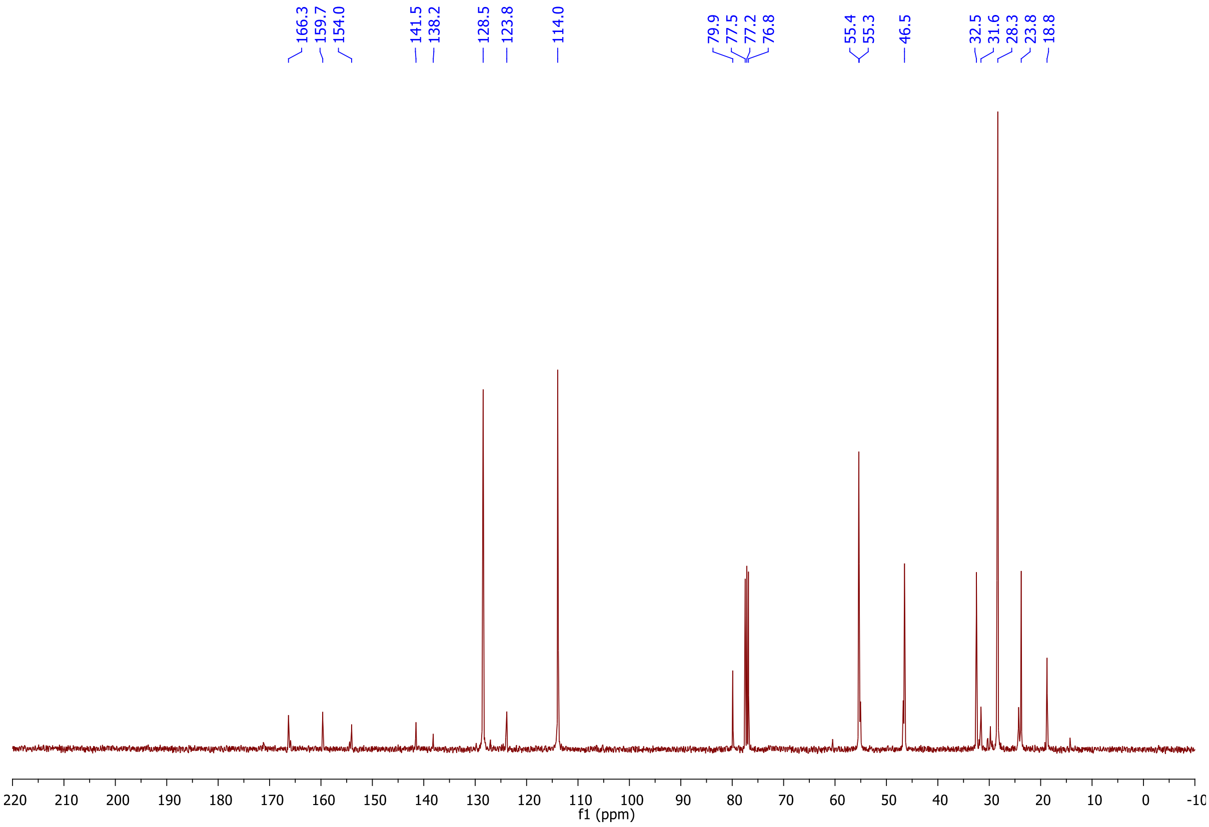

***tert*-Butyl(2*S*,4*R*)-[4-(benzyloxy)-2-(5-(ethylthio)-4-(4-methoxyphenyl)oxazol-2-yl]-pyrro-lidine-1-carboxylate (3gj-*20.0:1*)** in $CDCl_3$ ^{1}H-NMR and ^{13}C-NMR (Pendant)

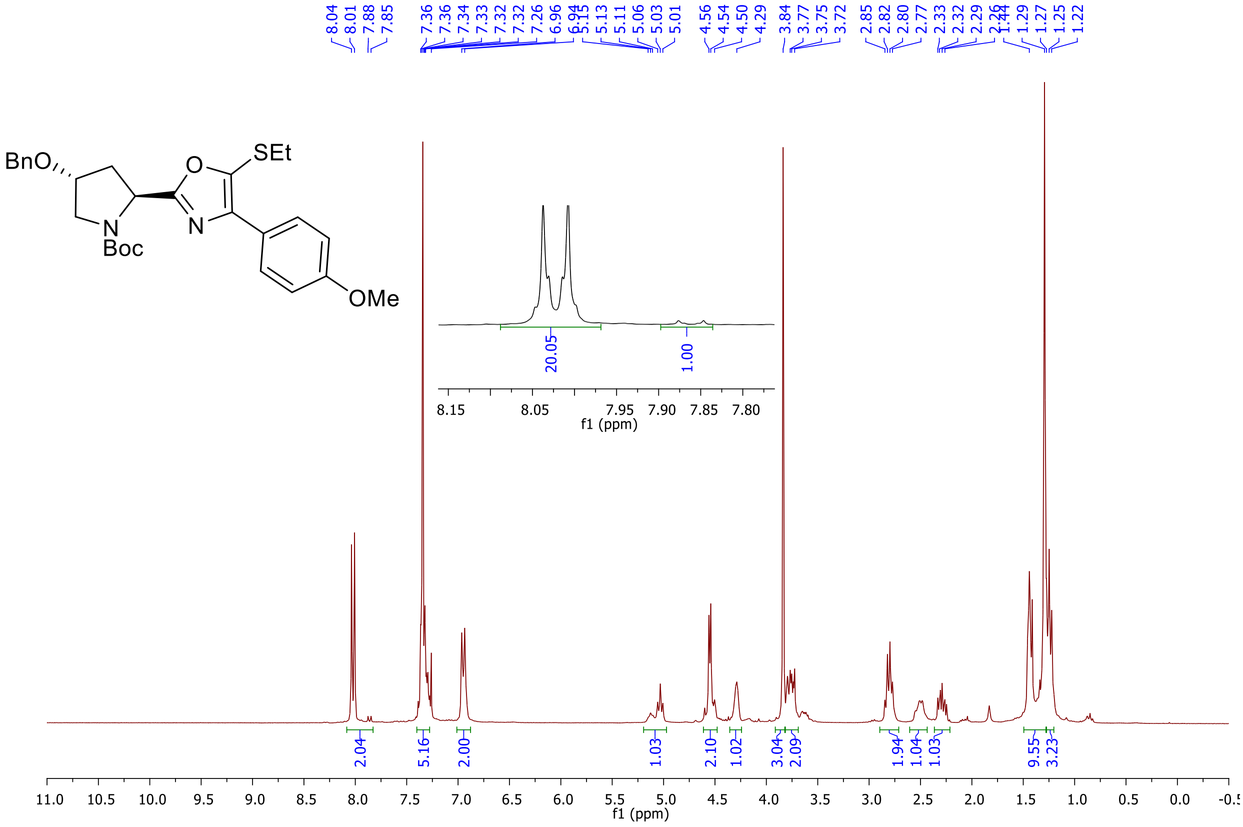

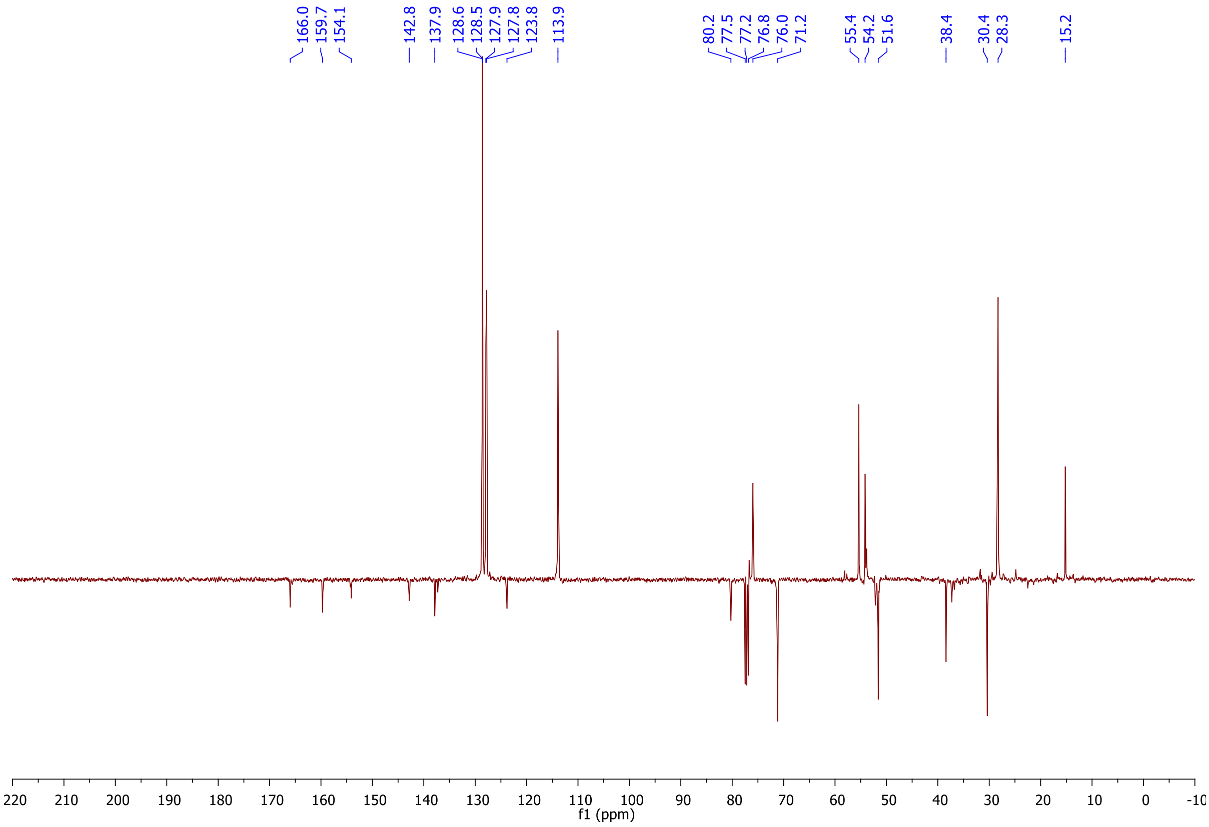

***tert*-Butyl (*S*)-2-[4-(3,4-dimethoxyphenyl)-5-(ethylthio)oxazol-2-yl]pyrrolidine-1-carbo-xylate (3ki)**
in $CDCl_3$ ^1H-NMR and ^{13}C-NMR (Pendant)

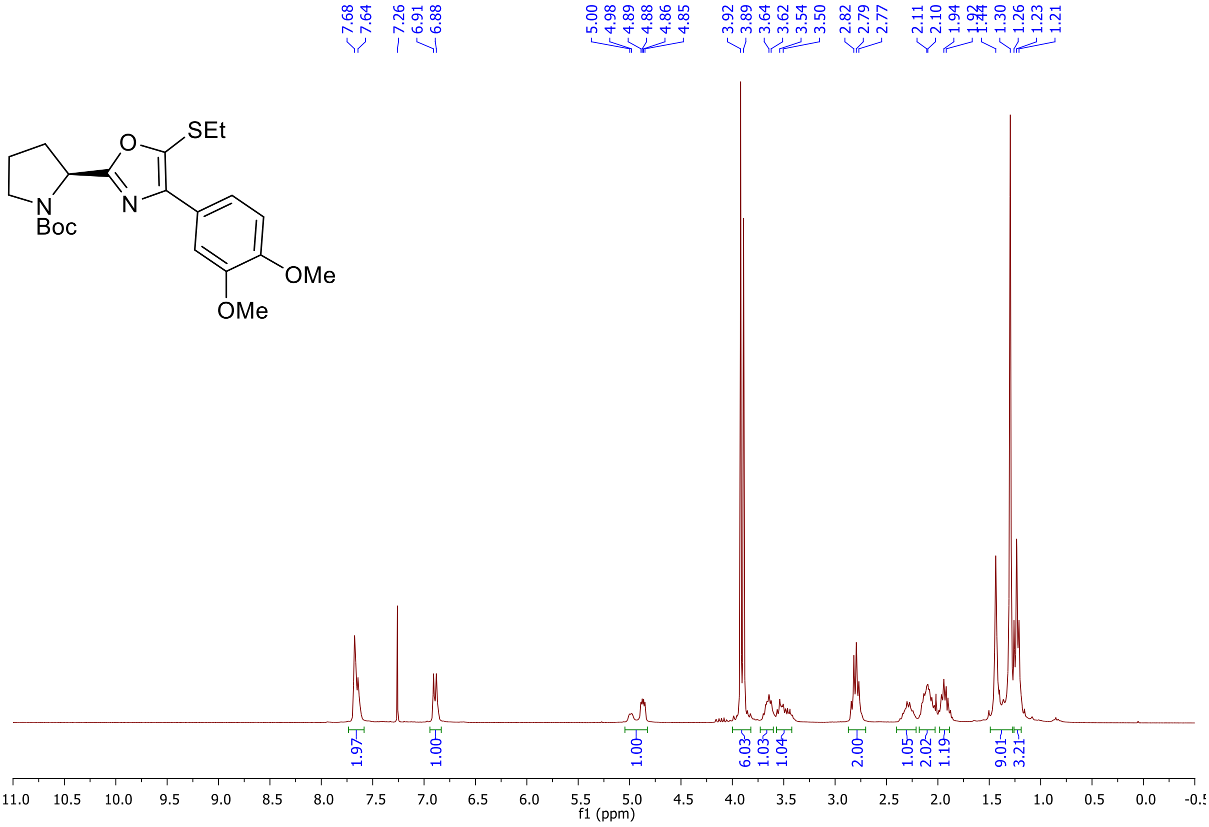

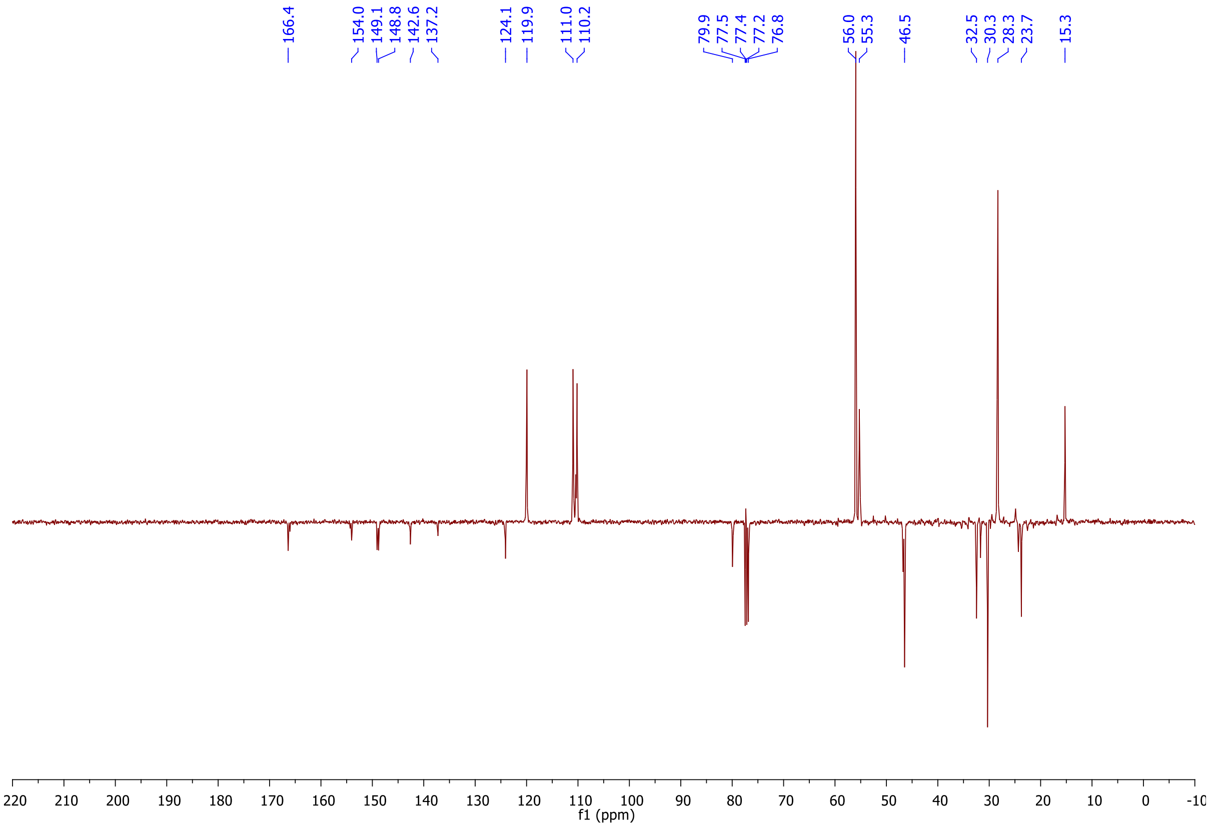

***tert*-Butyl (2*S*,4*R*)-[4-(benzyloxy)-2-(4-(3,4-dimethoxyphenyl)-5-(ethylthio)oxazol-2-yl]-pyrrolidine-1-carboxylate (3kj)** in $CDCl_3$ ^{1}H-NMR and ^{13}C-NMR (UDEFT)

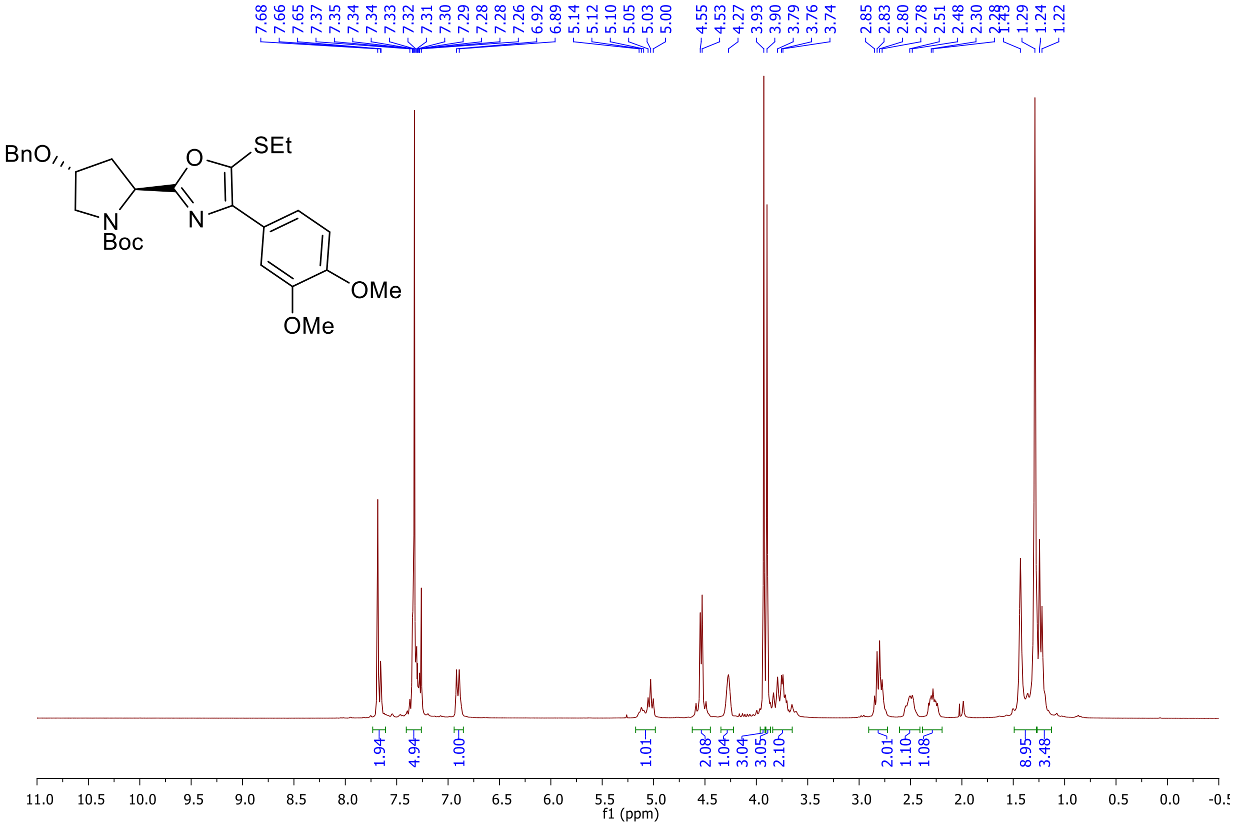

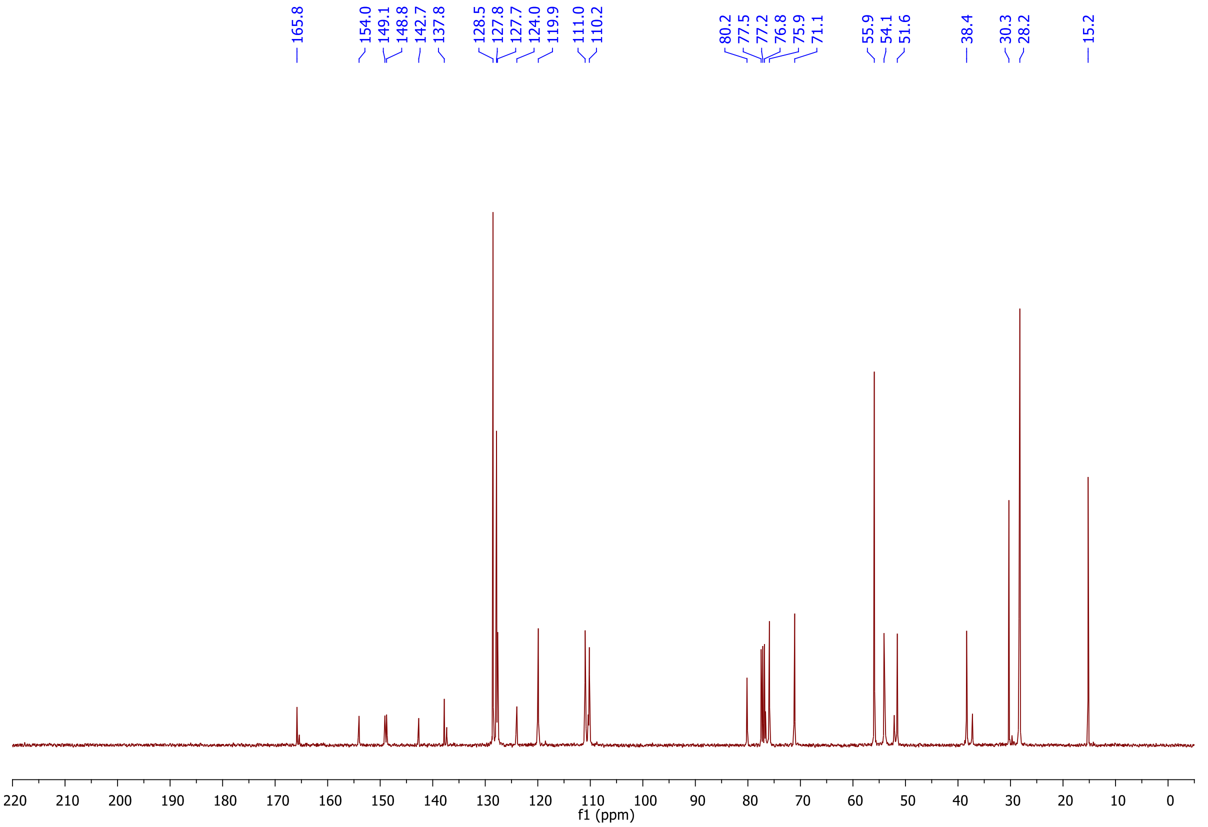

5-(Methylthio)-2-(naphthalen-2-yl)-4-(2,4,6-trimethylphenyl)oxazole (3lc) in $CDCl_3$ ^{1}H-NMR and ^{13}C-NMR (Pendant)

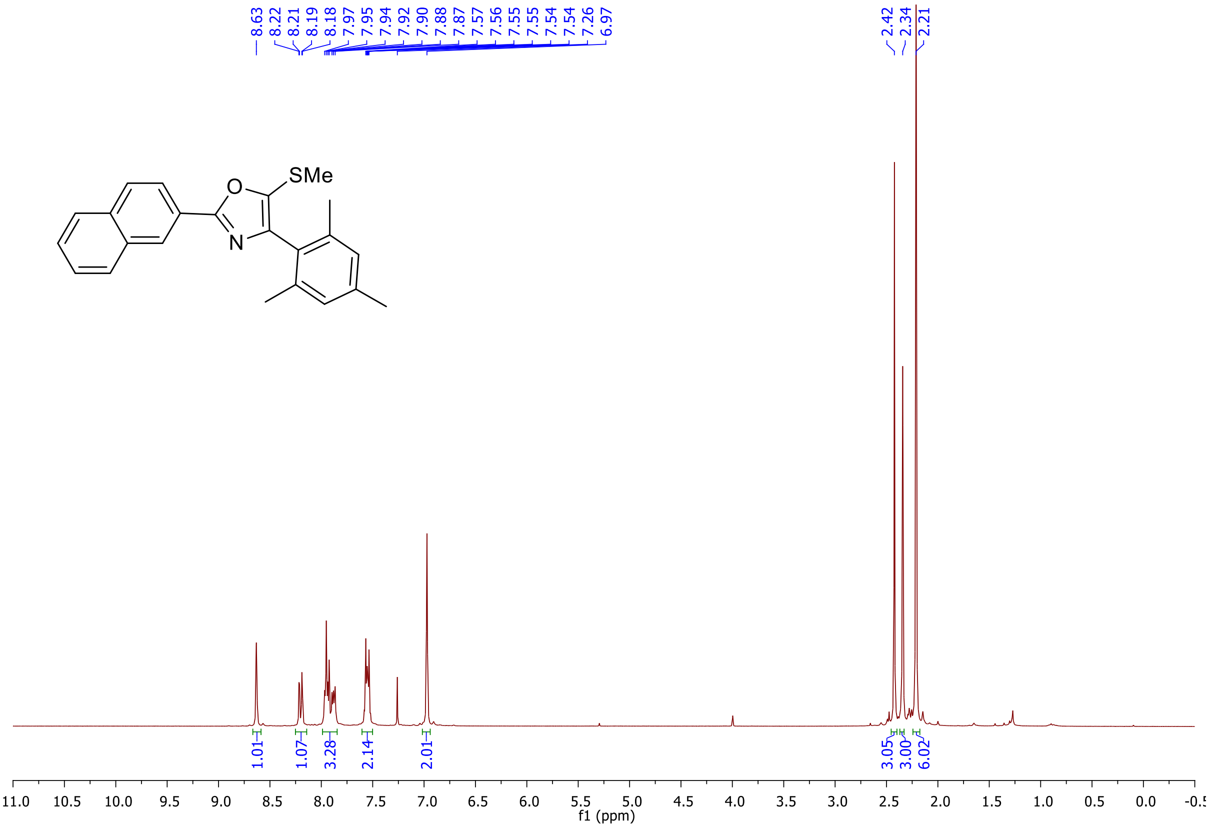

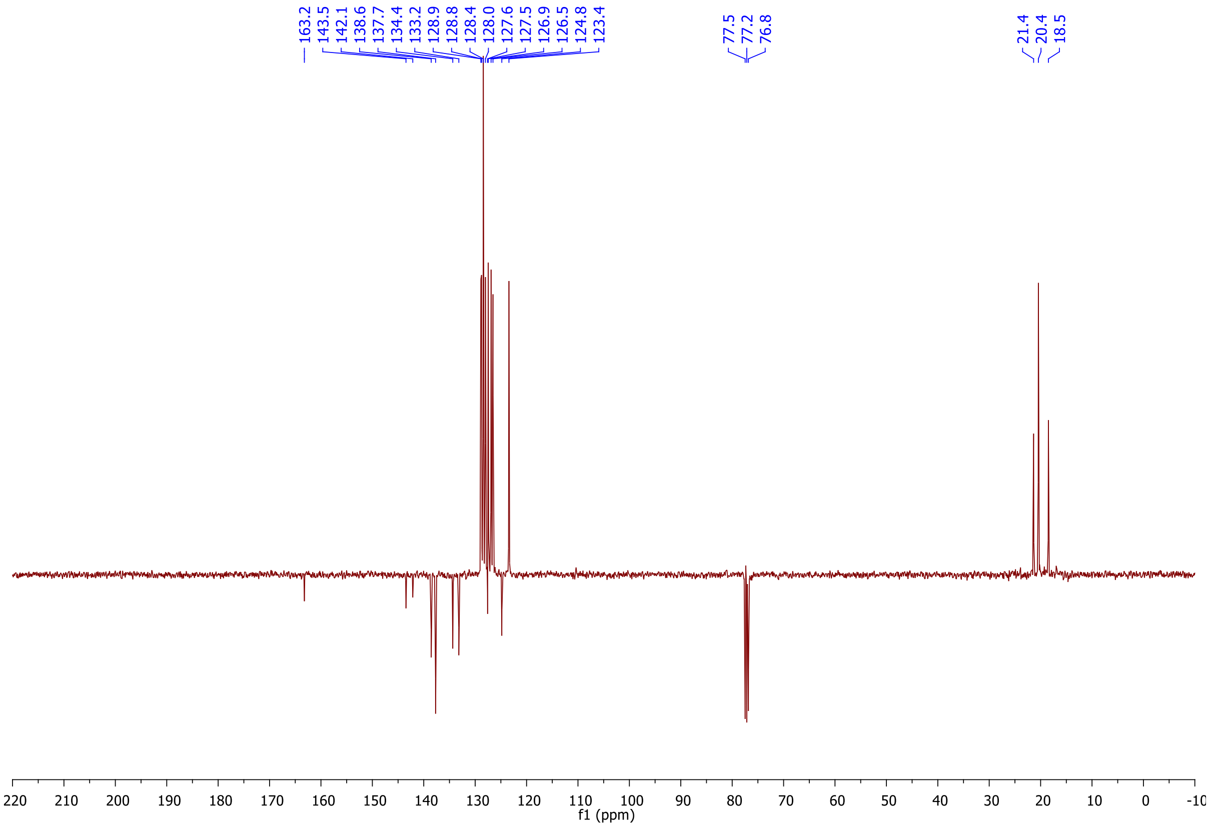

tert-Butyl ((4-(4-(diethylamino)phenyl)-5-(methylthio)oxazol-2-yl)methyl)carbamate (3mh) in $CDCl_3$ ^{1}H-NMR and ^{13}C-NMR (UDEFT)

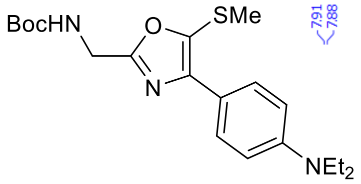

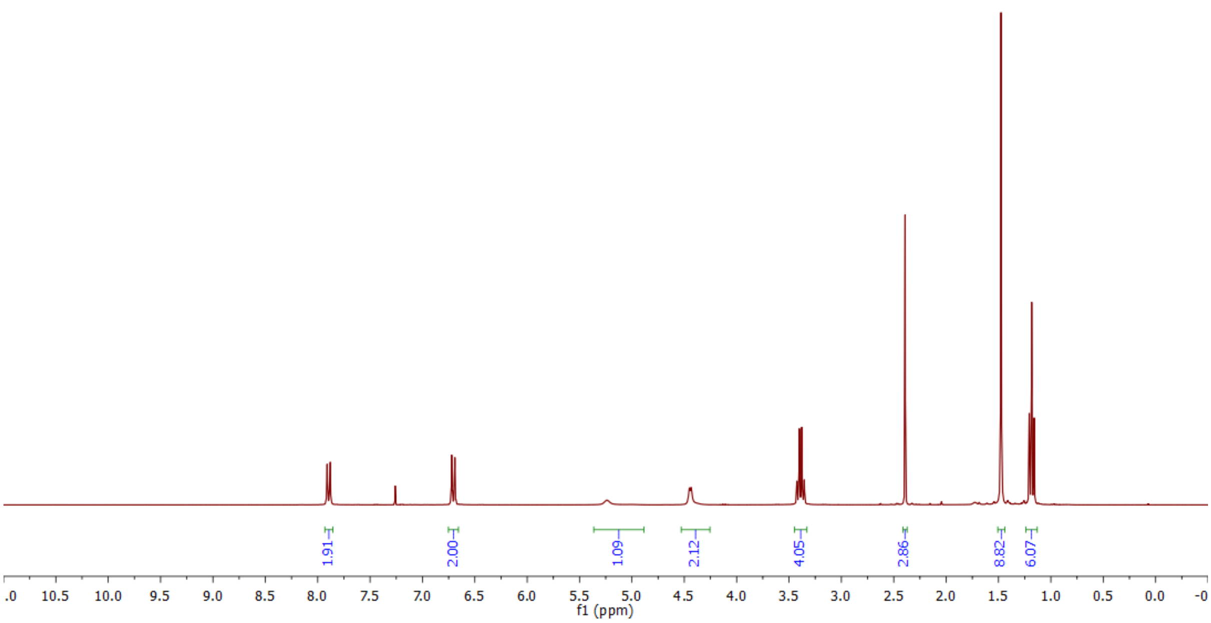

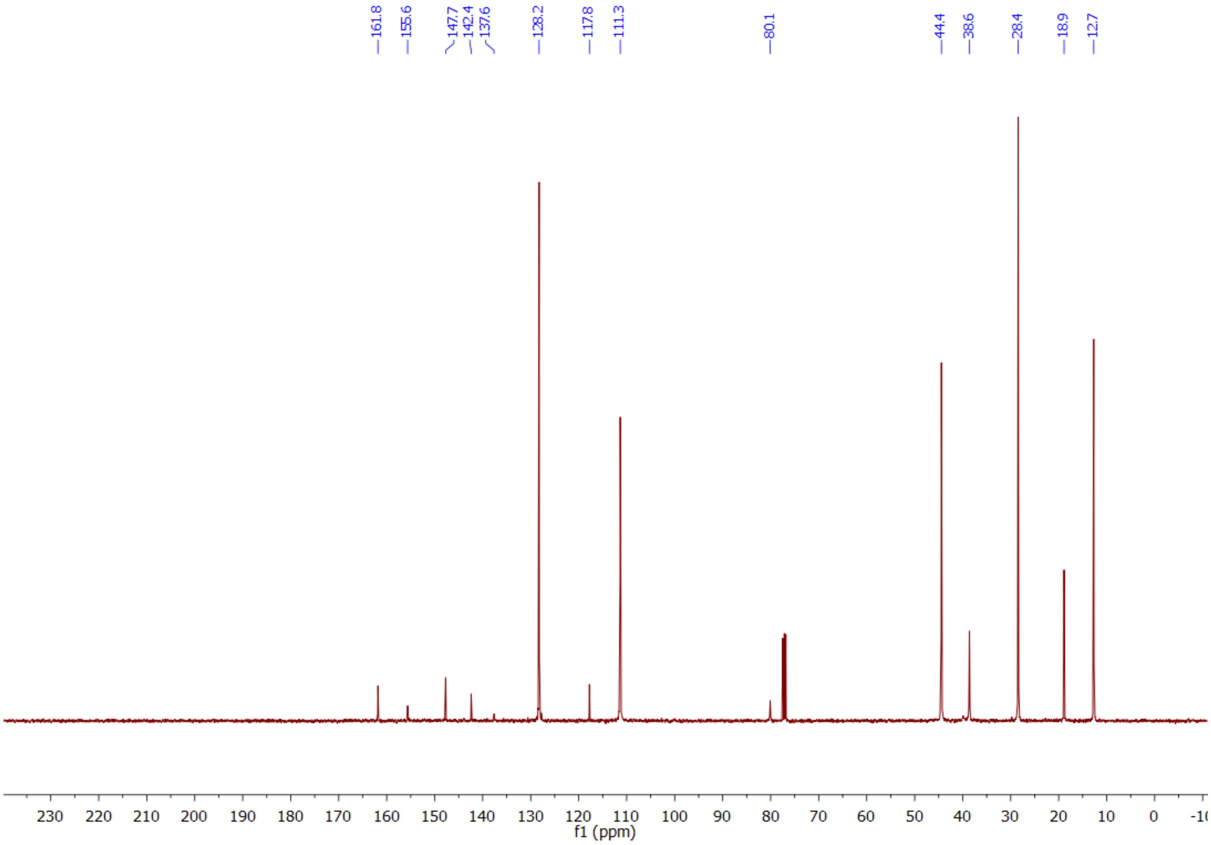

5-(Methylthio)-2-phenyl-4-(1-tosyl-1H-indol-3-yl)oxazole (3na-*30.5:1*) in $CDCl_3$ ^1H-NMR and ^{13}C-NMR (JMOD)

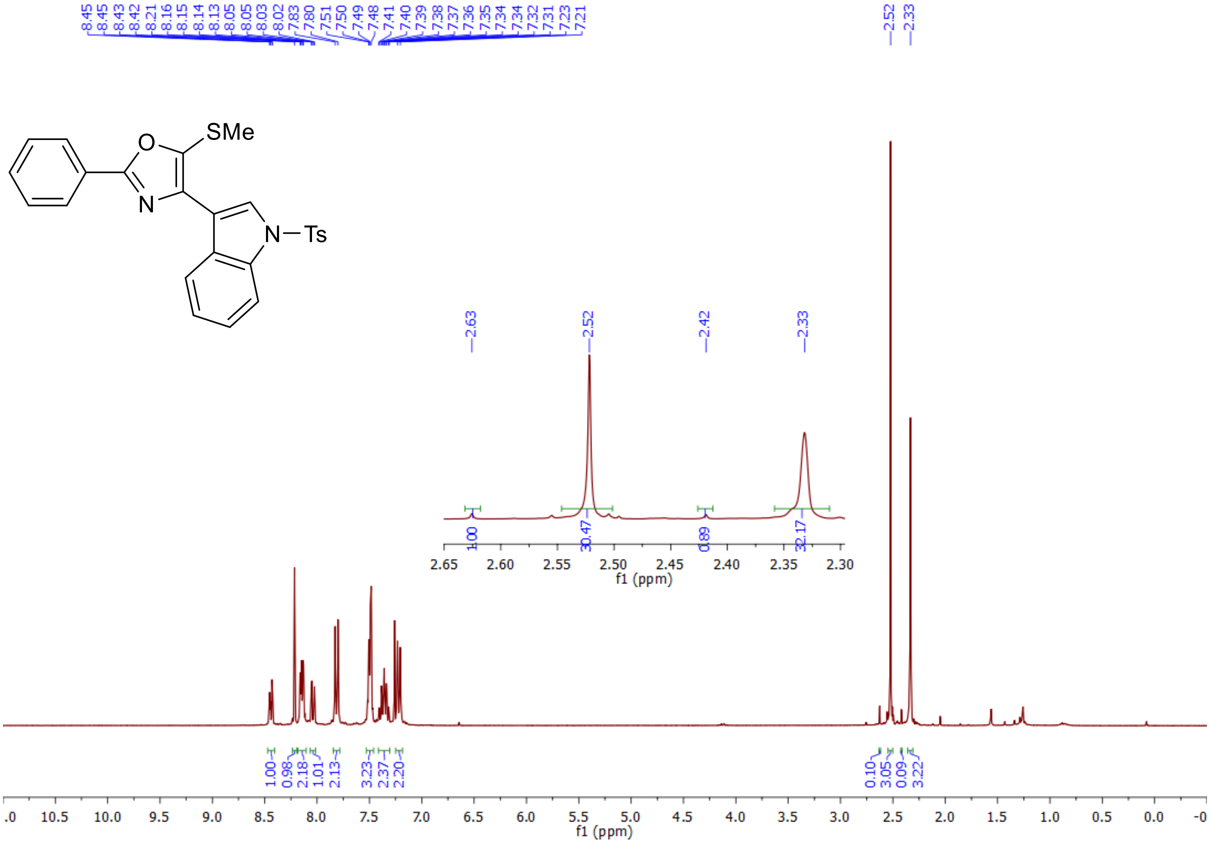

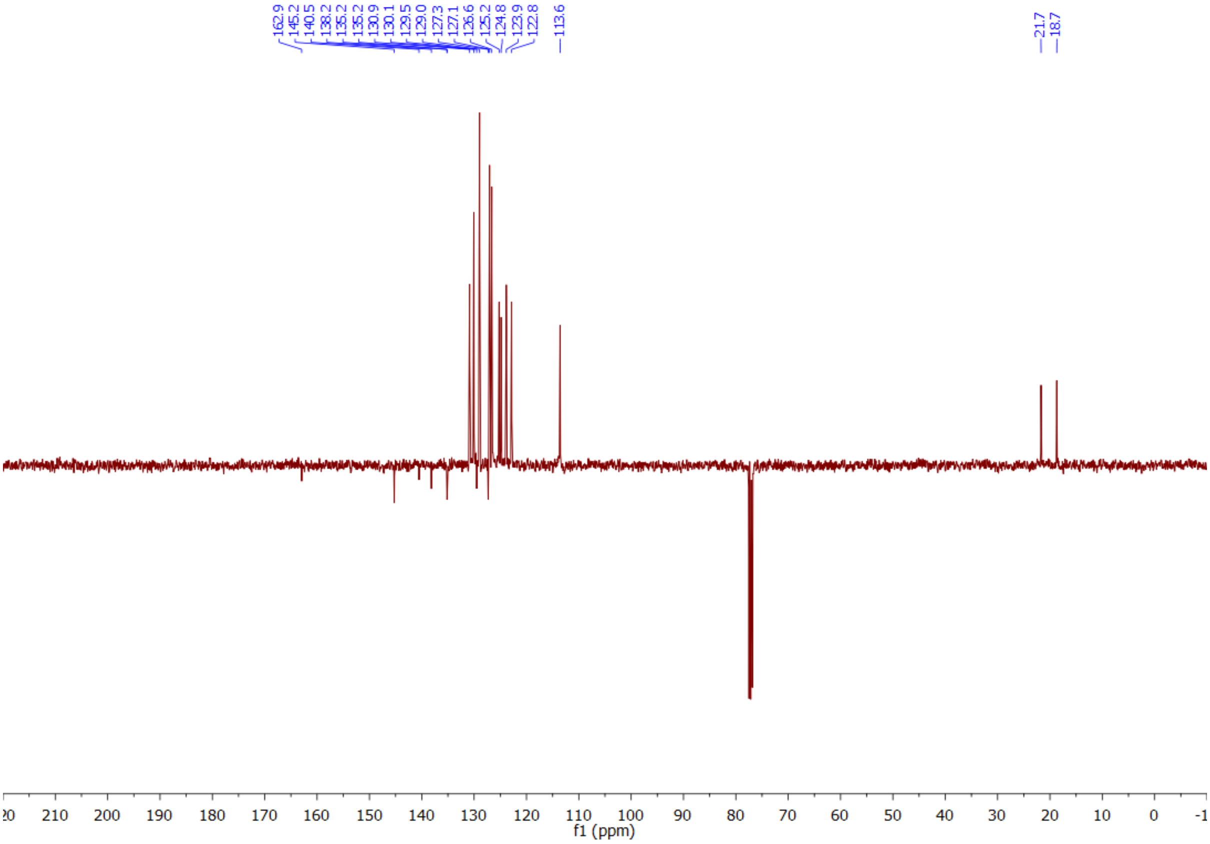

2-(2-Bromophenyl)-5-(methylthio)-4-(1-tosyl-1H-indol-3-yl)oxazole (3nb-*20.2:1*) in $CDCl_3$ ^{1}H-NMR and ^{13}C-NMR (UDEFT)

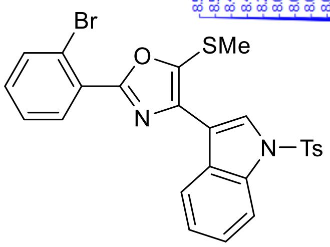

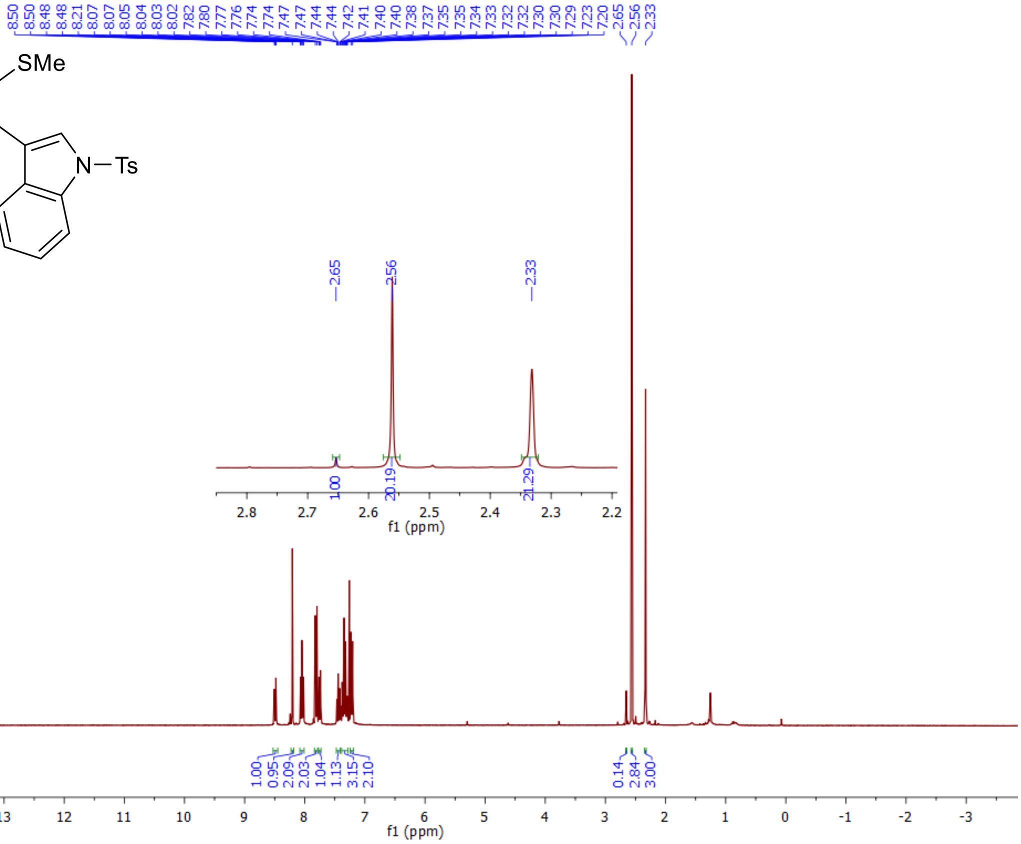

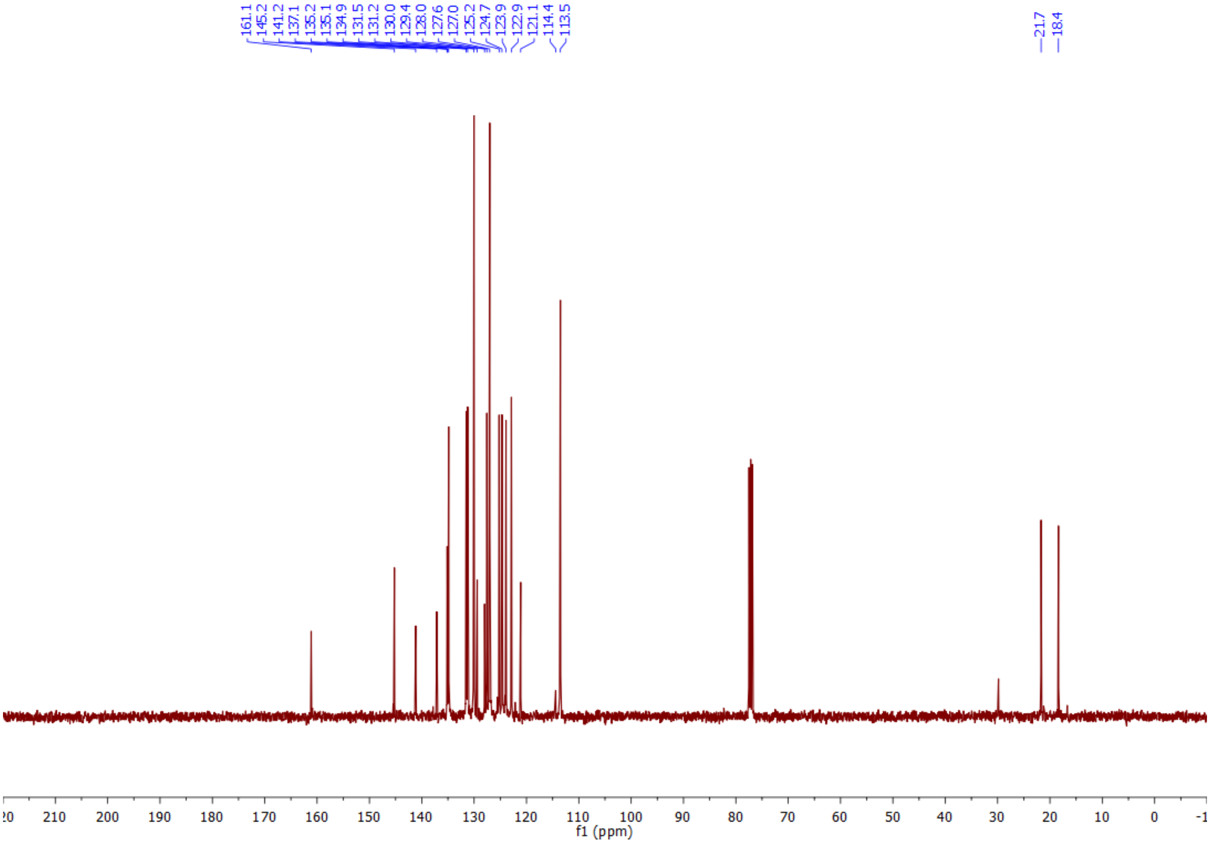

tert-Butyl ((5-(methylthio)-4-(1-tosyl-1H-indol-3-yl)oxazol-2-yl)methyl)carbamate (3nh) in $CDCl_3$ ^1H-NMR and ^{13}C-NMR (UDEFT)

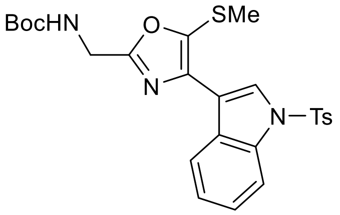

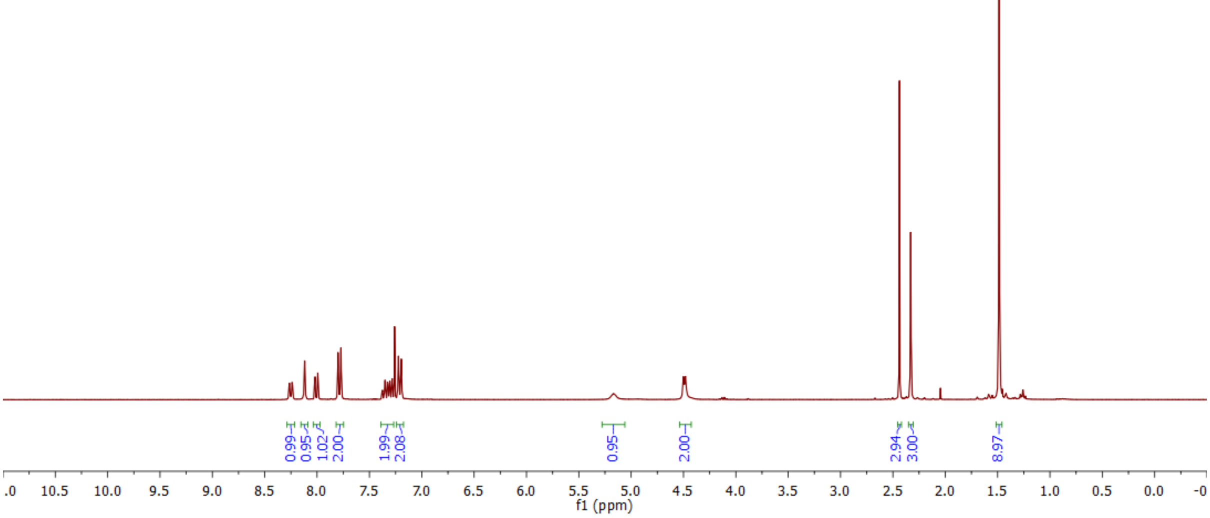

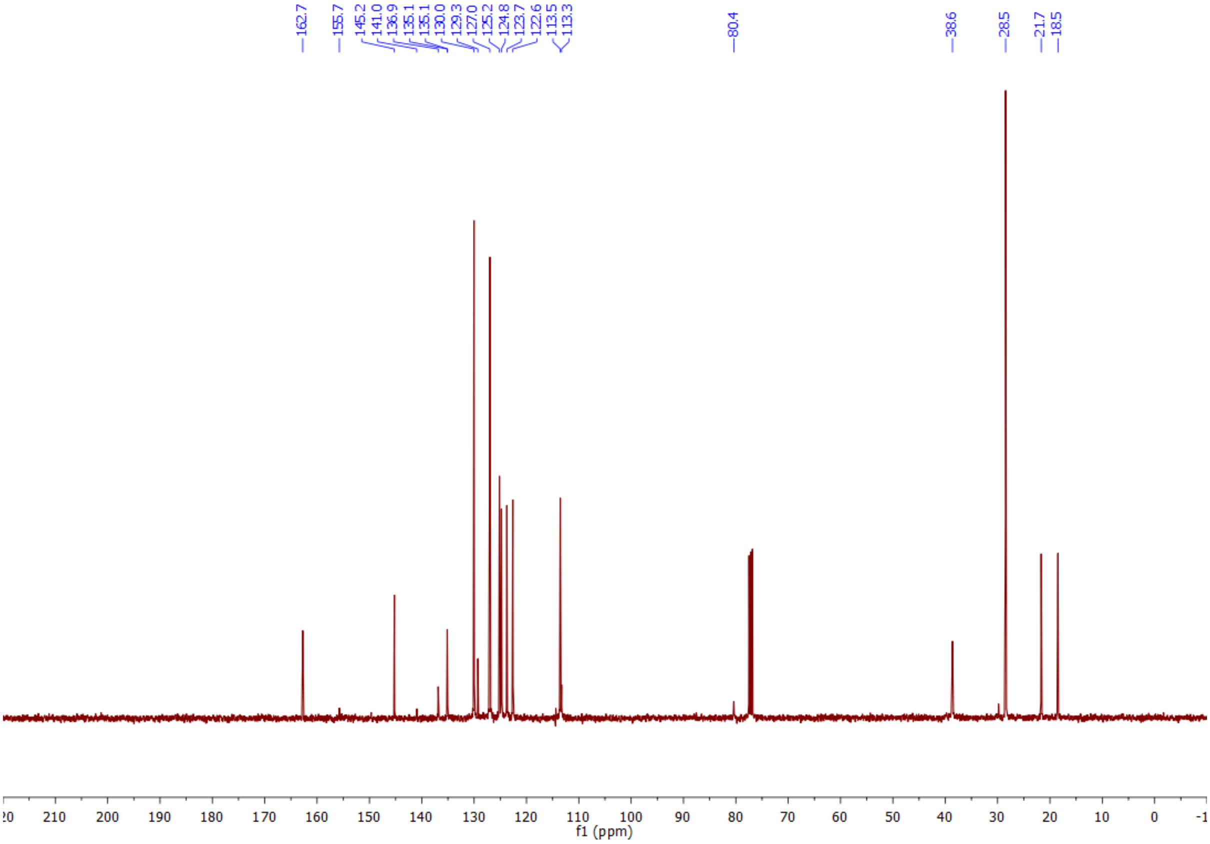

Methyl 2-(5-(methylthio)-4-(1-tosyl-1H-indol-3-yl)oxazol-2-yl)benzoate (3nl-*18.3:1*) in $CDCl_3$ ^{1}H-NMR and ^{13}C-NMR (UDEFT)

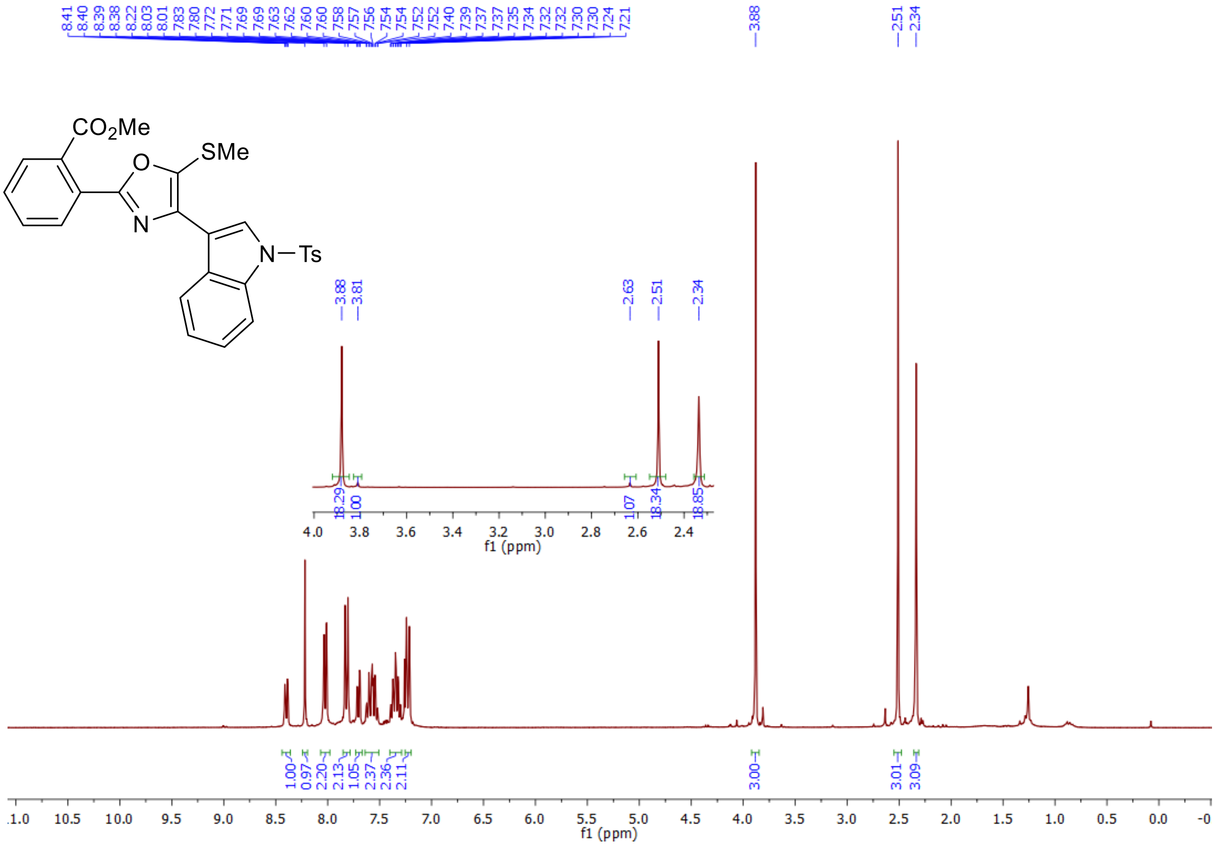

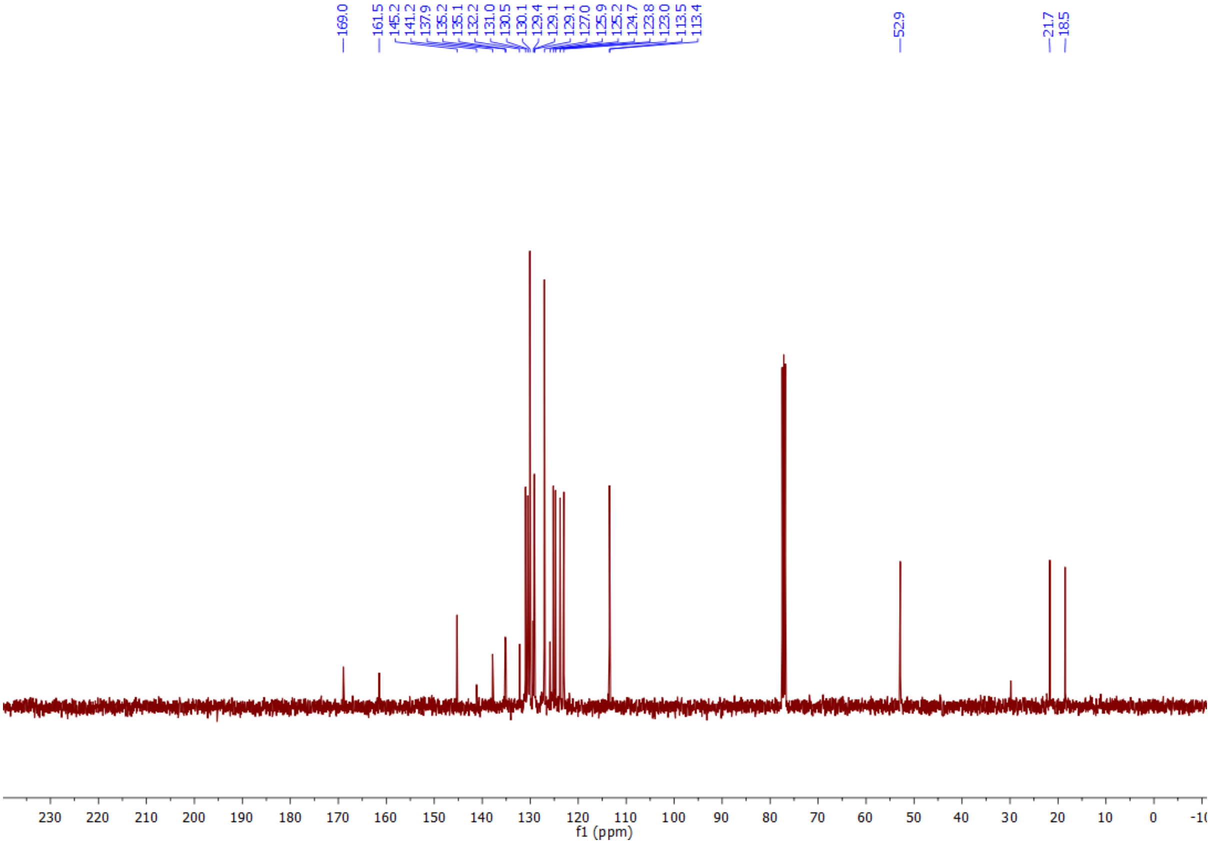

5-(Methylthio)-2-(pyridin-2-yl)-4-(1-tosyl-1H-indol-3-yl)oxazole (3nm) in $CDCl_3$ ^1H-NMR and ^{13}C-NMR (UDEFT)

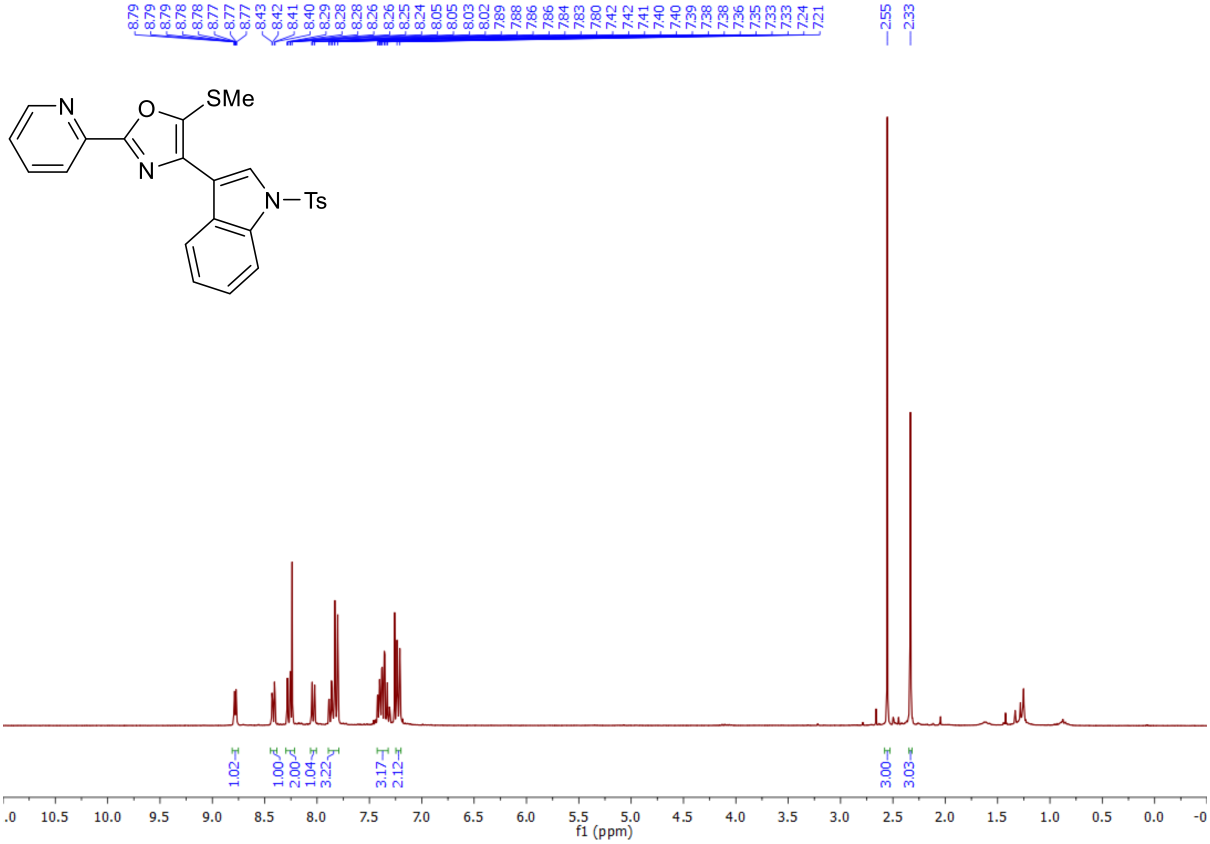

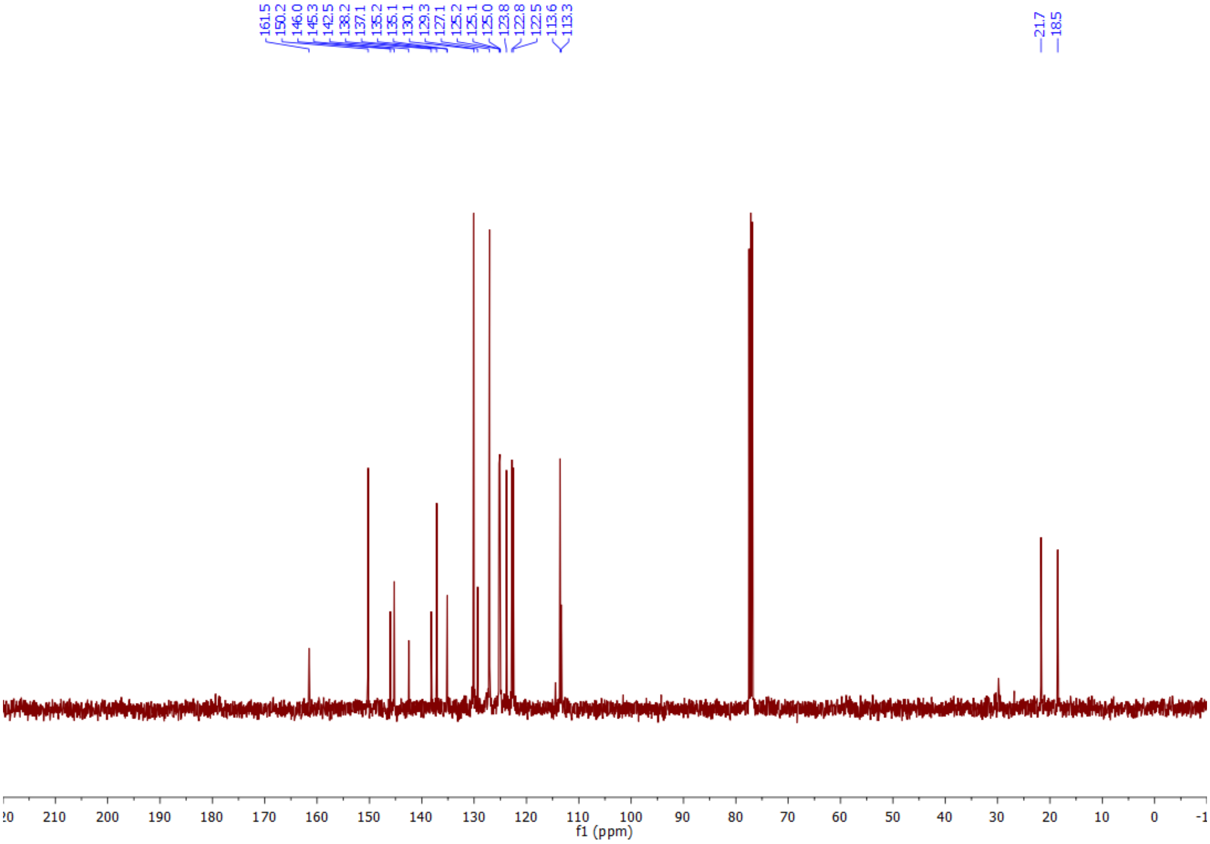

Methyl 2-(5-(methylthio)-4-(1-tosyl-1H-indol-3-yl)oxazol-2-yl)acetate (3nn) in $CDCl_3$ ^{1}H-NMR and ^{13}C-NMR (JMOD)

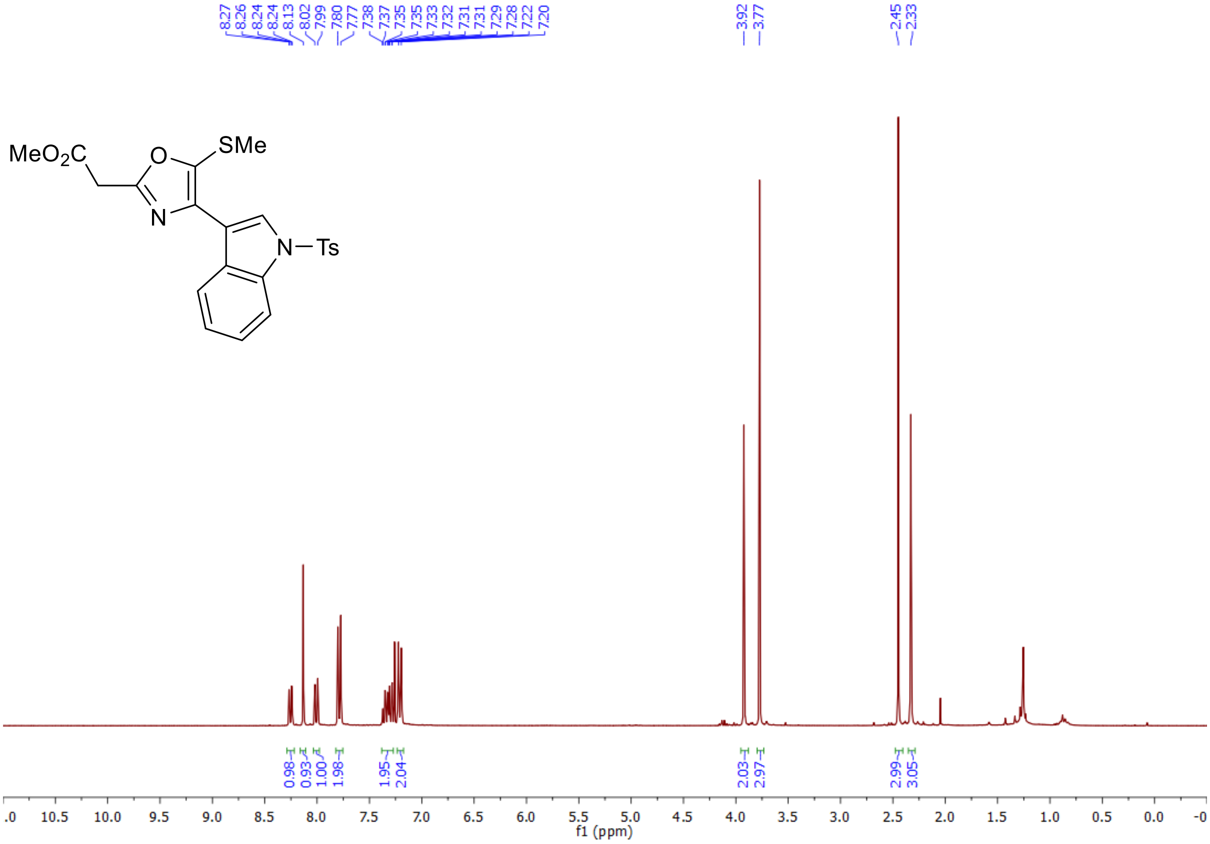

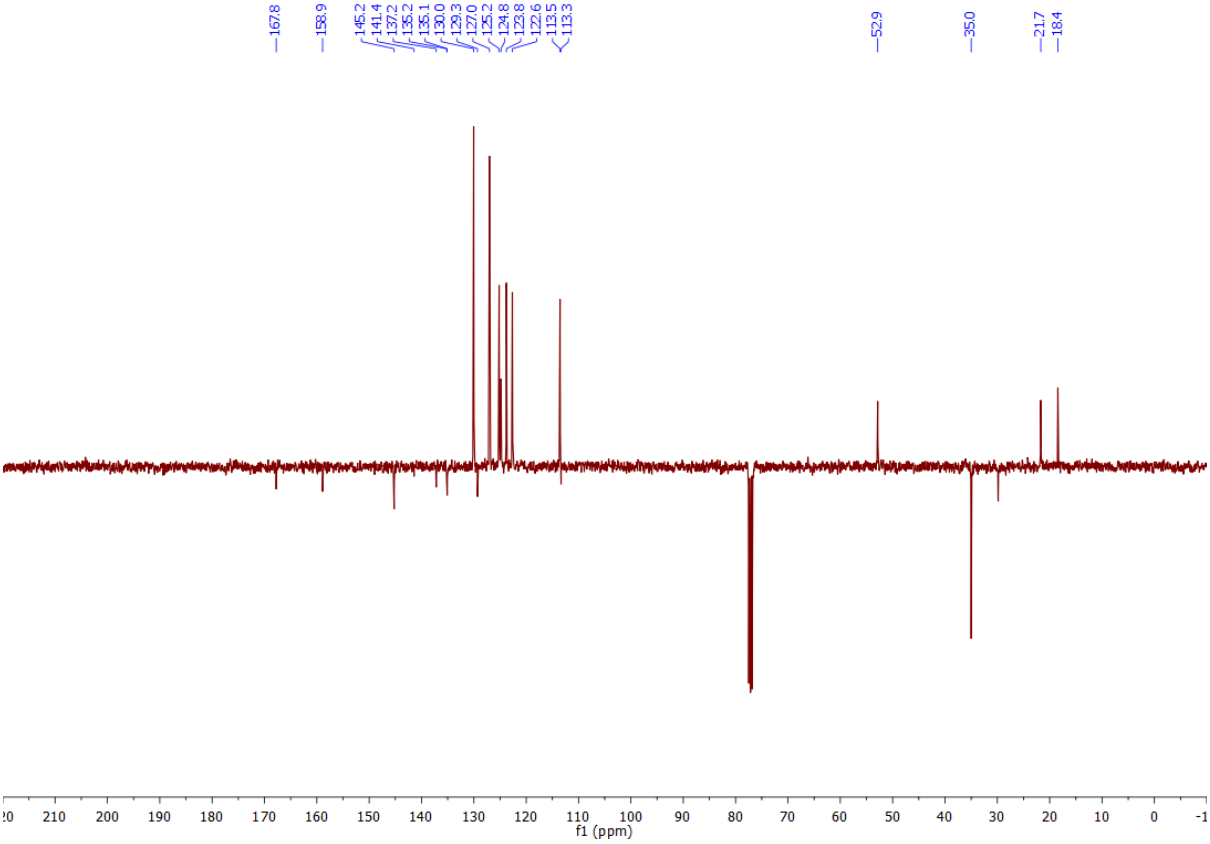

4-(Methylthio)-2,5-diphenyloxazole (3aa') in $CDCl_3$ ^1H-NMR and ^{13}C-NMR (UDEFT)

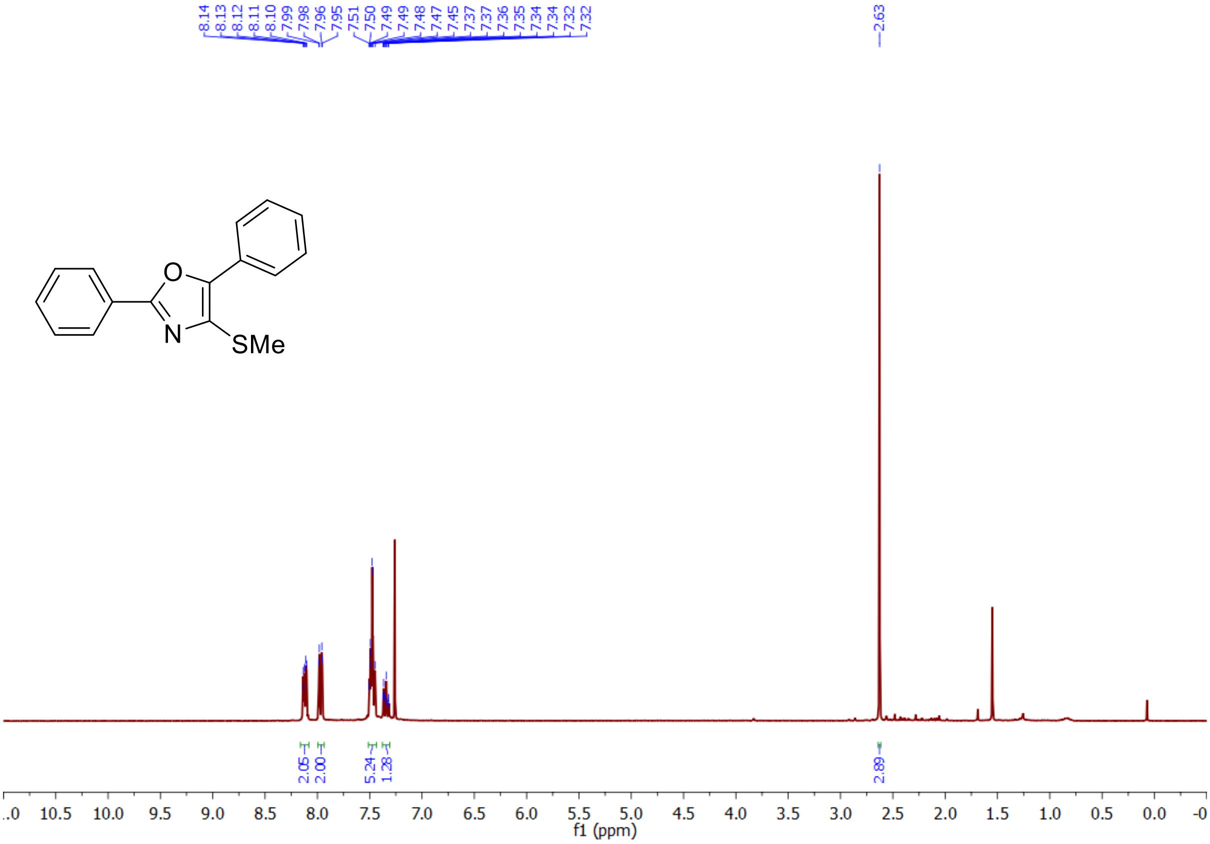

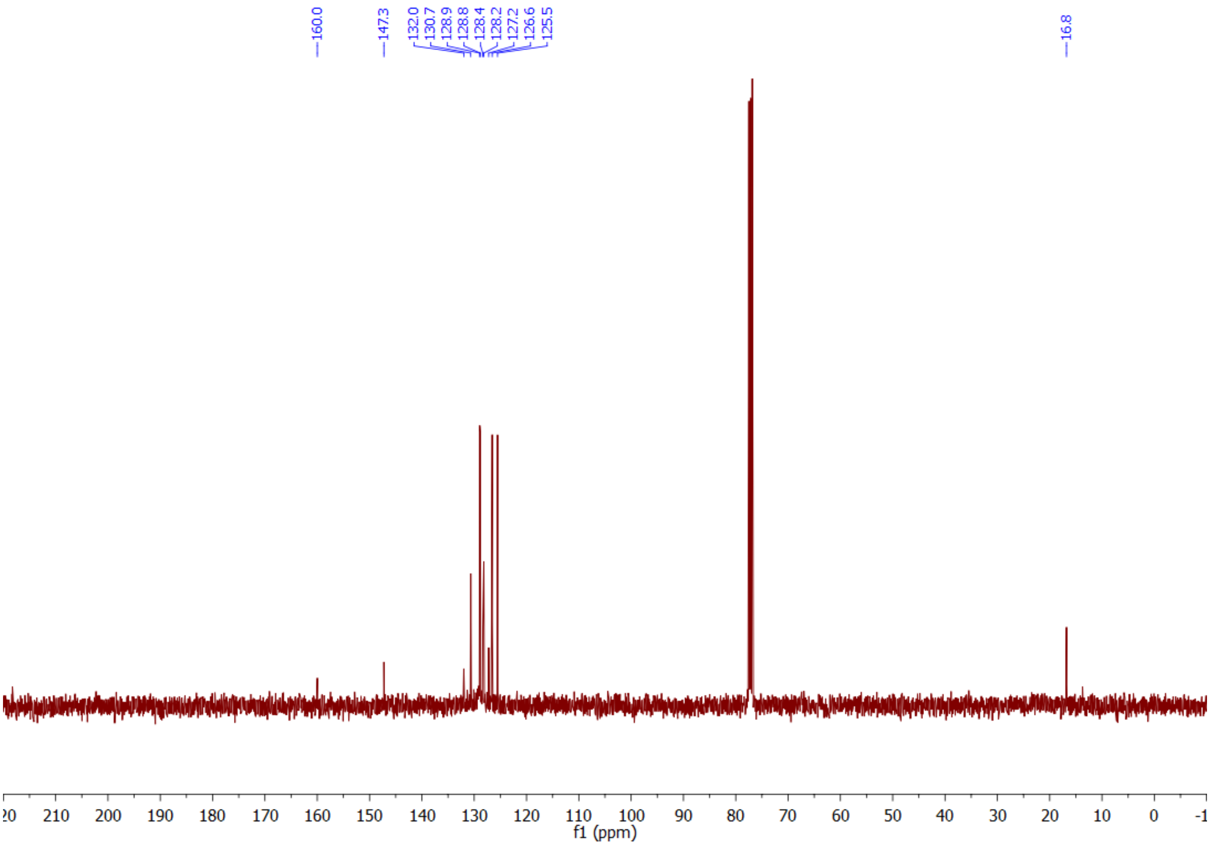

2,4-Diphenyl-5-methyloxazole (5a-major) in $CDCl_3$ ^{1}H-NMR and ^{13}C-NMR (Pendant)

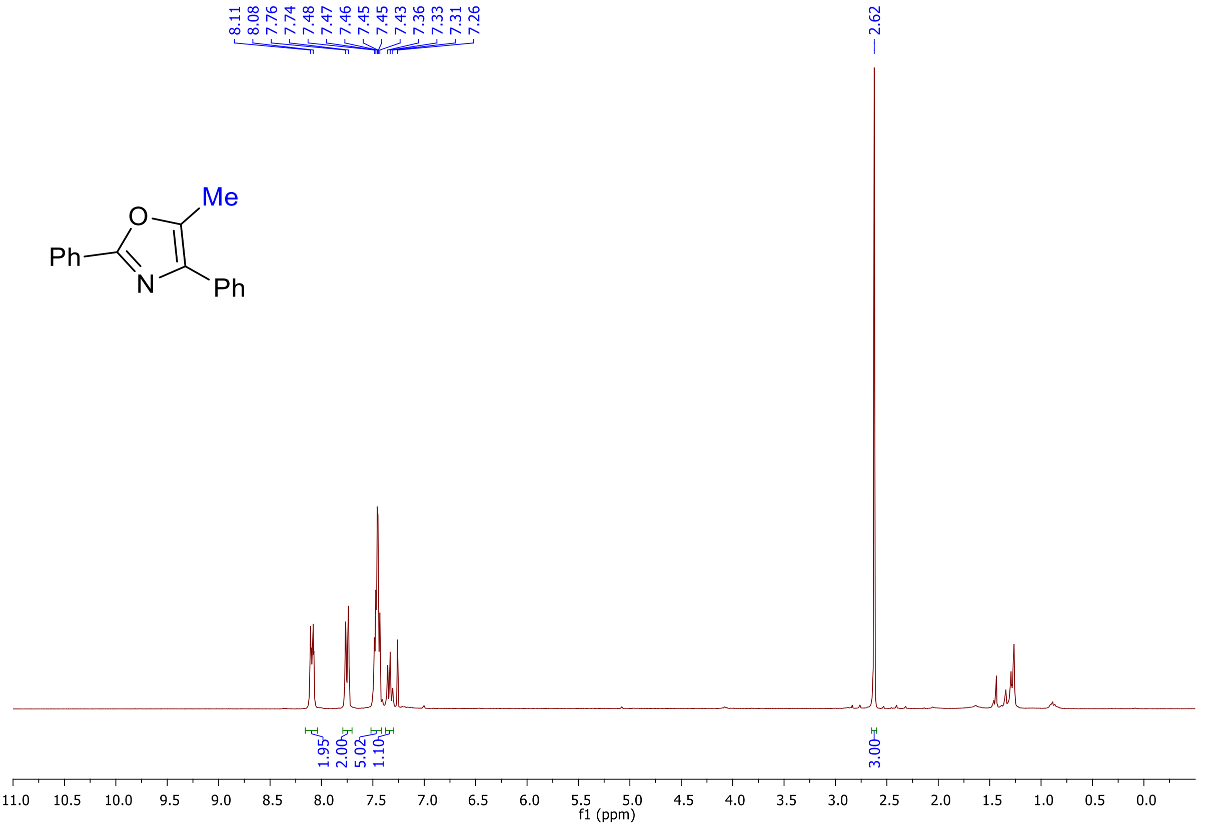

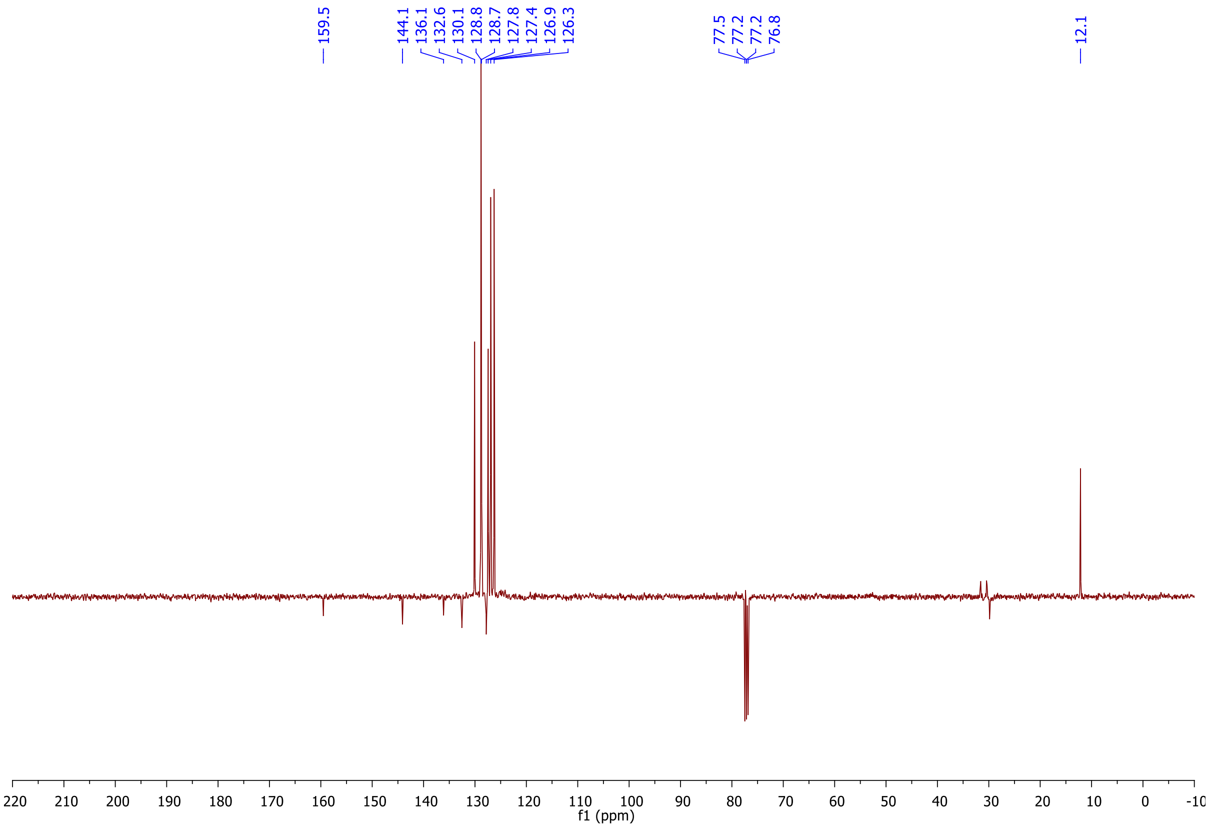

2,5-Diphenyl-4-methyloxazole (5a'-minor isomer) in $CDCl_3$ ^1H-NMR and ^{13}C-NMR (Pendant)

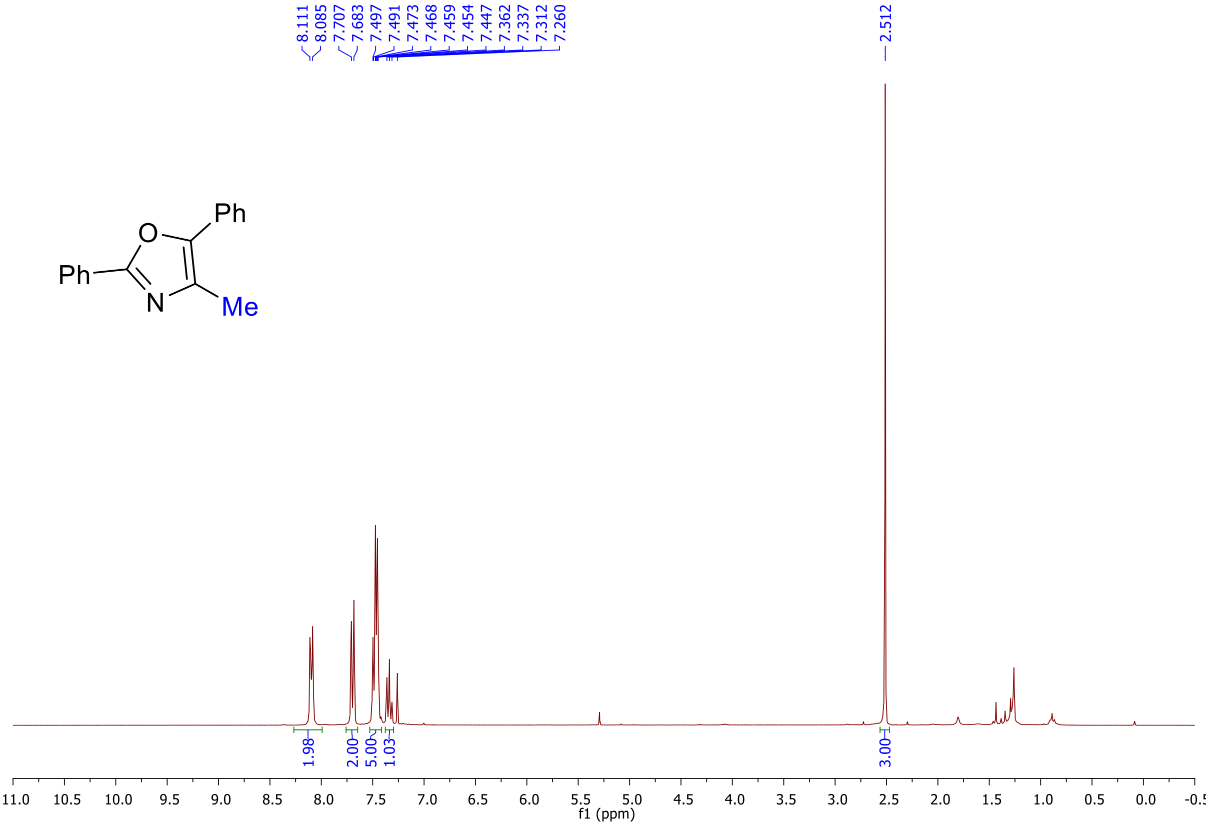

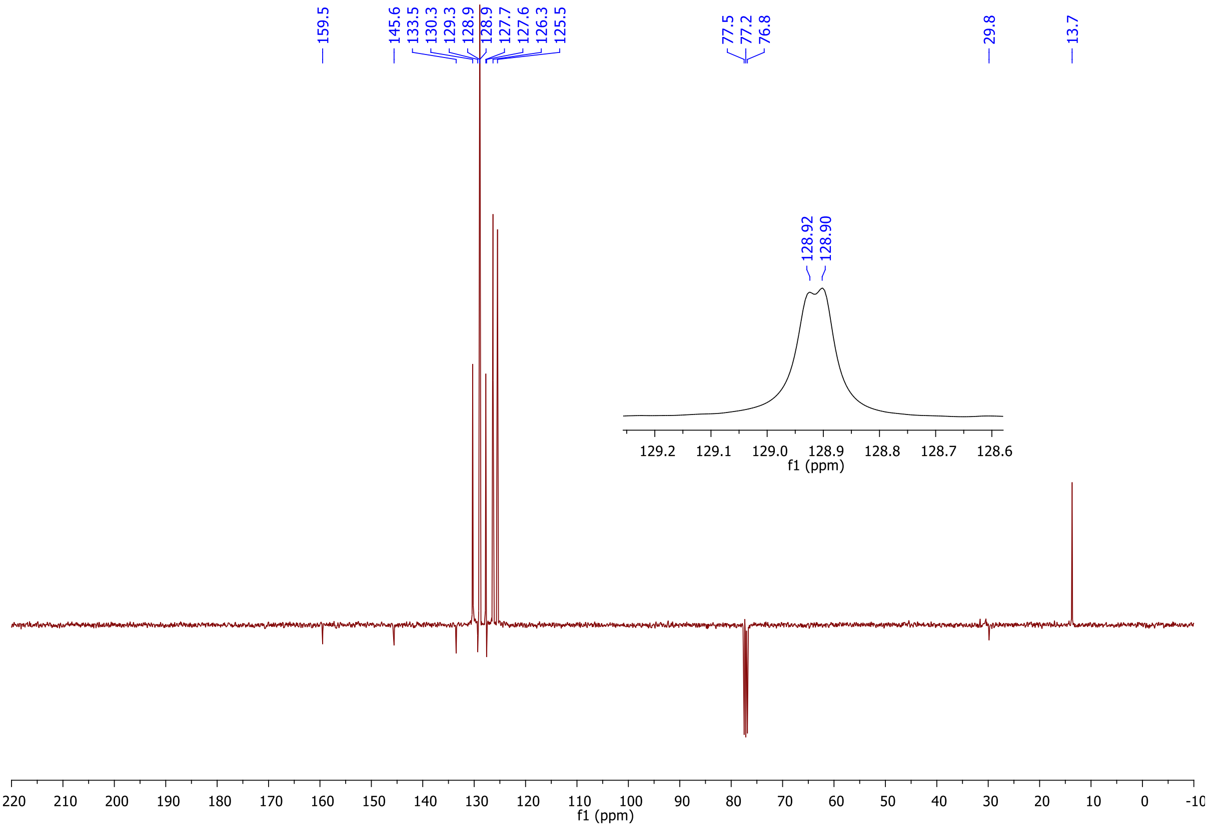

4-(4-Methoxyphenyl)-5-methyl-2-phenyloxazole (5b) in $CDCl_3$ ^{1}H-NMR and ^{13}C-NMR (Pendant)

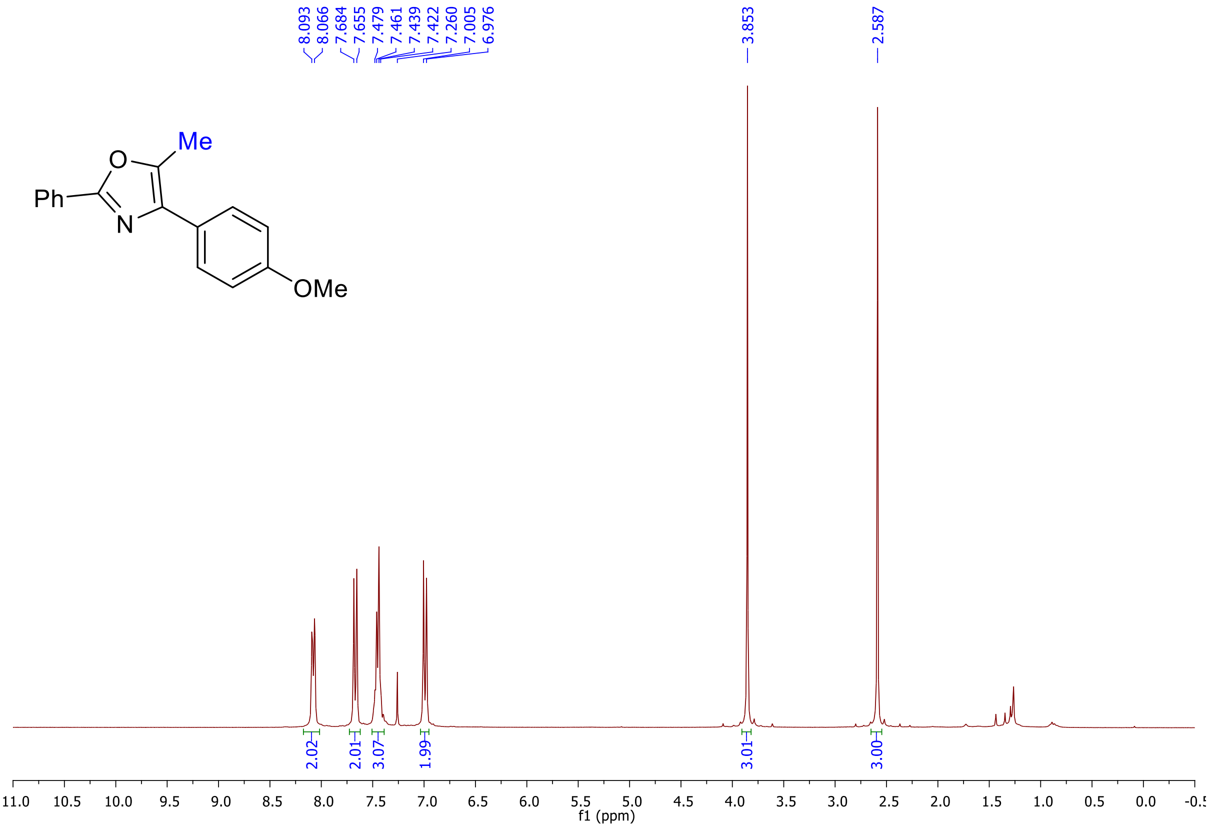

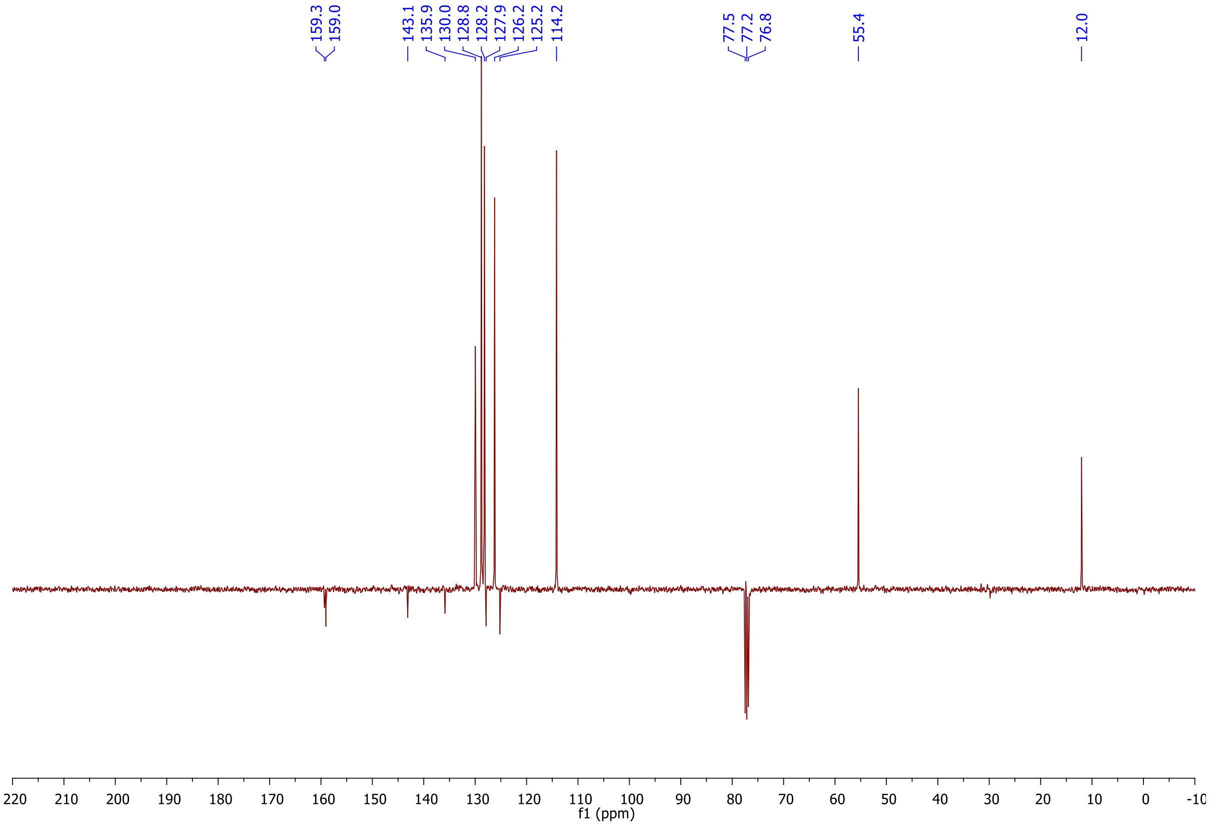

(*S*)-4-(3,4-Dimethoxyphenyl)-5-(ethylthio)-2-(pyrrolidin-2-yl)oxazole (6a) in $CDCl_3$ ^1H-NMR and ^{13}C-NMR (Pendant)

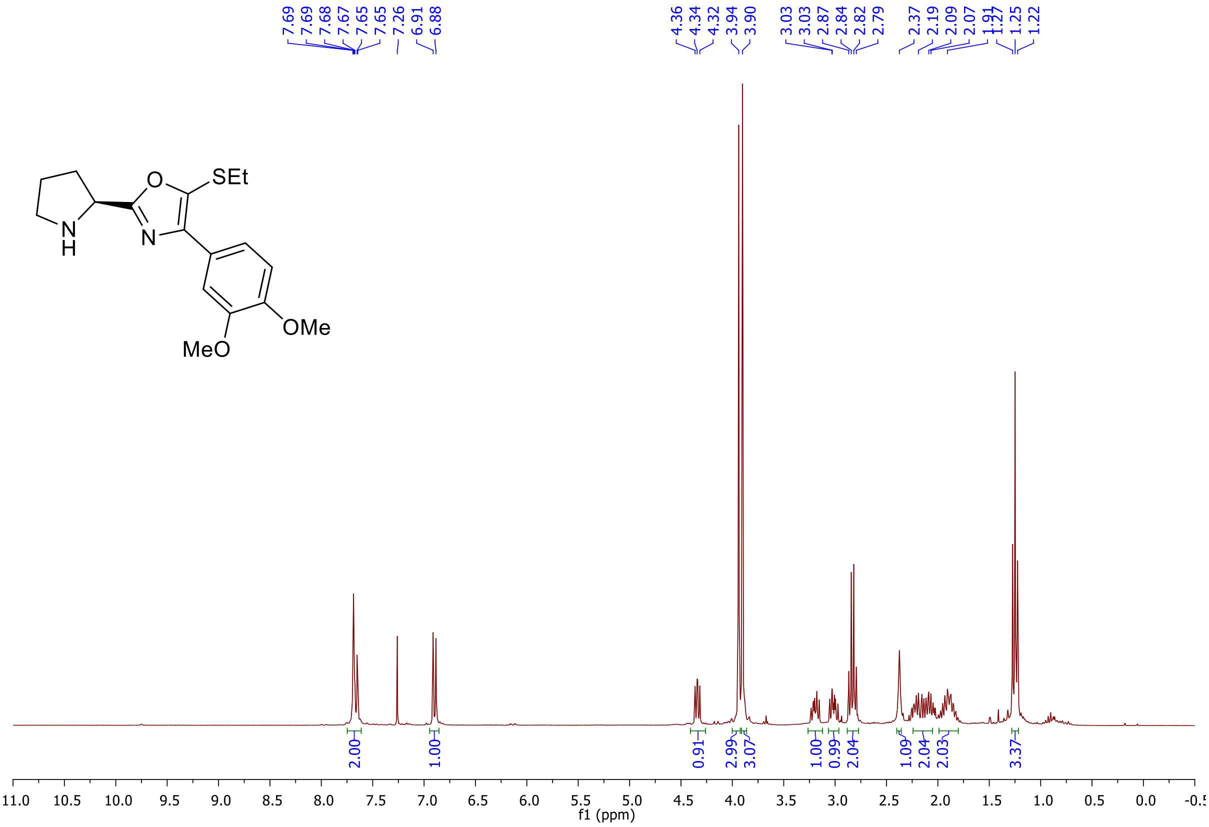

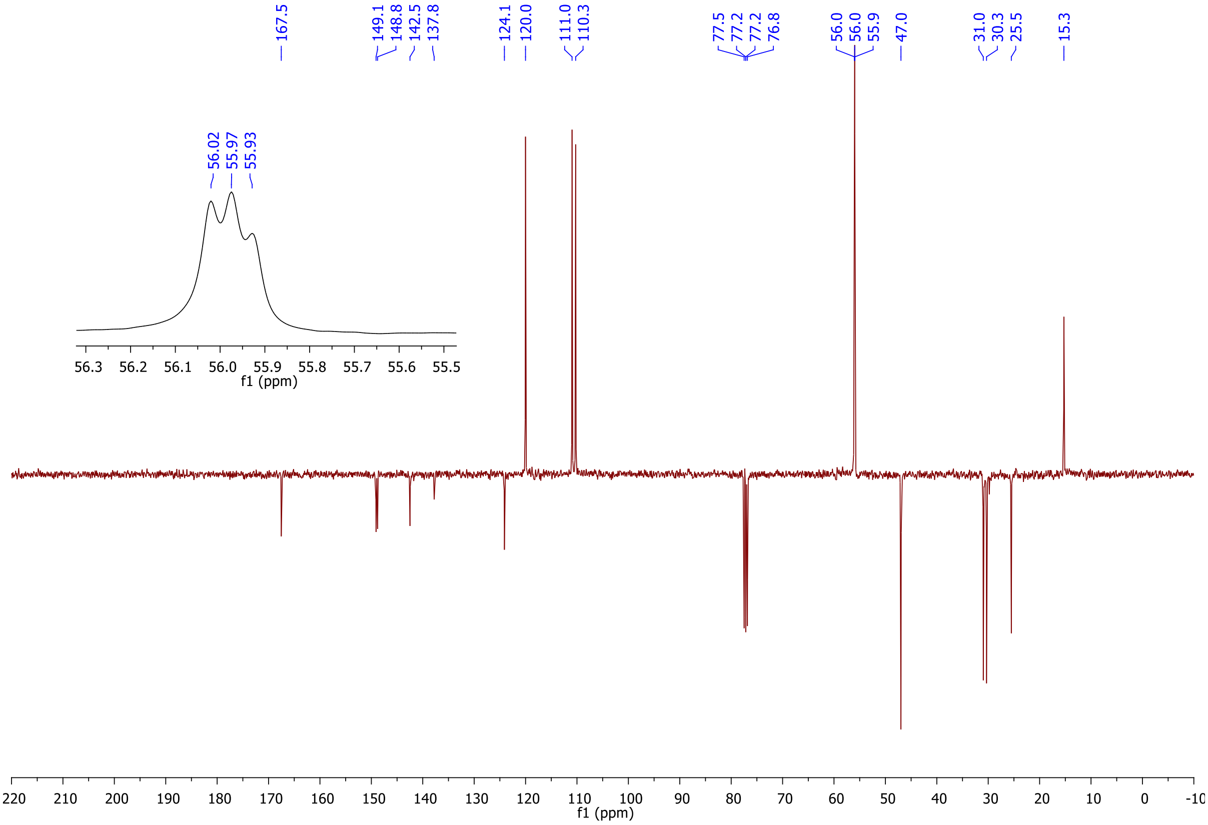

2-[(*2S*,*4R*)-4-(Benzyloxy)pyrrolidin-2-yl]-4-(3,4-dimethoxyphenyl)-5-(ethylthio)oxazole (6b) in $CDCl_3$
^{1}H-NMR and ^{13}C-NMR (Pendant)

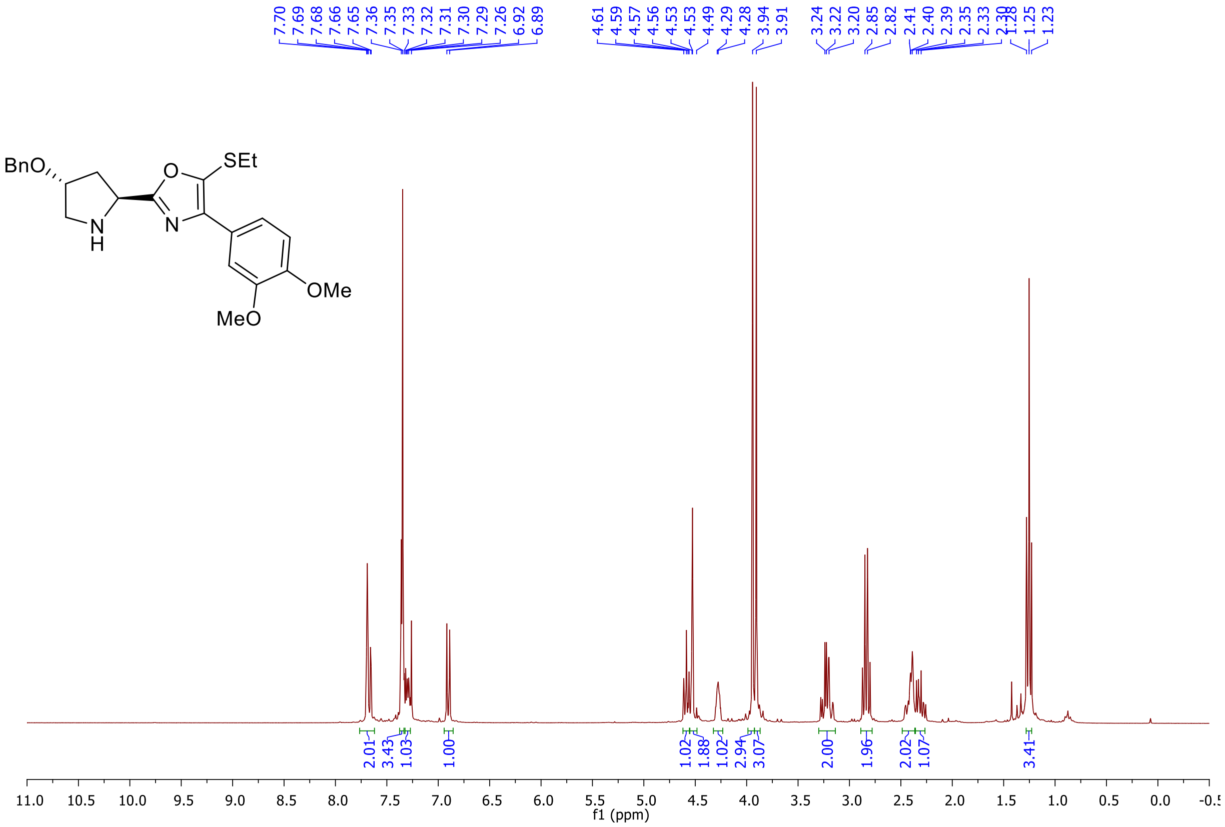

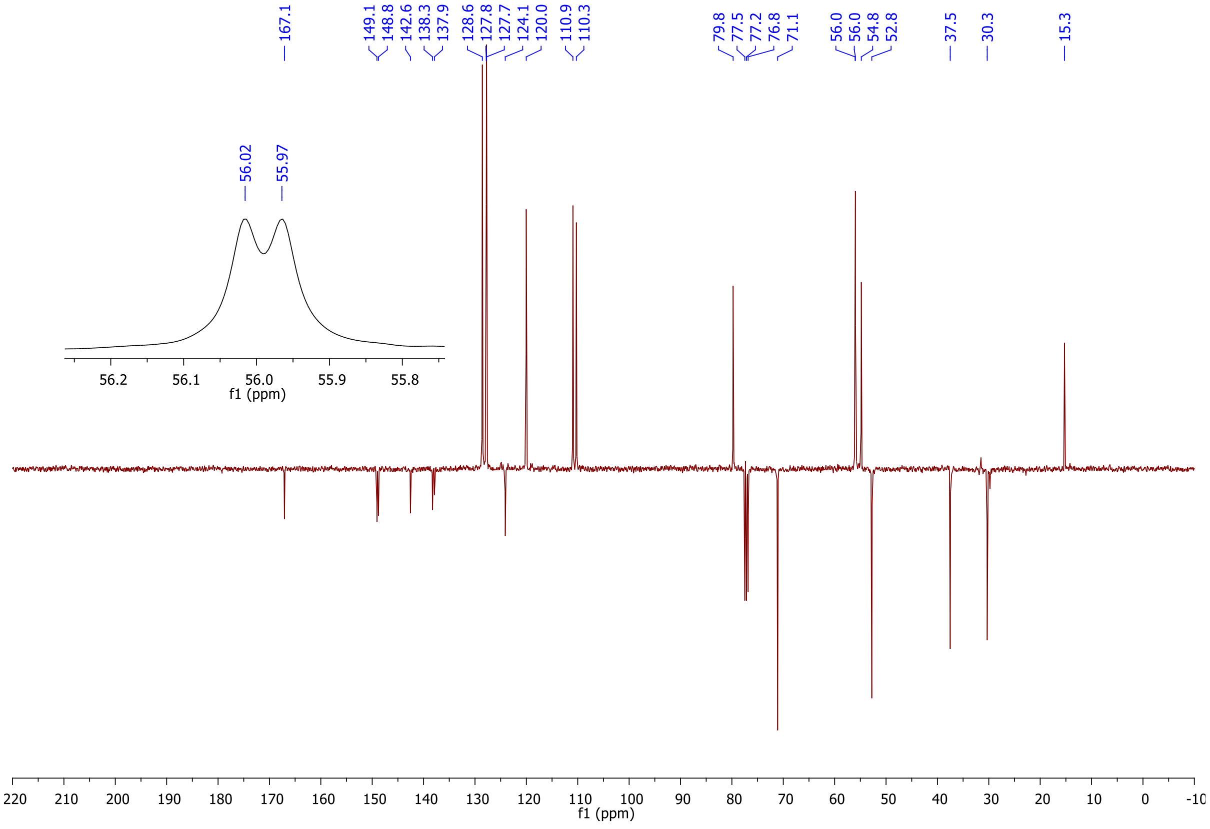

Supplement: Supplementary file 1 — Supplementary [file ANIE-56-13310-s001.pdf]
